# Supplementary material for: Unravelling the developmental and functional significance of an ancient Argonaute duplication
Source: Nat Commun. 2020 Dec 3;11:6187. doi: 10.1038/s41467-020-20003-8 (PMC7713132; doi:10.1038/s41467-020-20003-8)
Supplement: Supplementary file 1 — Supplementary Information [file 41467_2020_20003_MOESM1_ESM.pdf]

Supplementary information

Unravelling the developmental and functional significance of an ancient Argonaute duplication

Arie Fridrich[1,3], Vengamanaidu Modepalli[1,2,3], Magda Lewandowska[1], Reuven Aharoni[1] and Yehu Moran[1,*]

[1]Department of Ecology, Evolution and Behavior, Alexander Silberman Institute of Life Sciences, Faculty of Science, The Hebrew University of Jerusalem, 9190401 Jerusalem, Israel.

[2]The Marine Biological Association of the United Kingdom, Citadel Hill, Plymouth, United Kingdom.

[3]These authors contributed equally to this work.

*corresponding author: yehu.moran@mail.huji.ac.il

Content:

Supplementary Figure. 1: NveAGO1 and NveAGO2 knockdowns affect *Nematostella* development.

Supplementary Figure. 2: NveAGO1 and NveAGO2 knockdowns exhibit distinct transcriptomic signatures.

Supplementary Figure. 3: NveAGO2 is a carrier of previously annotated endo-siRNAs.

Supplementary Figure. 4: miRNA expression in distinct developmental stages and the *Nematostella* AGOs expression profile.

Supplementary Figure. 5: Correlation of miRNA counts in AGO-IP.

Supplementary Figure. 6: miRNAs exhibiting alternative strand selection in NveAGO1 and NveAGO2.

Supplementary Figure. 7: NveAGO knockdown effect on *Nematostella* strand selection and overall miRNA levels.

Supplementary Figure. 8: NveAGO1 and NveAGO2 miRNAs methylation levels in *Nematostella***.**

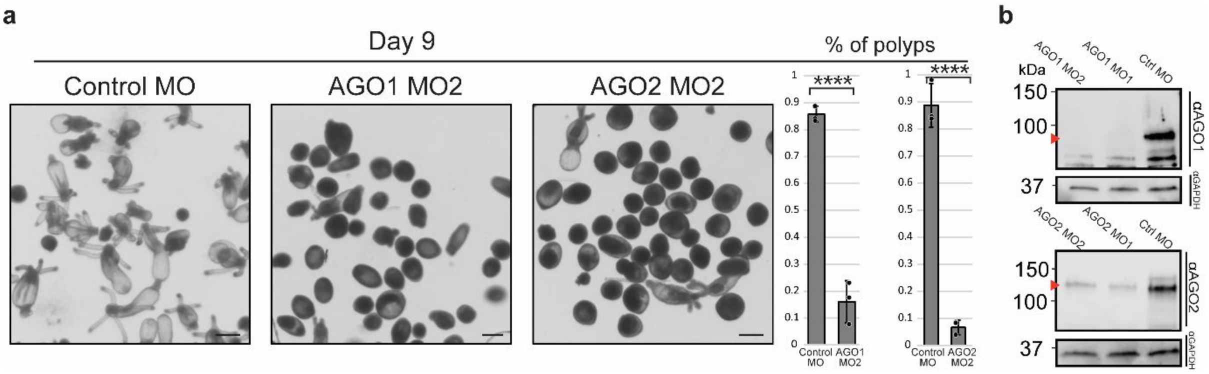

<u>Supplementary Fig. 1: NveAGO1 and NveAGO2 knockdowns affect *Nematostella* development.</u> **a,** Animals injected with NveAGO1 and NveAGO2 MOs 9 dpf. Majority of NveAGO1 and NveAGO2 depleted animals did not reach primary polyp stage (right upper and lower panels, respectively) at 9 dpf, three independent biological replicates. ****P= 0.000068, ****P= 0.000038 respectively (one tailed Student's *t*-test). Data are presented as mean values +/- SD. Scale bars are 250 μm. **b**, Western blot validation of knockdowns with NveAGO1 and NveAGO2 custom antibodies on extracts from 3 days old planulae. GAPDH antibody (Abcam, USA) was used as a loading control. Western blot validations were carried out twice independently.

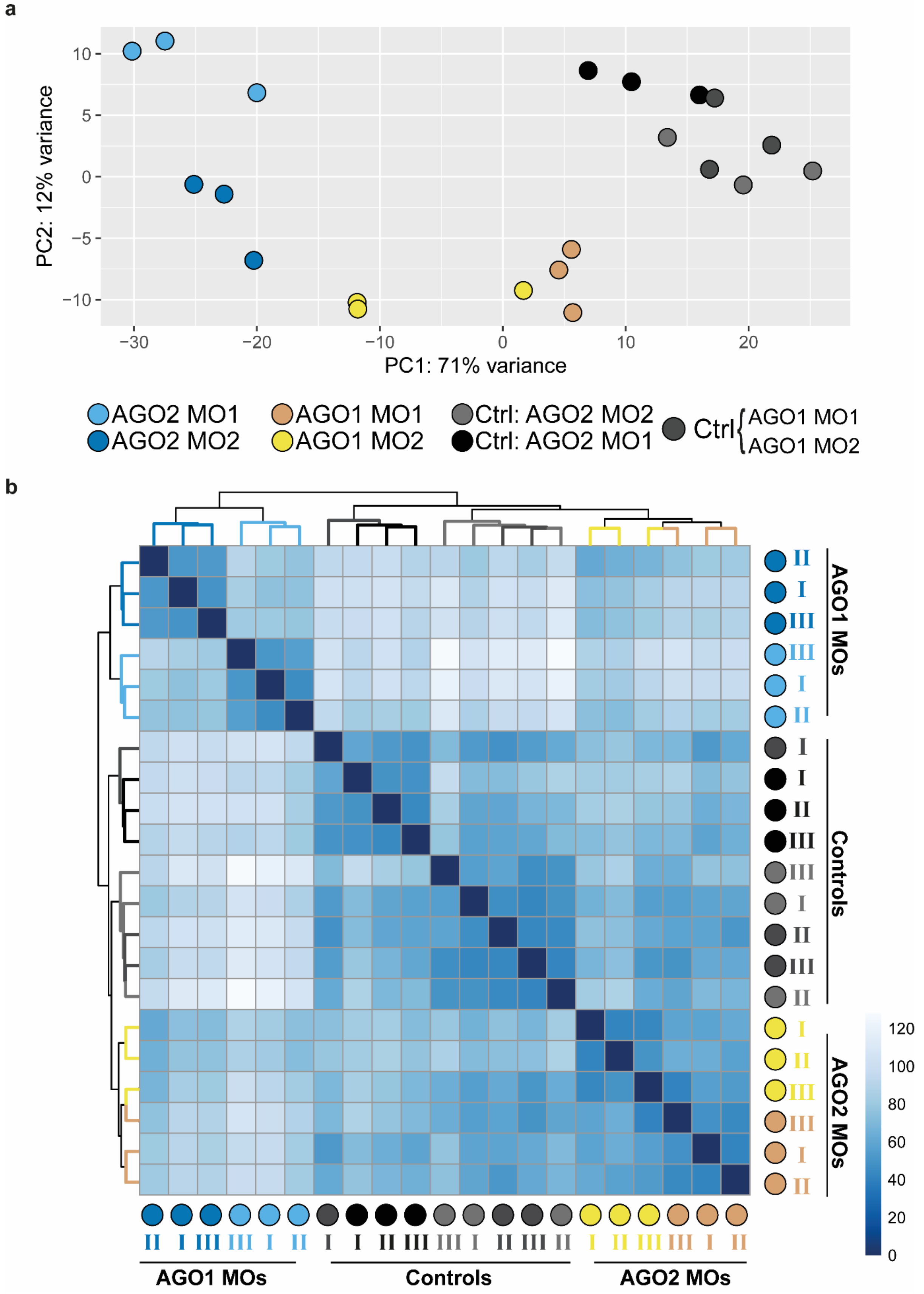

Supplementary Fig. 2: NveAGO1 and NveAGO2 knockdowns exhibit distinct transcriptomic signatures. a, Principal component analysis (PCA) plot and dendrogram (**b**) exhibiting differences in transcriptomic signatures for 3 distinct biological replicates of NveAGO1 and NveAGO2 knockdowns

(two different MOs for each NveAGO). NveAGO1 MO1 and NveAGO1 MO2 were injected in parallel to the same control MO. TopHat2[1] was used to align reads to the *Nematostella* genome.

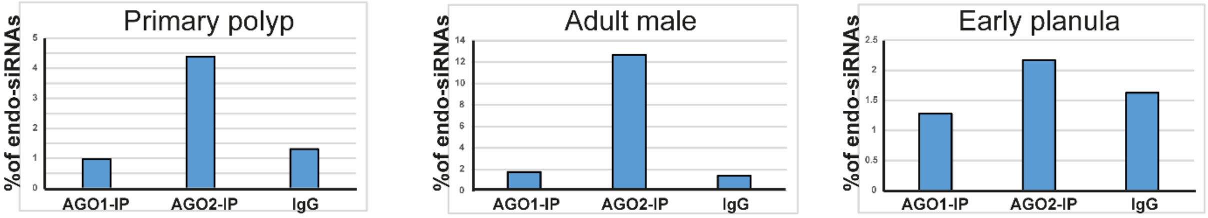

Supplementary Fig. 3: NveAGO2 is a carrier of previously annotated endo-siRNAs. a, Normalized read-counts of miRNAs (lower panels) and endo-siRNAs (upper panels) in sRNA libraries generated from *Nematostella* AGO IPs. Each bar represents the average of two distinct biological replicates normalized as described in the methods section. endo-siRNA sequences were taken from Calcino et al.[2].

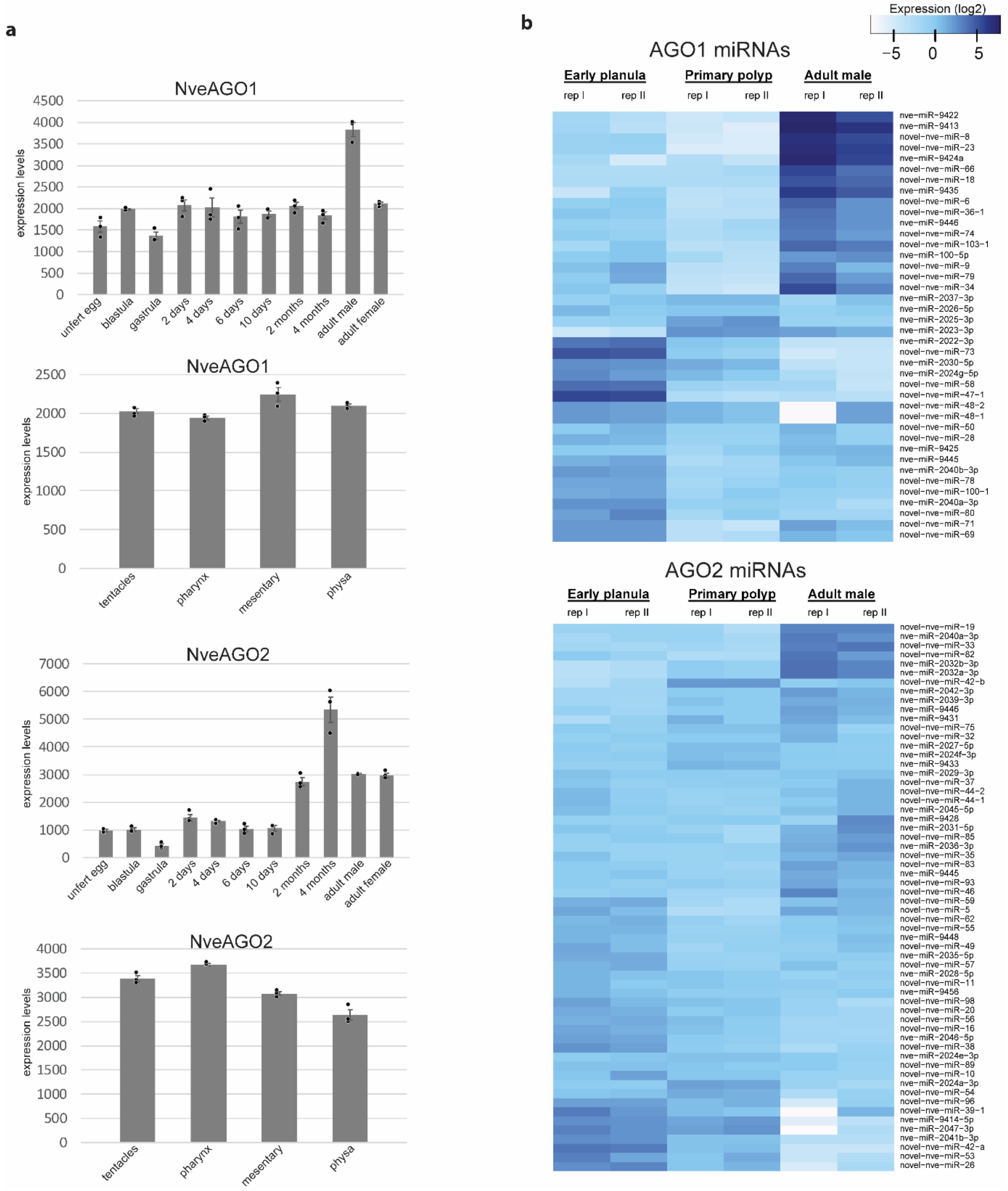

Supplementary Fig. 4: miRNA expression in distinct developmental stages and the *Nematostella* AGOs expression profile.

a, Expression of *Nematostella* AGOs throughout development and in distinct tissues. Data are presented as mean values +/- SD. Nanostring data to generate this representation (**Supplementary data 5**) was taken from Praher et al[3]. **b**, Heatmap representing $\log_2$ expression levels of known and novel miRNAs. Counts per Million (CPM) from two distinct biological replicates of each NveAGO-IP were used to generate this heatmap.

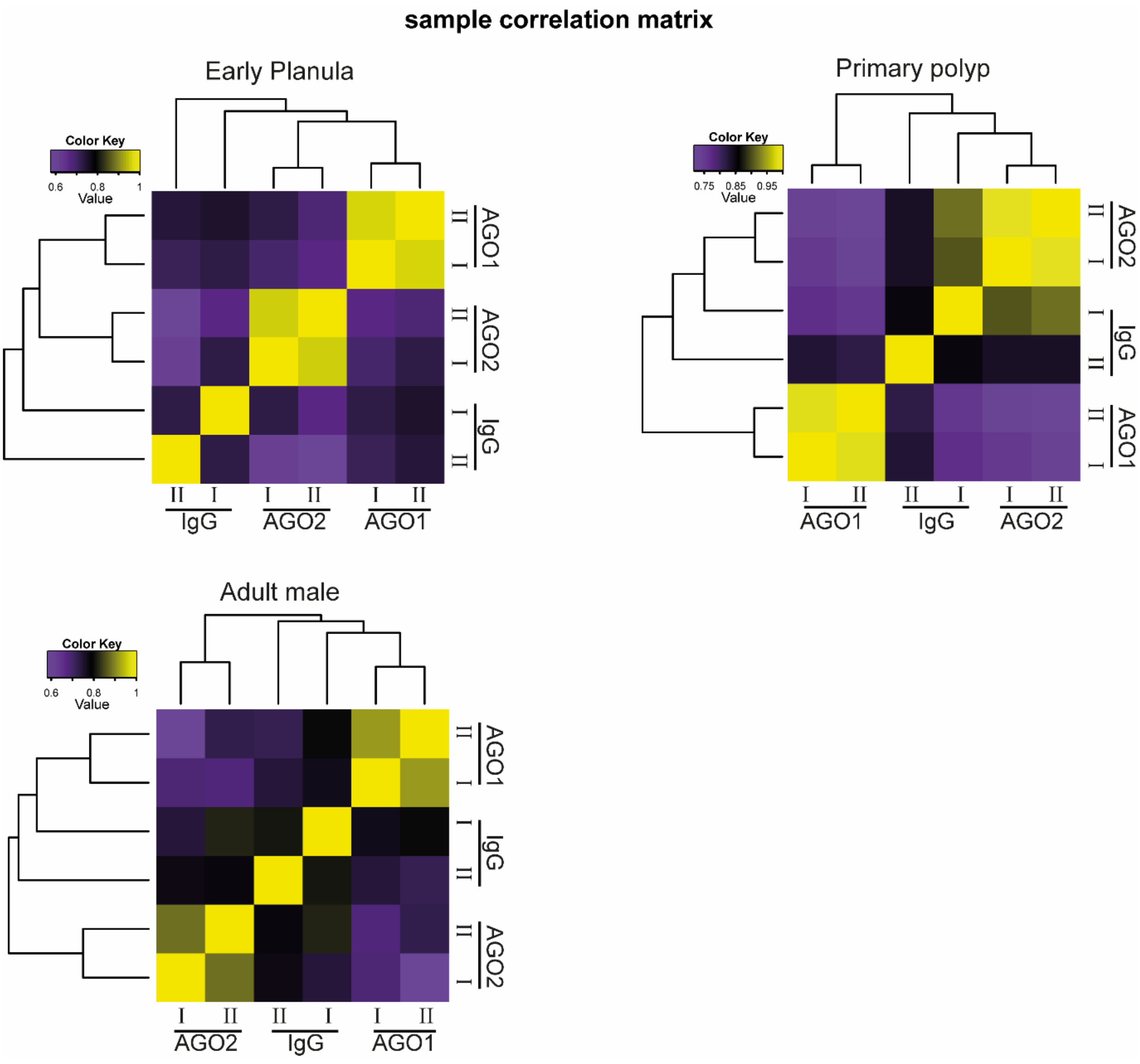

Supplementary Fig. 5: Correlation of miRNA counts in AGO-IP.

Pearson correlation heatmap of miRNA levels in IgG-IP, NveAGO1-IP and NveAGO2-IP duplicates from three developmental stages.

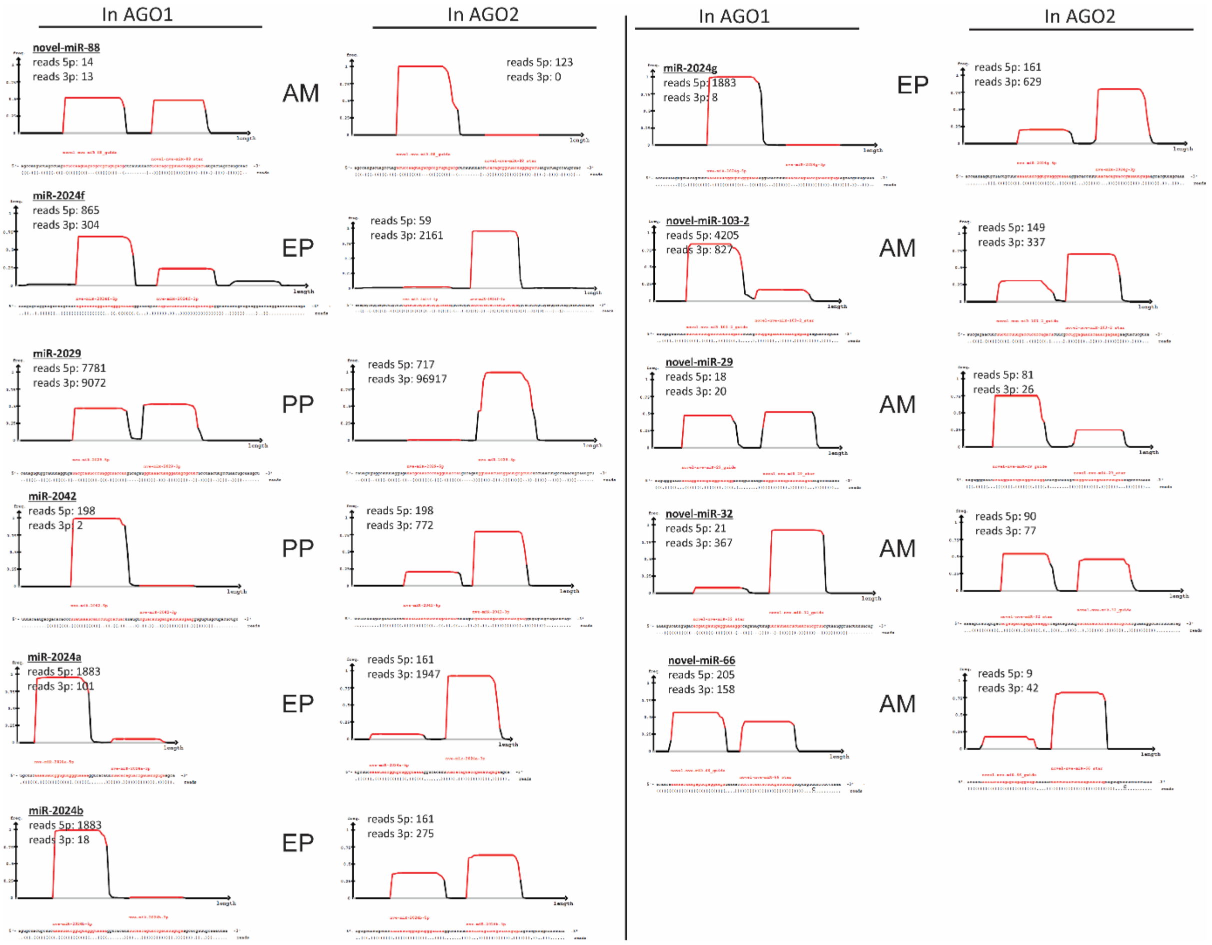

Supplementary Fig. 6: miRNAs exhibiting alternative strand selection in NveAGO1 and NveAGO2.

Eleven miRNAs exhibit alternative profiles of strand selection depending on their hosting AGO. Each pair represents miRNA signature as observed in NveAGO1 IP (left columns) or in NveAGO2 IP (right columns). The graphic outputs were generated using mirDeep2[4]. Abbreviations: AM, Adult male; EP, Early planula; PP, Primary polyp.

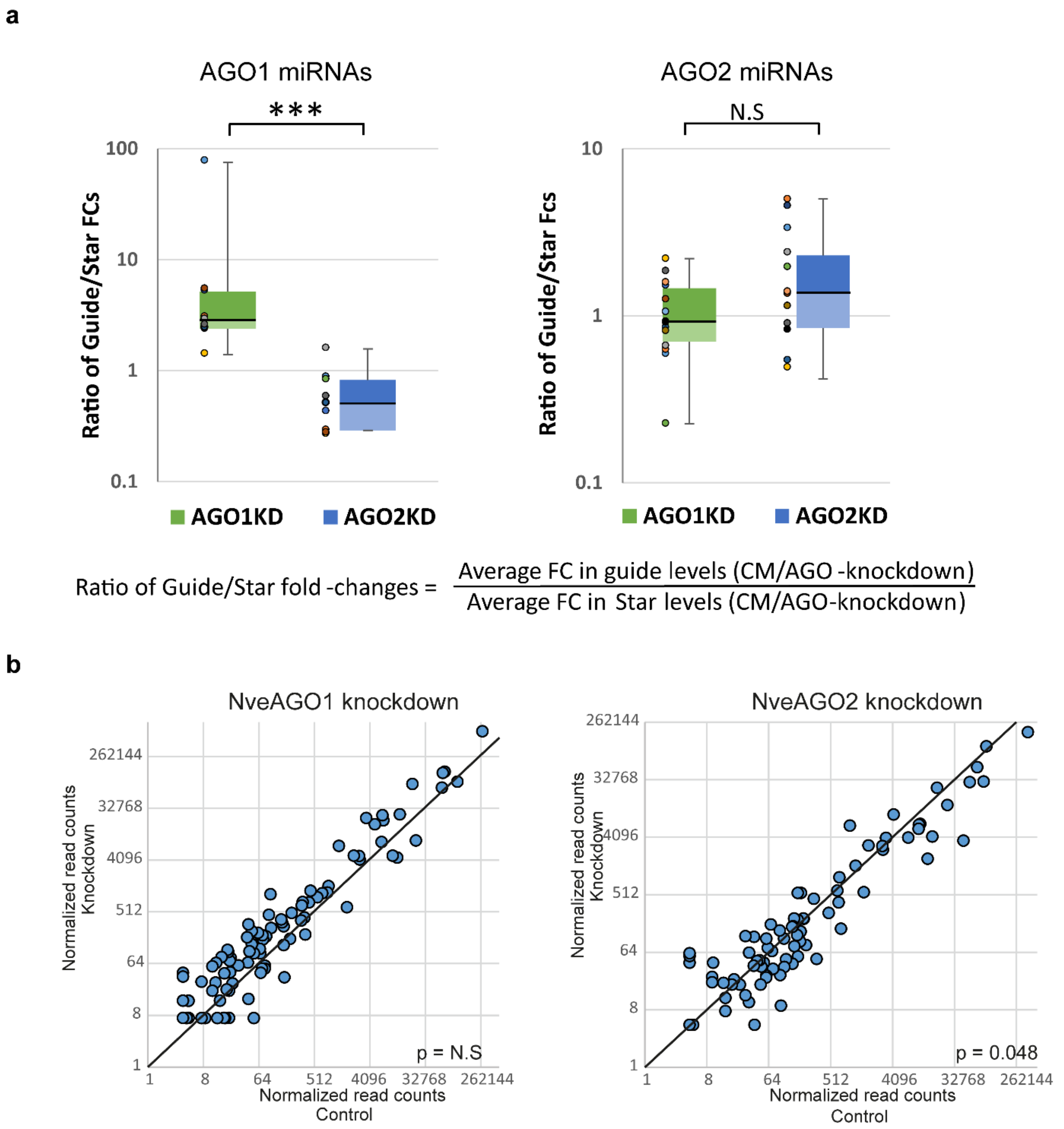

Supplementary Fig. 7: NveAGO knockdown effect on *Nematostella* strand selection and overall miRNA levels.

a, Ratio between the average guide fold-change to average star fold-change of NveAGO1 miRNAs in NveAGO knockdowns (left panel) and NveAGO2 miRNAs in NveAGO knockdown (right panel). The reads mapped to individual miRNAs were normalized using spike-ins and average reads were generated from biological triplicates (**Supplementary data 6**). Weakly expressed miRNAs (less than 50 read counts for an individual miRNA) and miRNA with no star reads were excluded from this analysis. Next we calculated the ratio between the average guide fold-change to the average star fold-change (lower panel). NveAGO1 enriched miRNAs are significantly affected in NveAGO1 KD (P=0.00029, one sided Mann-Whitney U Test). NveAGO2 enriched miRNAs did not exhibit a

significant change in NveAGO2 KD (P=0.15, one sided Mann-Whitney U Test). Internal black lines inside the box plots indicate median values. Boxes bounds include data between 25th and 75th percentiles. Top whiskers: 75th percentile plus 1.5IQR. Bottom whiskers: 25th percentile minus 1.5IQR. **b,** Each point represents the average normalized expression levels of an individual miRNA from three independent biological replicates in Control and AGO1 KD (p=0.058, one tailed Student's t-test, left panel) and control and AGO2 KD (p=0.048, one tailed Student's t-test, right panel).

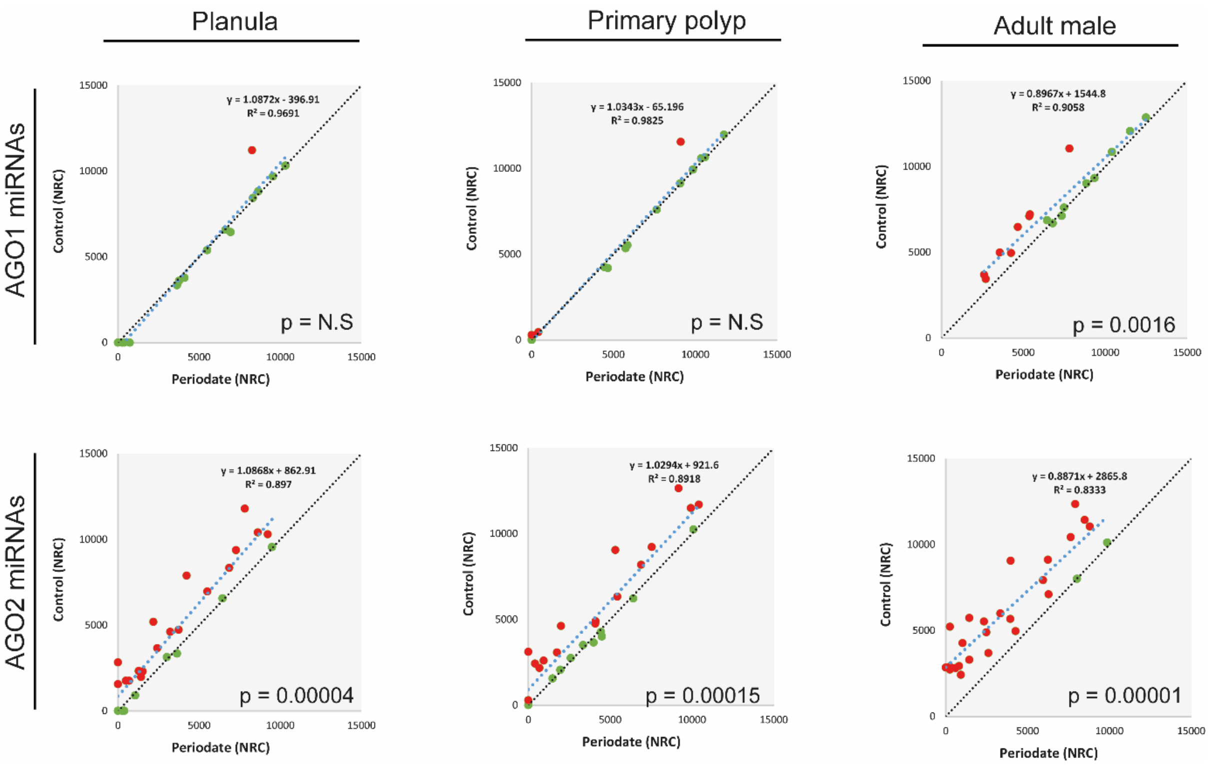

<u>Supplementary Fig. 8: NveAGO1 and NveAGO2 miRNAs methylation levels in *Nematostella.*</u> Scatter plots presenting the change of normalized read counts of individual AGO1 and AGO2 miRNAs in Control and Periodate treated libraries. In red, miRNA that dropped more than 1.3-fold. Significance was calculated using two-tailed Student's t-test for two dependent means.

References

1 Kim, D. *et al.* TopHat2: accurate alignment of transcriptomes in the presence of insertions, deletions and gene fusions. *Genome biology* **14**, R36, doi:10.1186/gb-2013-14-4-r36 (2013).

2 Calcino, A. D., Fernandez-Valverde, S. L., Taft, R. J. & Degnan, B. M. Diverse RNA interference strategies in early-branching metazoans. *BMC Evol Biol* **18**, 160, doi:10.1186/s12862-018-1274-2 (2018).

3 Praher, D. *et al.* Characterization of the piRNA pathway during development of the sea anemone Nematostella vectensis. *RNA Biol* **14**, 1727-1741, doi:10.1080/15476286.2017.1349048 (2017).

4 Friedlander, M. R., Mackowiak, S. D., Li, N., Chen, W. & Rajewsky, N. miRDeep2 accurately identifies known and hundreds of novel microRNA genes in seven animal clades. *Nucleic acids research* **40**, 37-52, doi:10.1093/nar/gkr688 (2012).
