# Supplementary material for: Unravelling the developmental and functional significance of an ancient Argonaute duplication
Source: Nat Commun. 2020 Dec 3;11:6187. doi: 10.1038/s41467-020-20003-8 (PMC7713132; doi:10.1038/s41467-020-20003-8)
Supplement: Supplementary file 3 — Reporting Summary [file 41467_2020_20003_MOESM3_ESM.pdf]

nature research

Corresponding author(s): Yehu Moran
Last updated by author(s): Oct 20, 2020

Reporting Summary

Nature Research wishes to improve the reproducibility of the work that we publish. This form provides structure for consistency and transparency in reporting. For further information on Nature Research policies, see our Editorial Policies and the Editorial Policy Checklist.

Statistics

For all statistical analyses, confirm that the following items are present in the figure legend, table legend, main text, or Methods section.

n/a	Confirmed	
☐	☒	The exact sample size (*n*) for each experimental group/condition, given as a discrete number and unit of measurement
☐	☒	A statement on whether measurements were taken from distinct samples or whether the same sample was measured repeatedly
☐	☒	The statistical test(s) used AND whether they are one- or two-sided<br>*Only common tests should be described solely by name; describe more complex techniques in the Methods section.*
☐	☒	A description of all covariates tested
☐	☒	A description of any assumptions or corrections, such as tests of normality and adjustment for multiple comparisons
☐	☒	A full description of the statistical parameters including central tendency (e.g. means) or other basic estimates (e.g. regression coefficient) AND variation (e.g. standard deviation) or associated estimates of uncertainty (e.g. confidence intervals)
☐	☒	For null hypothesis testing, the test statistic (e.g. *F*, *t*, *r*) with confidence intervals, effect sizes, degrees of freedom and *P* value noted<br>*Give P values as exact values whenever suitable.*
☒	☐	For Bayesian analysis, information on the choice of priors and Markov chain Monte Carlo settings
☐	☒	For hierarchical and complex designs, identification of the appropriate level for tests and full reporting of outcomes
☒	☐	Estimates of effect sizes (e.g. Cohen's *d*, Pearson's *r*), indicating how they were calculated

Our web collection on statistics for biologists contains articles on many of the points above.

Software and code

Policy information about availability of computer code

Data collection	Xcalibur version 4.0
Data analysis	-miRNA denovo identification and quantification: mirDeep2 version 2.0.0.8<br>-Phylogeny: PhyML version 20120412<br>-Phylogeny: MrBayes version 3.2.1<br>-Differential expression analysis: DEseq2 in Galaxy version 2.11.39<br>-Heatmap generation was made using R gplots version 3.0.0. Data was log2 transformed and median centered.<br>-Semi-quantitative LC-MS/MS analysis: MaxQuant version 1.5.3.1254<br>-Weblogo version 3.7.4<br>-Transcriptome, counts: featureCounts version 1.6.0.2<br>-endo-siRNA identification: ShortStack version 3.8.5<br>-miRNA origin from targets analysis:We used our own custom code. the code is available at: https://github.com/ArieFridrichHuji/miRNA_Origin.git<br>-RNAseq reads alignment to Nematostella genome: TopHat Version 2.1.1<br>-RNAseq reads alignment to Nematostella genome: STAR Version 2.7.3a<br>-Indexing Nematostella genome: Bowtie2 version 2.3.4.1

For manuscripts utilizing custom algorithms or software that are central to the research but not yet described in published literature, software must be made available to editors and reviewers. We strongly encourage code deposition in a community repository (e.g. GitHub). See the Nature Research guidelines for submitting code & software for further information.

April 2020

Data

Policy information about availability of data

All manuscripts must include a data availability statement. This statement should provide the following information, where applicable:

- Accession codes, unique identifiers, or web links for publicly available datasets
- A list of figures that have associated raw data
- A description of any restrictions on data availability

The datasets generated during the current study are available in the GEO, SRA, and ProteomeXchange Consortium repositories under the identification numbers: GSE144203, PRJNA658931 and PXD011644 respectively.

Field-specific reporting

Please select the one below that is the best fit for your research. If you are not sure, read the appropriate sections before making your selection.

☒ Life sciences ☐ Behavioural & social sciences ☐ Ecological, evolutionary & environmental sciences

For a reference copy of the document with all sections, see nature.com/documents/nr-reporting-summary-flat.pdf

Life sciences study design

All studies must disclose on these points even when the disclosure is negative.

Sample size

a. Morpholino microinjections for small-RNA sequencing: RNA was collected from 12 injected groups of ~150 early planulae in each. Three distinct biological AGO1 knockdown groups with their three corresponding control groups and three distinct biological AGO2 knockdown groups with their three corresponding control groups. As it was essential that each group would originate from the same parents and be injected in the same day to minimize biological and technical variability within each group, 150 was the highest feasible number of zygotes. Collection from ~150 early planulae per group provided enough RNA for all downstream applications.

b. Morpholino microinjections for transcriptome sequencing: RNA was collected from 21 injected groups of ~150 early planulae in each. The groups aere prepared as follows:
- AGO1 knockdowns: 3 distinct biological replicates. each biological replicate is comprised of 3 injected groups: AGO1 MO1, AGO1 MO2 and Control, together comprising 9 groups.
- AGO2 knockdowns:
(1) For AGO2 MO1: 3 distinct biological replicates. each biological replicate is comprised of 2 injected groups: AGO2 MO1 and control, together comprising 6 groups.
(2) For AGO2 MO2: 3 distinct biological replicates. each biological replicate is comprised of 2 injected groups: AGO2 MO2 and control, together comprising 6 groups.

As it was essential that each group would originate from the same parents and be injected in the same day to minimize biological and technical variability within each group, 150 was the highest feasible number of zygotes.
Collection from ~150 early planulae per group provided enough RNA for all downstream applications.

In total these groups comprise 21 samples from which RNA was extracted for downstrean sequencing of their transcriptomes.

c. AGO IP: RNA was collected from 12 immunorecipitated samples of animals that correspond to 100 µl of volume in each: two distinct biological samples for each AGO-IP, for control IgG-IP from three developmental stages: early planulae, primary polyps and adult males. This amount was sufficient for the downstream experiments as the RNA amounts in the immunoprecipitated samples were sufficient for small-RNA library preparation, and the amounts of protein in the immunoprecipitated samples were sufficient for identification by western blots.

Data exclusions

No data was excluded from the analysis.

Replication

a. AGO knockdown experiments for small-RNA sequencing were performed on three distinct biological replicates from groups of animals that spawned on different days. This amount was designed for generation an optimal amount of twelve libraries to be sequenced on the Illumina NextSeq 500 platform on a single flow-cell with a sufficient depth.
b. AGO knockdown experiments for transcriptome sequencing were performed on three distinct biological replicates from groups of animals that spawned on different days. This amount was designed for generation an optimal amount of twelve libraries to be sequenced on the Illumina NextSeq 500 platform on a single flow-cell with a sufficient depth.
c. AGO IP experiments were generated on two distinct biological replicates for each AGO and IgG in each of the three developmental stages. This amount of replicates was designed to generate an optimal amount of twelve libraries to be high-throughput sequenced on a single flowcell.
Additionally this amount is a result of a limited amount of these custom antibodies that will be required for future experiments.

All attemps of replication were successful.

Randomization

Randomization was achieved as all groups were treated in the experimental and computational workflow in parallel without taking into account their identity. Animals were never chosen specifically for each of the experimental groups.

Blinding

Practically due to the method of raising the animals there was no possibility for true blinding of the investigator. In order to account for this limitation each analysis was supervised by at least one additional author to ensure no bias.

Reporting for specific materials, systems and methods

We require information from authors about some types of materials, experimental systems and methods used in many studies. Here, indicate whether each material, system or method listed is relevant to your study. If you are not sure if a list item applies to your research, read the appropriate section before selecting a response.

Materials & experimental systems

n/a	Involved in the study
☐	☒ Antibodies
☒	☐ Eukaryotic cell lines
☒	☐ Palaeontology and archaeology
☐	☒ Animals and other organisms
☒	☐ Human research participants
☒	☐ Clinical data
☒	☐ Dual use research of concern

Methods

n/a	Involved in the study
☒	☐ ChIP-seq
☒	☐ Flow cytometry
☒	☐ MRI-based neuroimaging

Antibodies

Antibodies used

NveAGO1-IP: Custom polyclonal antibodies generated in rabbits against recombinant fragments corresponding to the C-terminal region:
QVGQEQRHTYLPLEVCNIVPGQRCVKKLTDTQTSKMIRATARSAPDREREIRGLVKKANFDEDAYVKDFSISIGKNMVELQGRVLPPPKLVYGGKQSS
PITPKGGVWDMRGRQLFHGIEIRTWAIACFVKQQMCTEDSLRRFSNQLMKISVEQGMPISCPPVFFRYARNPDEVERMFRRLKEAHPDLQMILVUL
PGKTP (GenScript, USA).
NveAGO2-IP: Custom polyclonal antibodies generated in rabbits against recombinant fragments corresponding to the N-terminal region:
MPKKSKGRGRGRGRGNPHHEKOQTLVGQQATSRNNEHKQLPKPTQTQQETSLSQQPYTCSSAAEAQGPLTPPNNTQGGSLTNAEPAQADTLGQ
KFETQLNLSPQSGKEQGAIPKTGARLKGNSLAPGSQGNGQFSSKNLLAQMQRTPRSASESQNEASSKSQQAHNNQSQAAAHQQTQAGPQQTPAR
SQQT (GenScript, USA).
Control IgG-IP: Sigma. Catalog number: I5006-10G. LOT: SLBM2617V
Secondary antibodies for Western-blot validation :
A. Jackson's immunoassays. Peroxidase-AffinitiPure Goat Anti-Rabbit IgG (H+L) (min X Hu, Ms, Rat Sr prot). Catalog number: 111-035-144. LOT: 123520

Validation

Each recombinant fragment was injected into three rabbits. After the first round of immunization, pre-immune and post-immune sera were used for screening by Western blot against Nematostella lysate to screen which of the sera provided the band that corresponds to the specific sizes of NveAGO1 and NveAGO2 (~96 and ~122 kDa respectively). Finally, the antigens were used by the company for affinity purification of the antibodies from the relevant rabbits. Later, the selected antibodies were further validated by morpholino-based knockdown of NveAGO1 or NveAGO2.
IgG from rabbit sera was applied as a negative control. From our experience no specific bands are visible when used on any developmental stage of Nematostella vectensis. No specific reactivity was documented for this reagent with N. vectensis extract in our experiments towards the Argonaute proteins. According to the manufacturer's declaration the purity of this reagent is 95% or more as assayed by SDS-PAGE.

Animals and other organisms

Policy information about studies involving animals; ARRIVE guidelines recommended for reporting animal research

Laboratory animals

The sea anemone Nematostella vectensis from the common lab strain which was used for genome sequencing in 2007 (Putnam et al. Science). This line originated from Rhode River, MD, USA, but is maintained in many labs around the world for more than a decade. Both male and female sea anemones were used in this study. Early developmental stages were used (first 9 days post fertilization) as well as adults (four months) sea anemones.

Wild animals

The study did not involve wild animals

Field-collected samples

The study did not involve samples collected from the field

Ethics oversight

No ethical approval or guidance was required as Nematostella is an invertebrate with a very simple nervous system (simpler than a fruit fly).

Note that full information on the approval of the study protocol must also be provided in the manuscript.
