# Supplementary material for: Unravelling the developmental and functional significance of an ancient Argonaute duplication
Source: Nat Commun. 2020 Dec 3;11:6187. doi: 10.1038/s41467-020-20003-8 (PMC7713132; doi:10.1038/s41467-020-20003-8)
Supplement: Supplementary file 4 — Description of Additional Supplementary Files [file 41467_2020_20003_MOESM4_ESM.pdf]

Description of Additional Supplementary Files

Supplementary Data 1: NveAGO-IP LC-MS/MS analysis.
Immunoprecipitations were carried out on primary polyps as described in the methods section using antibodies against NveAGO1 and NveAGO2. Immunoprecipitation with IgG was carried out as a negative control. Statistical analysis was performed on three technical replicates using the Perseus statistical analysis package. NveAGO1 and NveAGO2 gene models are NVE8924 and NVE1871, respectively.

Supplementary Data 2: **Transcriptomes of NveAGO1 and NveAGO2 knockdowns.**
The file contains read counts of *Nematostella* transcripts before and after knockdowns of NveAGO1 and NveAGO2. Knockdowns of each NveAGO were carried out with two distinct MOs. For each MO, the experiment was carried out on 3 distinct biological replicates. The reads were aligned to the genome using STAR and TOPHAT and the number of reads of each Nematostella gene models was generated using featurecounts. Differential expression analysis was carried out to compare transcript levels in morphants to control groups of animals injected with a control MO. Differential expression analysis was carried out using DESeq2, as described in the methods section.

Supplementary Data 3: Sequences and structure of novel *Nematostella* miRNAs.
mirDeep2 outputs from sRNA sequencing of NveAGO1 and NveAGO2 IP samples.

Supplementary Data 4: sRNA sequencing data from NveAGO1 and NveAGO2 IPs.
Read counts and sequences of known and novel *Nematostella* miRNAs and endo-siRNAs.

Supplementary Data 5: Expression of *Nematostella* AGOs throughout development and in distinct tissues.
Nanostring data was taken from Praher et al. Characterization of the piRNA pathway during development of the sea anemone *Nematostella vectensis*. RNA Biol 14, 1727-1741 doi:10.1080/15476286.2017.1349048 (2017).

Supplementary Data 6: NveAGO1 and NveAGO2 knockdown effects on miRNAs read counts and animal morphology.
The file contains read counts of miRNAs in NveAGO1 and NveAGO2 knockdowns (sheet "normalized to spike x1M": one tailed Student's t-test, sheet "90%AGO-preference_miRNAs": two-tailed binomial significance test, sheet "strand-selection": one sided Mann-Whitney U Test), as well as numbers of animals that managed develop into primary polyps (three independent biological replicates, one tailed Student's t-test).

Supplementary Data 7: NveAGO1 and NveAGO2 miRNAs methylation levels in *Nematostella*.
Normalized read counts of individual AGO1 and AGO2 miRNAs in control and periodate treated libraries. Significance was calculated using two-tailed Student's *t*-test for two dependent means.

Supplementary Data 8: **Extended homology between miRNA precursors and their targets**.

Number of precursors with extended homology was calculated for the list of 138 miRNAs, and an average for 50 lists with shuffled sequences of the guide stand's flanking regions.

Supplementary Data 9: **extended alignments of miRNAs to their targets**.
miRNA putative targets were aligned to the extended guide sequences using the Smith-Waterman algorithm with -gapopen set to 15 and -gapextend set to 2.0. miRNA-target pairs with extended homology of at least six consecutive nucleotides are presented.

Supplementary Data 10 Protein sequences of AGOs. Protein sequences that were used to construct the phylogeny presented in Figure 1a.
