# Supplementary material for: Unravelling the developmental and functional significance of an ancient Argonaute duplication
Source: Nat Commun. 2020 Dec 3;11:6187. doi: 10.1038/s41467-020-20003-8 (PMC7713132; doi:10.1038/s41467-020-20003-8)
Supplement: Supplementary file 7 — Supplementary Data 3 [file 41467_2020_20003_MOESM7_ESM.pdf]

Novel *Nematostella* miRNAs

miRBase precursor : novel-nve-miR-5
Total read count : 208
novel-nve-miR-5_guide read count : 203
novel-nve-miR-5_star read count : 5
remaining reads : 0

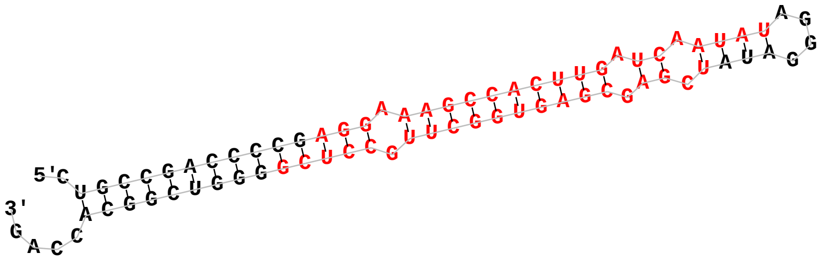

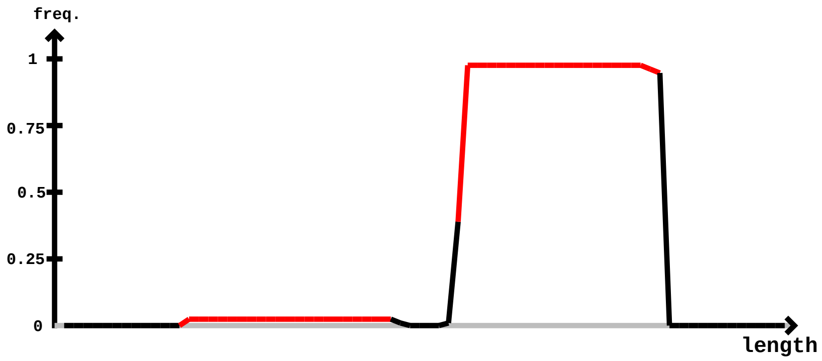

novel-nve-miR-5_star

novel-nve-miR-5_guide

```
5'- cugccgaccccgaggaaagccacuugaucaauauagggauaucgagcgaguggcuugccucggggucggcaccag   -3'    exp
    .(((((((((((((.((((((((((.((.((((....)))).)).))))))))))).)))))))))))))....    reads      mm         sample
    ............aggaaagccacuugaucaauau.........................................    1          0          seq
    ............aggaaagccacCugaucaauau.........................................    1          1          seq
    ............aggaaagccacuugaucaauauC........................................    1          1          seq
    ............aggaaagccacuugaucaauauU........................................    2          1          seq
    .......................................uaucgagcgaguggcuugUcu...............    1          1          seq
    .......................................uaucgagcgaUuggcuugccucg.............    1          1          seq
    ........................................aucgagcgaguggcuugccu...............    2          0          seq
    ........................................aucgagcgaguggcuugccuc..............    1          0          seq
    ........................................aucgagcgaUuggcuugccucg.............    3          1          seq
    ........................................aucgagcgaguggcuugccucU.............    56         1          seq
    ........................................aucgagcgaguggcuugccucg.............    5          0          seq
    ........................................aucgagcgaguggcuugccucA.............    5          1          seq
    ........................................aucgagcgaguggcuugccucC.............    7          1          seq
    .........................................ucgagcgaguggcuugccuc..............    2          0          seq
    .........................................ucgagcgaguggcuugccucU.............    88         1          seq
    .........................................ucgagcgaguggcuugccucC.............    16         1          seq
    .........................................ucgagcgaguggcuugccucg.............    3          0          seq
    .........................................ucgagcgaguggcuugccucA.............    13         1          seq
```

miRBase precursor : novel-nve-miR-6
Total read count : 1792
novel-nve-miR-6_guide read count : 1432
novel-nve-miR-6_star read count : 213
remaining reads : 147

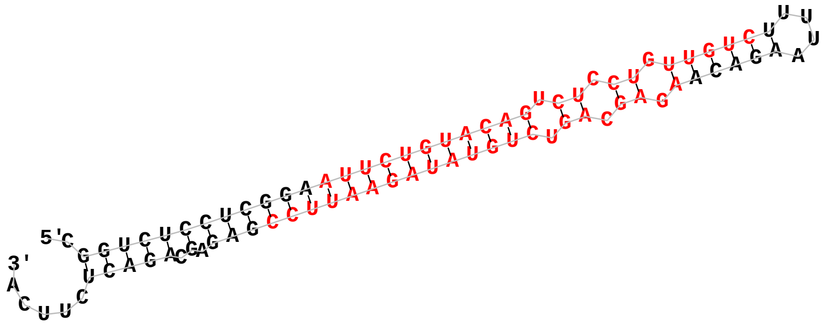

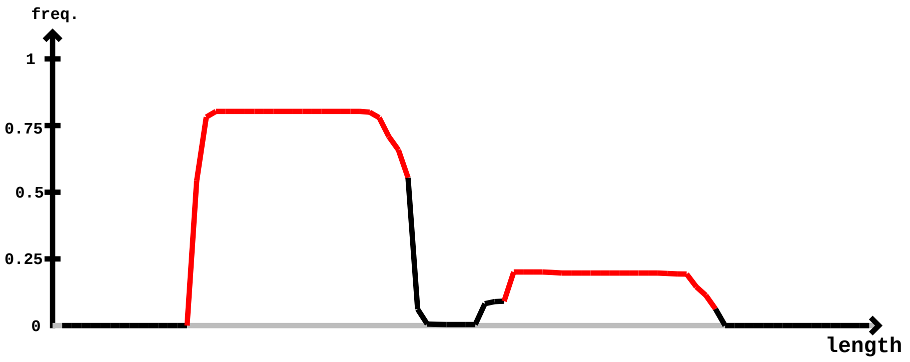

novel-nve-miR-6_guide

novel-nve-miR-6_star

```
5'- cggucuccucggaauucuguacagucuccuguugucuuuuaagacaagagcagucuguauagaauuccgagagcagacucuuca   -3'    exp
    .((((((((((((((((((((((.((.((.((((((....)))))).)).)).))))))))))))))))).).))))).....   reads      mm         sample
    .............auucuguacagucuccug.....................................................   3          0          seq
    .............auucuguacagucuccuA.....................................................   1          1          seq
    .............auucuguacGgucuccug.....................................................   1          1          seq
    .............auucuguacagucuccugu....................................................   29         0          seq
    .............auucuguacagucuccugC....................................................   1          1          seq
    .............Uuucuguacagucuccugu....................................................   1          1          seq
    .............auucuguaUagucuccugu....................................................   2          1          seq
    .............auucuguGcagucuccugu....................................................   1          1          seq
    .............Guucuguacagucuccuguu...................................................   1          1          seq
    .............auucuguacagucuccuguC...................................................   11         1          seq
    .............auucuguacagucuccuguG...................................................   2          1          seq
    .............auucuguacagucuccuguu...................................................   93         0          seq
    .............auucCguacagucuccuguu...................................................   2          1          seq
    .............auucugAacagucuccuguu...................................................   1          1          seq
    .............auucuguGcagucuccuguu...................................................   1          1          seq
    .............auucuguacagucuccugCu...................................................   1          1          seq
    .............auucuguacagucuccuguA...................................................   3          1          seq
    .............auucuguacUgucuccuguug..................................................   1          1          seq
    .............auucCguacagucuccuguug..................................................   1          1          seq
    .............auucuguacagucuccuguuA..................................................   4          1          seq
    .............auucuguacagucuccuguuC..................................................   4          1          seq
    .............auucuguacagucuccuguug..................................................   51         0          seq
    .............auucuguacagucuccuCuug..................................................   1          1          seq
    .............auucuguacagucuccCguug..................................................   1          1          seq
    .............auucuguacagucuccuguAg..................................................   1          1          seq
    .............auucuguacagucuccuguuU..................................................   10         1          seq
    .............Cuucuguacagucuccuguug..................................................   1          1          seq
    .............auucuguacagucuccuguugC.................................................   18         1          seq
    .............auucuguacagucuccuguugu.................................................   102        0          seq
    .............auucuguacagucucUuguugu.................................................   2          1          seq
    .............auucuguacagucuccuguugA.................................................   3          1          seq
    .............auucuguGcagucuccuguugu.................................................   1          1          seq
    .............auucuguacagucuccuguuUu.................................................   4          1          seq
    .............auucAguacagucuccuguugu.................................................   1          1          seq
```

```
             novel-nve-miR-6_guide
                                           novel-nve-miR-6_star
cggucuccucggaauucuguacagucuccuguugucuuuuaagacaagagcagucuguauagaauuccgagagcagacucuuca

.............auucuguacagucuccuguugG.................................................   4     1     seq
.............auucuguacagucuccuguCgu.................................................   1     1     seq
.............auucuguacGgucuccuguugu.................................................   1     1     seq
.............auucuguacUgucuccuguuguc................................................   1     1     seq
.............aAucuguacagucuccuguuguc................................................   1     1     seq
.............auucuguacagucuccAguuguc................................................   1     1     seq
.............auucuguacagAcuccuguuguc................................................   1     1     seq
.............auucuguacagucuccuguugCc................................................   2     1     seq
.............auuGuguacagucuccuguuguc................................................   1     1     seq
.............auucuguGcagucuccuguuguc................................................   3     1     seq
.............auucugCacagucuccuguuguc................................................   1     1     seq
.............auucuguUcagucuccuguuguc................................................   1     1     seq
.............auucuguaUagucuccuguuguc................................................   2     1     seq
.............auucuguacagucuccuguuUuc................................................   1     1     seq
.............auucuguacagucuccugCuguc................................................   1     1     seq
.............auucuguacagucCccuguuguc................................................   2     1     seq
.............Cuucuguacagucuccuguuguc................................................   1     1     seq
.............auucuguacGgucuccuguuguc................................................   7     1     seq
.............auucuguacagucuccuguuguU................................................   55    1     seq
.............auucuguacagucuccuguCguc................................................   1     1     seq
.............auucuguacagucuAcuguuguc................................................   1     1     seq
.............Guucuguacagucuccuguuguc................................................   6     1     seq
.............auucuguacaguUuccuguuguc................................................   1     1     seq
.............auucuguacagCcuccuguuguc................................................   3     1     seq
.............auucuguacagucuccuguuguc................................................   451   0     seq
.............auucuguacagucuccuguuguA................................................   21    1     seq
.............auCcuguacagucuccuguuguc................................................   2     1     seq
.............auucuguacagucuccuguAguc................................................   2     1     seq
.............Uuucuguacagucuccuguuguc................................................   1     1     seq
.............auucuguacagucuccuCuuguc................................................   2     1     seq
.............auucuguacagucuUcuguuguc................................................   1     1     seq
.............auuAuguacagucuccuguuguc................................................   1     1     seq
.............auucuguacagucucUuguuguc................................................   3     1     seq
.............auucuguacagucuccGguuguc................................................   1     1     seq
.............auAcuguacagucuccuguuguc................................................   1     1     seq
.............auucuguacagucuccuguuguG................................................   1     1     seq
.............auucuguacagucucGuguugucu...............................................   1     1     seq
.............auucuguacagucuccuguugCcu...............................................   1     1     seq
.............auucuguacagucuccuguugucu...............................................   12    0     seq
.............auucuguacagucuccuguugucC...............................................   4     1     seq
.............auucuguacagucuccuguugucG...............................................   1     1     seq
.............auucuguacagucuccuguugucA...............................................   1     1     seq
.............auuUuguacagucuccuguugucu...............................................   2     1     seq
.............auucuguacagucuccuguugucuu..............................................   2     0     seq
.............auucuAuacagucuccuguugucuuu.............................................   1     1     seq
.............auucuguacagucuccuguugucuuuuaagacaagagc.................................   1     0     seq
..............uucuguacagucuccugu....................................................   3     0     seq
..............uucuguacagucuccuguu...................................................   11    0     seq
..............uucuguacagucuccuguA...................................................   1     1     seq
..............uucuguacagucuccuguuA..................................................   1     1     seq
..............uucuguacagucuccuguug..................................................   9     0     seq
..............uucuguacagucuccuguuU..................................................   5     1     seq
..............uucuguacagCcuccuguugu.................................................   1     1     seq
..............uucuguaAagucuccuguugu.................................................   1     1     seq
..............Gucuguacagucuccuguugu.................................................   1     1     seq
..............uucuguacagucuccugCugu.................................................   2     1     seq
..............uucuguacagucuccuguugC.................................................   5     1     seq
..............uucuguacagucuccuguCgu.................................................   2     1     seq
..............uucuguacagucuccuguugu.................................................   37    0     seq
..............uucuguacagucuccuguuUuc................................................   1     1     seq
..............uucCguacagucuccuguuguc................................................   2     1     seq
..............uucuguacagucuccuguuguc................................................   235   0     seq
..............Gucuguacagucuccuguuguc................................................   1     1     seq
..............Aucuguacagucuccuguuguc................................................   2     1     seq
..............uucuguacagucCccuguuguc................................................   1     1     seq
..............uucuguacagucuccuguuguA................................................   7     1     seq
..............uucuguacagucucUuguuguc................................................   5     1     seq
..............uucuguacGgucuccuguuguc................................................   2     1     seq
..............uucugCacagucuccuguuguc................................................   1     1     seq
```

```
             novel-nve-miR-6_guide
                                            novel-nve-miR-6_star
cggucuccucggaauucuguacagucuccuguugucuuuuaagacaagagcagucuguauagaauuccgagagcagacucuuca

..............uucuCuacagucuccuguuguc................................................    1    1    seq
..............uucuguacagucuccuguugAc................................................    1    1    seq
..............uucuguacagucuccuguuguU................................................    28   1    seq
..............uucuguacagucuccugCuguc................................................    1    1    seq
..............uAcuguacagucuccuguuguc................................................    1    1    seq
..............uucuguacagucuccAguuguc................................................    1    1    seq
..............uucuguacagucAccuguuguc................................................    1    1    seq
..............uCcuguacagucuccuguuguc................................................    1    1    seq
..............uucuguacagucuAcuguuguc................................................    2    1    seq
..............Cucuguacagucuccuguuguc................................................    1    1    seq
..............uucuguacagCcuccuguugucu...............................................    1    1    seq
..............uucuguacagucuccuguugucA...............................................    5    1    seq
..............uucuguacagucuccuguuguGu...............................................    1    1    seq
..............uucuguacagucuccuguugucC...............................................    7    1    seq
..............Aucuguacagucuccuguugucu...............................................    1    1    seq
..............uucuguacagucuccuguugucu...............................................    33   0    seq
..............uucuguacGgucuccuguugucu...............................................    1    1    seq
..............uucuguacagucuccuguugucuuuuaagacaagag.................................    2    0    seq
..............uucuguacagucuccCguugucuuuuaagacaagag.................................    1    1    seq
..............uucuguacagucuccuguugucuuuuaagacaagagc................................    2    0    seq
..............uucuguacagucuccuguugucuuuuaagacaagagA................................    1    1    seq
...............ucuguacagucuccuguugC.................................................    1    1    seq
...............ucuguacagucuccuguuguU................................................    2    1    seq
...............ucuguacagucuccuguugGc................................................    1    1    seq
...............ucuguaUagucuccuguuguc................................................    2    1    seq
...............ucuguacagucuccuguuguc................................................    3    0    seq
...............ucuguacagucuccuguugucA...............................................    4    1    seq
...............ucuguacagucuccuguugucu...............................................    19   0    seq
...............ucugCacagucuccuguugucu...............................................    1    1    seq
...............ucuguacagucuccCguugucu...............................................    1    1    seq
...............ucuguacagucuccuguugucC...............................................    1    1    seq
...............ucuguacagucuccuguugucG...............................................    1    1    seq
...............ucuguacGgucuccuguugucu...............................................    1    1    seq
...............ucGguacagucuccuguugucu...............................................    1    1    seq
...........................................acaagagcagucuguauaA......................    1    1    seq
...........................................acaagagcagucuguauag......................    2    0    seq
...........................................acaagagcagucuguauaga.....................    3    0    seq
...........................................acaagagcagucuguauagaa....................    1    0    seq
...........................................Ucaagagcagucuguauagaau...................    1    1    seq
...........................................acGagagcagucuguauagaau...................    1    1    seq
...........................................acaagagcagucuguauagaGu...................    1    1    seq
...........................................acaaUagcagucuguauagaau...................    1    1    seq
...........................................acaagagcagucuguauagaaC...................    15   1    seq
...........................................acaagagcagCcuguauagaau...................    1    1    seq
...........................................acaagagUagucuguauagaau...................    1    1    seq
...........................................acaagagcagucuguauagaau...................    61   0    seq
...........................................acaagagcagucuguauagaaCu..................    5    1    seq
...........................................acaagagcaguAuguauagaauu..................    1    1    seq
...........................................acaagagcagucuguauagaauA..................    2    1    seq
...........................................acaagagcagucuguauagaauu..................    37   0    seq
...........................................Gcaagagcagucuguauagaauu..................    1    1    seq
...........................................acaagagcagucuguauagaaAu..................    1    1    seq
...........................................acaagagcagucuguauagaauC..................    3    1    seq
...........................................acaagagcagucuguauagaauuU.................    1    1    seq
............................................caagagcagucuguauagaau...................    2    0    seq
............................................caagagcagucuguauagaauC..................    1    1    seq
............................................caagagcagucuguauagaauuU.................    2    1    seq
............................................caagagcagucuguauagaauuc.................    9    0    seq
.............................................aagagcagucuguauagaauuU.................    1    1    seq
.............................................aagagcagucuguauagaauuc.................    2    0    seq
..............................................agagcagucuguauagaau...................    1    0    seq
..............................................agagcagucuguauagaauC..................    1    1    seq
..............................................agagcagucuguauagaauu..................    6    0    seq
..............................................agagcagucuguauGgaauuc.................    1    1    seq
..............................................agagcagucuguauagaGuuc.................    1    1    seq
..............................................agagcagucuguUuagaauuc.................    1    1    seq
..............................................agagcagucuguauagGauuc.................    1    1    seq
..............................................agagcagucuguaCagaauuc.................    1    1    seq
..............................................agGgcagucuguauagaauuc.................    1    1    seq
```

novel-nve-miR-6_guide

novel-nve-miR-6_star

cggucuccucggaauucuguacagucuccuguugucuuuuaagacaagagcagucuguauagaauuccgagagcagacucuuca

....agagcagucuguauagaauuc.................	70	0	seq
....agagcagucuguauagaauucU................	95	1	seq
....agagcagucuguauagaauucA................	6	1	seq
....agagcagucuguauagaauuAc................	1	1	seq
....agagcagucuguauagaauucc................	10	0	seq

miRBase precursor : novel-nve-miR-8
Total read count : 34694
novel-nve-miR-8_guide read count : 34269
novel-nve-miR-8_star read count : 425
remaining reads : 0

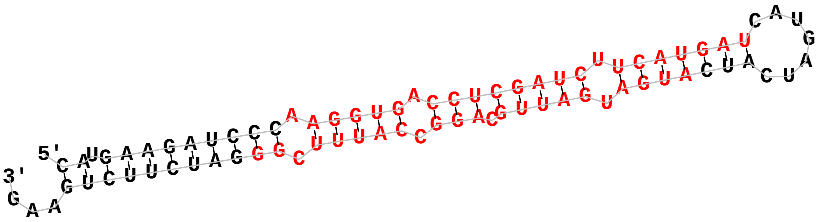

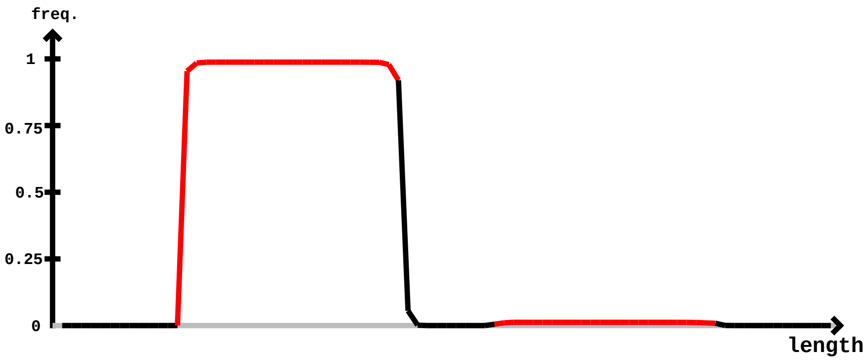


```
                                                        novel-nve-miR-8_star
              novel-nve-miR-8_guide
5'- caugaagaucccaaggugaccucgaucuucaugaucaugaucaucaugaugauugcaggccauuucgggaucuucugaag  -3'   exp
    ((.((((((((.(((((.((((((((.(((((((.......))))))).))))).))).))))).))))))))))...   reads    mm     sample
    ...........caaggugaccucgaucuucaugau.............................................   5        0      seq
    ...........caaggugaccucgaucuCcaugau.............................................   1        1      seq
    ............aaggugaccucgaucuuc..................................................   1        0      seq
    ............aaggugaccucgaucuuca.................................................   13       0      seq
    ............aaggugaccucgGucuuca.................................................   1        1      seq
    ............aaggugaccucgaucuucau................................................   6        0      seq
    ............Gaggugaccucgaucuucau................................................   1        1      seq
    ............aaggugaccucgaucuucGug...............................................   2        1      seq
    ............aaggugaccucgaucuucaCg...............................................   2        1      seq
    ............aaggugaccucgaucCucaug...............................................   1        1      seq
    ............aaggAgaccucgaucuucaug...............................................   3        1      seq
    ............aaggugaccuUgaucuucaug...............................................   2        1      seq
    ............aaggugaccucgaucAucaug...............................................   1        1      seq
    ............aaggugaccucgaucuucauA...............................................   23       1      seq
    ............aaggugaccCcgaucuucaug...............................................   1        1      seq
    ............aaggugaccucgauAuucaug...............................................   1        1      seq
    ............aagguCaccucgaucuucaug...............................................   1        1      seq
    ............aaggugacAucgaucuucaug...............................................   1        1      seq
    ............aaggugaUcucgaucuucaug...............................................   1        1      seq
    ............aaggugaccucgaucuCcaug...............................................   2        1      seq
    ............aaggCgaccucgaucuucaug...............................................   1        1      seq
    ............aaggugaccucgaGcuucaug...............................................   1        1      seq
    ............aaggugaccucgaucuucauU...............................................   6        1      seq
    ............aaggugaccucgaucuucaug...............................................   232      0      seq
    ............aaggugaccucgaucuuUaug...............................................   1        1      seq
    ............aaggugaccucgaucuucaGg...............................................   1        1      seq
    ............Gaggugaccucgaucuucaug...............................................   1        1      seq
    ............aaggugaccucgaucuucaugC..............................................   2        1      seq
    ............aaggugaccucgaucuucaAga..............................................   1        1      seq
    ............aaggugaccucAaucuucauga..............................................   1        1      seq
    ............aagguCaccucgaucuucauga..............................................   2        1      seq
    ............aaggugaccucgaucuuUauga..............................................   6        1      seq
    ............aagAugaccucgaucuucauga..............................................   1        1      seq
    ............aagguAaccucgaucuucauga..............................................   4        1      seq
```

```
                                             novel-nve-miR-8_star
            novel-nve-miR-8_guide
caugaagaucccaaggugaccucgaucuucaugaucaugaucaucaugaugauugcaggccauuucgggaucuucugaag

............aaggugaccucgaucuucaGga..............................................    1       1       seq
............aaggugaccucgaucuucaugU..............................................    23      1       seq
............Gaggugaccucgaucuucauga..............................................    16      1       seq
............aUggugaccucgaucuucauga..............................................    1       1       seq
............aaggugacGucgaucuucauga..............................................    2       1       seq
............aaggugGccucgaucuucauga..............................................    16      1       seq
............aaggugaccucgaucuGcauga..............................................    1       1       seq
............aaggugaccucgaucGucauga..............................................    1       1       seq
............aaggugaccucgaucCucauga..............................................    7       1       seq
............aaggGgaccucgaucuucauga..............................................    2       1       seq
............aaggugUccucgaucuucauga..............................................    2       1       seq
............aaggugaccucgaucuucGuga..............................................    4       1       seq
............aGggugaccucgaucuucauga..............................................    8       1       seq
............aaggugaccucgauUuucauga..............................................    3       1       seq
............aaggugaccucgaucuucaugG..............................................    21      1       seq
............aaggugaccucgaGcuucauga..............................................    1       1       seq
............aaggugaccuUgaucuucauga..............................................    2       1       seq
............aaggCgaccucgaucuucauga..............................................    5       1       seq
............Caggugaccucgaucuucauga..............................................    1       1       seq
............aaggugaccucgauAuucauga..............................................    1       1       seq
............aaggugaccAcgaucuucauga..............................................    1       1       seq
............aaAgugaccucgaucuucauga..............................................    3       1       seq
............aaggugaccucgaucuucUuga..............................................    2       1       seq
............aaggugaccucgaucuucCuga..............................................    1       1       seq
............aaggugaUcucgaucuucauga..............................................    2       1       seq
............aaggugaccucUaucuucauga..............................................    1       1       seq
............Uaggugaccucgaucuucauga..............................................    1       1       seq
............aaggugaccucgaucuCcauga..............................................    10      1       seq
............aaggugaccucgGucuucauga..............................................    12      1       seq
............aagCugaccucgaucuucauga..............................................    2       1       seq
............aaggugaccucgaAcuucauga..............................................    1       1       seq
............aaggugaccCcgaucuucauga..............................................    7       1       seq
............aaggugacUucgaucuucauga..............................................    1       1       seq
............aaggugaccucgaucuucauAa..............................................    3       1       seq
............aaggugaccucgaucuuGauga..............................................    2       1       seq
............aaggugaccucgaCcuucauga..............................................    3       1       seq
............aaggugacAucgaucuucauga..............................................    4       1       seq
............aaggugaccuAgaucuucauga..............................................    2       1       seq
............aaggugaccucgaucuucauga..............................................    1739    0       seq
............aaggugaccucgaucuucaCga..............................................    3       1       seq
............aaggAgaccucgaucuucauga..............................................    5       1       seq
............aaAgugaccucgaucuucaugau.............................................    31      1       seq
............aGggugaccucgaucuucaugau.............................................    89      1       seq
............aaggugaccucgaCcuucaugau.............................................    72      1       seq
............aaggugaccucgaucuAcaugau.............................................    21      1       seq
............aaUgugaccucgaucuucaugau.............................................    11      1       seq
............aaggugaccucgaucuuGaugau.............................................    1       1       seq
............aagguUaccucgaucuucaugau.............................................    10      1       seq
............aagguAaccucgaucuucaugau.............................................    25      1       seq
............aaggugaccucgaucuGcaugau.............................................    7       1       seq
............aaggugaccucgaucuucaGgau.............................................    6       1       seq
............aaggugaccucgaucuucaugCu.............................................    5       1       seq
............aaggugaccucgaucuCcaugau.............................................    91      1       seq
............aaggugaccucgaucuucaCgau.............................................    54      1       seq
............aaggugaccucgaucuucaugau.............................................    23287   0       seq
............aaggugUccucgaucuucaugau.............................................    28      1       seq
............aaggugaccucgaucuuUaugau.............................................    36      1       seq
............aaggugaAcucgaucuucaugau.............................................    10      1       seq
............aaggugaccuGgaucuucaugau.............................................    2       1       seq
............aaggugaccucgaucuuAaugau.............................................    8       1       seq
............aaggugaccucgauUuucaugau.............................................    15      1       seq
............aaggugaccucUaucuucaugau.............................................    6       1       seq
............aUggugaccucgaucuucaugau.............................................    24      1       seq
............aaggugaUcucgaucuucaugau.............................................    28      1       seq
............Uaggugaccucgaucuucaugau.............................................    34      1       seq
............aaggugaccucgaucuucaAgau.............................................    34      1       seq
............aaggugaccucgaAcuucaugau.............................................    20      1       seq
............aaggGgaccucgaucuucaugau.............................................    19      1       seq
............aaggugaccucgaucAucaugau.............................................    19      1       seq
```

novel-nve-miR-8_star

novel-nve-miR-8_guide

caugaagauccc aaggugaccucgaucuucaugau caugaucauc augaugauugcaggccauuucgg gaucuucugaag

............aaggugaccucgaucCucaugau...	110	1	seq
............aaggugaccucgaucGucaugau...	7	1	seq
............aaggugaccCgaucuucaugau...	102	1	seq
............aaggugaccucgaucuucaugaA...	430	1	seq
............aaggugaccucgaGcuucaugau...	4	1	seq
............aaggugaccucgaucuucauCau...	6	1	seq
............Caggugaccucgaucuucaugau...	4	1	seq
............aaggugCccucgaucuucaugau...	29	1	seq
............aaggugaccucgGucuucaugau...	95	1	seq
............aaggugaccuAgaucuucaugau...	20	1	seq
............aaggugacUucgaucuucaugau...	65	1	seq
............Gaggugaccucgaucuucaugau...	199	1	seq
............aaggugaccucgCucuucaugau...	5	1	seq
............aaggugaccucgaucuucCugau...	6	1	seq
............aagAugaccucgaucuucaugau...	21	1	seq
............aCggugaccucgaucuucaugau...	6	1	seq
............aaCgugaccucgaucuucaugau...	11	1	seq
............aaggugaccucgauGucaugau...	1	1	seq
............aaggugacAucgaucuucaugau...	44	1	seq
............aagCugaccucgaucuucaugau...	20	1	seq
............aagUugaccucgaucuucaugau...	11	1	seq
............aaggugaccucgaucuucaugUu...	13	1	seq
............aaggugaccucCaucuucaugau...	5	1	seq
............aaggugaccucgaucuucauAau...	23	1	seq
............aaggAgaccucgaucuucaugau...	54	1	seq
............aaggugaccucAaucuucaugau...	26	1	seq
............aaggugaccucgUucuucaugau...	8	1	seq
............aagguCaccucgaucuucaugau...	12	1	seq
............aaggugaccucgaucuucauUau...	4	1	seq
............aaggugaccGcgaucuucaugau...	8	1	seq
............aaggugaccucgaucuucUugau...	16	1	seq
............aaggugGccucgaucuucaugau...	124	1	seq
............aaggugaccAcgaucuucaugau...	33	1	seq
............aaggugaccucgaucuucaugaG...	210	1	seq
............aaggugaccucgaucuucGugau...	105	1	seq
............aaggugaccucgaucuucaugaC...	2960	1	seq
............aaggugaccuUgaucuucaugau...	44	1	seq
............aaggugaccucgaucuucaugGu...	134	1	seq
............aaggugaccucgauAuucaugau...	18	1	seq
............aaggugacGucgaucuucaugau...	4	1	seq
............aaggCgaccucgaucuucaugau...	75	1	seq
............aaggugaccucgaucuucaugauU..	1504	1	seq
............aaggugaccucgaucuucaCgauc..	1	1	seq
............aaggugaccucgaCcuucaugauc..	1	1	seq
............aaggugacUucgaucuucaugauc..	1	1	seq
............aaggugGccucgaucuucaugauc..	1	1	seq
............aaggugaccucgaucuucaugauA..	40	1	seq
............aaggugCccucgaucuucaugauc..	1	1	seq
............Uaggugaccucgaucuucaugauc..	1	1	seq
............aaggugaccucgaucuucaAgauc..	2	1	seq
............aaggugaccucgaucuucaugauc..	172	0	seq
............aaggugaccucgaucuuUaugauc..	1	1	seq
............aaggugaccucgGucuucaugauc..	1	1	seq
............aaggugaccucgaucuucaugauG..	7	1	seq
............aaggugaccucgaucuucaugGuc..	1	1	seq
............aagAugaccucgaucuucaugauc..	1	1	seq
............aaggugaccucgaucuucGugauc..	2	1	seq
............aaggugaccucgaucuucauCauc..	1	1	seq
............aaggugaccucgaucuucaugauAa...	1	1	seq
............aaggugaccucgaucuucaugauUa...	42	1	seq
............aaggugaccucgaucuucaugauca...	2	0	seq
............aaggugaccucgaucuucaugaucU...	2	1	seq
............aaggugaccucgaucuucaugauUau..	1	1	seq
.............Gggugaccucgaucuucaug...	1	1	seq
.............aggugaccucgGucuucaug...	1	1	seq
.............aggugaccucgaucuucaug...	6	0	seq
.............aggugaccucgaucuucaugG..	1	1	seq
.............aAgugaccucgaucuucauga..	2	1	seq
.............Gggugaccucgaucuucauga..	3	1	seq

```
                                            novel-nve-miR-8_star
                novel-nve-miR-8_guide
caugaagauccc aaggugaccucgaucuucaugau caugaucauc augaugauugcaggccauuucgg gaucuucugaag
```

```
caugaagaucccaaggugaccucgaucuucaugaucaugaucaucaugaugauugcaggccauuucgggaucuucugaag

.............aggugaccucgaucuucauga..............................................    89     0     seq
.............aggugaccucgaucuCcauga..............................................    1      1     seq
.............Uggugaccucgaucuucauga..............................................    1      1     seq
.............aggugaccucgaucuucaugU..............................................    2      1     seq
.............aggugaccucgaucuucaAgau.............................................    1      1     seq
.............aggugaccucgaucuCcaugau.............................................    2      1     seq
.............aggugaccuUgaucuucaugau.............................................    3      1     seq
.............aggugaccucgaucuucaugau.............................................    663    0     seq
.............aggCgaccucgaucuucaugau.............................................    1      1     seq
.............aAgugaccucgaucuucaugau.............................................    11     1     seq
.............aggugaccucgaucGucaugau.............................................    1      1     seq
.............agCugaccucgaucuucaugau.............................................    2      1     seq
.............agguCaccucgaucuucaugau.............................................    1      1     seq
.............aggugaccuGgaucuucaugau.............................................    1      1     seq
.............aggugaccucgaucuucGugau.............................................    3      1     seq
.............aggugaUcucgaucuucaugau.............................................    1      1     seq
.............aggugaccucgaucuucaugaA.............................................    10     1     seq
.............aggugaccucgaucuucaCgau.............................................    3      1     seq
.............aggugaccucgGucuucaugau.............................................    3      1     seq
.............aggugCccucgaucuucaugau.............................................    1      1     seq
.............aggugGccucgaucuucaugau.............................................    2      1     seq
.............Gggugaccucgaucuucaugau.............................................    5      1     seq
.............aggugaccucgaucuGcaugau.............................................    1      1     seq
.............aggugaccucgaucuucaugaC.............................................    71     1     seq
.............aggugacUucgaucuucaugau.............................................    2      1     seq
.............Uggugaccucgaucuucaugau.............................................    3      1     seq
.............aggugaccucgaCcuucaugau.............................................    1      1     seq
.............aggugaccucgaucuucaugaG.............................................    7      1     seq
.............aggugaccucgaucCucaugau.............................................    1      1     seq
.............aggugacAucgaucuucaugau.............................................    2      1     seq
.............aggAgaccucgaucuucaugau.............................................    4      1     seq
.............aggugaccucgaucuucaugUu.............................................    1      1     seq
.............agguAaccucgaucuucaugau.............................................    2      1     seq
.............aggugaccAcgaucuucaugau.............................................    1      1     seq
.............aggugaccucgaucuucaugGu.............................................    7      1     seq
.............aggugaccucgaucuucauAau.............................................    1      1     seq
.............aggugaccCcgaucuucaugau.............................................    3      1     seq
.............aggugaccucgaucuucaugauc............................................    8      0     seq
.............aggugaccucgaucuucaugauA............................................    5      1     seq
.............aggugaccucgaucCucaugauc............................................    1      1     seq
.............aggugaccucgaucuucaugauU............................................    107    1     seq
.............aggugaccucgaucuucaugauUa...........................................    2      1     seq
.............aggugaccucgaucuucaugaucU...........................................    3      1     seq
..............ggugaccucgaucuucaug...............................................    2      0     seq
..............ggugaccucgaucuucauga..............................................    3      0     seq
..............ggugaccucgaucuucaugaC.............................................    9      1     seq
..............gguCaccucgaucuucaugau.............................................    1      1     seq
..............ggCgaccucgaucuucaugau.............................................    1      1     seq
..............ggugaccucgauUuucaugau.............................................    1      1     seq
..............ggugaccucgaucuucaugaA.............................................    1      1     seq
..............ggugacUucgaucuucaugau.............................................    1      1     seq
..............ggugaccucgaucuucaugau.............................................    76     0     seq
..............ggugaccucgaCcuucaugau.............................................    1      1     seq
..............ggugaccucgaucuucaugauU............................................    10     1     seq
..............ggugaccucgaucuucaugauc............................................    1      0     seq
...............gugaccucgaucuucaugau.............................................    5      0     seq
............................................caugaugauugcaggcca..................    1      0     seq
............................................caugaugauugcaggccaC.................    1      1     seq
............................................caugaugGuugcaggccau.................    1      1     seq
............................................caugaugauugcaggccau.................    3      0     seq
............................................caugaugauugUaggccauu................    2      1     seq
............................................caugaugauugcaggccauu................    1      0     seq
............................................caugaugauugcaggccauuC...............    1      1     seq
............................................Gaugaugauugcaggccauuu...............    1      1     seq
............................................caugaugauugcaggccauuu...............    12     0     seq
............................................caugaugauugcaggccauuuA..............    1      1     seq
............................................caugaugauugcaggccauuuU..............    1      1     seq
............................................caugaAugcaggccauuuc.................    1      1     seq
............................................caugaugauugcaggccauuuc..............    23     0     seq
```

```
                                          novel-nve-miR-8_star
           novel-nve-miR-8_guide
caugaagauccc aaggugaccucgaucuucaugau caugaucauc augaugauugcaggccauuucgg gaucuucugaag
```

```
caugaagatcccaaggugaccucgaucuucaugaucaugaucaucaugaugauugcaggccauuucgggaucuucugaag
```

```
............................................caugaugauugcaggccGuuuc..............    1     1    seq
............................................caugaugauugcaggccauuucg.............    16    0    seq
............................................caugaugauugcaggccauuucA.............    2     1    seq
............................................caugaCgauugcaggccauuucg.............    1     1    seq
............................................caugaugauugcaggccauuucU.............    4     1    seq
............................................caugaugauugcaggccauuucgg............    82    0    seq
............................................caugGugauugcaggccauuucgg............    2     1    seq
............................................caugaugauugcaggccauuucgA............    7     1    seq
............................................caGgaugauugcaggccauuucgg............    1     1    seq
............................................caugaugauugcGggccauuucgg............    1     1    seq
............................................caugaugauugcaggccaAuucgg............    1     1    seq
............................................caugaAgauugcaggccauuucgg............    1     1    seq
............................................caugaugauugUaggccauuucgg............    1     1    seq
............................................caugaugauugcaggccauuucAg............    1     1    seq
............................................caugaugauugcaggccauuucgU............    1     1    seq
............................................caugaugauugcaggccauuucggU...........    4     1    seq
............................................caugaugauugcaggccauuucggA...........    4     1    seq
............................................caugaugauugcaggccauuucggUa..........    1     1    seq
.............................................augaugauugcaggccauA................    1     1    seq
.............................................augaugauugcaggccauuuA..............    1     1    seq
.............................................augaugauugcaggccauuuc..............    18    0    seq
.............................................augaugauugcaggccauuuU..............    1     1    seq
.............................................augaugauugcUggccauuuc..............    1     1    seq
.............................................augGugauugcaggccauuucg.............    2     1    seq
.............................................augaugauugcaggccauuucA.............    1     1    seq
.............................................augaugauugcaggccauuucg.............    11    0    seq
.............................................aAgaugauugcaggccauuucg.............    1     1    seq
.............................................augaugauugcaggccauuucgA............    9     1    seq
.............................................Gugaugauugcaggccauuucgg............    3     1    seq
.............................................augaugauCgcaggccauuucgg............    1     1    seq
.............................................augaCgauugcaggccauuucgg............    1     1    seq
.............................................aCgaugauugcaggccauuucgg............    1     1    seq
.............................................augaugauugcaggccGuuucgg............    3     1    seq
.............................................augaugauugcaggccauuucgU............    1     1    seq
.............................................augaAgauugcaggccauuucgg............    3     1    seq
.............................................augaugauugcaggccauuucgg............    110   0    seq
.............................................augaugauugcaUgccauuucgg............    1     1    seq
.............................................augaugauugcaggccauuucggA...........    3     1    seq
.............................................augaugauugcaggccauuucggUau.........    1     1    seq
..............................................ugaugauugcaggccauuu...............    2     0    seq
..............................................ugaugauugcaggccauuuc..............    5     0    seq
..............................................ugaugauugcCggccauuucg.............    1     1    seq
..............................................ugaugauugcagAccauuucg.............    1     1    seq
..............................................ugaugauugcaggccauuucA.............    1     1    seq
..............................................ugaugauCgcaggccauuucg.............    1     1    seq
..............................................ugaugauugcaggccauuucg.............    5     0    seq
..............................................ugaugauugcaggccauuucAg............    1     1    seq
..............................................ugaugauugcaggccauuucgC............    1     1    seq
..............................................ugaugauugcaggccauuucgg............    40    0    seq
..............................................Agaugauugcaggccauuucgg............    1     1    seq
..............................................Cgaugauugcaggccauuucgg............    1     1    seq
..............................................ugaugauugcaggccauuucgU............    2     1    seq
..............................................ugaugauugcaggccauuucgA............    6     1    seq
..............................................ugaugauugcaggccauuucggU...........    3     1    seq
```

miRBase precursor : novel-nve-miR-9
Total read count : 162
novel-nve-miR-9_guide read count : 161
novel-nve-miR-9_star read count : 1
remaining reads : 0

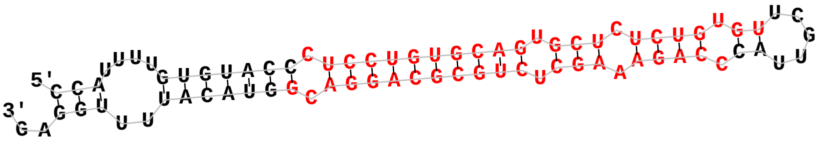

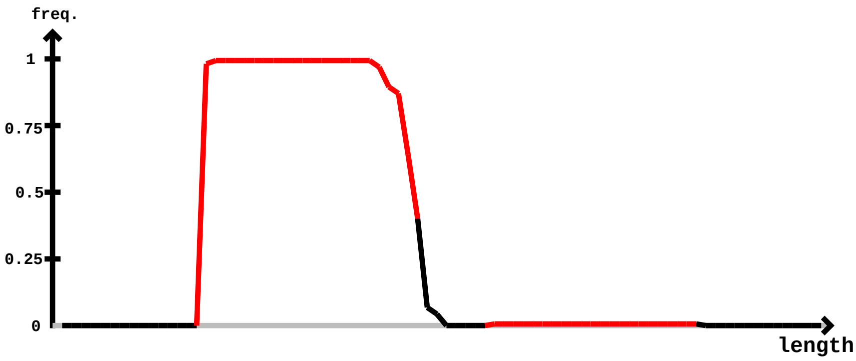


```
                              novel-nve-miR-9_guide
                                                            novel-nve-miR-9_star
5'- ccauuuuguguacccuccugugcagugcucucuguguucguuacccagaaagcucugcgcaggacgguacauuuuggag   -3'    exp
    (((....(((((((.((((((((((.(((.((((.((.....)).)))).))).)))))))))).)))))))..)))..    reads      mm         sample
    .............cuccugugcagugcucuc............................................    4          0          seq
    .............cuccugugcagugcucucC...........................................    2          1          seq
    .............cuccugugcagugcucucu...........................................    9          0          seq
    .............cCccugugcagugcucucu...........................................    1          1          seq
    .............cuccugugcagugcucucug..........................................    4          0          seq
    .............cuccugugcagCgcucucugu.........................................    1          1          seq
    .............cuccugugcagugcucucugC.........................................    1          1          seq
    .............cuccugugcagugcucucugu.........................................    35         0          seq
    .............cuccugugcagugcucucugug........................................    37         0          seq
    .............cuccugugcagugcucucugugC.......................................    4          1          seq
    .............cuccugugcagCgcucucugugu.......................................    1          1          seq
    .............cuccugugcagugcucucugugA.......................................    1          1          seq
    .............cuccugugcagugcucucuguUu.......................................    1          1          seq
    .............cuccugugcagugcAcucugugu.......................................    1          1          seq
    .............cuccugugcagugcucucugugu.......................................    44         0          seq
    .............cuccugugcagugcucucugugG.......................................    1          1          seq
    .............cuccugugcGgugcucucugugu.......................................    1          1          seq
    .............cuccugugcagugcucucuguguu......................................    2          0          seq
    .............cuccugugcagugcucucuguguC......................................    2          1          seq
    .............cuccugugcagugcucucuguguuc.....................................    1          0          seq
    .............cuccugugcagugcucucuguguuU.....................................    6          1          seq
    ..............uccugugcagugcucucuguA........................................    1          1          seq
    ..............uccugugcagugcucucugug........................................    1          0          seq
    ........................................ccagaaagcucugcgcaggacg............    1          0          seq
```

miRBase precursor : novel-nve-miR-10
Total read count : 586
novel-nve-miR-10_guide read count : 565
novel-nve-miR-10_star read count : 11
remaining reads : 10

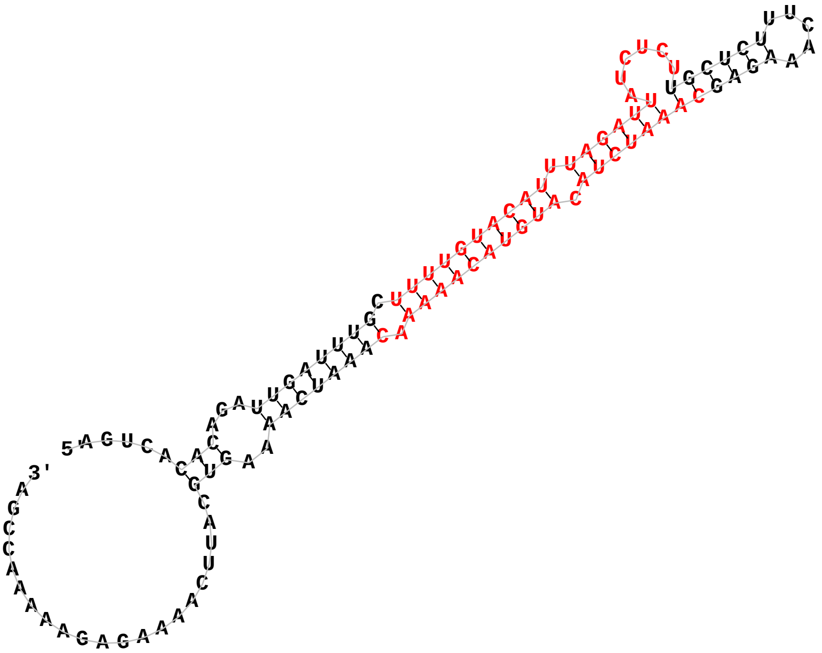

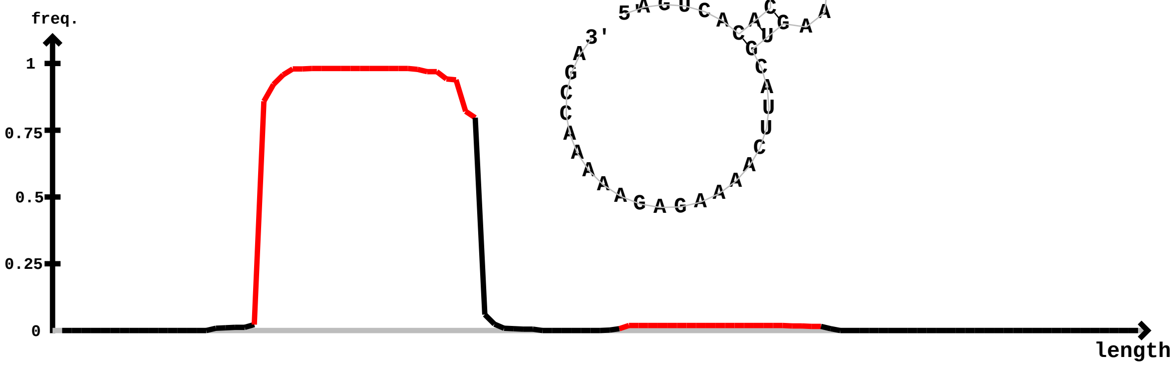


```
                                   novel-nve-miR-10_guide
                                                                               novel-nve-miR-10_star
5'- agucacacagauugauuugcuuuuguacauuuagauuaucucuugcucuuucaaagagcaaaucuacauguacaaaaacaaaucaaaagugcauucaaaagagaaaaaccga  -3'   exp
    .....(((...((((((((.((((((((((.((((((......((((((.....)))))))))))).)))))))))).))))))))..))).....................  reads   mm   sample
    ...............uuugcuuuuguacauGuagau............................................................................  1       1    seq
    ...............uuugcuuuuguacauGuagauu...........................................................................  4       1    seq
    ................uugcuuuuguacauGuagauu...........................................................................  1       1    seq
    .................ugcuuuuguacauGuagau............................................................................  1       1    seq
    ...................cuuuuguacauGuagauuau.........................................................................  1       1    seq
    ...................cuuuuguacauGuagauuaucu.......................................................................  1       1    seq
    ...................cuuuuguacauuuagauuaucucu.....................................................................  1       0    seq
    ...................cuuuuguacauGuagauuaucucu.....................................................................  3       1    seq
    ....................uuuuguacauGuagauuau.........................................................................  15      1    seq
    ....................uuuuguacauGuagauuauc........................................................................  2       1    seq
    ....................uuuuguacauuuagauuaucA.......................................................................  1       1    seq
    ....................uuuuguacauuuagauuaucu.......................................................................  4       0    seq
    ....................uuuuguacauGuagauuaucu.......................................................................  59      1    seq
    ....................uuuuguacauuuagauuaucuc......................................................................  1       0    seq
    ....................uuuuguacauGuagauuaucuc......................................................................  11      1    seq
    ....................uuuuguacauAuagauuaucucu.....................................................................  1       1    seq
    ....................uuuuguacauuuagauuaucucG.....................................................................  2       1    seq
    ....................uuuuguacauuuagauuaucucu.....................................................................  37      0    seq
    ....................uuuuguacauGuagauuaucucu.....................................................................  327     1    seq
    ....................uuuuguacauuuagauuaucucC.....................................................................  7       1    seq
    ....................uuuuguacauuuagauuaucucA.....................................................................  2       1    seq
    ....................uuuuguacauuuagauuaucucuu....................................................................  2       0    seq
    ....................uuuuguacauGuagauuaucucuu....................................................................  10      1    seq
    ....................uuuuguacauuuagauuaucucuug...................................................................  2       0    seq
    ....................uuuuguacauGuagauuaucucuug...................................................................  3       1    seq
    ....................uuuuguacauGuagauuaucucuugcu.................................................................  1       1    seq
    ....................uuuuguacauGuagauuaucucuugcucu...............................................................  3       1    seq
    .....................uuuguacauGuagauuaucu.......................................................................  4       1    seq
    .....................uuuguacauGuagauuaucuc......................................................................  1       1    seq
    .....................uuuguacauGuagauuaucucu.....................................................................  25      1    seq
    .....................uuuguacauuuagauuaucucu.....................................................................  1       0    seq
    .....................uuuguacauGuagauuaucucuu....................................................................  3       1    seq
    .....................uuuguacauGuagauuaucucuug...................................................................  2       1    seq
    .....................uuuguacauGuagauuaucucuugc..................................................................  1       1    seq
```

```
                    novel-nve-miR-10_guide
                                                          novel-nve-miR-10_star
agucacacagauugauuugcuuuuguacauuuagauuaucucuugcucuuucaaagagcaaaucuacauguacaaaaacaaaucaaaagugcauucaaaagagaaaaaccga

......................uuguacauGuagauuaucucu.....................................................................    19    1    seq
......................uuguacauuuagauuaucucu.....................................................................    1     0    seq
......................uuguacauuuagauuaucucA.....................................................................    1     1    seq
.......................uguacauuuagauuaucuc......................................................................    1     0    seq
.......................uguacauGuagauuaucucu.....................................................................    4     1    seq
.......................uguacauuuagauuaucucu.....................................................................    1     0    seq
.......................uguacauuuagauuaucucuu....................................................................    1     0    seq
.......................uguacauuuagauuaucucuC....................................................................    1     1    seq
.......................uguacauGuagauuaucucuu....................................................................    3     1    seq
.......................uguacauGuagauuaucucuug...................................................................    1     1    seq
.......................uguacauuuagauuaucucuuU...................................................................    1     1    seq
.........................uacauGuagauuaucucuu....................................................................    1     1    seq
........................................................agcaaaucuacauguacaa.....................................    1     0    seq
.........................................................gcaaaucuacauguacaaaaaca................................    1     0    seq
.........................................................gcaaaucuacauguacaaaaacU................................    2     1    seq
..........................................................caaaucuacauguacaaaa...................................    1     0    seq
..........................................................caaaucuacauguacaaaaac.................................    5     0    seq
..........................................................caaaucuacauguacaaaaacC................................    1     1    seq
```

miRBase precursor : novel-nve-miR-11
Total read count : 62
novel-nve-miR-11_guide read count : 58
novel-nve-miR-11_star read count : 4
remaining reads : 0

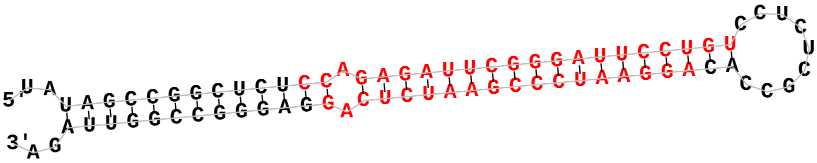

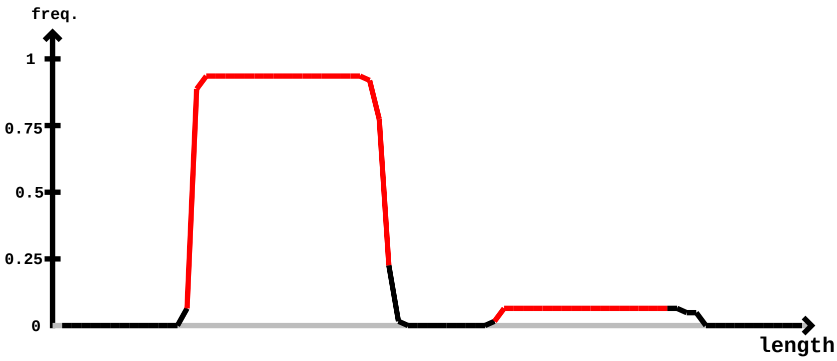


```
                               novel-nve-miR-11_star
                novel-nve-miR-11_guide
5'- uauagccggcucuccagagauucgggauuccuguccucucgccacaggaaucccgaaucucaggagggccgguuaga   -3'    exp
    ..(((((((((((((.((((((((((((((((((.........)))))))))))))))))).)))))))))))))..    reads     mm        sample
    ............uccagagauucgggauuccug............................................    2         0         seq
    ............uccGgagauucgggauuccug............................................    1         1         seq
    ............uccGgagauucgggauuccuguc..........................................    1         1         seq
    .............ccGgagauucgggauucc..............................................    1         1         seq
    .............ccagagauucgggauuccu.............................................    1         0         seq
    .............ccGgagauucgggauuccu.............................................    6         1         seq
    .............ccGgagauucgggauuccug............................................    28        1         seq
    .............ccUgagauucgggauuccug............................................    2         1         seq
    .............ccGgagauucgggauuccugu...........................................    13        1         seq
    ..............cGgagauucgggauuccu.............................................    2         1         seq
    ..............cGgagauucgggauuccug............................................    1         1         seq
    ............................................caggaaucccgaaucucagg.............    1         0         seq
    .............................................aggaaucccgaaucucaggag...........    1         0         seq
    .............................................aggaaucccgaaucucaggaU...........    1         1         seq
    .............................................aggaaucccgaaucucCggag...........    1         1         seq
```

miRBase precursor : novel-nve-miR-12
Total read count : 135
novel-nve-miR-12_guide read count : 121
novel-nve-miR-12_star read count : 8
remaining reads : 6

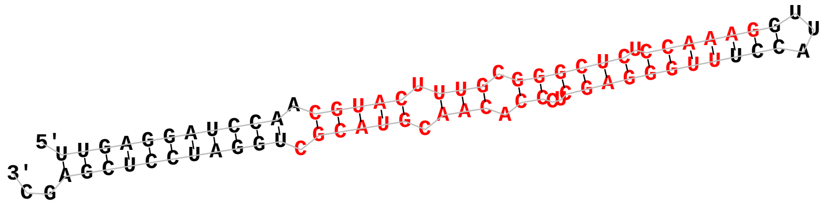

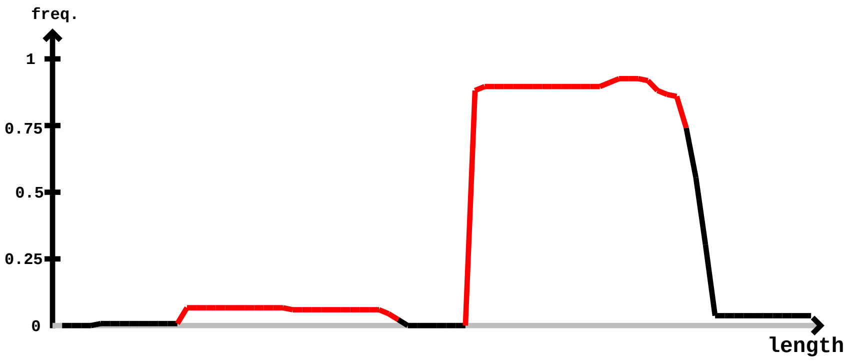


```
                          novel-nve-miR-12_star
                                                    novel-nve-miR-12_guide
5'- uugaggauccaacguacuuugcgggcucuccaaagguuaccuuugggagcucccacaacguacgcuggauccucgagc   -3'    exp
    ((((((((((.(((((.(((.((((((.(((((((...)))))))))))))..)).))).))))).))))))))))..   reads      mm         sample
    ...aggauccaGcguacuuugcg.......................................................   1          1          seq
    ............cguacuuugcgggcucuccaa.............................................   2          0          seq
    ............cguacuuugcgggcucuccaaa............................................   3          0          seq
    ............cguacuuugcgggcucuccaaaU...........................................   1          1          seq
    ............cguacuuugcgggcucuccaaag...........................................   2          0          seq
    ..........................................uugggagcucccacaacg..................   2          0          seq
    ..........................................uugggagcucccacaacgA.................   1          1          seq
    ..........................................uugggagcucccacaacgC.................   2          1          seq
    ..........................................uugggagcucccacaacgu.................   2          0          seq
    ..........................................uugggagcucccacaacgua................   2          0          seq
    ..........................................uugggagcucccacaacguac...............   1          0          seq
    ..........................................uugggagcucccacaacguacU..............   1          1          seq
    ..........................................uugggagcucccacaacguacA..............   2          1          seq
    ..........................................uugggagcucccacaacguacg..............   12         0          seq
    ..........................................uugAgagcucccacaacguacgc.............   1          1          seq
    ..........................................uugggagcucccacaacguacgU.............   9          1          seq
    ..........................................uugggagcucccacaacguacgA.............   3          1          seq
    ..........................................uugggagcucccacaacguacgc.............   12         0          seq
    ..........................................uugggagcucGcacaacguacgcu............   1          1          seq
    ..........................................uugggagcucccacaacguacgcA............   1          1          seq
    ..........................................uugggagcucccacaacguacgcu............   20         0          seq
    ..........................................uugggagcucccacaacguacgcG............   3          1          seq
    ..........................................uugggagcucccacaacguacgcC............   8          1          seq
    ..........................................uugggagcuAccacaacguacgcu............   1          1          seq
    ..........................................uugggagcucccacaacguacgcuU...........   28         1          seq
    ..........................................uugggagcucccacaacguacgcuC...........   7          1          seq
    ...........................................ugggagcucccacaacguacg..............   1          0          seq
    ...........................................ugggagcucccacaacguacgcuU...........   1          1          seq
    ........................................................aaAguacgcuggauccucgagc   2          1          seq
    .........................................................aAguacgcuggauccucgagc   2          1          seq
    ............................................................uacgcuggauccucgagc   1          0          seq
```

miRBase precursor : novel-nve-miR-13
Total read count : 98
novel-nve-miR-13_guide read count : 77
novel-nve-miR-13_star read count : 5
remaining reads : 16

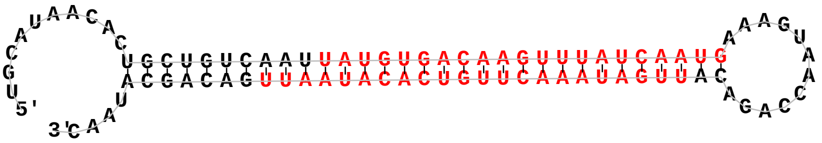

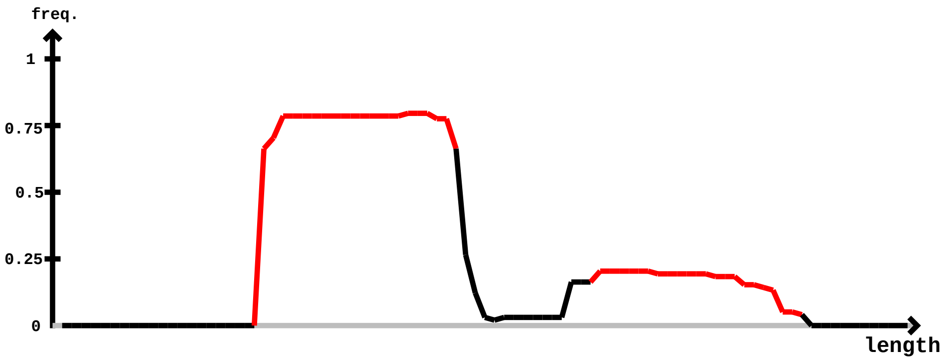


```
                                                                      novel-nve-miR-13_star
                        novel-nve-miR-13_guide
5'- ugcauaacacugcugucaauuaugugacaaguuuaucaaugaaaguaaccagacauugauaaacuugucacauaauugacagcauaac  -3'   exp
    ..........(((((((((((((((((((((((((((((((............)))))))))))))))))))))))))))))))....    reads  mm  sample
    ....................uaugugacaaguuuaucG..................................................    1      1   seq
    ....................uaugugacaaguuuaCca..................................................    1      1   seq
    ....................uaugugacaaguuuaucaa.................................................    1      0   seq
    ....................uaugugacaaguuuaucaau................................................    8      0   seq
    ....................uGugugacaaguuuaucaau................................................    1      1   seq
    ....................uaugGgacaaguuuaucaau................................................    1      1   seq
    ....................uaugCgacaaguuuaucaaug...............................................    1      1   seq
    ....................uaugugacaaguuuaCcaaug...............................................    2      1   seq
    ....................uaugugacaaguuuaucaaug...............................................    26     0   seq
    ....................uaugugacGaguuuaucaaug...............................................    1      1   seq
    ....................uaugugacaaguuuaucaauA...............................................    4      1   seq
    ....................uaugugacaaguuuaucaauga..............................................    2      0   seq
    ....................uaugugacaaguuuaucaaugU..............................................    8      1   seq
    ....................uaugugacaaguuuaucaaugC..............................................    1      1   seq
    ....................uaugugacaaguuuaucaaugaC.............................................    1      1   seq
    ....................uaugugacaaguuuaucaaugaU.............................................    6      1   seq
    .....................Gugugacaaguuuaucaaug...............................................    1      1   seq
    .....................augugacaaguuuaucaaug...............................................    1      0   seq
    .....................augugacaaguuuaucGaug...............................................    1      1   seq
    .....................augugacaaguuuaucaaugaCa............................................    1      1   seq
    ......................ugugacaaguuuaucaau................................................    1      0   seq
    ......................ugugaAaaguuuaucaaug...............................................    1      1   seq
    ......................ugugacaaguuuaucaaug...............................................    1      0   seq
    ......................ugugacaaguuuaucaaugU..............................................    3      1   seq
    ......................ugugacaaguuuaucaaugaU.............................................    2      1   seq
    ...................................ucaaugaaaguaaccagaca.................................    1      0   seq
    .......................................ugaaaguaaccagacauugaua...........................    1      0   seq
    .............................................uaaccagacauugauaaacuuU.....................    1      1   seq
    ....................................................Ucauugauaaacuuguca..................    3      1   seq
    ....................................................Ucauugauaaacuugucaca................    1      1   seq
    ....................................................Ucauugauaaacuugucacau...............    1      1   seq
    ....................................................Ucauugauaaacuugucacaua..............    8      1   seq
    .......................................................uugauaaacuugucacauaauu...........    1      0   seq
    .......................................................uugauaaacuugucacauaaCu...........    1      1   seq
```

```
                                                       novel-nve-miR-13_star
                    novel-nve-miR-13_guide
ugcauaacacugcugucaauuaugugacaaguuuaucaaugaaaguaaccagacauugauaaacuugucacauaauugacagcauaac

.......................................................uugauaaacuugucacauaauu...........    1          0          seq
.......................................................uugauGaacuugucacauaauu...........    2          1          seq
```

miRBase precursor : novel-nve-miR-14
Total read count : 115
novel-nve-miR-14_guide read count : 90
novel-nve-miR-14_star read count : 25
remaining reads : 0

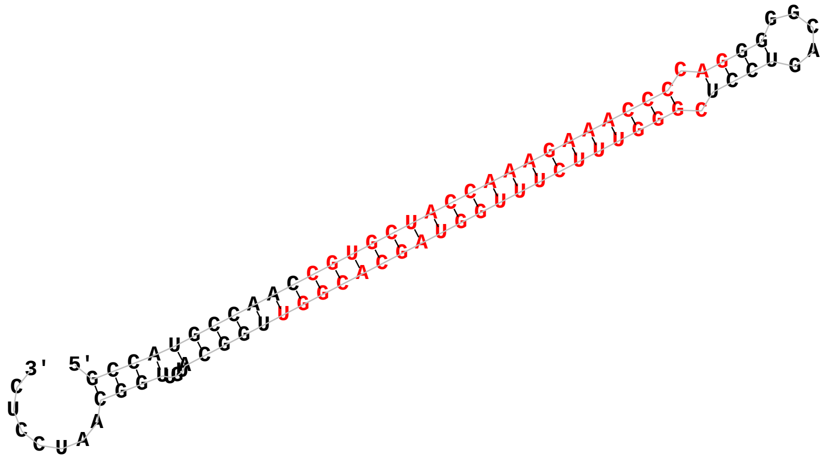

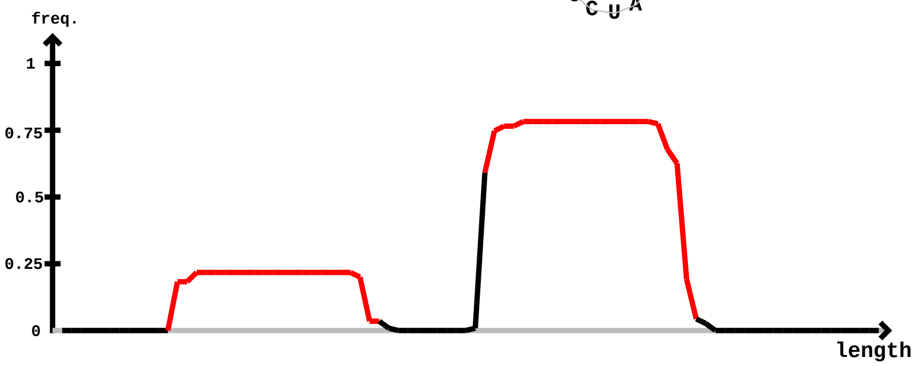


```
                                          novel-nve-miR-14_guide
               novel-nve-miR-14_star
5'- gccaugccaaccgugcuaccaaagaaaccccagggggcaguccucggguuucuuugguagcacgguuggcauguuggcaauccuc  -3'    exp
    ((((((((((((((((((((((((((((((.((((.....)))).)))))))))))))))))))))))))...)))).......    reads      mm         sample
    ...........cgugcuaccaaagaaaccc......................................................    1          0          seq
    ...........cgugcuaccaaagaaaccA......................................................    1          1          seq
    ...........cgugcuaccaaagaaacccU.....................................................    8          1          seq
    ...........cgugcuaccaaagaaacccc.....................................................    1          0          seq
    ...........cgugcuaccaaagaaacccG.....................................................    9          1          seq
    ...........cgugcuaccaaagaaacccA.....................................................    1          1          seq
    .............ugcuaccaaagaaaccccaA...................................................    1          1          seq
    .............ugcuaccaaagaaaccccag...................................................    2          0          seq
    .............ugcuaccaaagaaaccccaCg..................................................    1          1          seq
    .........................................cucggguuucuuugguagcacU.....................    1          1          seq
    ..........................................ucggguuucuuugguagG........................    1          1          seq
    ..........................................ucggguuucuuugguagca.......................    2          0          seq
    ..........................................ucggguuucuuugguagUa.......................    1          1          seq
    ..........................................ucggguuucuuugguagcG.......................    8          1          seq
    ..........................................ucggguuucuuugguagcac......................    2          0          seq
    ..........................................ucggguuucuuugguagcaU......................    1          1          seq
    ..........................................ucggguuucuuugguagcacg.....................    25         0          seq
    ..........................................ucggguuucuuugguagcacA.....................    2          1          seq
    ..........................................ucggguuuUuuugguagcacg.....................    1          1          seq
    ..........................................uGggguuucuuugguagcacg.....................    8          1          seq
    ..........................................ucggguuucuuugguagcacC.....................    2          1          seq
    ..........................................ucggguuucuuugguagcacU.....................    6          1          seq
    ..........................................ucggguuucuuugguagcacgC....................    1          1          seq
    ..........................................ucggguuucuuugguagcacgU....................    1          1          seq
    ..........................................uGggguuucuuugguagcacgg....................    1          1          seq
    ..........................................ucggguuucuuugguagcacgA....................    1          1          seq
    ..........................................ucggguuucuuugguagcacgg....................    4          0          seq
    ...........................................cggguuucuuugguagcac......................    3          0          seq
    ...........................................cgggCuucuuugguagcacg.....................    1          1          seq
    ...........................................cggguuucuuugguaUcacg.....................    1          1          seq
    ...........................................cggguuucuuugguagcacg.....................    3          0          seq
    ...........................................cggguuucuuugguagcacgg....................    6          0          seq
    ...........................................cggguuucuuugguagcacggu...................    2          0          seq
    ...........................................cggguuucuuugguagcacgguA..................    1          1          seq
```

```
                                            novel-nve-miR-14_guide
           novel-nve-miR-14_star
gccaugccaaccgugcuaccaaagaaaccccagggggcaguccucggguuucuuugguagcacgguuggcauguuggcaauccuc

............................................cggguuucuuugguagcacgguu..................    1          0          seq
.............................................ggguuucuuugguagcacgg....................    1          0          seq
.............................................ggguuucuuugguagcacgU....................    1          1          seq
...............................................guuucuuugguagcacgg....................    1          0          seq
...............................................guuucuuugguagcacgguu..................    1          0          seq
```

miRBase precursor : novel-nve-miR-15
Total read count : 545
novel-nve-miR-15_guide read count : 538
novel-nve-miR-15_star read count : 7
remaining reads : 0

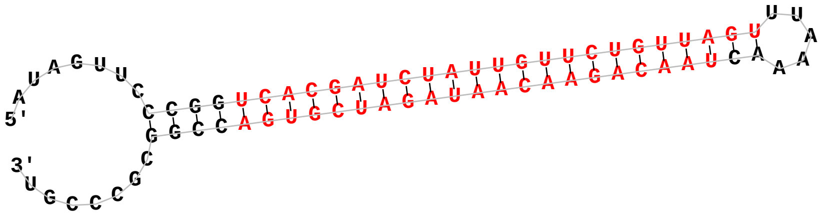

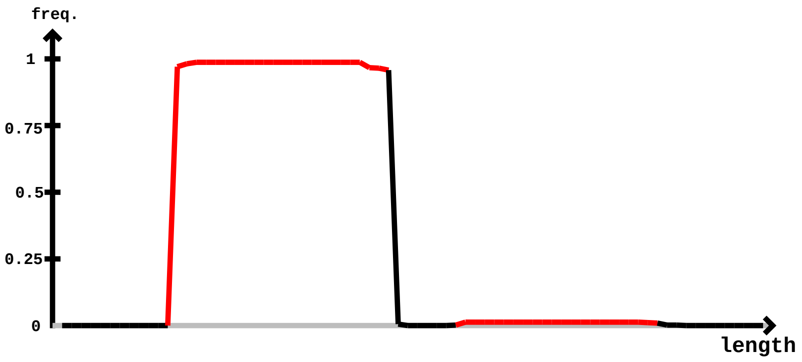


```
                                novel-nve-miR-15_guide
                                                            novel-nve-miR-15_star
5'- auaguucccggucacgaucuauuguucuguuaguuuaaaacuaacagaacaauagaucgugaccggcgcccgu   -3'    exp
    ...(..((((((((((((((((((((((((((((.....))))))))))))))))))))))))))).)..)..   reads   mm   sample
    ...........ucacgaucuauuguucuguu..........................................   7       0    seq
    ...........ucacgaucuauuguucuguC..........................................   3       1    seq
    ...........ucacgaucuauuguucugCu..........................................   1       1    seq
    ...........ucacgaucuauuguucuguua.........................................   1       0    seq
    ...........ucacgaucuauuguucuguuaC........................................   1       1    seq
    ...........ucacgaucuauuguucuguuaU........................................   3       1    seq
    ...........ucacgaucCauuguucuguuagu.......................................   4       1    seq
    ...........ucacgaucuauuguucugGuagu.......................................   1       1    seq
    ...........ucacgaucuauuguucuguuagG.......................................   12      1    seq
    ...........Acacgaucuauuguucuguuagu.......................................   5       1    seq
    ...........ucGcgaucuauuguucuguuagu.......................................   1       1    seq
    ...........ucacgaCcuauuguucuguuagu.......................................   2       1    seq
    ...........ucacgaucuauuguucCguuagu.......................................   1       1    seq
    ...........ucacgaucuauuguucGguuagu.......................................   1       1    seq
    ...........ucacgaucuauuguAcuguuagu.......................................   1       1    seq
    ...........uUacgaucuauuguucuguuagu.......................................   1       1    seq
    ...........ucacgaucuauuguuUuguuagu.......................................   1       1    seq
    ...........ucaUgaucuauuguucuguuagu.......................................   1       1    seq
    ...........ucacgaucuauCguucuguuagu.......................................   1       1    seq
    ...........ucacgaucuGuuguucuguuagu.......................................   1       1    seq
    ...........Ccacgaucuauuguucuguuagu.......................................   4       1    seq
    ...........ucacgGucuauuguucuguuagu.......................................   2       1    seq
    ...........ucacgaucuauuguCcuguuagu.......................................   2       1    seq
    ...........ucacgaucuauuguucugAuagu.......................................   1       1    seq
    ...........Gcacgaucuauuguucuguuagu.......................................   1       1    seq
    ...........ucacgaucuauuguucuguuagC.......................................   95      1    seq
    ...........ucacgaucuauugCucuguuagu.......................................   1       1    seq
    ...........ucaAgaucuauuguucuguuagu.......................................   1       1    seq
    ...........ucacgaucuauuguucuguuagA.......................................   2       1    seq
    ...........ucacgaucuauuguucuguuagu.......................................   368     0    seq
    ...........ucUcgaucuauuguucuguuagu.......................................   1       1    seq
    ...........ucacgaucuauuguucuguuaguu......................................   1       0    seq
    ...........ucacgaucuauuguucuguuaguC......................................   1       1    seq
    ............cacgaucuauuguucuguuagA.......................................   1       1    seq
```

```
           novel-nve-miR-15_guide
                                         novel-nve-miR-15_star
auaguucccggucacgaucuauuguucuguuaguuuaaaacuaacagaacaauagaucgugaccggcgcccgu

............cacgaucuauuguucuguuagu.......................................    5    0    seq
.............acgaucuauuguucuguuagu.......................................    2    0    seq
.............acgaucuauuguucuguuaguC......................................    1    1    seq
........................................cuaacagaacaauagaucgG.............    1    1    seq
.........................................uaacagaacaauagaucgug............    1    0    seq
.........................................uaacagaUcaauagaucguga...........    1    1    seq
.........................................uaacagaacaauagaucguga...........    3    0    seq
.........................................Aaacagaacaauagaucgugacc.........    1    1    seq
```

miRBase precursor : novel-nve-miR-16
Total read count : 333
novel-nve-miR-16_guide read count : 249
novel-nve-miR-16_star read count : 79
remaining reads : 5

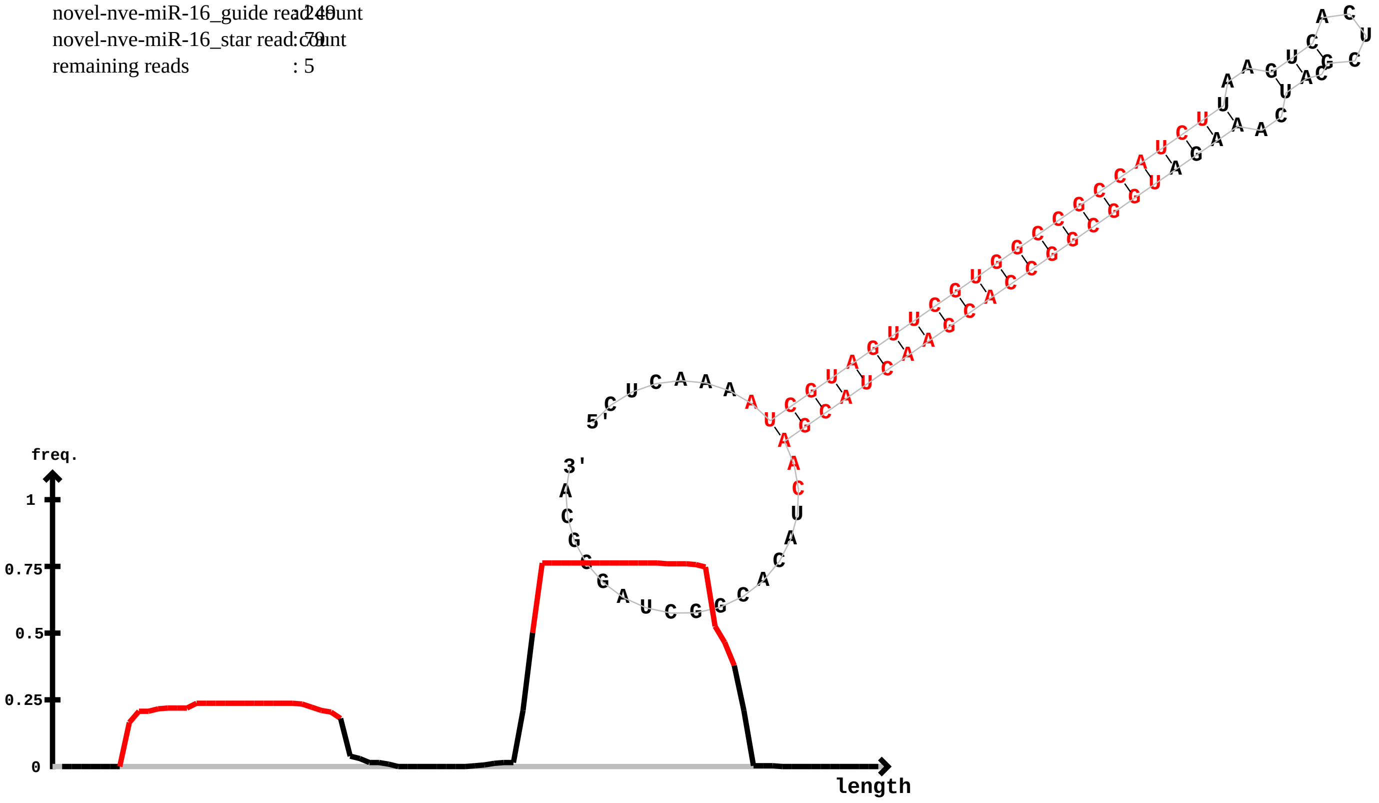


```
                                                           novel-nve-miR-16_guide
      novel-nve-miR-16_star
5'- cucaaaaucguaguucguggccgccaucuuaagucacucgcaucaaagauggcggccacgaacuacgaacuacacggcuagggca  -3'   exp
    .......((((((((((((((((((((((..(((....).))..)))))))))))))))))))))))........((....)).   reads   mm   sample
    ......aucguaguucguggccgc.............................................................   1       0    seq
    ......aucguaguucguggccgcU............................................................   1       1    seq
    ......aucguaguucguggccgcG............................................................   1       1    seq
    ......aucguaguucguggccgcc............................................................   1       0    seq
    ......aucguaguucguggccgcGa...........................................................   1       1    seq
    ......aucguaguucguggccgccG...........................................................   2       1    seq
    ......aucguaguucguggccgcca...........................................................   1       0    seq
    ......aucguaguucguggccgccau..........................................................   2       0    seq
    ......aucguaguucguggccgccauA.........................................................   2       1    seq
    ......aucguaguucguggccgccauc.........................................................   2       0    seq
    ......aucguaguucguggccgccaucA........................................................   2       1    seq
    ......aucguaguucguggccgccGucu........................................................   1       1    seq
    ......aucguaguucguggccgccaucG........................................................   13      1    seq
    ......aucguaguucguggccgccaucC........................................................   5       1    seq
    ......aucguaguucguUgccgccaucu........................................................   1       1    seq
    ......aucguaguucguggccgccaucu........................................................   16      0    seq
    ......aucguaguucguggcUgccaucu........................................................   1       1    seq
    ......aucguaguucguggccgccaucuu.......................................................   1       0    seq
    ......aucguaguucguggccgccaucuuG......................................................   1       1    seq
    .......ucguaguucguggccgcc............................................................   1       0    seq
    .......ucguaguucguggccgccaCc.........................................................   1       1    seq
    .......ucguaguucguggccgccauU.........................................................   1       1    seq
    .......ucguaguucguggccgccauc.........................................................   2       0    seq
    .......ucguaguucguggccgccaucC........................................................   1       1    seq
    .......ucguaguucguggccgccaucG........................................................   5       1    seq
    .......ucguaguucguggccgccaucu........................................................   2       0    seq
    .......ucguaguucguggccgccaucuG.......................................................   1       1    seq
    .........guaguucguggccgccaucuu.......................................................   1       0    seq
    .........guaguucguggccgccaucuuU......................................................   2       1    seq
    ..........uaguucguggccgccaucuuU......................................................   1       1    seq
    ............uucguggccgccaucuuG.......................................................   1       1    seq
    ............uucguggccgccaucuuaag.....................................................   2       0    seq
    ............uucguggccgccaucuuaagG....................................................   1       1    seq
    ............uucguggccgccaucuuaagu....................................................   2       0    seq
```

```
                                                novel-nve-miR-16_guide
      novel-nve-miR-16_star
cucaaaaucguaguucguggccgccaucuuaagucacucgcaucaaagauggcggccacgaacuacgaacuacacggcuagggca

..........................................ucaaagauggcggccacgaU.......................    1    1    seq
...........................................cCaagauggcggccacgaacua....................    1    1    seq
............................................Uaagauggcggccacgaacuac...................    1    1    seq
............................................Uaagauggcggccacgaacuacg..................    1    1    seq
.............................................aagauggcggccacgaacuaU...................    1    1    seq
...............................................gauggcggccacgaacuaG...................    1    1    seq
...............................................gauggcggccacgaacuacg..................    27   0    seq
...............................................gauggcggccacgaacuacC..................    1    1    seq
...............................................gauggcggUcacgaacuacg..................    1    1    seq
...............................................gauggcggccacgaGcuacg..................    1    1    seq
...............................................gauggcggccCgaacuacg...................    1    1    seq
...............................................gauggcggccacAaacuacg..................    1    1    seq
...............................................gauggcggccacgaacuacA..................    1    1    seq
...............................................gauggcggccacgaacuacgG.................    4    1    seq
...............................................gauggcggccacgCacuacga.................    1    1    seq
...............................................gauggcggccacgaGcuacga.................    1    1    seq
...............................................gauggcggccacgaacuacga.................    6    0    seq
...............................................gauggcggccacgaacuacgaG................    1    1    seq
...............................................gauggcggccacgaacuacgaa................    8    0    seq
...............................................gauggcggccacgaacuacgaU................    1    1    seq
...............................................gauggcggccacgaacuacgaUc...............    1    1    seq
...............................................gauggcggccacgaacuacgaaU...............    4    1    seq
...............................................gauggcggcUacgaacuacgaac...............    2    1    seq
...............................................gauggcggccacgaacuacgaaUu..............    2    1    seq
................................................auggcggAcacgaacuacg..................    4    1    seq
................................................auggcggccacgaacuacC..................    1    1    seq
................................................auggcggccacgaacuacA..................    1    1    seq
................................................auggcggccacgaacuacg..................    24   0    seq
................................................auggcggccacgaacuacgG.................    2    1    seq
................................................auggcggccacgaacuacga.................    3    0    seq
................................................auggcggccaUgaacuacgaa................    1    1    seq
................................................auggcggccacgaacuacgaa................    7    0    seq
................................................auggcggccacgaacuacgaG................    3    1    seq
................................................auggcggccacgaacuacgaU................    3    1    seq
................................................aAggcggccacgaacuacgaa................    2    1    seq
................................................auggcggccacgaacuacgaUc...............    1    1    seq
................................................auggcggccacgaacuacgaaU...............    9    1    seq
................................................auggcggccacgaacuacgaaG...............    1    1    seq
................................................auggcggccacgaacuacgaac...............    8    0    seq
................................................auggcgUccacgaacuacgaacu..............    1    1    seq
................................................auggcggccacgaacuacgaacu..............    5    0    seq
................................................auggcggccacgaacuacgaaUu..............    2    1    seq
................................................auggcggccacgaacuacgaacG..............    13   1    seq
................................................auggcggccacgaacuacgaacA..............    2    1    seq
................................................auggcggccacgaacuacgaacC..............    2    1    seq
................................................Guggcggccacgaacuacgaacu..............    1    1    seq
................................................auggcggccacgaacuacgaacuacG...........    1    1    seq
.................................................uggcggccacgaacuacg..................    8    0    seq
.................................................uggcggccacgaacuacA..................    1    1    seq
.................................................uggcggccacgaaAuacg..................    1    1    seq
.................................................uggcggccacgaacuacga.................    3    0    seq
.................................................uggcggccacgaacuacgaG................    1    1    seq
.................................................uggcggccacgaacuacgaU................    2    1    seq
.................................................uggcggccacgaacuacgaaU...............    22   1    seq
.................................................uggcggccacgaacuacgaUc...............    2    1    seq
.................................................uggcggccacgaacuacgaac...............    1    0    seq
.................................................uggcggccacgaacuacgaaA...............    2    1    seq
.................................................uggcggccacgaacuacgaaG...............    3    1    seq
.................................................uggcggccacgaacuacgaaUu..............    2    1    seq
.................................................uggcggccacgaacuacgaacA..............    1    1    seq
.................................................uggcggUcacgaacuacgaacu..............    1    1    seq
.................................................uggcggccacgaacuacgaacC..............    2    1    seq
.................................................uggcggccacgaacuacgaacu..............    15   0    seq
.................................................uggcggccacgaacuacgaacG..............    20   1    seq
```

miRBase precursor : novel-nve-miR-18
Total read count : 233
novel-nve-miR-18_guide read count : 224
novel-nve-miR-18_star read count : 9
remaining reads : 0

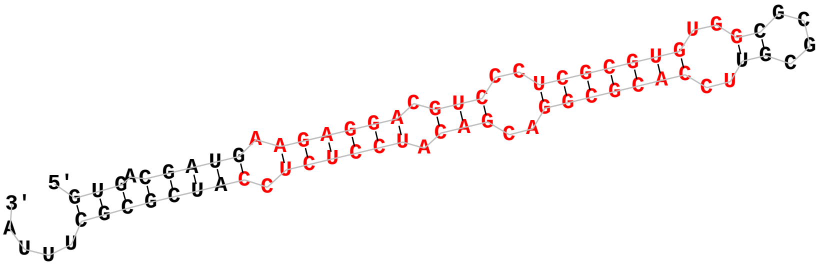

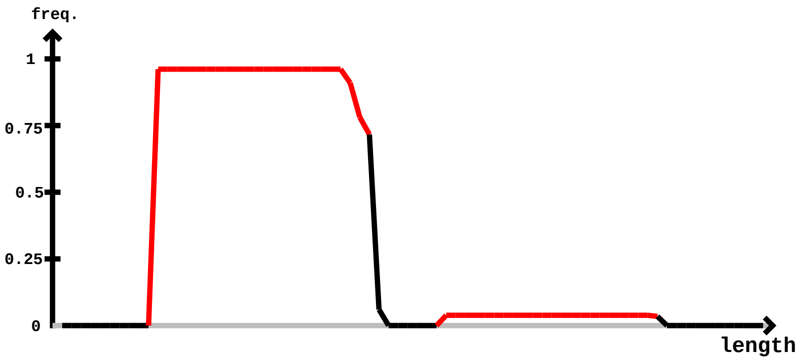


```
                                    novel-nve-miR-18_star
          novel-nve-miR-18_guide
5'- gugacgaugaagaggacgucccucgcgugugggcgcgcguuccacgcggacgacauccucuccaucgcgcuuua   -3'    exp
    (((.(((((.((((((.(((..(((((((..((....))..)))))))..))).)))))).))))))))....   reads     mm      sample
    .........aagaggacgucccucgcguA............................................   1         1       seq
    .........aagaggacgucccucgUgug............................................   1         1       seq
    .........aagaggacgucccucgcgug............................................   10        0       seq
    .........aagaggacgucccucgcgugu...........................................   21        0       seq
    .........Gagaggacgucccucgcgugu...........................................   2         1       seq
    .........aagaggacgucccucgcgugC...........................................   3         1       seq
    .........aagaggacgucccucgcAugu...........................................   1         1       seq
    .........aagaggacgCcccucgcgugu...........................................   1         1       seq
    .........aagagAacgucccucgcgugu...........................................   1         1       seq
    .........aagaggacgucccucgcgugA...........................................   1         1       seq
    .........aagaggacgucccucgcgugug..........................................   14        0       seq
    .........aagaggacgucccucgcguguA..........................................   1         1       seq
    .........aagaggacgucccucgcgugugA.........................................   17        1       seq
    .........aagaggaUgucccucgcgugugg.........................................   1         1       seq
    .........aagaggacgucccucCcgugugg.........................................   1         1       seq
    .........aagaggacguccUucgcgugugg.........................................   1         1       seq
    .........aagaggacgCcccucgcgugugg.........................................   1         1       seq
    .........aagaggacAucccucgcgugugg.........................................   3         1       seq
    .........aagaggacgucccCcgcgugugg.........................................   1         1       seq
    .........aagaggacgucccuUgcgugugg.........................................   1         1       seq
    .........aaCaggacgucccucgcgugugg.........................................   1         1       seq
    .........aagaggacgucccucgcgugugg.........................................   116       0       seq
    .........aagaggacgucccucgcgugugC.........................................   2         1       seq
    .........aagaggacgucccucgcgugugU.........................................   7         1       seq
    .........aagaggaAgucccucgcgugugg.........................................   1         1       seq
    .........aagaggacgucccucgcguguggU........................................   9         1       seq
    .........aagaggacgucccucgcguguggc........................................   1         0       seq
    .........aagaggacgucccucgcguguggA........................................   4         1       seq
    .......................................uccacgcggacgGcauccucuc............   1         1       seq
    .......................................uccacgcggacgacauccucucc...........   5         0       seq
    .......................................uccacgcggacgacauccucucA...........   1         1       seq
    .......................................uccaUgcggacgacauccucucc...........   1         1       seq
    .......................................uccacgcggacgacauccucucU...........   1         1       seq
```

miRBase precursor : novel-nve-miR-19
Total read count : 1469
novel-nve-miR-19_guide read count : 1374
novel-nve-miR-19_star read count : 82
remaining reads : 13

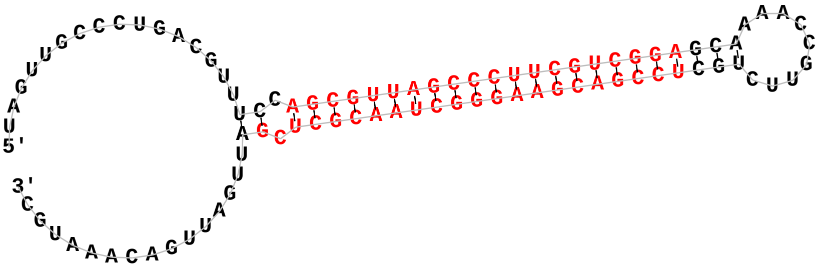

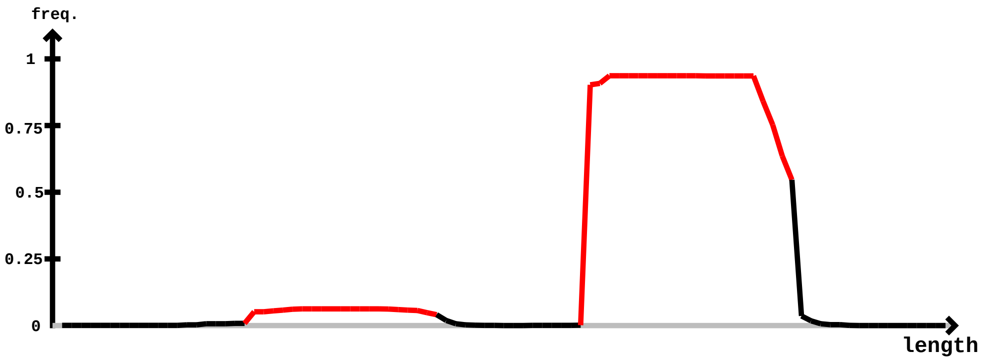


```
                novel-nve-miR-19_star
                                                   novel-nve-miR-19_guide
5'- uaguugcccugacguuuccagcguuagcccuucgucggagcaaaaccguucugcuccgacgaagggcuaacgcucgauugauugacaaaugc  -3'    exp
    ..(((.....)))...((.(((((((((((((((((((((((.........))))))))))))))))))))))).))...............   reads    mm    sample
    uGguugcccugacguuuc..........................................................................   1        1     seq
    ............cguuucGagcguuagcccuuc...........................................................   1        1     seq
    ............cguuucGagcguuagcccuucg..........................................................   2        1     seq
    ..............uuucGagcguuagcccuucg..........................................................   1        1     seq
    ..............uuucGagcguuagcccuucgu.........................................................   3        1     seq
    ..............uuucGagcguuagcccuucguc........................................................   2        1     seq
    .................cGagcguuagcccuucgucg.......................................................   1        1     seq
    .................cGagcguuagcccuucgucgga.....................................................   1        1     seq
    ..................Gagcguuagcccuucgucgg......................................................   1        1     seq
    ...................agcguuagcccuucgucg.......................................................   1        0     seq
    ...................agcguuGgcccuucgucg.......................................................   10       1     seq
    ...................agcguuagcccuucgucgg......................................................   2        0     seq
    ...................agcguuGgcccuucgucgg......................................................   8        1     seq
    ...................agcguuagcccuucgucggG.....................................................   2        1     seq
    ...................agcguuGgcccuucgucgga.....................................................   25       1     seq
    ...................agcguuagcccuucgucgga.....................................................   3        0     seq
    ...................agcguuGgcccuucgucggag....................................................   9        1     seq
    ...................agcguuagcccuucgucggaA....................................................   2        1     seq
    ...................agcguuagcccuucgucggaUc...................................................   1        1     seq
    ...................agcguuagcccuucgucggagUa..................................................   1        1     seq
    .....................cguuGgcccuucgucgga.....................................................   1        1     seq
    .....................cguuagcccuucgucggag....................................................   2        0     seq
    .....................cguuagccUuucgucggag....................................................   1        1     seq
    .....................cguuGgcccuucgucggag....................................................   1        1     seq
    ......................guuagcccuucgucggag....................................................   3        0     seq
    ......................guuagcccuucgucggagA...................................................   1        1     seq
    .......................uuagcccuucgucggagU...................................................   1        1     seq
    .......................uuagcccuucgucggagG...................................................   3        1     seq
    .......................uuGgcccuucgucggagca..................................................   1        1     seq
    ........................uagcccuucgucggagcaa.................................................   1        0     seq
    ........................uGgcccuucgucggagcaaaa...............................................   1        1     seq
    ................................................uuUugcuccgacgaaggg..........................   1        1     seq
    .....................................................cuccgacgaagggcuaacgG...................   1        1     seq
    ......................................................uccgacgaGgggcuaacg....................   1        1     seq
```

```
                   novel-nve-miR-19_star
                                                      novel-nve-miR-19_guide
uaguugcccugacguuuccagcguuagcccuucgucggagcaaaaccguucugcuccgacgaagggcuaacgcucgauugauugacaaaugc

......................................................uccgacgaagggcuaGcg....................    15    1    seq
......................................................uccgacgaagggcuaacC....................    2     1    seq
......................................................uccgacgaagggcuCacg....................    1     1    seq
......................................................uccgacgaagggcuaacA....................    9     1    seq
......................................................ucGgacgaagggcuaacg....................    1     1    seq
......................................................uccgacgaagggcuaacg....................    58    0    seq
......................................................uccgacgaagggcuGacg....................    36    1    seq
......................................................uccgacgaagggcuaacU....................    16    1    seq
......................................................uccgacgaagggcuaacgc...................    11    0    seq
......................................................uccgacgaagggcuGacgc...................    3     1    seq
......................................................uccgaAgaagggcuaacgc...................    1     1    seq
......................................................uccgacgaagggcuaacUc...................    1     1    seq
......................................................uccgacgaagggcuaacgG...................    31    1    seq
......................................................uccgacgaagggcuaacAc...................    1     1    seq
......................................................uccgacgGagggcuaacgc...................    1     1    seq
......................................................uccgacgaagggcuaacgU...................    8     1    seq
......................................................uccgacgaagggcuaacgA...................    69    1    seq
......................................................uccgacgaagggcuaacgcG..................    30    1    seq
......................................................uccgacgaagggcuaaAgcu..................    1     1    seq
......................................................uccgacgaagggcuaaUgcu..................    1     1    seq
......................................................uccgacgaagggcuaacgcA..................    10    1    seq
......................................................uccgacgaagggcuaGcgcu..................    6     1    seq
......................................................uccgacgaagggcuGacgcu..................    11    1    seq
......................................................uccgacgGagggcuaacgcu..................    1     1    seq
......................................................uccgacgaagggcuaacgAu..................    52    1    seq
......................................................uccgacgaagggcuaacCcu..................    1     1    seq
......................................................uccgacgaagggcuaacgcC..................    7     1    seq
......................................................Cccgacgaagggcuaacgcu..................    1     1    seq
......................................................uccgacgaagggcuaacgcu..................    50    0    seq
......................................................uccgacgaagggcuaacgcuG.................    16    1    seq
......................................................uccgacgaagggcuaacgcuc.................    33    0    seq
......................................................uccgacgGagggcuaacgcuc.................    1     1    seq
......................................................uccgacgaGgggcuaacgcuc.................    2     1    seq
......................................................uccgacgaagggcuGacgcuc.................    11    1    seq
......................................................uccgGcgaagggcuaacgcuc.................    4     1    seq
......................................................uccgacgaagggcuaacgcuU.................    21    1    seq
......................................................uccgacgaagggcuaCcgcuc.................    1     1    seq
......................................................uccgacgaagggcuaacgcuA.................    25    1    seq
......................................................uccgacgaagggcuaGcgcuc.................    14    1    seq
......................................................uAcgacgaagggcuaacgcuc.................    1     1    seq
......................................................uccgacgaagggcuaacgcuAg................    12    1    seq
......................................................uccgacgaagggcuaacAcucg................    2     1    seq
......................................................uccgacgaagggcuaUcgcucg................    3     1    seq
......................................................uccgacgaagggcuaacgcucC................    8     1    seq
......................................................Cccgacgaagggcuaacgcucg................    3     1    seq
......................................................uccgaAgaagggcuaacgcucg................    1     1    seq
......................................................uUcgacgaagggcuaacgcucg................    1     1    seq
......................................................uccgacgaagggcCaacgcucg................    2     1    seq
......................................................uccgacgaUgggcuaacgcucg................    1     1    seq
......................................................uccgacgaagggcuaacgcAcg................    13    1    seq
......................................................Gccgacgaagggcuaacgcucg................    1     1    seq
......................................................Accgacgaagggcuaacgcucg................    6     1    seq
......................................................uccAacgaagggcuaacgcucg................    1     1    seq
......................................................uccgacgGagggcuaacgcucg................    1     1    seq
......................................................uccgacgaagggcuaacgcuUg................    4     1    seq
......................................................uccgacgaGgggcuaacgcucg................    2     1    seq
......................................................ucUgacgaagggcuaacgcucg................    2     1    seq
......................................................uAcgacgaagggcuaacgcucg................    1     1    seq
......................................................ucAgacgaagggcuaacgcucg................    2     1    seq
......................................................uccgacgaagggcuaacgcucU................    22    1    seq
......................................................uccgacgaagggcuaacgcucA................    86    1    seq
......................................................uccgacgaagggcuGacgcucg................    92    1    seq
......................................................uccgacgaagggUuaacgcucg................    1     1    seq
......................................................uccgacgaagggcuaaGgcucg................    1     1    seq
......................................................uccgacgaagggcuaacgcucg................    315   0    seq
......................................................uccgacgaagggcuaGcgcucg................    117   1    seq
......................................................uccgGcgaagggcuaacgcucg................    2     1    seq
......................................................uccgacgaagggcuaacgUucg................    1     1    seq
......................................................uccgUcgaagggcuaacgcucg................    2     1    seq
```

novel-nve-miR-19_star

novel-nve-miR-19_guide

```
uaguugcccugacguuuccagcguuagcccuucgucggagcaaaaccguucugcuccgacgaagggcuaacgcucgauugauugacaaaugc

......................................................uccCacgaagggcuaacgcucg................    1    1    seq
......................................................uccgacgaagggcuGacgcucga...............    2    1    seq
......................................................uccgacgaGgggcuaacgcucga...............    3    1    seq
......................................................uccgacgaagggcuaacgcucgU...............    3    1    seq
......................................................uccgacgaagggcuaacgcucgG...............    3    1    seq
......................................................uccgacgaagggcuaacgcucUa...............    1    1    seq
......................................................uccgacgaagggcuaacgcucga...............    11   0    seq
......................................................uccgacgaagggcuaacgcucgC...............    1    1    seq
......................................................uccgacgaagggcuaacgcucAa...............    3    1    seq
......................................................uccgacgaagggcuGacgcucgau..............    2    1    seq
......................................................uccgacgaagggcuaacgcucgau..............    6    0    seq
......................................................uccgacgaagggcuaacgcucgaG..............    4    1    seq
......................................................uccgacgaagggcuaacgcucgaC..............    4    1    seq
......................................................uccgacgaagggcuGacgcucgauu.............    2    1    seq
......................................................uccgacgaagggcuaacgcucgauA.............    1    1    seq
......................................................uccgacgaagggcuaacgcucgauu.............    1    0    seq
......................................................uccgacgaagggcuaacgcucgUuu.............    1    1    seq
......................................................uccgacgaagggcuaacgcucgauuAa...........    2    1    seq
......................................................uccgacgaagggcuaacgcucgauuUa...........    2    1    seq
......................................................uccgacgaagggcuaacgcucgauuUau..........    1    1    seq
.......................................................ccgacgaagggcuaacgA...................    3    1    seq
.......................................................ccgacgaagggcuaacgcucA................    1    1    seq
.......................................................ccgacgaagggcuaacgcucg................    1    0    seq
.......................................................ccgacgaagggcuaGcgcucg................    3    1    seq
........................................................cgacgaagggcuaacgcu..................    1    0    seq
........................................................cgacgaagggcuaacgcuU.................    2    1    seq
........................................................cgaAgaagggcuaacgcucg................    2    1    seq
........................................................cgacgaagggcuaacgcucg................    24   0    seq
........................................................cgacgaagggcuaGcgcucg................    1    1    seq
........................................................cgacgaaAggcuaacgcucg................    1    1    seq
........................................................Agacgaagggcuaacgcucg................    5    1    seq
........................................................cgacgaagggcuUacgcucg................    1    1    seq
........................................................cgacgaagggcuGacgcucg................    3    1    seq
........................................................cgacgaagggcuaacgcucA................    2    1    seq
```

miRBase precursor : novel-nve-miR-20
Total read count : 929
novel-nve-miR-20_guide read count : 926
novel-nve-miR-20_star read count : 0
remaining reads : 3

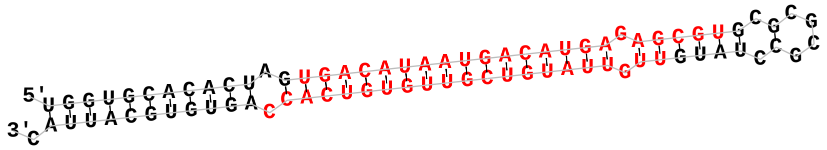

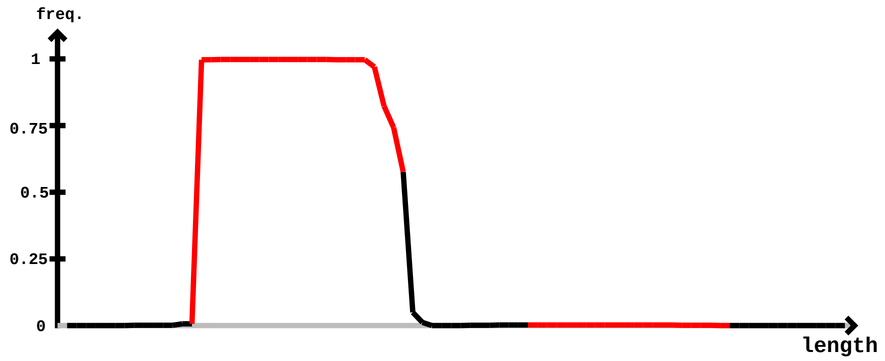


```
                    novel-nve-miR-20_guide
                                              novel-nve-miR-20_star
5'- uggugcacacuagugacauaaugacaugagagcgugcgcgcgccuauguuguuaugucguugugucaccagugugcauuac  -3'   exp
    (((((((((((.(((((((((((((((((.((((((.(....).)))))).))))))))))))))))).))))))))))).   reads   mm   sample
    ......acacuagugacaCaaugacau......................................................   1       1    seq
    ...........agugacauCaugacaugagag.................................................   1       1    seq
    ...........agugacauaaugacaugagaA.................................................   2       1    seq
    ...........agugacauaaugacaugagag.................................................   2       0    seq
    .............ugacauaaugacaugaga..................................................   23      0    seq
    .............ugGcauaaugacaugaga..................................................   1       1    seq
    .............Agacauaaugacaugaga..................................................   1       1    seq
    .............Ggacauaaugacaugagag.................................................   1       1    seq
    .............ugacauaaugacaugagag.................................................   104     0    seq
    .............Agacauaaugacaugagag.................................................   1       1    seq
    .............ugacauaaAgacaugagag.................................................   1       1    seq
    .............ugacauaaugacaugagGg.................................................   2       1    seq
    .............ugacauaauCacaugagag.................................................   1       1    seq
    .............ugacauaaugacGugagag.................................................   1       1    seq
    .............ugacauaaugacaugagaU.................................................   2       1    seq
    .............ugacauaaugacaugaAag.................................................   1       1    seq
    .............ugacauaaugacaugagaA.................................................   14      1    seq
    .............Cgacauaaugacaugagag.................................................   1       1    seq
    .............ugGcauaaugacaugagag.................................................   2       1    seq
    .............ugacauaaugGcaugagagc................................................   1       1    seq
    .............ugacaAaaugacaugagagc................................................   1       1    seq
    .............Agacauaaugacaugagagc................................................   2       1    seq
    .............ugacauaaugacaugagagU................................................   16      1    seq
    .............ugacauaaugacaugUgagc................................................   2       1    seq
    .............ugacauaaugacaugagagA................................................   4       1    seq
    .............ugacauaaugacaugagagc................................................   49      0    seq
    .............Agacauaaugacaugagagcg...............................................   4       1    seq
    .............uAacauaaugacaugagagcg...............................................   1       1    seq
    .............uCacauaaugacaugagagcg...............................................   1       1    seq
    .............ugacauaaugacaCgagagcg...............................................   1       1    seq
    .............Ggacauaaugacaugagagcg...............................................   2       1    seq
    .............ugacauaaugacaugagagcU...............................................   9       1    seq
    .............ugacauaaugacaugagagcC...............................................   2       1    seq
    .............ugacauaaugacaugagagcA...............................................   33      1    seq
```

```
             novel-nve-miR-20_guide
                                                  novel-nve-miR-20_star
uggugcacacuagugacauaaugacaugagagcgugcgcgcgccuauguuguuaugucguugugucaccagugugcauuac

.............ugacGuaaugacaugagagcg...............................................    1     1     seq
.............ugacauaaugacaugagagUg...............................................    1     1     seq
.............ugUcauaaugacaugagagcg...............................................    1     1     seq
.............ugacauaaugacaugagagcg...............................................    99    0     seq
.............ugacauaaugacaugagaUcgu..............................................    3     1     seq
.............ugaAauaaugacaugagagcgu..............................................    1     1     seq
.............Ggacauaaugacaugagagcgu..............................................    1     1     seq
.............ugacauaaugacaugagagcgA..............................................    7     1     seq
.............ugacGuaaugacaugagagcgu..............................................    1     1     seq
.............ugacauaaAgacaugagagcgu..............................................    1     1     seq
.............ugacauaGugacaugagagcgu..............................................    1     1     seq
.............ugCcauaaugacaugagagcgu..............................................    1     1     seq
.............ugUcauaaugacaugagagcgu..............................................    1     1     seq
.............ugacauaaugaUaugagagcgu..............................................    1     1     seq
.............ugacauaaugacaugagagcgC..............................................    65    1     seq
.............ugacauaaugacaugagagUgu..............................................    1     1     seq
.............ugacauaaugacaugagagcAu..............................................    2     1     seq
.............ugacauaaugacaugGgagcgu..............................................    2     1     seq
.............Agacauaaugacaugagagcgu..............................................    6     1     seq
.............uAacauaaugacaugagagcgu..............................................    1     1     seq
.............ugacauaaugacaugagagcgu..............................................    359   0     seq
.............ugacauaaugacaugagagcCu..............................................    1     1     seq
.............ugacUuaaugacaugagagcgu..............................................    2     1     seq
.............ugacauaaCgacaugagagcgu..............................................    1     1     seq
.............Cgacauaaugacaugagagcgu..............................................    2     1     seq
.............ugacauaaugCcaugagagcgu..............................................    1     1     seq
.............ugGcauaaugacaugagagcgu..............................................    2     1     seq
.............ugacauaaugacaGgagagcgu..............................................    1     1     seq
.............ugacauaaugacauAagagcgu..............................................    2     1     seq
.............ugacauaaugaAaugagagcgu..............................................    1     1     seq
.............ugacauaaugGcaugagagcgu..............................................    3     1     seq
.............ugacauaaugacGugagagcgu..............................................    4     1     seq
.............ugacauaaugacaugagagcgG..............................................    14    1     seq
.............ugacauaaugacaugagagcguC.............................................    5     1     seq
.............ugacauaaugacaugagagcguU.............................................    28    1     seq
.............ugacauaaugacaugagagcguA.............................................    2     1     seq
.............ugacauaaugacaugagagcguAc............................................    2     1     seq
.............ugacauaaugacaugagagcguUc............................................    9     1     seq
...............acauaaugacaugagagcgu..............................................    1     0     seq
.........................................gccuauguuguuaugucguugu..................    1     0     seq
............................................uauguuguCaugucguuguguca..............    1     1     seq
```

miRBase precursor : novel-nve-miR-23
Total read count : 18943
novel-nve-miR-23_guide read count : 18938
novel-nve-miR-23_star read count : 5
remaining reads : 0

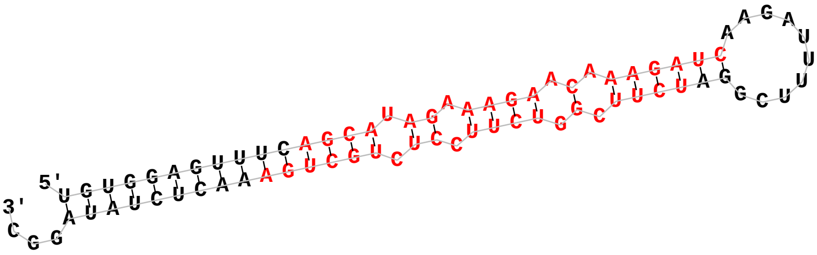

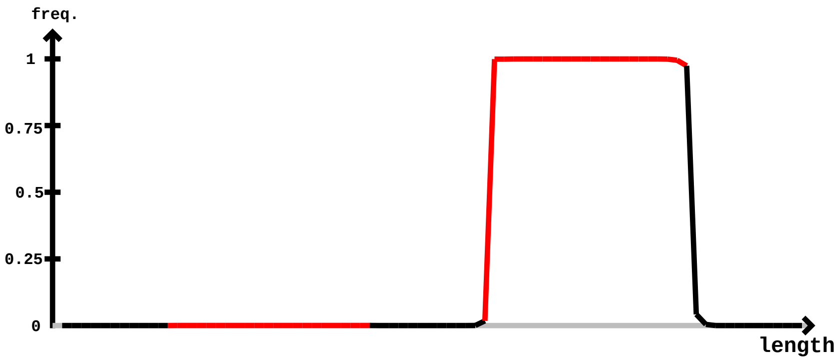


```
                    novel-nve-miR-23_star
                                               novel-nve-miR-23_guide
5'- uguggaguuucagcauagaaagaacaaagaucaagauuuucggaucuucggucuuccucugcugaaacucuauaggc   -3'    exp
    ((((((((((((((.((.((((.(.((((((............)))))).).)))).)).))))))))))))))...   reads      mm         sample
    ...........agcauagaaagaacaaagauU.............................................   1          1          seq
    ...........agcauagaaagaacaaagauc.............................................   4          0          seq
    ..........................................Aaucuucggucuuccucugcuga............   5          1          seq
    ...........................................aucuucggucuuccucug................   1          0          seq
    ...........................................aucuucggucuuccucugcA..............   1          1          seq
    ...........................................aucuucggucuuccucugcu..............   12         0          seq
    ...........................................aucuucggucuuccucugcC..............   4          1          seq
    ...........................................Uucuucggucuuccucugcu..............   1          1          seq
    ...........................................aucuucggucuuccucugcug.............   1          0          seq
    ...........................................Gucuucggucuuccucugcuga............   7          1          seq
    ...........................................aucuucggucuucUucugcuga............   1          1          seq
    ...........................................aucuucggucCuccucugcuga............   2          1          seq
    ...........................................aucuucggucuuccucugcugG............   6          1          seq
    ...........................................Cucuucggucuuccucugcuga............   1          1          seq
    ...........................................aucuucggucuuccucCgcuga............   2          1          seq
    ...........................................aucuucggucuCccucugcuga............   1          1          seq
    ...........................................aucuucggCcuuccucugcuga............   4          1          seq
    ...........................................aucuucAgucuuccucugcuga............   1          1          seq
    ...........................................aucuucggucuuccucugcuga............   258        0          seq
    ...........................................aucuCcggucuuccucugcuga............   1          1          seq
    ...........................................aucuucggucuuccucGgcuga............   2          1          seq
    ...........................................aucuucggucuuccucugcAga............   1          1          seq
    ...........................................aucCucggucuuccucugcuga............   2          1          seq
    ...........................................aucuucggucuuccGcugcuga............   1          1          seq
    ...........................................aucuucggucuucCCcugcuga............   2          1          seq
    ...........................................aucuucggucuuccucAgcuga............   2          1          seq
    ...........................................aucuucggucuuccAcugcuga............   1          1          seq
    ...........................................aAcuucggucuuccucugcuga............   2          1          seq
    ...........................................aucAucggucuuccucugcuga............   3          1          seq
    ...........................................aucuucggucuuccucugcugU............   10         1          seq
    ...........................................aucuucggucuuccucugcugaU...........   1          1          seq
    ............................................ucuucggucuuccucugc...............   3          0          seq
    ............................................ucuucggucuuccucugU...............   7          1          seq
    ............................................ucuucggucuuccucugA...............   1          1          seq
```

novel-nve-miR-23_star

novel-nve-miR-23_guide

```
uguggaguuucagcauagaaagaacaaagaucaagauuuucggaucuucggucuucccucugcugaaacucuauaggc

............................................ucuucggucuucccucugcG..............   1     1    seq
............................................ucuucggucuucccucCgcu..............   1     1    seq
............................................ucuucggucuucccucugcu..............   41    0    seq
............................................ucuucggucuucccucugcA..............   8     1    seq
............................................ucuucggucuucccucugcC..............   4     1    seq
............................................ucuuAggucuucccucugcu..............   1     1    seq
............................................ucuucggucuucccucugAu..............   5     1    seq
............................................ucuucgCucuucccucugcu..............   1     1    seq
............................................ucuCcggucuucccucugcug.............   3     1    seq
............................................ucuucggucuucccucugcuA.............   34    1    seq
............................................Ccuucggucuucccucugcug.............   3     1    seq
............................................ucuGcggucuucccucugcug.............   1     1    seq
............................................ucuucggAcuucccucugcug.............   1     1    seq
............................................ucuucggucuucccucugcug.............   319   0    seq
............................................ucuucggucuucAcucugcug.............   2     1    seq
............................................ucuucggucuAcccucugcug.............   2     1    seq
............................................ucuucggucuucccucugcuU.............   12    1    seq
............................................ucuucggCcuucccucugcug.............   3     1    seq
............................................ucuucggucuucccucugAug.............   1     1    seq
............................................ucuucggucuucUcucugcug.............   1     1    seq
............................................ucuucggucuCcccucugcug.............   1     1    seq
............................................ucuucggucuucccucugcCg.............   2     1    seq
............................................ucuucggucuucccAcugcug.............   1     1    seq
............................................Acuucggucuucccucugcug.............   2     1    seq
............................................ucuucggucCucccucugcug.............   1     1    seq
............................................ucAucggucuucccucugcug.............   2     1    seq
............................................ucuucggucuucccucugcuC.............   1     1    seq
............................................Gcuucggucuucccucugcug.............   1     1    seq
............................................ucCucggucuucccucugcuga............   72    1    seq
............................................ucuucggucuAcccucugcuga............   19    1    seq
............................................ucuuAggucuucccucugcuga............   6     1    seq
............................................ucuucggucuucccucugGuga............   6     1    seq
............................................ucuucggucuuccUucugcuga............   22    1    seq
............................................ucuucgUucuucccucugcuga............   6     1    seq
............................................ucuucggucuucccucugcugG............   186   1    seq
............................................ucuucgguAuucccucugcuga............   7     1    seq
............................................ucuucggucuucccucugcuCa............   6     1    seq
............................................ucuucgguUuucccucugcuga............   15    1    seq
............................................ucuucggucuucUcucugcuga............   20    1    seq
............................................ucuucAgucuucccucugcuga............   24    1    seq
............................................ucuucggucAucccucugcuga............   16    1    seq
............................................ucGucggucuucccucugcuga............   4     1    seq
............................................ucuucggCcuucccucugcuga............   60    1    seq
............................................uAuucggucuucccucugcuga............   12    1    seq
............................................ucuucggucuCcccucugcuga............   69    1    seq
............................................ucuuUggucuucccucugcuga............   28    1    seq
............................................ucuucgCucuucccucugcuga............   6     1    seq
............................................ucuucggucuucccucugAuga............   5     1    seq
............................................uUuucggucuucccucugcuga............   32    1    seq
............................................ucuucggucuucccuUugcuga............   4     1    seq
............................................ucuGcggucuucccucugcuga............   9     1    seq
............................................ucuucggucuucccucugcuUa............   4     1    seq
............................................ucuucggucuucccCcugcuga............   79    1    seq
............................................ucuucggucuucccucugcGga............   6     1    seq
............................................ucuucggucuGcccucugcuga............   1     1    seq
............................................Ccuucggucuucccucugcuga............   68    1    seq
............................................ucuucggucuucccucugcugC............   63    1    seq
............................................Gcuucggucuucccucugcuga............   26    1    seq
............................................ucuucgguGuucccucugcuga............   4     1    seq
............................................ucuucggucuucccuAugcuga............   5     1    seq
............................................ucuucgAucuucccucugcuga............   10    1    seq
............................................ucuucUgucuucccucugcuga............   9     1    seq
............................................ucuCcggucuucccucugcuga............   52    1    seq
............................................ucuucggucuucccucGgcuga............   8     1    seq
............................................Acuucggucuucccucugcuga............   133   1    seq
............................................ucuucggGcuucccucugcuga............   6     1    seq
............................................ucuucggucuucAcucugcuga............   11    1    seq
............................................ucuucggucuucccucugcuAa............   13    1    seq
............................................uGuucggucuucccucugcuga............   1     1    seq
```

```
           novel-nve-miR-23_star
                                            novel-nve-miR-23_guide
uguggaguuucagcauagaaagaacaaagaucaagauuuucggaucuucggucuuccucugcugaaacucuauaggc

............................................ucuucggucGuccucugcuga............	5	1	seq
............................................ucuuGggucuuccucugcuga............	2	1	seq
............................................ucuAcggucuuccucugcuga............	28	1	seq
............................................ucuucggAcuuccucugcuga............	19	1	seq
............................................ucuucggucuuccGcugcuga............	3	1	seq
............................................ucuucggucuuGcucugcuga............	3	1	seq
............................................ucuucggucuuccucugUuga............	50	1	seq
............................................ucuucggucuuccucugcAga............	9	1	seq
............................................ucuucggucuuccucCgcuga............	54	1	seq
............................................ucuucggucuuccucuUcuga............	6	1	seq
............................................ucuucCgucuuccucugcuga............	9	1	seq
............................................ucuucggucuucGucugcuga............	4	1	seq
............................................ucuucggucuuccAcugcuga............	16	1	seq
............................................ucAucggucuuccucugcuga............	67	1	seq
............................................ucuucggucuuccucuAcuga............	16	1	seq
............................................ucuucggucuuccucugcCga............	43	1	seq
............................................ucuucggucuuccucugcugU............	420	1	seq
............................................ucuucggucCuccucugcuga............	78	1	seq
............................................ucuucggucuuccucuCcuga............	5	1	seq
............................................ucuucggucuuccucugcuga............	15325	0	seq
............................................ucuucggucuuccucAgcuga............	23	1	seq
............................................ucuucggucuucAucugcuga............	9	1	seq
............................................ucuucggucuuccCcugcugaa...........	2	1	seq
............................................ucuucAgucuuccucugcugaa...........	1	1	seq
............................................ucuucggucuucUucugcugaa...........	2	1	seq
............................................uUuucggucuuccucugcugaa...........	1	1	seq
............................................ucuucggCcuuccucugcugaa...........	1	1	seq
............................................ucuucggucuuccucugUugaa...........	3	1	seq
............................................ucuAcggucuuccucugcugaa...........	1	1	seq
............................................ucuucggucuuccucugcugUa...........	4	1	seq
............................................ucuucggucuuccucugcugaC...........	14	1	seq
............................................ucuucggucuuccAcugcugaa...........	1	1	seq
............................................ucuucggucuuccucuAcugaa...........	1	1	seq
............................................ucuucggAcuuccucugcugaa...........	1	1	seq
............................................ucuucggucuuccucugcugaU...........	184	1	seq
............................................ucAucggucuuccucugcugaa...........	1	1	seq
............................................ucuucggucuuccucugcugaG...........	6	1	seq
............................................Acuucggucuuccucugcugaa...........	3	1	seq
............................................ucuucggucCuccucugcugaa...........	3	1	seq
............................................ucuuUggucuuccucugcugaa...........	1	1	seq
............................................ucuucggucuuccucugcugaa...........	478	0	seq
............................................ucuucgguUuuccucugcugaa...........	1	1	seq
............................................Ccuucggucuuccucugcugaa...........	3	1	seq
............................................ucuucggucuuccucCgcugaa...........	1	1	seq
............................................ucuucggucuuccucugcugaaa..........	3	0	seq
............................................ucuucggucuuccucugcugaaC..........	4	1	seq
............................................ucuucggucuuccucugcugaaG..........	1	1	seq
............................................ucuucggucuuccucugcugaUa..........	14	1	seq
............................................ucuucggucuuccucugcugaaU..........	59	1	seq
............................................ucuucggucuuccucugcugaUac.........	1	1	seq
.............................................cuucggucuuccucugcuga............	1	0	seq
..............................................uucggucuuccucugcuga............	8	0	seq
..............................................uucggucuuccucugcugaaU..........	2	1	seq
...............................................ucggucuuccucugcuga............	3	0	seq
```

miRBase precursor : novel-nve-miR-24
Total read count : 368
novel-nve-miR-24_guide read count : 295
novel-nve-miR-24_star read count : 54
remaining reads : 19

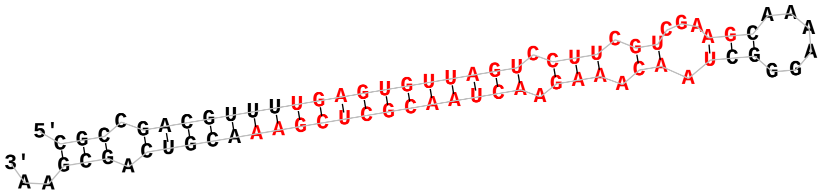

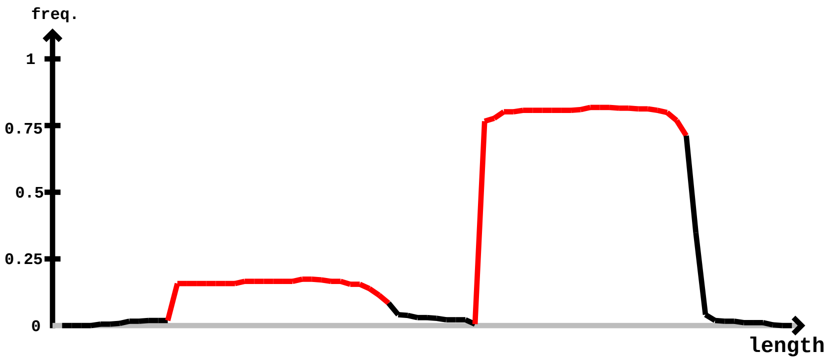

novel-nve-miR-24_star

novel-nve-miR-24_guide

```
5'- cgccgacguuuugaguguuaguccuucgucgaagcaaaagggcuaacaaagaacuaacgcucgaaacgucagcgaa   -3'    exp
    (((.((((((((((((((((((.(((.((...(((......))).)).))).)))))))))))))))))).)))..    reads    mm    sample
    ...cgacguuuugaguguuagucA....................................................    1    1    seq
    ...cgacguuuugaguguuagucU....................................................    1    1    seq
    .....acguuuugaguguuagucc....................................................    1    0    seq
    ......cguuuugaguguuagucUuu..................................................    1    1    seq
    ......cguuuugaguguuaguccuuU.................................................    1    1    seq
    ......cguuuugaguguuaguccuuc.................................................    1    0    seq
    ........uuuugaguguuaguccuucgu...............................................    1    0    seq
    ...........ugaguguuaguccuucgu...............................................    2    0    seq
    ...........ugagAguuaguccuucgu...............................................    1    1    seq
    ...........ugaguguuaguccuucgucg.............................................    3    0    seq
    ...........ugaguguuaguccuucgucA.............................................    2    1    seq
    ...........ugaguguuagucc Cucgucg............................................    1    1    seq
    ...........ugaguguuaguccuucgucga............................................    9    0    seq
    ...........ugaguguuaguccuucgucgaC...........................................    1    1    seq
    ...........ugaguguuaguccuucgucgaa...........................................    8    0    seq
    ...........ugaguguuaguccuucgucgaG...........................................    1    1    seq
    ...........ugaguguuaguccuucgucgaU...........................................    1    1    seq
    ...........ugaguguuaguccuucgucgaag..........................................    8    0    seq
    ...........ugaguguuaguccuucgucgaaA..........................................    1    1    seq
    ...........ugaguguuaguccuucgucgaaU..........................................    5    1    seq
    ...........ugagugCuaguccuucgucgaag..........................................    1    1    seq
    ...........ugaguguuaguccuucgucgaaC..........................................    1    1    seq
    ...........ugaguguuaguccuucgucgaagc.........................................    1    0    seq
    ...........ugaguguuaguccuucgucgaagU.........................................    1    1    seq
    ...........ugaguguuaguccuucgucgaagcU........................................    3    1    seq
    ...........ugaguguuaguccuucgucgaagca........................................    1    0    seq
    ..................uagCccuucgucgaagcaaa......................................    1    1    seq
    ..................uagCccuucgucgaagcaaaa.....................................    2    1    seq
    ........................uucgucgaagcaaaaggg..................................    5    0    seq
    ........................uucgucgaagcaaaaggA..................................    1    1    seq
    ...................................aaaagggcuaacaaagaacuaU...................    1    1    seq
    ....................................aaagggcuaacaaagaacuaacC.................    1    1    seq
    ...........................................uaacaaagaacuaacgcu...............    2    0    seq
    ...........................................uaacaaagaacuaacgcuc..............    2    0    seq
```

```
           novel-nve-miR-24_star
                                           novel-nve-miR-24_guide
cgccgacguuuugaguguuaguccuucgucgaagcaaaagggcuaacaaagaacuaacgcucgaaacgucagcgaa

...........................................uaacaaagaacuaacgcuU..............   1    1    seq
...........................................uaacaaagaacuaacgcucg.............   9    0    seq
...........................................uaacaaagaacuaacgcucA.............   1    1    seq
...........................................uaacaaagGacuaacgcucg.............   1    1    seq
...........................................uaacaaagaacuaacgcucgU............   1    1    seq
...........................................uaacaaagaacuaacgcucga............   18   0    seq
...........................................uaacaaagaacuaacgcucgaU...........   14   1    seq
...........................................uGacaaagaacuaacgcucgaa...........   1    1    seq
...........................................uaacaaagaacuaacgcucgGa...........   1    1    seq
...........................................uaacaaagaacuaacgcucgaG...........   5    1    seq
...........................................uaacaaagaacuaacgcucgaa...........   87   0    seq
...........................................uaacaaagaacuaacgcucgaC...........   5    1    seq
...........................................uaacaaagaacuaacgcucUaa...........   1    1    seq
...........................................uaacaaagaacuaGcgcucgaa...........   3    1    seq
...........................................uaacaaagaacCaacgcucgaa...........   1    1    seq
...........................................uaacGaagaacuaacgcucgaa...........   1    1    seq
...........................................uaacaGagaacuaacgcucgaa...........   1    1    seq
...........................................uaacaaagaacuGacgcucgaa...........   1    1    seq
...........................................Aaacaaagaacuaacgcucgaa...........   3    1    seq
...........................................uaacaaagaacuaacgcAcgaa...........   1    1    seq
...........................................uaacaaagaacuaacgcucgaaa..........   13   0    seq
...........................................uaacaaagaacuaacgcucgaaG..........   1    1    seq
...........................................uaacaaagaacuaacgcucgaaU..........   77   1    seq
...........................................uaacaaagaacuaacgcucgaaC..........   20   1    seq
...........................................uaacaaagaacuaacgcucgaaUc.........   2    1    seq
...........................................uaacaaagaacuaacgcucgaaac.........   6    0    seq
...........................................uaacaaagaacuaacgcucgaaacU........   1    1    seq
............................................aacaaagaacuaacgcucga............   1    0    seq
............................................aacaaagaacuaacgcucgaa...........   3    0    seq
.............................................acaaagaacuaacgcucga............   1    0    seq
.............................................acaaagaacuaacgcucgaa...........   6    0    seq
.............................................acaaagaacuaacgcucgaaU..........   2    1    seq
...............................................aaagaacuaacgcucgaaacguU......   1    1    seq
...............................................aaagaacuaacgcucgaaacguc......   1    0    seq
.....................................................cuaacgcucgaaacgucGgc...   1    1    seq
......................................................uaacgcucgaaacgucGgc...   2    1    seq
......................................................uaacgcucgaaacgucagcU..   1    1    seq
```

miRBase precursor : novel-nve-miR-25
Total read count : 79
novel-nve-miR-25_guide read count : 74
novel-nve-miR-25_star read count : 5
remaining reads : 0

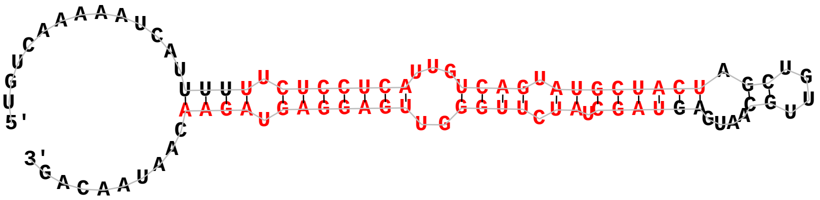

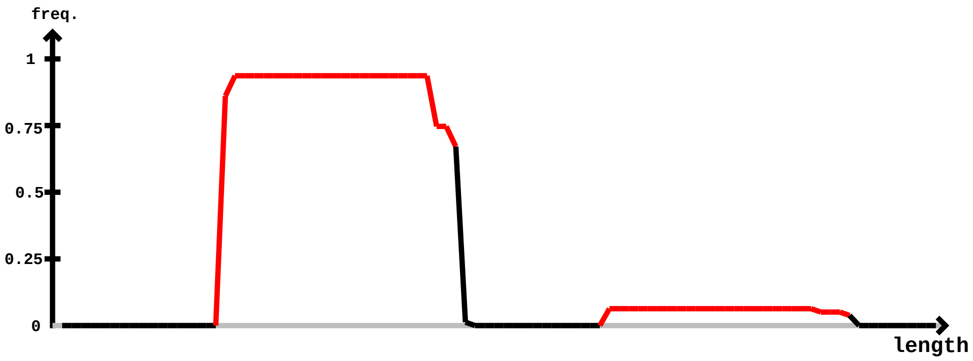


```
                                                   novel-nve-miR-25_star
                 novel-nve-miR-25_guide
5'- ugucaaaaaucauuuuuucuccucauugucaguaugcuacuagcuguugcaaugaguagcuaucuuggguugaggaguagaacaauaacag   -3'    exp
    .............((((.(((((((...((((.((((((((.((....))....)))))).)).))))..))))))).)))).........    reads    mm    sample
    ................uucuccucauugucaguaugcu.....................................................    8    0    seq
    ................uucuccucauugucaguaugAu.....................................................    1    1    seq
    ................uucuccucauugucaguaugcC.....................................................    4    1    seq
    ................Gucuccucauugucaguaugcu.....................................................    1    1    seq
    ................uucuccucauugucaguaugcuaU...................................................    1    1    seq
    ................uucuccucauugucaguaugcuac...................................................    5    0    seq
    ................uucuccGcauugucaguaugcuacu..................................................    1    1    seq
    ................uucuccucauugucaguaugcuacC..................................................    11    1    seq
    ................uCcuccucauugucaguaugcuacu..................................................    1    1    seq
    ................uucuccCcauugucaguaugcuacu..................................................    1    1    seq
    ................uucuccucauugucaguaugcuacu..................................................    33    0    seq
    ................uucuccucauugucaguaugcuacuU.................................................    1    1    seq
    .................ucuccucauugucaguaugcu.....................................................    1    0    seq
    .................ucuccucauugucaguaugcuacu..................................................    2    0    seq
    .................ucuccucauugucaguaugcuacC..................................................    1    1    seq
    .................uUuccucauugucaguaugcuacu..................................................    1    1    seq
    .................ucuccCcauugucaguaugcuacu..................................................    1    1    seq
    ........................................................uagcuaucuuggguugaggagu.............    1    0    seq
    ........................................................uagcuaucuuggguugaggaguaga..........    1    0    seq
    ........................................................uagcuaucuuggguugaggaguagaC.........    1    1    seq
    ........................................................uagcuaucuuggguugaggaguagaU.........    2    1    seq
```

miRBase precursor : novel-nve-miR-26
Total read count : 551
novel-nve-miR-26_guide read count : 449
novel-nve-miR-26_star read count : 92
remaining reads : 10

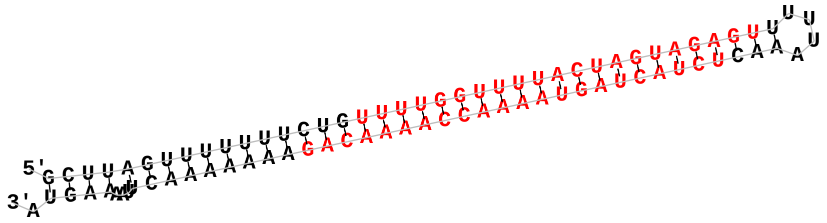

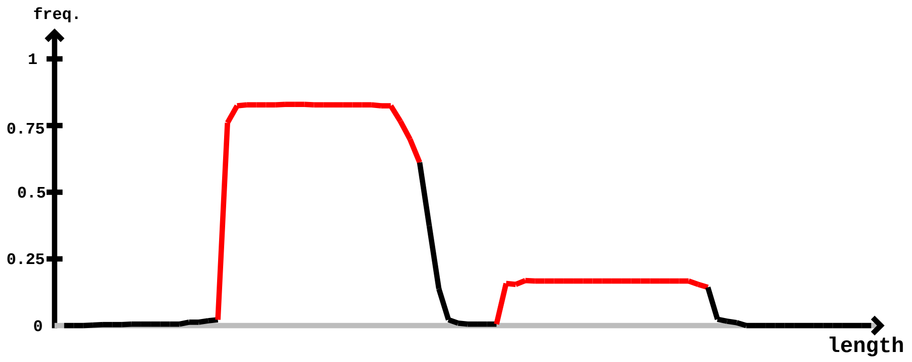

novel-nve-miR-26_star

novel-nve-miR-26_guide

```
5'- gcuuaguuuuuuucuguuuugguuuuacuaguagaguuuuuaaacucuacuaguaaaaccaaaacagaaaaaaacuuaaaagua   -3'    exp
    ((((((((((((((((((((((((((((((((((((((....))))))))))))))))))))))))))))))))))...)))).   reads   mm   sample
    ..uAaguuuuuuucuguuuugg..............................................................   1       1    seq
    ...uaUuuuuuuucuguuuugg..............................................................   1       1    seq
    ......uuuuuuucuguuuugguGu...........................................................   1       1    seq
    ............ucuguuuugguuuuacuagC....................................................   1       1    seq
    ............ucuguuuugguuuuacuagu....................................................   1       0    seq
    ............ucuguCuugguuuuacuaguaga.................................................   1       1    seq
    ............ucuguuuugguuuuacuaguagaU................................................   1       1    seq
    ..............uguuuugguuuuacuaguagag................................................   2       0    seq
    ..............uguuuugguuuuacuaguagagC...............................................   1       1    seq
    ...............guuuugguuuuacuaguag..................................................   1       0    seq
    ...............guuuugguuuuacuaguagag................................................   1       0    seq
    ................uuuugguuuuacuGguag..................................................   1       1    seq
    ................uuuugguuuuacuaguag..................................................   29      0    seq
    ................uuuugguuuuacuaguaA..................................................   1       1    seq
    ................uuuAgguuuuacuaguaga.................................................   1       1    seq
    ................uuuugguuuuaUuaguaga.................................................   1       1    seq
    ................Auuugguuuuacuaguaga.................................................   1       1    seq
    ................uuuugguuuuacuaguaga.................................................   29      0    seq
    ................Cuuugguuuuacuaguaga.................................................   1       1    seq
    ................uuuugguuuCacuaguaga.................................................   1       1    seq
    ................uuuugguuuuacAaguaga.................................................   1       1    seq
    ................uuuugguuuuacuaguagaA................................................   5       1    seq
    ................uuuugguuuuacuaguagaC................................................   1       1    seq
    ................uuuugguuuuacuaguagag................................................   35      0    seq
    ................uuCugguuuuacuaguagag................................................   1       1    seq
    ................uuuugguuuCacuaguagagu...............................................   1       1    seq
    ................uAuugguuuuacuaguagagu...............................................   2       1    seq
    ................Auuugguuuuacuaguagagu...............................................   1       1    seq
    ................uuuugguuuuacuaguagagA...............................................   1       1    seq
    ................Guuugguuuuacuaguagagu...............................................   1       1    seq
    ................uuuuggCuuuacuaguagagu...............................................   1       1    seq
    ................uuuugguuuuacuaguagagG...............................................   1       1    seq
    ................Cuuugguuuuacuaguagagu...............................................   1       1    seq
    ................uuuugguuuuacuaguagagu...............................................   87      0    seq
```

```
                                             novel-nve-miR-26_star
                novel-nve-miR-26_guide
gcuuaguuuuuuucuguuuugguuuuacuaguagaguuuuuaaacucuacuaguaaaaccaaaacagaaaaaaacuuaaaagua

................uuuugguuuuacuaguagagC...............................................    17    1    seq
................uuuugguuuuacuGguagaguu..............................................    1     1    seq
................Cuuugguuuuacuaguagaguu..............................................    1     1    seq
................uuuugguuuuacuaguagaguA..............................................    5     1    seq
................uuuugguuuuacuaguagaguC..............................................    22    1    seq
................uuuugguuuuacuaguaAaguu..............................................    1     1    seq
................uuuCgguuuuacuaguagaguu..............................................    1     1    seq
................uuuuCguuuuacuaguagaguu..............................................    1     1    seq
................uuuugguuuuacuaguagaguu..............................................    86    0    seq
................Auuugguuuuacuaguagaguu..............................................    1     1    seq
................uuuuggCuuuacuaguagaguu..............................................    1     1    seq
................uuuugguuuuacuaguagUguu..............................................    1     1    seq
................uuuugguuuuacuaguagaguuu.............................................    39    0    seq
................uuuugguuuuacuaguagaguuC.............................................    10    1    seq
................uuuugguuuuacuaguagaguuA.............................................    8     1    seq
................uuuugguuuuacuaguagaguuAu............................................    3     1    seq
................uuuugguuuuacuaguagaguuuA............................................    1     1    seq
................uuuugguuuuacuaguagaguuuu............................................    2     0    seq
................uuuugguuuuacuaguagaguuuAu...........................................    1     1    seq
................uuuugguuuuacuaguagaguuuuu...........................................    1     0    seq
.................uuugguuuuacuaguaga.................................................    1     0    seq
.................uuugguuuuacuaguagag................................................    2     0    seq
.................uuugguuuuacuaguagagA...............................................    1     1    seq
.................uuugguuuuacuaguagagC...............................................    1     1    seq
.................uuugguuuuacuaguagagu...............................................    15    0    seq
.................uuugguuuuacuaguagaguu..............................................    6     0    seq
.................uuugguuuuacuaguagaguA..............................................    3     1    seq
.................uuugguuuuacuaguagaguuu.............................................    4     0    seq
.................uuugguuuuacuaguagaguuC.............................................    1     1    seq
.................uuugguuuuacuaguagaguuuA............................................    1     1    seq
..................uugguuuuacuaguagaguu..............................................    1     0    seq
..................uugguuuuacuaguagaguuA.............................................    1     1    seq
......................uuuuacuaguagaguuuuuaaacC......................................    1     1    seq
......................uuuuacuaguagaguuuuuaaacu......................................    1     0    seq
......................uuuuacuaguagaguuuuuaaacucu....................................    1     0    seq
.............................................ucuacuaguaaaaccaaaac...................    7     0    seq
.............................................ucuacuaguaaaaccaaaaca..................    6     0    seq
.............................................ucuacuaguaaaaccaaaacag.................    47    0    seq
.............................................ucuacuaguaaaaccaaaacaA.................    9     1    seq
.............................................ucuacuaguaaaGccaaaacag.................    1     1    seq
.............................................uUuacuaguaaaaccaaaacag.................    1     1    seq
.............................................ucuacuaguaaaaccaGaacag.................    1     1    seq
.............................................ucuacuaguaaaaccaaaacaC.................    1     1    seq
.............................................ucuacuaguaaaaccaaaGcag.................    2     1    seq
.............................................ucuacAaguaaaaccaaaacag.................    1     1    seq
.............................................ucuacuaguaGaaccaaaacag.................    1     1    seq
.............................................uGuacuaguaaaaccaaaacag.................    1     1    seq
.............................................ucuacuaguaaaaccaaaacaU.................    1     1    seq
.............................................ucuacuaguaaaaccaaaacagU................    1     1    seq
.............................................ucuacuaguaaaaccaaaacagaU...............    2     1    seq
.............................................ucuacuaguaaaaccaaaacagaaU..............    2     1    seq
...............................................uacuaguaaaaccaaaacaga................    2     0    seq
...............................................uacuaguaaaaccaaaacagG................    1     1    seq
...............................................uacuaguaaaaccaaaacagaa...............    1     0    seq
...............................................uacuaguaaaaccaaaacagaaC..............    2     1    seq
...............................................uacuaguaaaaccaaaacagaaU..............    2     1    seq
```

miRBase precursor : novel-nve-miR-28
Total read count : 422
novel-nve-miR-28_guide read count : 421
novel-nve-miR-28_star read count : 1
remaining reads : 0

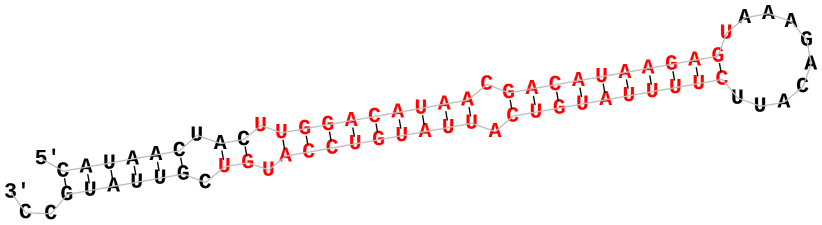

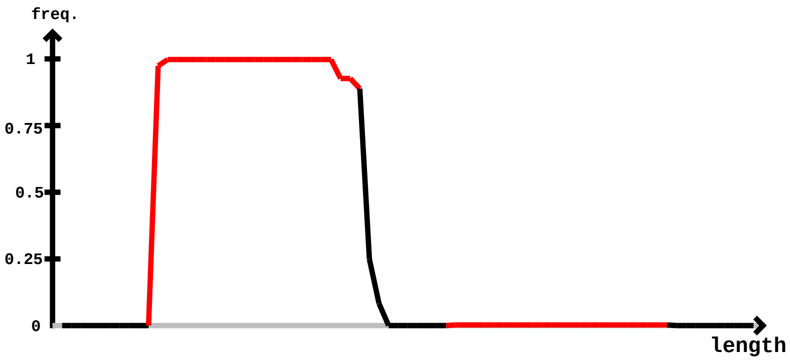


```
           novel-nve-miR-28_guide
                                       novel-nve-miR-28_star
5'- cauaacuacuuggacauaacgacauaagaguaaagacauucuuuuaugucauuauguccaugucguuaugcc  -3'   exp
    ((((((.((.(((((((((.(((((((((.........)))))))))).))))))))).)).))))))..   reads    mm    sample
    .........uuggUcauaacgacauaag...........................................  1        1     seq
    .........uuggGcauaacgacauaag...........................................  1        1     seq
    .........uuggacauaacgacauaaA...........................................  1        1     seq
    .........uuggacauaacgacauaag...........................................  23       0     seq
    .........uuggacauaacgacauaagaA.........................................  2        1     seq
    .........uuggacauaacgacauaagag.........................................  10       0     seq
    .........Auggacauaacgacauaagag.........................................  1        1     seq
    .........uuggacauaGcgacauaagagu........................................  1        1     seq
    .........uuggacauaacgacauaagagA........................................  3        1     seq
    .........uuggacauaacgacauaagagG........................................  3        1     seq
    .........Guggacauaacgacauaagagu........................................  1        1     seq
    .........uuggacauaacgacaCaagagu........................................  1        1     seq
    .........uCggacauaacgacauaagagu........................................  3        1     seq
    .........uuggacauaacgacauaagagu........................................  218      0     seq
    .........Auggacauaacgacauaagagu........................................  2        1     seq
    .........uuggacauaacgacauaagagC........................................  27       1     seq
    .........uuggacauaacgacauaUgagu........................................  1        1     seq
    .........uuggacauaacgacauaGgagu........................................  1        1     seq
    .........uAggacauaacgacauaagagu........................................  1        1     seq
    .........uuggacauaacgacUuaagagu........................................  1        1     seq
    .........uuggacauaacgacGuaagagu........................................  2        1     seq
    .........uuggacauaacgacauGagagu........................................  1        1     seq
    .........uuggacauaacgaUauaagagu........................................  1        1     seq
    .........uuggaAauaacgacauaagagu........................................  2        1     seq
    .........uuggacauaacgGcauaagagua.......................................  1        1     seq
    .........uuggacauaacgacauaagagua.......................................  54       0     seq
    .........uuggacauaacgacauaagaguU.......................................  7        1     seq
    .........uuggacauaacgacauaagaguG.......................................  1        1     seq
    .........uuggacauaacgacauaagaguC.......................................  4        1     seq
    .........uuCgacauaacgacauaagagua.......................................  1        1     seq
    .........uuggacauaacgacauaagaguaC......................................  4        1     seq
    .........uuggacauaacgacauaagaguaU......................................  31       1     seq
    ..........uggacauaacgacauaag...........................................  3        0     seq
    ..........uggacaGaacgacauaag...........................................  1        1     seq
```

```
         novel-nve-miR-28_guide
                                          novel-nve-miR-28_star
cauaacuacuuggacauaacgacauaaagaguaaagacauucuuuuaugucauuauguccaugucguuaugcc

..........uggacaGaacgacauaagag..........................................    3          1          seq
..........uggacauaacgacauaagagA.........................................    1          1          seq
..........uggacauaacgacauaagagGa........................................    2          1          seq
........................................Uuuuuaugucauuauguccaugu.........    1          1          seq
```

miRBase precursor : novel-nve-miR-29
Total read count : 108
novel-nve-miR-29_guide read count : 81
novel-nve-miR-29_star read count : 26
remaining reads : 1

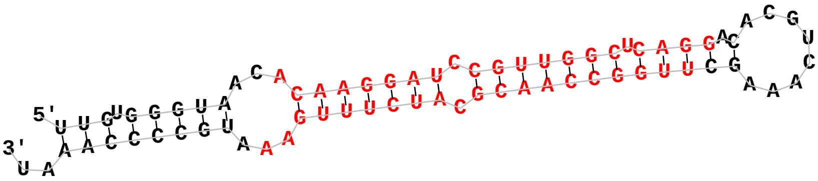

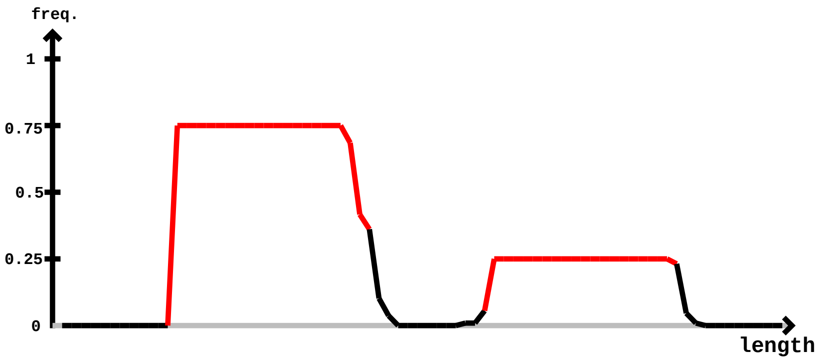


```
                          novel-nve-miR-29_guide
                                                          novel-nve-miR-29_star
5'- uuguggguaacacaaggauccguuggcucaggacacgucaaagcuuggccaacgcaucuuugaaaugccccaaau  -3'    exp
    (((.(((((...(((((((.(((((((.((((.(........))))))))))))).)))))))...))))))))..    reads    mm    sample
    ...........acaaggauccguuggcuG..............................................    4        1     seq
    ...........acaaggauccguuggcuc..............................................    2        0     seq
    ...........acaaggauccguuggcuU..............................................    1        1     seq
    ...........acaaggauccguuggcucG.............................................    16       1     seq
    ...........acaaggGuccguuggcuca.............................................    1        1     seq
    ...........acaaggauccguuggcuca.............................................    12       0     seq
    ...........acaaggauccguuggcucag............................................    6        0     seq
    ...........acaaggauccguuggcucagU...........................................    1        1     seq
    ...........Gcaaggauccguuggcucagg...........................................    1        1     seq
    ...........acaaggauccguuggcucagA...........................................    6        1     seq
    ...........acaaggauccguuggcAcagg...........................................    1        1     seq
    ...........acaaggauccguuggcucagg...........................................    16       0     seq
    ...........acaaggauccguuggcucaCg...........................................    3        1     seq
    ...........acaaggauccguuggcucaggC..........................................    1        1     seq
    ...........acaaggauccguuggcucaggU..........................................    1        1     seq
    ...........acaaggauccguuggcucagga..........................................    5        0     seq
    ...........acaaggauccguuggcucaggaU.........................................    4        1     seq
    .........................................agcuuggccaacgcaucuuugaC...........    1        1     seq
    ...........................................cuuggccaacgcaucuuugaa...........    1        0     seq
    ...........................................cuuggccaacgcaucuuugaG...........    2        1     seq
    ...........................................cuuggccaacgcaucuuugaU...........    1        1     seq
    ...........................................cuuggccaacgcaucuuugaaC..........    1        1     seq
    ............................................uuggccaacgcaucuuuga............    2        0     seq
    ............................................uuggccaacgcaucuuugaC...........    1        1     seq
    ............................................uuggccaacgcaucuuugaG...........    1        1     seq
    ............................................uuggccaacgcaucuuugaa...........    10       0     seq
    ............................................uuggccaacgcaucuuugaU...........    3        1     seq
    ............................................uuggccaacgcaucuuugaaa..........    2        0     seq
    ............................................uuggccaacgcaucuuugaUa..........    1        1     seq
    ............................................uuggccaacgcaucuuugaUau.........    1        1     seq
```

miRBase precursor : novel-nve-miR-30
Total read count : 82
novel-nve-miR-30_guide read count : 73
novel-nve-miR-30_star read count : 8
remaining reads : 1

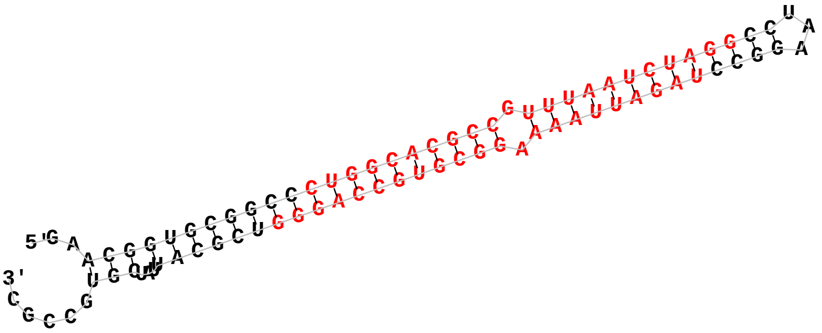

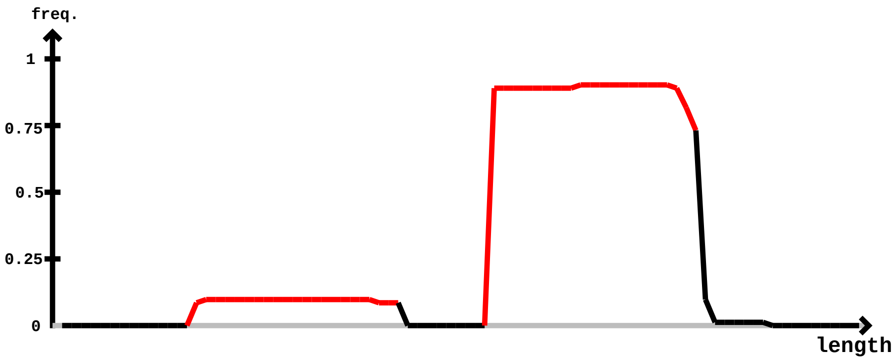


```
                                               novel-nve-miR-30_guide
                  novel-nve-miR-30_star
5'- gaacggugcggcccuggcacgccguuuaaucuaggccuaaggccuagauuaaaaggcgugccagggucgcauuaucgugccgc  -3'    exp
    ..((((((((((((((((((((.((((((((((((...))))))))))))).)))))))))))))))))...))).....    reads      mm         sample
    .............cuggcacgccguuuaaucu...................................................    1          0          seq
    .............cuggcacgccguuuaaucuagC................................................    1          1          seq
    .............cuggcacgccguuuaaucuagU................................................    1          1          seq
    .............cuggcacgccguuuaaucuagg................................................    4          0          seq
    ..............uggcacgccguuuaaucuagA................................................    1          1          seq
    ............................................uagauuaaaaggcgugcca....................    1          0          seq
    ............................................uagauuaaaaggcgCgccag...................    1          1          seq
    ............................................uagauuaaaaggcgugccaA...................    1          1          seq
    ............................................uagauuaaaaggcgugccaU...................    1          1          seq
    ............................................uagauuaaaaggcgugccag...................    3          0          seq
    ............................................uagauuaaaaggcgugccagA..................    2          1          seq
    ............................................uagauuaaaaggcgugccagg..................    5          0          seq
    ............................................uagauuaaaaggcgugccaggA.................    8          1          seq
    ............................................uagauuaaaaggcgugccaggC.................    2          1          seq
    ............................................uagauuaaaaggUgugccaggg.................    1          1          seq
    ............................................uagauuaaaaggcgugccaggU.................    2          1          seq
    ............................................uagauuaaaaggcgugccaggg.................    36         0          seq
    ............................................uagauuaaaagCcgugccaggg.................    1          1          seq
    ............................................Gagauuaaaaggcgugccaggg.................    2          1          seq
    ............................................uagauuaaaaggcgugccagggA................    3          1          seq
    ............................................uagauuaaaaggcgugccagggu................    3          0          seq
    ............................................uagauuaaaaggcgugccagggC................    1          1          seq
    .....................................................Cggcgugccagggucgcauu..........    1          1          seq
```

miRBase precursor : novel-nve-miR-32
Total read count : 611
novel-nve-miR-32_guide read count : 595
novel-nve-miR-32_star read count : 16
remaining reads : 0

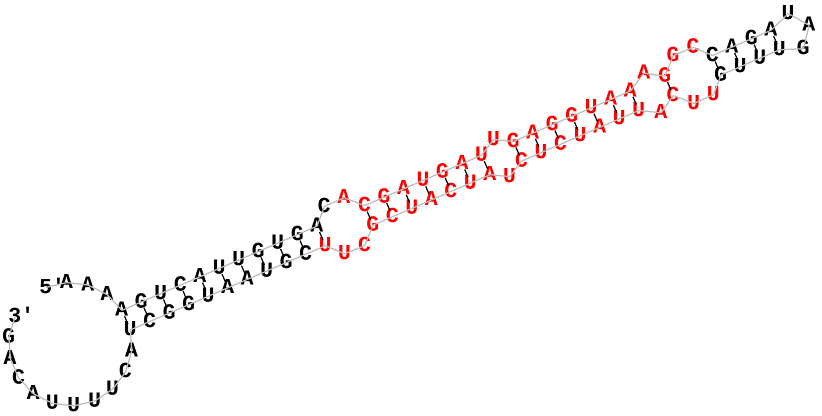

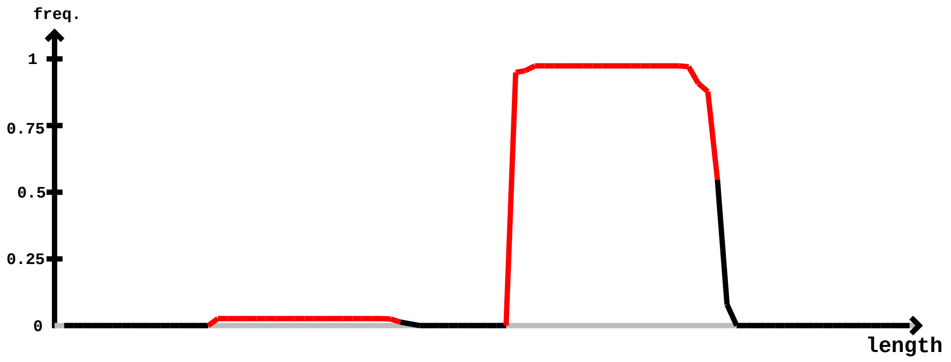


```
                                                novel-nve-miR-32_guide
                    novel-nve-miR-32_star
5'- aaaagucauugugacacgaugauugagguaaaggccagauaguuuguucauuaucucuaucaucgcuucguaauggcuacuuuuacag  -3'    exp
    ...((((((((((..(((((((.(((((((.(..((((...))))..).))))))).)))))))..))))))))))............    reads      mm        sample
    ...............acgaugauugagguaaag.......................................................    1          0         seq
    ...............acgaugauugagguaaagg......................................................    6          0         seq
    ...............acgaugauugagguaaagA......................................................    1          1         seq
    ...............acgaugauugagguaaaggc.....................................................    3          0         seq
    ...............acgaugauugagguaaaggU.....................................................    1          1         seq
    ...............acgaugauugagguaaaggcU....................................................    2          1         seq
    ...............acgaugauugagguaaaggAc....................................................    1          1         seq
    ...............acgaugauugagguaaaggcc....................................................    1          0         seq
    .............................................uucauuaucucuaucauc.........................    2          0         seq
    .............................................uucauuaucucuaucaucA........................    5          1         seq
    .............................................uAcauuaucucuaucaucg........................    1          1         seq
    .............................................uucauuaucucuaucaucU........................    3          1         seq
    .............................................uucauuaucucuaucaucg........................    26         0         seq
    .............................................Aucauuaucucuaucaucg........................    1          1         seq
    .............................................uucauuaucucuUucaucg........................    1          1         seq
    .............................................uucauuaucucuaucauAg........................    1          1         seq
    .............................................uucauuaucucuaucaucgc.......................    7          0         seq
    .............................................uucauuaucucuaucaucgA.......................    1          1         seq
    .............................................uucauuaucucuaucaucgU.......................    8          1         seq
    .............................................uucauuaucucuaucaucgG.......................    1          1         seq
    .............................................uucauuaucucuaCcaucgc.......................    1          1         seq
    .............................................uucauuaucCcuaucaucgc.......................    1          1         seq
    .............................................uucauuaucuAuaucaucgcu......................    1          1         seq
    .............................................uucauuaAcucuaucaucgcu......................    1          1         seq
    .............................................uucauuaucucuaucaucgcu......................    134        0         seq
    .............................................uucauuaucucuaucaucUcu......................    1          1         seq
    .............................................uucauuaucucuaucGucgcu......................    1          1         seq
    .............................................uuUauuaucucuaucaucgcu......................    1          1         seq
    .............................................uucauuaucucuaucaCcgcu......................    2          1         seq
    .............................................uucauuaucucuaucaucgUu......................    1          1         seq
    .............................................uCcauuaucucuaucaucgcu......................    1          1         seq
    .............................................uucaAuaucucuaucaucgcu......................    1          1         seq
    .............................................uucauuaucucuaucaucgcC......................    32         1         seq
    .............................................uucauuaucucuauAaucgcu......................    1          1         seq
```

```
                                              novel-nve-miR-32_guide
               novel-nve-miR-32_star
aaaagucauugugacacgaugauugagguaaaggccagauaguuuguucauuaucucuaucaucgcuucguaauggcuacuuuuacag

..............................................Gucauuaucucuaucaucgcu.....................    1      1      seq
..............................................uucauuaucucuaucaucgcG.....................    4      1      seq
..............................................uucauuaucucuUucaucgcu.....................    1      1      seq
..............................................uucUuuaucucuaucaucgcu.....................    1      1      seq
..............................................uucauuaCcucuaucaucgcu.....................    1      1      seq
..............................................uuAauuaucucuaucaucgcu.....................    1      1      seq
..............................................uucauuaucucuaucaucgcA.....................    12     1      seq
..............................................uucauuUucucuaucaucgcuu....................    1      1      seq
..............................................uucauuaucucuaAcaucgcuu....................    1      1      seq
..............................................uucauuaucucuaucaucgcuA....................    9      1      seq
..............................................uucauuaCcucuaucaucgcuu....................    1      1      seq
..............................................uucauuaucucuaucaucgcGu....................    1      1      seq
..............................................Aucauuaucucuaucaucgcuu....................    1      1      seq
..............................................uucauuaucGcuaucaucgcuu....................    1      1      seq
..............................................uucauuauAucuaucaucgcuu....................    1      1      seq
..............................................uucauuaucucuaucaucgcuC....................    52     1      seq
..............................................uucauuaucucuaucaucgcuG....................    9      1      seq
..............................................uucauAaucucuaucaucgcuu....................    1      1      seq
..............................................uAcauuaucucuaucaucgcuu....................    2      1      seq
..............................................uucauuaucCcuaucaucgcuu....................    1      1      seq
..............................................uucauuaucucCaucaucgcuu....................    1      1      seq
..............................................uucauuaucucuaucaCcgcuu....................    1      1      seq
..............................................uucauuaucucuaucaucgUuu....................    1      1      seq
..............................................uucauuaucucuGucaucgcuu....................    1      1      seq
..............................................uucauuaucucuaucaucgcuu....................    190    0      seq
..............................................uucauuaucucuaucaucgcAu....................    1      1      seq
..............................................uucauuaucucuaucaucgcuuG...................    1      1      seq
..............................................uucauuaucucuaucaucgcuuA...................    2      1      seq
..............................................uucauuaucucuaucaucgcuuc...................    8      0      seq
..............................................uucauuaucucuaucaucgcuuU...................    36     1      seq
...............................................ucauuaucucuaucaucgcu.....................    2      0      seq
...............................................ucauuaucucuaucaucgcuC....................    1      1      seq
...............................................ucauuaucucuaucaucgcuu....................    1      0      seq
................................................cauuaucucuaucaucCcu.....................    1      1      seq
................................................cauuaucucuaucaucgcu.....................    1      0      seq
................................................cauuaucucuaucaucgcuC....................    2      1      seq
................................................cauuaucucuaucaucgcuu....................    6      0      seq
................................................cauuaucucuaucaucgcuAc...................    1      1      seq
```

miRBase precursor : novel-nve-miR-33
Total read count : 376
novel-nve-miR-33_guide read count : 361
novel-nve-miR-33_star read count : 1
remaining reads : 14

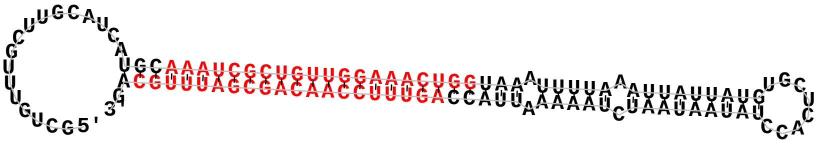

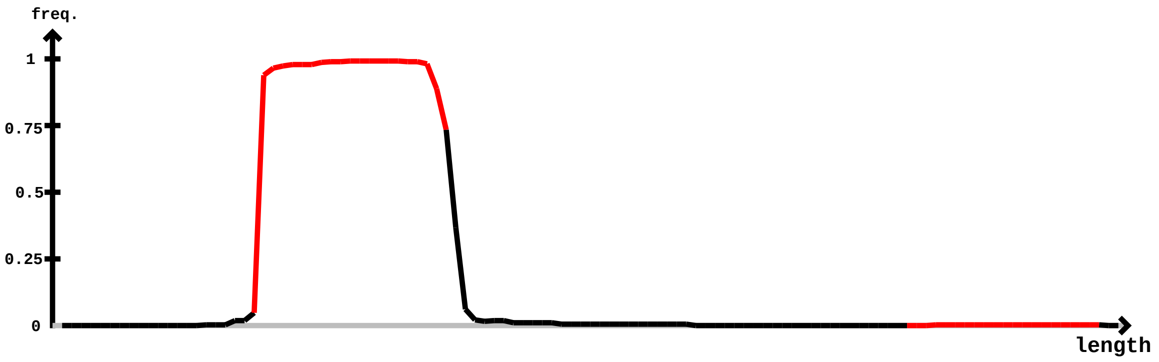


```
                                   novel-nve-miR-33_guide
                                                                                    novel-nve-miR-33_star
5'- gcuguuugcuugcaucaugcaaaucgcuguuggaaacugguaaauuuuaaauuauuaugugcucaccuauaauaaucuaaaaaauuaccaguuuccaacagcgauuugcag  -3'    exp
    ((.....))........((((((((((((((((((((((((((.(((((.(((((((((........))))))))).))))).))))))))))))))))))))))))))).   reads   mm   sample
    ..............ucGugcaaaucgcuguuggaa............................................................................   1       1    seq
    .................ugcaaaucgcuguuggaaac..........................................................................   1       0    seq
    .................ugcaaaucgcugAuggaaac..........................................................................   1       1    seq
    .................Cgcaaaucgcuguuggaaac..........................................................................   1       1    seq
    .................ugcaaaucgcuguuggaaacA.........................................................................   1       1    seq
    .................ugcaaaucgcuguuggaaacu.........................................................................   2       0    seq
    ...................caaaucgcuguuggaaacug........................................................................   1       0    seq
    ...................caaaucgcuguuggaaacugg.......................................................................   1       0    seq
    ...................cGaaucgcuguuggaaacugg.......................................................................   2       1    seq
    ...................caaaucgcuguuggaaacugA.......................................................................   1       1    seq
    ...................caaaucgcuguuggaaacuggA......................................................................   2       1    seq
    ...................caaaucgcuguuggaaacuggG......................................................................   1       1    seq
    ...................caaaucgcuguuggaaacuggu......................................................................   1       0    seq
    ...................caaaucgcuguuggaaacugguaC....................................................................   2       1    seq
    ....................aaaucgcugCuggaaacu.........................................................................   1       1    seq
    ....................aaaucgcuguuggaaacA.........................................................................   1       1    seq
    ....................aaaucgcuguuggaaacC.........................................................................   7       1    seq
    ....................aaaucgcCguuggaaacu.........................................................................   1       1    seq
    ....................aaaucgcuguuggaaacu.........................................................................   11      0    seq
    ....................aaaucgcuguuggaGacu.........................................................................   1       1    seq
    ....................aGaucgcuguuggaaacu.........................................................................   10      1    seq
    ....................aaaucgcuguuggaaacug........................................................................   33      0    seq
    ....................aGaucgcuguuggaaacug........................................................................   10      1    seq
    ....................aaaucgcuguuggaGacug........................................................................   1       1    seq
    ....................aaaucgcuguuggaaacuC........................................................................   1       1    seq
    ....................aaaucgcuguuggaaacuA........................................................................   9       1    seq
    ....................aaaucgcugCuggaaacug........................................................................   1       1    seq
    ....................aaaucgcCguuggaaacug........................................................................   1       1    seq
    ....................Gaaucgcuguuggaaacug........................................................................   1       1    seq
    ....................aaaucgcuguuggaaacugA.......................................................................   21      1    seq
    ....................Uaaucgcuguuggaaacugg.......................................................................   1       1    seq
    ....................aaaucgcugCuggaaacugg.......................................................................   1       1    seq
    ....................aaGucgcuguuggaaacugg.......................................................................   1       1    seq
    ....................aaaucgcuguuggaGacugg.......................................................................   6       1    seq
```

novel-nve-miR-33_guide

novel-nve-miR-33_star

gcuguuugcuugcaucaugcaaaucgcuguuggaaacugguaaauuuuaaauuauuaugugcucaccuauaauaaucuaaaaauuaccaguuuccaacagcgauuugcag

....................aaaucAcuguuggaaacugg..	1	1	seq
....................aaauAgcuguuggaaacugg..	1	1	seq
....................aaaucgcuguuggaaaUugg..	1	1	seq
....................aaaucgcuguugCaaacugg..	1	1	seq
....................aaaucgcuguuggaaacugg..	68	0	seq
....................Gaaucgcuguuggaaacugg..	3	1	seq
....................aaaucgcuguuggaaacugU..	1	1	seq
....................aGaucgcuguuggaaacugg..	19	1	seq
....................aaauUgcuguuggaaacugg..	1	1	seq
....................aGaucgcuguuggaaacuggu...	4	1	seq
....................aaaucgcuguuggaGacuggu...	3	1	seq
....................aaaucgcuguuggaaacuggu...	34	0	seq
....................aaaucgcuguuggaaacuggG...	2	1	seq
....................aaaucgcugCuggaaacuggu...	1	1	seq
....................aaaCcgcuguuggaaacuggu...	1	1	seq
....................aaaucgcuguuggaaaUuggu...	1	1	seq
....................aaaucCcuguuggaaacuggu...	1	1	seq
....................aaaucgcuguuggaaacuggC...	6	1	seq
....................aaaucAcuguuggaaacuggu...	1	1	seq
....................aaaucgcCguuggaaacuggu...	2	1	seq
....................Gaaucgcuguuggaaacuggu...	1	1	seq
....................aaaucgcuguuggaaacuggA...	48	1	seq
....................aaauUgcuguuggaaacuggu...	3	1	seq
....................Gaaucgcuguuggaaacuggua..	1	1	seq
....................aaaucgcuguuggaGacuggua..	1	1	seq
....................aGaucgcuguuggaaacuggua..	4	1	seq
....................aaaucgcuguuggaaacugAua..	1	1	seq
....................aaaucgcuguuggaaacugguC..	3	1	seq
....................aaaucgcuguuggaaacuggua..	2	0	seq
.....................aaucgcuguuggaaacugA..	2	1	seq
.....................aaucgcuguuggaaacAgg..	1	1	seq
.....................aaucgcuguuggaaacugg..	1	0	seq
.....................Caucgcuguuggaaacugg..	1	1	seq
.....................aaucgcuguuggaaacugU..	2	1	seq
.....................aaucgcuguuggaaacuggu...	1	0	seq
.....................aaucgcuguuggaaacuggA...	2	1	seq
......................aucgcuguuggaGacugg..	1	1	seq
......................aucgcuguuggaaacuggA...	1	1	seq
......................aucgcuguuggaaacuggAa..	1	1	seq
.......................ucgcuguuggaaacuggua..	2	0	seq
..........................cuguuggaaacugguaaaauu...	2	0	seq
..........................cuguuggaaacugguaaaauC...	1	1	seq
...........................uguuggaaacugguaaaauuuuaUa..	1	1	seq
.............................uuggaaacugguaaaauuuuaUa..	1	1	seq
....Uaauuuuaaauuauuaugugcuca...	1	1	seq
....uuuuaaauuauuaugugcucU...	1	1	seq
....uuuccaaUagcgauuugc..	1	1	seq

miRBase precursor : novel-nve-miR-37
Total read count : 2843
novel-nve-miR-37_guide read count : 2819
novel-nve-miR-37_star read count : 2
remaining reads : 22

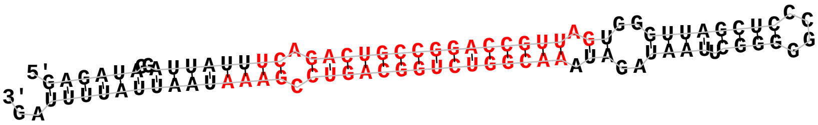

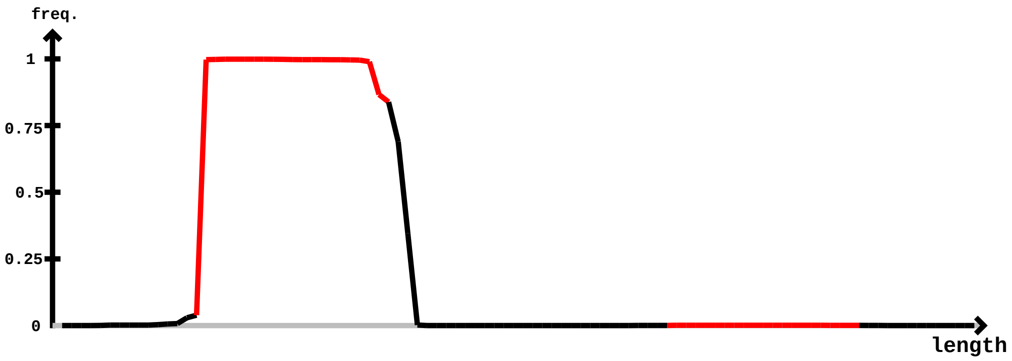

novel-nve-miR-37_star

novel-nve-miR-37_guide

5'- gagauaggauuauuucagacugccggaccguuaguggguuagcucccgggggcuuaauagauaaacggucuggcaguccgaaauaauuauuuuag -3'	exp		
((((((..((((((((.(((((((((((((((.((..((((((((....)))).))))..)).))))))))))))))).))))))))))))))..	reads	mm	sample
...auaggauuauuucGgacu..	1	1	seq
....uaggauuauuucagacug...	1	0	seq
....uaggauuauuucagacugc..	2	0	seq
....uaggauuauuucagacugcc...	1	0	seq
.........uuauuucGgacugccgga..	1	1	seq
.........uuauuucGgacugccggacc..	2	1	seq
.........uuauuucagacugccggaccguua..	1	0	seq
.........uuauuucagacugccggaccguuU..	1	1	seq
..........uauuucagacugccggaccgu..	5	0	seq
..........uauuucagacugccggaccguuU..	1	1	seq
..........uauuucagacugccggaccguuaU...	1	1	seq
...........auucGgacugccggaccgu...	1	1	seq
...........auucagacugccggaccgu...	1	0	seq
...........auucagacugccggaccguu..	2	0	seq
............uucGgacugccggaccg..	2	1	seq
............uucagacugccggaccgu...	3	0	seq
............uucGgacugccggaccgu...	5	1	seq
............uucagacugccggaccgC...	1	1	seq
............Aucagacugccggaccgu...	1	1	seq
............uucagacugccggaccguC..	3	1	seq
............uucagacugcUggaccguu..	1	1	seq
............uucagacugccggaccguu..	1	0	seq
............uucGgacugccggaccguu..	11	1	seq
............uucagacugUcggaccguua...	1	1	seq
............uucGgacugccggaccguua...	14	1	seq
............uucagacugccggaccguuC...	1	1	seq
............uucagacugccggaccguua...	5	0	seq
............uucagacugccggaccguuU...	4	1	seq
............uucagacugccggaccguuaA..	1	1	seq
............uucagacugccggaccguuaU..	2	1	seq
............uucagacugccggaccguuag..	6	0	seq
.............ucagacugccggaccguC..	1	1	seq
.............ucGgacugccggaccguu..	4	1	seq
.............ucGgacugccggaccguua...	2	1	seq

```
                                                               novel-nve-miR-37_star
              novel-nve-miR-37_guide
gagauaggauuauuucagacugccggaccguuaguggguuagcucccgggggcuuaauagauaaacggucuggcaguccgaaauaauuauuuuag

.............uucagacugccggaccguuU..............................................................    1     1     seq
.............uucagacugccggaccguuag.............................................................    6     0     seq
.............uucagacugccggaccguuaC.............................................................    1     1     seq
.............uAcagacugccggaccguuagu............................................................    1     1     seq
.............uucagacugccggaccguuagu............................................................    10    0     seq
.............uucagacugccggaccguuagC............................................................    1     1     seq
.............uucagacugccggaccguuaguA...........................................................    1     1     seq
..............ucagacugccggaccAuu...............................................................    1     1     seq
..............ucagGcugccggaccguu...............................................................    2     1     seq
..............ucagacugccggacUguu...............................................................    1     1     seq
..............ucagacuAccggaccguu...............................................................    1     1     seq
..............ucagacugcAggaccguu...............................................................    1     1     seq
..............ucUgacugccggaccguu...............................................................    2     1     seq
..............uAagacugccggaccguu...............................................................    1     1     seq
..............ucagacugccggaccguC...............................................................    53    1     seq
..............ucagacugccggUccguu...............................................................    1     1     seq
..............Acagacugccggaccguu...............................................................    2     1     seq
..............ucagacugccggaccguu...............................................................    244   0     seq
..............ucagacugcUggaccguu...............................................................    1     1     seq
..............ucagacugccggaccguA...............................................................    13    1     seq
..............Ccagacugccggaccguu...............................................................    1     1     seq
..............ucagacuCccggaccguu...............................................................    1     1     seq
..............ucagacAgccggaccguu...............................................................    1     1     seq
..............ucagacugccggaccgCu...............................................................    1     1     seq
..............ucagacugccggaccguuU..............................................................    5     1     seq
..............uUagacugccggaccguua..............................................................    1     1     seq
..............ucagacugccggaccguAa..............................................................    1     1     seq
..............ucagacugccggaccguua..............................................................    41    0     seq
..............ucagacugcUggaccguua..............................................................    1     1     seq
..............ucagacugccggaccAuuag.............................................................    1     1     seq
..............ucagacugccggaccguuUg.............................................................    1     1     seq
..............ucagacugccggaccguuag.............................................................    349   0     seq
..............ucagacugcUggaccguuag.............................................................    3     1     seq
..............ucagacCgccggaccguuag.............................................................    1     1     seq
..............ucaUacugccggaccguuag.............................................................    1     1     seq
..............ucagacugccggaccguAag.............................................................    2     1     seq
..............ucagacugccggaccguuaA.............................................................    16    1     seq
..............ucGgacugccggaccguuag.............................................................    2     1     seq
..............ucaAacugccggaccguuag.............................................................    1     1     seq
..............Ccagacugccggaccguuag.............................................................    2     1     seq
..............ucagacuCccggaccguuag.............................................................    1     1     seq
..............Acagacugccggaccguuag.............................................................    2     1     seq
..............ucagacugAcggaccguuag.............................................................    1     1     seq
..............ucagacugccggaccgCuag.............................................................    2     1     seq
..............ucagacugccggaGcguuag.............................................................    1     1     seq
..............ucagacugccggaccguuaC.............................................................    4     1     seq
..............ucagacugccggaccguuaU.............................................................    6     1     seq
..............ucagGcugccggaccguuag.............................................................    1     1     seq
..............ucagacugccggaccguCag.............................................................    2     1     seq
..............ucagCcugccggaccguuag.............................................................    2     1     seq
..............ucagacugccAgaccguuag.............................................................    1     1     seq
..............ucagacugccggaccguuGg.............................................................    2     1     seq
..............ucagacugccggacUguuag.............................................................    1     1     seq
..............ucagacugccgUaccguuagu............................................................    1     1     seq
..............ucagGcugccggaccguuagu............................................................    3     1     seq
..............ucagacugccggacAguuagu............................................................    1     1     seq
..............ucagacugccAgaccguuagu............................................................    2     1     seq
..............ucagacugccggaUcguuagu............................................................    1     1     seq
..............ucagacuUccggaccguuagu............................................................    1     1     seq
..............ucagacugccggaccCuuagu............................................................    1     1     seq
..............ucagacugccggGccguuagu............................................................    3     1     seq
..............ucagacugccggacUguuagu............................................................    3     1     seq
..............ucagacugccggaccguuaAu............................................................    3     1     seq
..............uUagacugccggaccguuagu............................................................    1     1     seq
..............uAagacugccggaccguuagu............................................................    1     1     seq
..............Ccagacugccggaccguuagu............................................................    2     1     seq
..............ucGgacugccggaccguuagu............................................................    2     1     seq
..............ucagacugccggaccguuagA............................................................    17    1     seq
..............ucagacugccggaccguuagG............................................................    14    1     seq
```

```
                                                                novel-nve-miR-37_star
              novel-nve-miR-37_guide
gagauaggauuauuucagacugccggaccguuaguggguuagcucccgggggcuuaaauagauaaacggucuggcaguccgaaauaauuauuuuag

..............ucagacugccggaccguuagu.............................................................   710   0   seq
..............ucagacugccggaccguuagC.............................................................   167   1   seq
..............ucagacCgccggaccguuagu.............................................................   3     1   seq
..............Gcagacugccggaccguuagu.............................................................   1     1   seq
..............ucagacugAcggaccguuagu.............................................................   1     1   seq
..............ucaUacugccggaccguuagu.............................................................   1     1   seq
..............ucagacugccggaccgAuagu.............................................................   1     1   seq
..............ucagacugccggaccguuGgu.............................................................   3     1   seq
..............Acagacugccggaccguuagu.............................................................   12    1   seq
..............ucagacugccggCccguuagu.............................................................   1     1   seq
..............ucagacugcAggaccguuagu.............................................................   2     1   seq
..............ucagacugccggaccAuuagu.............................................................   3     1   seq
..............ucagacugccggaccguCagu.............................................................   1     1   seq
..............ucagacugccggaccguuaguC............................................................   166   1   seq
..............ucagacugccggaccguuaguU............................................................   767   1   seq
..............ucagacugccggaccguuaguA............................................................   30    1   seq
..............ucagacugccggaccguuagug............................................................   12    0   seq
..............ucagacugccggaccguuaguUg...........................................................   3     1   seq
..............ucagacugccggaccguuagugA...........................................................   2     1   seq
..............ucagacugccggaccguuagugU...........................................................   1     1   seq
...............cagacugccggaccguuag..............................................................   1     0   seq
...............cagacugccggaccguuaguU............................................................   1     1   seq
................agacugccggaccguuagC.............................................................   1     1   seq
................agacugcAggaccguuagu.............................................................   1     1   seq
................agacugccggaccguuaguU............................................................   1     1   seq
............................................................gauaaacggucuggcaguUc................   1     1   seq
................................................................aacggucuggcaguccgaaa............   1     0   seq
................................................................aacggucuggcaguccgaaaua..........   1     0   seq
```

miRBase precursor : novel-nve-miR-42-a
Total read count : 107
novel-nve-miR-42-a_guide read count : 105
novel-nve-miR-42-a_star read count : 1
remaining reads : 1

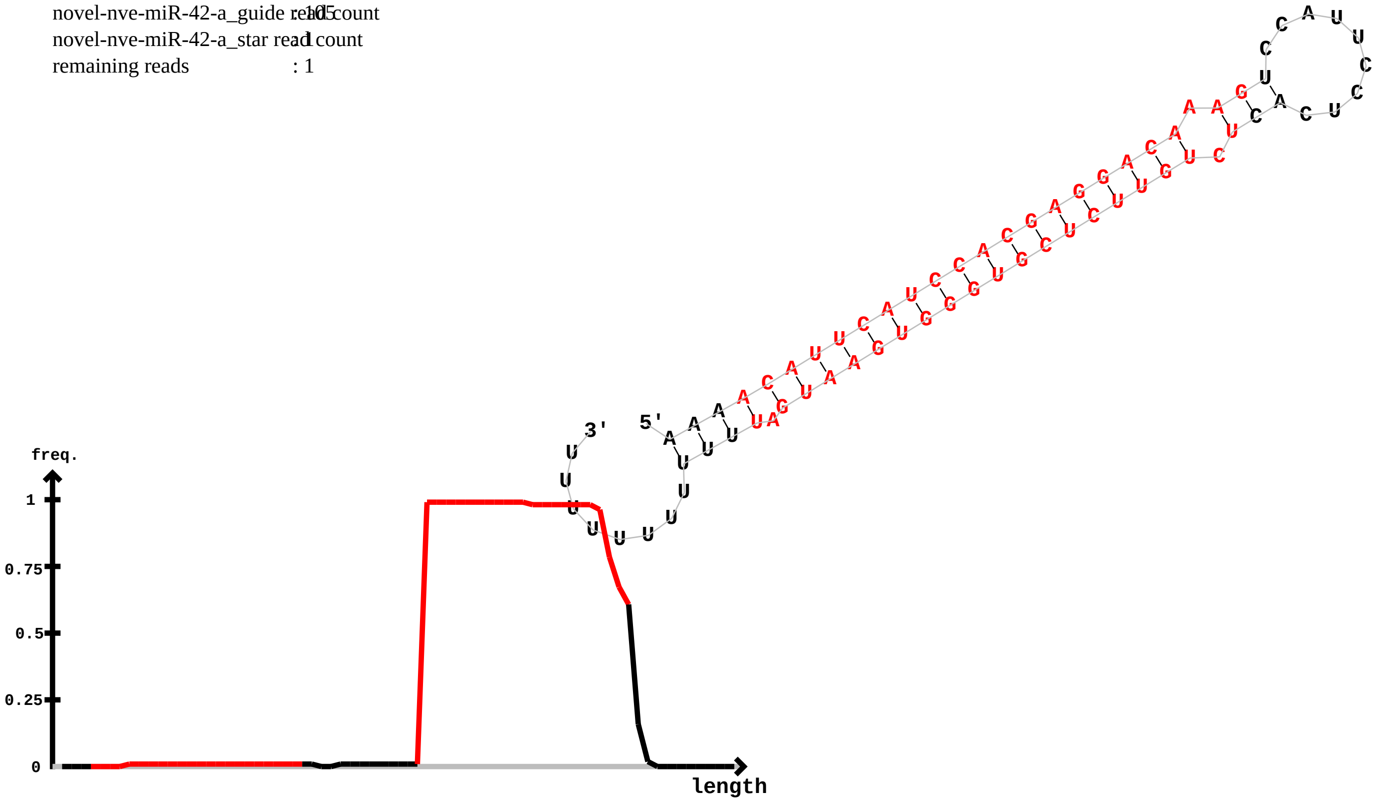


```
novel-nve-miR-42-a_star
                                   novel-nve-miR-42-a_guide
5'- aaaacauucauccacgaggacaaaguccauuccucacucuguucucgugggugaaugauuuuuuuuuuuu   -3'    exp
    ....((((((((((((((((((.(((.........))).))))))))))))))))))).............    reads      mm         sample
    ......uucauccacgaggacaaagu.............................................    1          0          seq
    ............................auuccucacucuguucucgA......................    1          1          seq
    .....................................ucuguucucgugggugaU...............    1          1          seq
    .....................................ucuguucucgugggugaa...............    1          0          seq
    .....................................ucuguucucgugggugaau..............    9          0          seq
    .....................................ucuguucucgugggugaaG..............    1          1          seq
    .....................................ucuguucCcgugggugaau..............    1          1          seq
    .....................................ucuguucucgugggugaaC..............    4          1          seq
    .....................................ucuguucucgugggugaaA..............    1          1          seq
    .....................................ucuguucucguggCugaau..............    1          1          seq
    .....................................ucuguucucgugggugaUu..............    1          1          seq
    .....................................ucuguucucgugggugGau..............    1          1          seq
    .....................................ucuguucucgugggugaauU.............    5          1          seq
    .....................................ucuguucucgugggugaaug.............    3          0          seq
    .....................................ucuguucucgugggugaauA.............    2          1          seq
    .....................................ucuguucucgugggugaauC.............    2          1          seq
    .....................................ucuguucucgugggugaauga............    6          0          seq
    .....................................ucuguucucgugggugaaugU............    1          1          seq
    .....................................ucuguAcucgugggugaaugau...........    1          1          seq
    .....................................ucuguucucgugggugaaugau...........    35         0          seq
    .....................................ucuguucucgugggugaaugaG...........    1          1          seq
    .....................................uAuguucucgugggugaaugau...........    1          1          seq
    .....................................uUuguucucgugggugaaugau...........    1          1          seq
    .....................................ucuguucucgugggCgaaugau...........    1          1          seq
    .....................................ucuguucucgugggugaaugaC...........    8          1          seq
    .....................................ucuguucucAugggugaaugauu..........    1          1          seq
    .....................................ucuguucucgugggugaaugauu..........    11         0          seq
    .....................................ucuguucucgugggugaaugauC..........    3          1          seq
    .....................................ucuguucucgugggugaaugauAu.........    1          1          seq
    .....................................ucuguucucgugggugaaugauuu.........    1          0          seq
```

miRBase precursor : novel-nve-miR-42-b
Total read count : 435
novel-nve-miR-42-b_guide read count : 434
novel-nve-miR-42-b_star read count : 1
remaining reads : 0

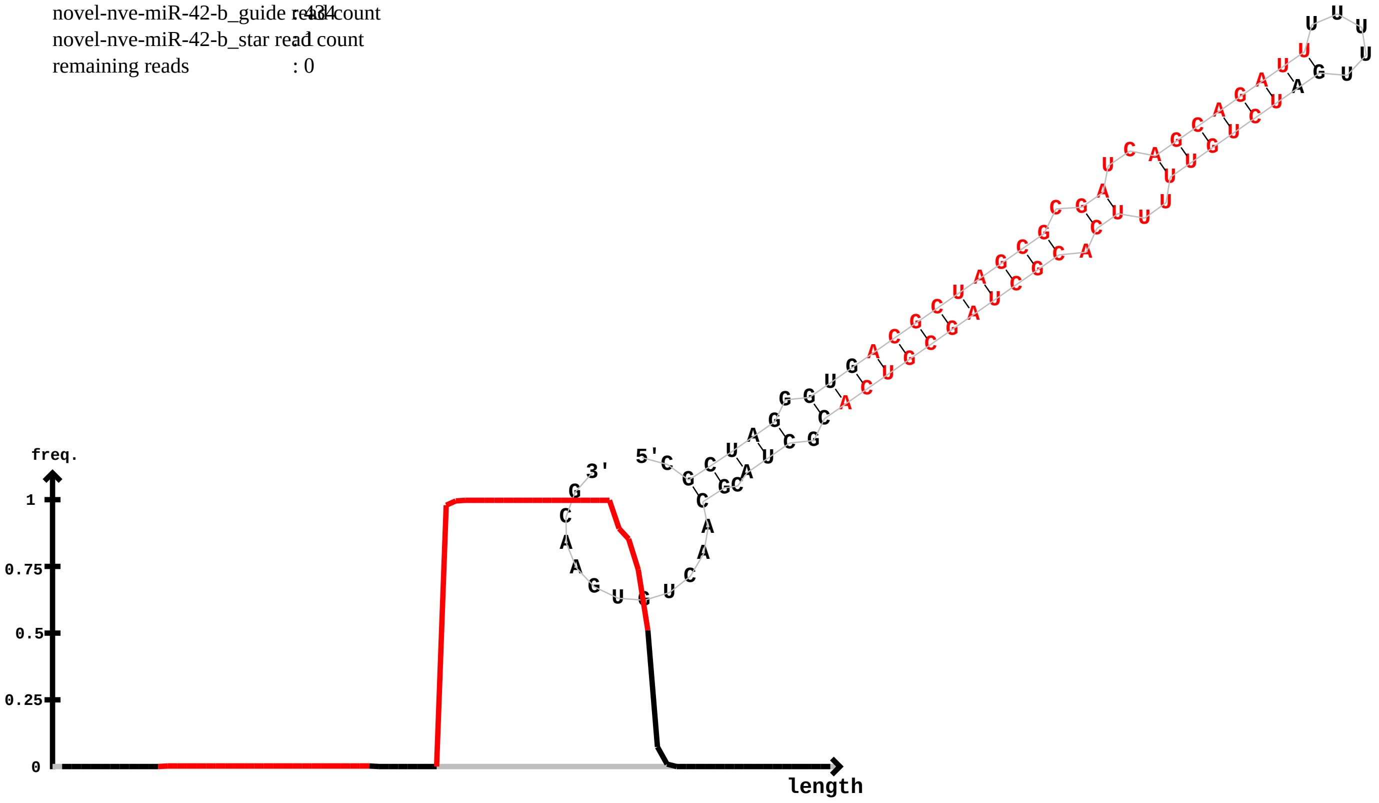

novel-nve-miR-42-b_guide

novel-nve-miR-42-b_star

```
5'- cgcuagggugacgcuagcgcgaucagcagauuuuuugaucuguuuuucacgcuagcgucacgcuacgcaacugugaacg  -3'    exp
    .(((((.((((((((((((.((..((((((((.....))))))))..)).))))))))))))).))).))..........    reads    mm    sample
    ..........acgcuagcgcgaucagcagauu................................................    1        0     seq
    .......................................ucuguuuuucacgcuagc.......................    30       0     seq
    .......................................ucuguuuuucacgcuagU.......................    12       1     seq
    .......................................ucuguuuCucacgcuagc.......................    1        1     seq
    .......................................ucuguuuuucacgcuagA.......................    2        1     seq
    .......................................ucuguuuuucaAgcuagc.......................    1        1     seq
    .......................................ucuguuuuucacgcuagcU......................    1        1     seq
    .......................................ucAguuuuucacgcuagcg......................    1        1     seq
    .......................................ucuguuuuucacgcuagcA......................    4        1     seq
    .......................................ucugAuuuucacgcuagcg......................    1        1     seq
    .......................................ucuguuuuucacgcuagcg......................    10       0     seq
    .......................................ucuguuuuucGcgcuagcgu.....................    1        1     seq
    .......................................ucuguuCuucacgcuagcgu.....................    1        1     seq
    .......................................ucuguuuuucacgcuagcgC.....................    5        1     seq
    .......................................uUuguuuuucacgcuagcgu.....................    1        1     seq
    .......................................ucuguAuuucacgcuagcgu.....................    1        1     seq
    .......................................ucuguuuuucacgcuagcgG.....................    2        1     seq
    .......................................ucuguuuuucacgcuagcgu.....................    35       0     seq
    .......................................ucuguuuuucacgcuagcgA.....................    2        1     seq
    .......................................ucuUuuuuucacgcuagcguc....................    1        1     seq
    .......................................Ccuguuuuucacgcuagcguc....................    1        1     seq
    .......................................ucuguuuuucacUcuagcguc....................    1        1     seq
    .......................................ucuguuuuucacgcuagcguA....................    4        1     seq
    .......................................ucuguuuuAacgcuagcguc....................    1        1     seq
    .......................................ucuguuuuucacgcuagcguc....................    62       0     seq
    .......................................ucuguuuuCcacgcuagcguc....................    1        1     seq
    .......................................ucuguuuuucacgcuagcguU....................    24       1     seq
    .......................................ucuguuuuucacgcuagcgCc....................    1        1     seq
    .......................................ucuguuAuucacgcuagcguca...................    1        1     seq
    .......................................ucuguuuuucacgcuagcgucC...................    2        1     seq
    .......................................ucuguuuuCcacgcuagcguca...................    1        1     seq
    .......................................ucuguuuuucacAcuagcguca...................    2        1     seq
    .......................................ucuguuCuucacgcuagcguca...................    2        1     seq
    .......................................ucuguuuuucacgcuagcgCca...................    2        1     seq
```

novel-nve-miR-42-b_guide

novel-nve-miR-42-b_star

cgcuagggugacgcuagcgcgaucagcagauuuuuuugaucuguuuuucacgcuagcgucacgcuacgcaacugugaacg

....ucuguuuuucacgcGagcguca...................	1	1	seq
....ucuguuuuucacgcuagcguca...................	154	0	seq
....ucugGuuuucacgcuagcguca...................	1	1	seq
....ucuguuuuucGcgcuagcguca...................	1	1	seq
....ucuguuuuucaAgcuagcguca...................	1	1	seq
....ucugCuuuucacgcuagcguca...................	1	1	seq
....ucuguuuuucacgcuagcgucU...................	13	1	seq
....Ccuguuuuucacgcuagcguca...................	1	1	seq
....ucuguuuuucacgcuagcgucG...................	5	1	seq
....ucuguuuuucacgcuagcgucaA..................	2	1	seq
....ucuguuuuucacgcuagcgucac..................	7	0	seq
....ucuguuuuucacgcuagcgucaU..................	18	1	seq
....ucuguuuuucacgcuagcgucacC.................	1	1	seq
....ucuguuuuucacgcuagcgucacU.................	2	1	seq
....ucuguuuuucacgcuagcgucacA.................	1	1	seq
....cuguuuuucacgcuagUgu.....................	1	1	seq
....cuguuuuucacgcuagcgu.....................	1	0	seq
....cuguuuuucacgcuagcguA....................	1	1	seq
....cuguuuuucacgcuagcguc....................	2	0	seq
....cuguuuCucacgcuagcguca...................	1	1	seq
....cuguuuuucacgcuagcgucU...................	1	1	seq
....uguuuuucacgcuagcgucaU..................	1	1	seq

miRBase precursor : novel-nve-miR-43
Total read count : 81
novel-nve-miR-43_guide read count : 81
novel-nve-miR-43_star read count : 0
remaining reads : 0

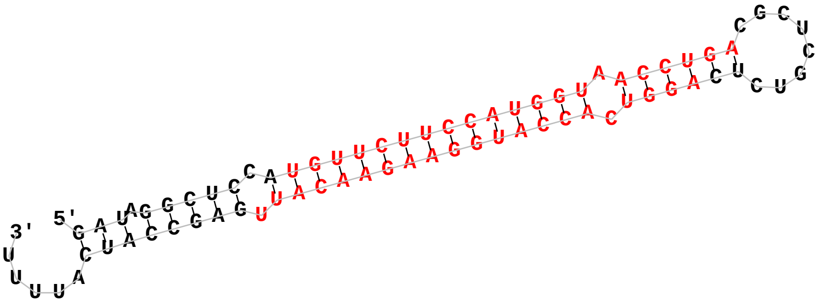

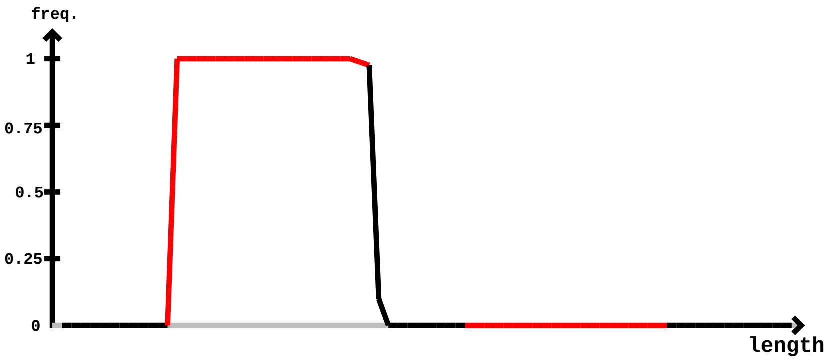


```
                                                            novel-nve-miR-43_star
                novel-nve-miR-43_guide
5'- gauaggcuccauguucuuccaugguaaccugacgcucgucucaggucaccauggaagaacauugagccaucauuuu   -3'    exp
    (((.(((((.(((((((((((((((.((((((........)))))).))))))))))))))).)))))))).....    reads     mm        sample
    ...........uguucuuccaugguaaccu...............................................    1         0         seq
    ...........uguucuuccaugguaaccug..............................................    1         0         seq
    ...........uguucuuccaugguaaccugU.............................................    1         1         seq
    ...........uguucCuccaugguaaccuga.............................................    2         1         seq
    ...........uguucuuccaugguaaccugG.............................................    1         1         seq
    ...........uguucuuccaugguaaccuga.............................................    64        0         seq
    ...........uguuAuuccaugguaaccuga.............................................    1         1         seq
    ...........uguucuuccaugguaaccCga.............................................    2         1         seq
    ...........uguucuuccaugguaaccugac............................................    2         0         seq
    ...........uguucuuccaugguaaccugaU............................................    6         1         seq
```

miRBase precursor : novel-nve-miR-46
Total read count : 5578
novel-nve-miR-46_guide read count : 2789
novel-nve-miR-47-1_star read count : 2782
novel-nve-miR-46_star read count : 3
remaining reads : 4

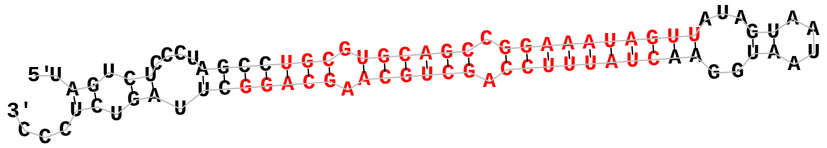

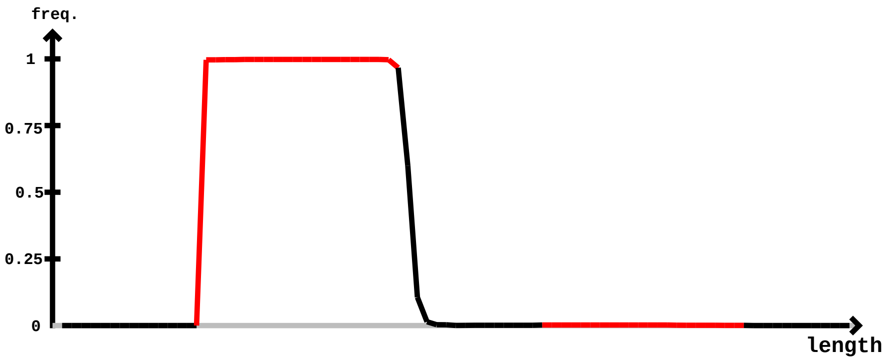


```
                                                    novel-nve-miR-46_star
               novel-nve-miR-47-1_star
               novel-nve-miR-46_guide
5'- uagucucccuagccugcgugcagccggaaauaguuauaguaauaauggaacuauuuccagcugcaagcaggcuuagucuccc   -3'    exp
    .((.((....(((((((.((((((.((((((((((...((....))..)))))))))).)))))).))))))).)).))...     reads      mm          sample
    ..............ugcgugcagccggaaauag.................................................     2          0           seq
    ..............ugcgugUagccggaaauag.................................................     1          1           seq
    ..............ugcgugcagccggaaauaAu................................................     3          1           seq
    ..............ugcgugcagccggaaauagu................................................     66         0           seq
    ..............ugcgugcagccggaaauagC................................................     5          1           seq
    ..............uAcgugcagccggaaauagu................................................     1          1           seq
    ..............ugcgugcagccggaGauagu................................................     1          1           seq
    ..............ugcgugcagccggaaaCagu................................................     5          1           seq
    ..............ugcgugcagccggaaauagA................................................     1          1           seq
    ..............ugcgugcagccggaaauagG................................................     2          1           seq
    ..............ugcgugcagGcggaaauaguu...............................................     2          1           seq
    ..............Agcgugcagccggaaauaguu...............................................     6          1           seq
    ..............ugcgAgcagccggaaauaguu...............................................     1          1           seq
    ..............ugcCugcagccggaaauaguu...............................................     2          1           seq
    ..............ugcgugcGgccggaaauaguu...............................................     1          1           seq
    ..............ugcgugcagccggaaauaguC...............................................     138        1           seq
    ..............ugcgugcagccggaaauUguu...............................................     1          1           seq
    ..............ugcgugcagccggaCauaguu...............................................     1          1           seq
    ..............ugcgugcagccggGaauaguu...............................................     5          1           seq
    ..............ugcgugcagccggaaGuaguu...............................................     3          1           seq
    ..............ugAgugcagccggaaauaguu...............................................     1          1           seq
    ..............uUcgugcagccggaaauaguu...............................................     3          1           seq
    ..............ugcgugcagccggaaauGguu...............................................     1          1           seq
    ..............ugcgugUagccggaaauaguu...............................................     4          1           seq
    ..............ugcgugcagccggaaaAaguu...............................................     1          1           seq
    ..............ugcgugcagccggaaaCaguu...............................................     1          1           seq
    ..............ugcgugcagccggaaauaguu...............................................     805        0           seq
    ..............ugcgugcagccggaaauaAuu...............................................     2          1           seq
    ..............ugcAugcagccggaaauaguu...............................................     2          1           seq
    ..............ugcUugcagccggaaauaguu...............................................     1          1           seq
    ..............ugcguCcagccggaaauaguu...............................................     1          1           seq
    ..............ugcgugcagccggaaauagGu...............................................     1          1           seq
    ..............Cgcgugcagccggaaauaguu...............................................     2          1           seq
```

```
                                                  novel-nve-miR-46_star
                  novel-nve-miR-47-1_star
              novel-nve-miR-46_guide
uagucucccuagcc[illegible]uaguaauaauggaacuauuuccagcugcaagcaggcuuagucuccc

..............ugcgugcagccggaaauagAu...............................................    3      1      seq
..............uAcgugcagccggaaauaguu...............................................    1      1      seq
..............ugcgugcagcGggaaauaguu...............................................    1      1      seq
..............ugcgugcagccUgaaauaguu...............................................    3      1      seq
..............ugcgCgcagccggaaauaguu...............................................    4      1      seq
..............ugcgugcagccggaaUuaguu...............................................    1      1      seq
..............ugcgugcagccggaGauaguu...............................................    7      1      seq
..............Ggcgugcagccggaaauaguu...............................................    2      1      seq
..............ugcgugcagccggaaauagCu...............................................    2      1      seq
..............ugGgugcagccggaaauaguu...............................................    1      1      seq
..............ugcguAcagccggaaauaguu...............................................    2      1      seq
..............ugcgugcagccggaUauaguu...............................................    1      1      seq
..............ugcgugcagccgAaaauaguu...............................................    2      1      seq
..............ugcgugcagccggaaauaguA...............................................    12     1      seq
..............ugcgugGagccggaaauaguua..............................................    1      1      seq
..............ugcgugcagccggaaauaguuC..............................................    74     1      seq
..............ugcgugcagccggaaaAaguua..............................................    1      1      seq
..............ugcgugcagccggaaUuaguua..............................................    1      1      seq
..............ugcgugcagUcggaaauaguua..............................................    4      1      seq
..............ugcgugcagccggaaauGguua..............................................    2      1      seq
..............ugcgugcUgccggaaauaguua..............................................    2      1      seq
..............ugcgugcagccggaaauaguua..............................................    1106   0      seq
..............ugcgugcagccgCaaauaguua..............................................    2      1      seq
..............ugcgugcagccUgaaauaguua..............................................    1      1      seq
..............Ggcgugcagccggaaauaguua..............................................    1      1      seq
..............ugcgugAagccggaaauaguua..............................................    1      1      seq
..............ugcgugcagcGggaaauaguua..............................................    1      1      seq
..............ugcgugcagccggaaaCaguua..............................................    1      1      seq
..............Agcgugcagccggaaauaguua..............................................    10     1      seq
..............ugcgugcagccggaaauUguua..............................................    1      1      seq
..............ugcgugcagccggaUauaguua..............................................    1      1      seq
..............ugcgCgcagccggaaauaguua..............................................    8      1      seq
..............ugcgAgcagccggaaauaguua..............................................    5      1      seq
..............ugcgugcagcAggaaauaguua..............................................    2      1      seq
..............uAcgugcagccggaaauaguua..............................................    2      1      seq
..............ugcgugcagccggaaauagCua..............................................    2      1      seq
..............ugcgugcagccggaGauaguua..............................................    7      1      seq
..............ugcgugcagccggaaauaguCa..............................................    4      1      seq
..............ugcgugcagccggaaauaguuU..............................................    80     1      seq
..............ugcgugcagccAgaaauaguua..............................................    1      1      seq
..............ugcgugcaAccggaaauaguua..............................................    2      1      seq
..............ugcgugcGgccggaaauaguua..............................................    6      1      seq
..............ugcgugUagccggaaauaguua..............................................    2      1      seq
..............ugcgugcagccggaaauaguGa..............................................    1      1      seq
..............ugcgugcagccggaaauaguuG..............................................    2      1      seq
..............ugUgugcagccggaaauaguua..............................................    7      1      seq
..............ugcgugcagccggaaauagAua..............................................    2      1      seq
..............ugcgugcagccggGaauaguua..............................................    5      1      seq
..............ugcgugcagccgUaaauaguua..............................................    1      1      seq
..............ugcgugcagccgAaaauaguua..............................................    1      1      seq
..............Cgcgugcagccggaaauaguua..............................................    8      1      seq
..............ugcAugcagccggaaauaguua..............................................    3      1      seq
..............ugcgugcagccggaaauaAuua..............................................    2      1      seq
..............ugcgugcagcUggaaauaguua..............................................    4      1      seq
..............ugcgugcagccggaaauagGua..............................................    1      1      seq
..............ugcgugcagccggaaGuaguua..............................................    10     1      seq
..............ugcgugcagccggaaauaguuCu.............................................    17     1      seq
..............ugcgugUagccggaaauaguuau.............................................    1      1      seq
..............ugcgugcagccggaaauaguuUu.............................................    5      1      seq
..............ugcgugcagccggaaauagCuau.............................................    1      1      seq
..............ugcgAgcagccggaaauaguuau.............................................    1      1      seq
..............ugcguCcagccggaaauaguuau.............................................    1      1      seq
..............ugcgugcagccAgaaauaguuau.............................................    2      1      seq
..............Cgcgugcagccggaaauaguuau.............................................    1      1      seq
..............ugcgugcagccggaaauaguuaG.............................................    3      1      seq
..............ugUgugcagccggaaauaguuau.............................................    1      1      seq
..............ugcgugcagccggaaauUguuau.............................................    1      1      seq
..............ugcgugcagccUgaaauaguuau.............................................    1      1      seq
```

novel-nve-miR-46_star

novel-nve-miR-47-1_star

novel-nve-miR-46_guide

uagucucccuagccugcgugcagccggaaauaguuauaguaauaauggaacuauuuccagcugcaagcaggcuuagucuccc

..............ugcgugcagccggaaauaguuaC..	54	1	seq
..............ugcguAcagccggaaauaguuau..	1	1	seq
..............ugcgugcagccggaaauaguuau..	143	0	seq
..............ugcgugcagccggaaauGguuau..	1	1	seq
..............ugcgugcagccggaaauaguuaA..	19	1	seq
..............ugcgugcagccggGaauaguuau..	1	1	seq
..............ugcgugcagccggaaauaguuaCa...	4	1	seq
..............ugcgugcagccggaaauaguuauC...	3	1	seq
..............ugcgugcagccggaaauaguuaua...	7	0	seq
..............ugcgugcagccggaaauaguuaAa...	2	1	seq
..............ugcgugcagccggaaauCguuaua...	1	1	seq
..............ugcgugcagccggaaauaguuauU...	14	1	seq
..............ugcgugcagccggaaauaguuauaC..	1	1	seq
..............ugcgugcagccggaaauaguuauaAu...	4	1	seq
..............ugcgugcagccggaaauaguuauaCu...	3	1	seq
..............ugcgugcagccggaaauaguuauaUua..	1	1	seq
................cgugcagccggaaauaguuaC..	1	1	seq
................cgugcagccggaaauaguuau..	1	0	seq
..................ugcagccggaaauagAua...	2	1	seq
....auaauggaacuauuuccGgcug..................	2	1	seq
....uaauggaacuauuuccGgcugc.................	1	1	seq
....acuauuuccagcugcaCgc.............	1	1	seq
....acuauuuccagcugcaCgcagg..........	2	1	seq

Provisional ID		novel-nve-miR-47-1
Score total	:	850.4
Score for star read(s)	:	3.9
Score for read counts	:	839.8
Score for mfe	:	2.1
Score for randfold	:	1.6
Score for cons. seed	:	3
Total read count	:	1659
Mature read count	:	1654
Loop read count	:	0
Star read count	:	5

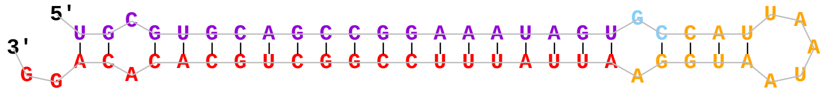

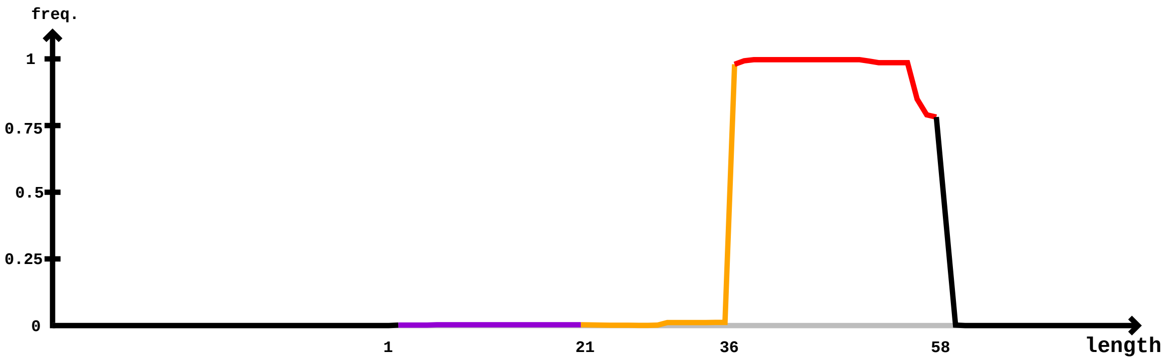


```
                                       Star                                Mature
5'- ucucaacauaacacaguuucaguuuauuuuagccugcgugcagccggaaauagugccauuaauaauggaauuauuuccggcugcacacaggcuucauuaaauaguuaaauaaa  -3'   obs
    ucucaacauaacacaguuucaguuuauuuuagccugcgugcagccggaaauagugccauuaauaauggaauuauuuccggcugcacacaggcuucauuaaauaguuaaauaaa        exp
    .......................(((((..((((((.((((((((((((((((((.((((.....)))).)))))))))))))))))).)))))).....)))))........   reads   mm   sample
    ..................................ugcgugcagccggaaauagu...........................................................   1       0    seq
    ..................................ugcgugcagccggaaauagG...........................................................   1       1    seq
    ..................................ugUgugcagccggaaauagugc.........................................................   1       1    seq
    ......................................ugcagccggaaauaguCccau......................................................   1       1    seq
    ......................................ugcagccggaaauagugccauua....................................................   1       0    seq
    .............................................................auaauggaaCuauuuccggcug..............................   3       1    seq
    ..............................................................uaauggaaCuauuuccggcug..............................   6       1    seq
    ..............................................................uaauggaaCuauuuccggcugc.............................   10      1    seq
    ...................................................................Aaauuauuuccggcugcacacagg......................   1       1    seq
    .....................................................................auuauuuccggcugcacaA.........................   8       1    seq
    .....................................................................auuauuuccggcugcaNac.........................   1       1    seq
    .....................................................................auuauuuccggcuUcacac.........................   1       1    seq
    .....................................................................auuauAuccggcugcacac.........................   1       1    seq
    .....................................................................auuauuuccggcugcacaU.........................   66      1    seq
    .....................................................................auuauuuccggcugcacac.........................   145     0    seq
    .....................................................................auuauuuAcggcugcacac.........................   1       1    seq
    .....................................................................Guuauuuccggcugcacac.........................   3       1    seq
    .....................................................................auuauuCccggcugcacac.........................   1       1    seq
    .....................................................................auuauuuccggcAgcacac.........................   1       1    seq
    .....................................................................auuauuuccggcugcGcac.........................   1       1    seq
    .....................................................................auuauuuccggcugcacacU........................   4       1    seq
    .....................................................................auuauuuccggcugcacacG........................   2       1    seq
    .....................................................................aAuauuuccggcugcacaca........................   1       1    seq
    .....................................................................auuauuuccggcCgcacaca........................   1       1    seq
    .....................................................................Guuauuuccggcugcacaca........................   1       1    seq
    .....................................................................auuauuuGcggcugcacaca........................   1       1    seq
    .....................................................................auCauuuccggcugcacaca........................   1       1    seq
    .....................................................................auuauuuccggcugcacaca........................   86      0    seq
    .....................................................................auuauuCccggcugcacaca........................   2       1    seq
    .....................................................................auuauuuccggcugcacacaA.......................   3       1    seq
    .....................................................................auuauuuccggcugcacacag.......................   10      0    seq
    .....................................................................auuauuuccggcugcacacaC.......................   1       1    seq
    .....................................................................auuauuuccggcugcacacaAg......................   1       1    seq
```

```
                                   Star                                 Mature
ucucaacauaacacaguuucaguuuauuuuagccugcgugcagccggaaauagugccauuaaauaauggaauuauuuccggcugcacacaggcuucauuaaauaguuaaauaaa
......................................................................auCauuuccggcugcacacagg......................	4	1	seq
......................................................................aAuauuuccggcugcacacagg......................	6	1	seq
......................................................................auuauuuccggcugcacacCgg......................	4	1	seq
......................................................................auuauuuccggcugcacacagU......................	44	1	seq
......................................................................auuauuuccggcuAcacacagg......................	1	1	seq
......................................................................auAauuuccggcugcacacagg......................	1	1	seq
......................................................................auuauuuccAgcugcacacagg......................	1	1	seq
......................................................................auuauuuccggcugcUcacagg......................	4	1	seq
......................................................................auuauuuccggcugUacacagg......................	3	1	seq
......................................................................Guuauuuccggcugcacacagg......................	14	1	seq
......................................................................auuauuuccggcCgcacacagg......................	3	1	seq
......................................................................auuauuucAggcugcacacagg......................	3	1	seq
......................................................................auuauuuccggUugcacacagg......................	2	1	seq
......................................................................auuauuuccggcugcacacUgg......................	3	1	seq
......................................................................auuaCuuccggcugcacacagg......................	1	1	seq
......................................................................auuauuuccggcugcacacagC......................	10	1	seq
......................................................................auuauuuccggcugcacacGgg......................	7	1	seq
......................................................................auuGuuuccggcugcacacagg......................	1	1	seq
......................................................................auuauCuccggcugcacacagg......................	2	1	seq
......................................................................auuauuuccggcGgcacacagg......................	1	1	seq
......................................................................auuauuuccggcugcacaUagg......................	1	1	seq
......................................................................auuauuuccggcugcaUacagg......................	6	1	seq
......................................................................auuauuuccggcugcacacagg......................	963	0	seq
......................................................................auuCuuuccggcugcacacagg......................	1	1	seq
......................................................................auuauuAccggcugcacacagg......................	2	1	seq
......................................................................auuauuuccggcugcacacagA......................	145	1	seq
......................................................................auuauuCccggcugcacacagg......................	5	1	seq
......................................................................auuauuuccggcugcacUcagg......................	2	1	seq
......................................................................auuauuuccggcAgcacacagg......................	1	1	seq
......................................................................auuauAuccggcugcacacagg......................	2	1	seq
......................................................................auuauuuccggcugcacacaUg......................	1	1	seq
......................................................................auuauuuccgAcugcacacagg......................	1	1	seq
......................................................................aGuauuuccggcugcacacagg......................	1	1	seq
......................................................................auuauuuccggcugcGcacagg......................	4	1	seq
......................................................................auuauuuccggAugcacacagg......................	2	1	seq
......................................................................auuaAuuccggcugcacacagg......................	2	1	seq
......................................................................Uuuauuuccggcugcacacagg......................	2	1	seq
......................................................................auuauuuccggcugcacacaggA.....................	1	1	seq
......................................................................auuauuuccggcugcacacaggU.....................	22	1	seq
......................................................................auuauuuccggcugcacacaggc.....................	2	0	seq
.......................................................................uuauuuccggcugcacacagg......................	7	0	seq
.......................................................................uuauuuccggcugcacacagA......................	1	1	seq
.......................................................................uuauuuccggcugcacacaggc.....................	10	0	seq
.......................................................................uuauuuccggcugcacacaggU.....................	4	1	seq
........................................................................uauuuccggcugcacacagg......................	1	0	seq
........................................................................Aauuuccggcugcacacagg......................	1	1	seq
........................................................................uauuuccggcugcacacaggc.....................	2	0	seq
........................................................................uauuuccggcugcacacaggcA....................	1	1	seq
........................................................................uauuuccggcugcacacaggcu....................	2	0	seq
```

Provisional ID	:	Novel-nve-miR-47-2
Score total	:	957.3
Score for star read(s)	:	3.9
Score for read counts	:	946.4
Score for mfe	:	2.4
Score for randfold	:	1.6
Score for cons. seed	:	3
Total read count	:	1868
Mature read count	:	1654
Loop read count	:	0
Star read count	:	214

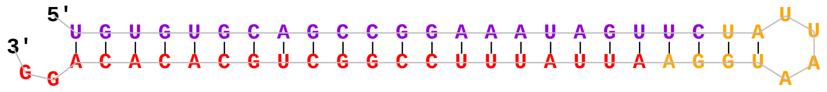

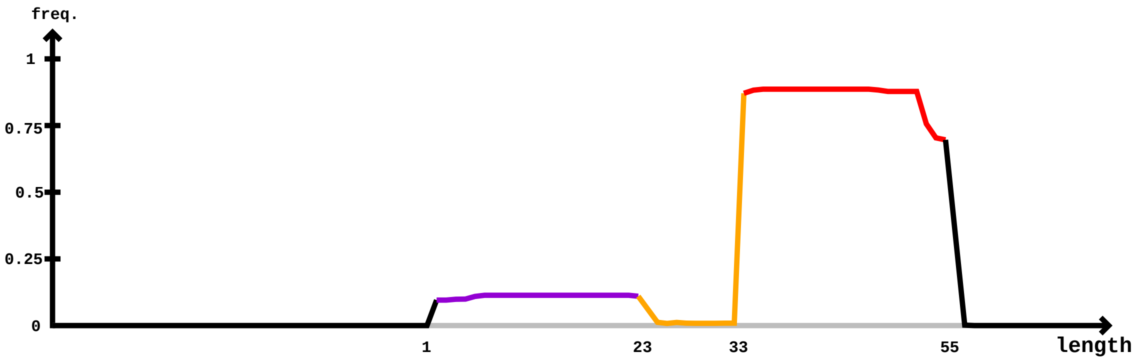


```
                                          Star                            Mature
5'- ugucgaaacgcuacaaaaagccauggcaggucuaggccugugugcagccggaaauaguucuauuaauggaauuauuuccggcugcacacaggcuaagcaacgcuaaga  -3'   obs
    ugucgaaacgcuacaaaaagccauggcaggucuaggccugugugcagccggaaauaguucuauuaauggaauuauuuccggcugcacacaggcuaagcaacgcuaaga         exp
    .........(((......)))..((((..(.((.((((((((((((((((((((((((((((....)))))))))))))))))))))))))))).)))...))))...   reads      mm        sample
    ......................................ugugugcagccggaaauGguu.................................................   1          1         seq
    ......................................ugugugcagccggaaauaguC.................................................   2          1         seq
    ......................................ugugugcagccggaaauaguu.................................................   4          0         seq
    ......................................ugugugcagccggaaauaguGc................................................   1          1         seq
    ......................................Agugugcagccggaaauaguuc................................................   2          1         seq
    ......................................ugugugcGgccggaaauaguuc................................................   4          1         seq
    ......................................ugugugcagcAggaaauaguuc................................................   1          1         seq
    ......................................ugugugcagccggaaauaguCc................................................   1          1         seq
    ......................................Cgugugcagccggaaauaguuc................................................   1          1         seq
    ......................................ugugugcagccggaaauaguuA................................................   7          1         seq
    ......................................ugugugcagccggaaauagCuc................................................   1          1         seq
    ......................................ugugugcagccggaUauaguuc................................................   1          1         seq
    ......................................ugugugcagcUggaaauaguuc................................................   1          1         seq
    ......................................ugugugcagcGggaaauaguuc................................................   1          1         seq
    ......................................ugugugcagccggaaauaguuc................................................   81         0         seq
    ......................................ugugugcagccggaaauaguuU................................................   14         1         seq
    ......................................Cgugugcagccggaaauaguucu...............................................   1          1         seq
    ......................................ugugugcagAcggaaauaguucu...............................................   1          1         seq
    ......................................ugugugcagccggaaGuaguucu...............................................   1          1         seq
    ......................................ugugugcagccggaaauaguucC...............................................   4          1         seq
    ......................................ugugugcagccggaaauaguucu...............................................   39         0         seq
    ......................................ugugugcagccggaaauaguucA...............................................   5          1         seq
    ......................................ugugugcagccggaaauaguucG...............................................   2          1         seq
    ......................................ugugugcagccggaaauaAuucua..............................................   1          1         seq
    ......................................ugugugcagccggaaauaguucuC..............................................   2          1         seq
    ......................................ugugugcagccggaaauaguucuU..............................................   1          1         seq
    ........................................ugugcagccggaaauaguucu...............................................   2          0         seq
    ........................................ugugcagccggaaauaguucua..............................................   3          0         seq
    ........................................ugugcagccggaaauaguucuau.............................................   1          0         seq
    .........................................gugcagccggaaauaguucA...............................................   1          1         seq
    ..........................................ugcagccggaaauaguuc................................................   2          0         seq
    ..........................................ugcagccggaaauaguucA...............................................   1          1         seq
    ..........................................ugcagccggaaauaguucu...............................................   9          0         seq
```

Star **Mature**

```
ugucgaaacgcuacaaaaagccauggcaggucuaggccugugugcagccggaaauaguucuauuaauggaauuauuuccggcugcacacaggcuaagcaacgcuaaga
..........................................ugcagccggaaauaguucua..............................................  1    0  seq
..........................................ugcagccggaaauaguucuaC.............................................  1    1  seq
..........................................ugcagccggaaauaguucuau.............................................  1    0  seq
..........................................ugcagccggaaauaguucuauC............................................  2    1  seq
..........................................ugcagccggaaauaguucuauu............................................  1    0  seq
..........................................ugcagccggaaGuaguucuauua...........................................  1    1  seq
...........................................gcagccggaaauaguucuaC.............................................  1    1  seq
...........................................gcagccggaaauaguucuau.............................................  5    0  seq
...........................................gcagccggaaauaguucuauu............................................  2    0  seq
...............................................................uaauggaaCuauuuccggcug........................  6    1  seq
...............................................................uaauggaaCuauuuccggcugc.......................  10   1  seq
....................................................................Aaauuauuuccggcugcacacagg................  1    1  seq
......................................................................auuauuuAcggcugcacac...................  1    1  seq
......................................................................auuauuuccggcugcGcac...................  1    1  seq
......................................................................auuauuuccggcuUcacac...................  1    1  seq
......................................................................auuauuCccggcugcacac...................  1    1  seq
......................................................................auuauuuccggcugcacaA...................  8    1  seq
......................................................................auuauAuccggcugcacac...................  1    1  seq
......................................................................auuauuuccggcugcacaU...................  66   1  seq
......................................................................Guuauuuccggcugcacac...................  3    1  seq
......................................................................auuauuuccggcugcaNac...................  1    1  seq
......................................................................auuauuuccggcugcacac...................  145  0  seq
......................................................................auuauuuccggcAgcacac...................  1    1  seq
......................................................................auuauuuccggcugcacacG..................  2    1  seq
......................................................................auuauuuccggcugcacaca..................  86   0  seq
......................................................................auuauuuccggcCgcacaca..................  1    1  seq
......................................................................auuauuuGcggcugcacaca..................  1    1  seq
......................................................................Guuauuuccggcugcacaca..................  1    1  seq
......................................................................auuauuCccggcugcacaca..................  2    1  seq
......................................................................auCauuuccggcugcacaca..................  1    1  seq
......................................................................auuauuuccggcugcacacU..................  4    1  seq
......................................................................aAuauuuccggcugcacaca..................  1    1  seq
......................................................................auuauuuccggcugcacacaC.................  1    1  seq
......................................................................auuauuuccggcugcacacag.................  10   0  seq
......................................................................auuauuuccggcugcacacaA.................  3    1  seq
......................................................................auuauuuccggcugcacacagg................  963  0  seq
......................................................................auuauuuccggcugcacacagU................  44   1  seq
......................................................................auuauAuccggcugcacacagg................  2    1  seq
......................................................................auuauCuccggcugcacacagg................  2    1  seq
......................................................................auuauuuccggUugcacacagg................  2    1  seq
......................................................................auuauuuccggcugcacacCgg................  4    1  seq
......................................................................auuauuuccggcugcaUacagg................  6    1  seq
......................................................................auAauuuccggcugcacacagg................  1    1  seq
......................................................................auuaAuuccggcugcacacagg................  2    1  seq
......................................................................auuauuuccggcugcacacaUg................  1    1  seq
......................................................................auuauuuccggcugcGcacagg................  4    1  seq
......................................................................auuauuuccggcugcacaUagg................  1    1  seq
......................................................................auuaCuuccggcugcacacagg................  1    1  seq
......................................................................auuauuuccAgcugcacacagg................  1    1  seq
......................................................................auuauuuccggcugcacacaAg................  1    1  seq
......................................................................auuauuuccggcGgcacacagg................  1    1  seq
......................................................................auuauuuccgAcugcacacagg................  1    1  seq
......................................................................auCauuuccggcugcacacagg................  4    1  seq
......................................................................auuauuuccggcuAcacacagg................  1    1  seq
......................................................................auuauuuccggAugcacacagg................  2    1  seq
......................................................................auuauuucAggcugcacacagg................  3    1  seq
......................................................................auuauuuccggcugcacacagA................  145  1  seq
......................................................................auuauuCccggcugcacacagg................  5    1  seq
......................................................................auuauuuccggcugcacUcagg................  2    1  seq
......................................................................auuCuuuccggcugcacacagg................  1    1  seq
......................................................................aGuauuuccggcugcacacagg................  1    1  seq
......................................................................auuauuuccggcugcacacagC................  10   1  seq
......................................................................auuauuuccggcAgcacacagg................  1    1  seq
......................................................................auuauuuccggcugcacacGgg................  7    1  seq
......................................................................Uuuauuuccggcugcacacagg................  2    1  seq
......................................................................aAuauuuccggcugcacacagg................  6    1  seq
......................................................................auuauuuccggcugUacacagg................  3    1  seq
......................................................................auuGuuuccggcugcacacagg................  1    1  seq
......................................................................auuauuuccggcugcacacUgg................  3    1  seq
......................................................................Guuauuuccggcugcacacagg................  14   1  seq
```

```
                                      Star                          Mature
ugucgaaacgcuacaaaaagccauggcaggucuaggccugugugcagccggaaauaguucuauuaaauggaauuauuuccggcugcacacaggcuaagcaacgcuaaga
.......................................................................auuauuAccggcugcacacagg................	2	1	seq
.......................................................................auuauuuccggcugcUcacagg................	4	1	seq
.......................................................................auuauuuccggcCgcacacagg................	3	1	seq
.......................................................................auuauuuccggcugcacacaggU...............	22	1	seq
.......................................................................auuauuuccggcugcacacaggA...............	1	1	seq
.......................................................................auuauuuccggcugcacacaggc...............	2	0	seq
........................................................................uuauuuccggcugcacacagg................	7	0	seq
........................................................................uuauuuccggcugcacacagA................	1	1	seq
........................................................................uuauuuccggcugcacacaggc...............	10	0	seq
........................................................................uuauuuccggcugcacacaggU...............	4	1	seq
.........................................................................uauuuccggcugcacacagg................	1	0	seq
.........................................................................Aauuuccggcugcacacagg................	1	1	seq
.........................................................................uauuuccggcugcacacaggc...............	2	0	seq
.........................................................................uauuuccggcugcacacaggcA..............	1	1	seq
.........................................................................uauuuccggcugcacacaggcu..............	2	0	seq
```

miRBase precursor : novel-nve-miR-48-1
Total read count : 2176
novel-nve-miR-48-1_guide read count : 2175
novel-nve-miR-48-1_star read count : 1
remaining reads : 0

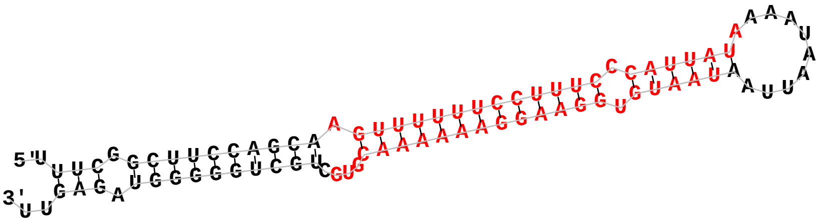

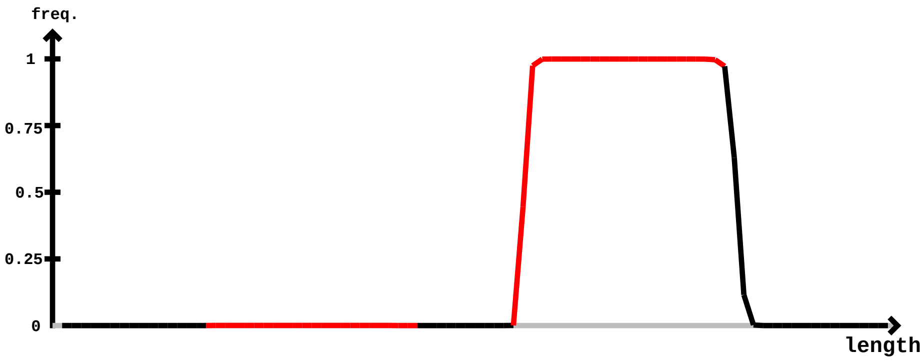


```
                                  novel-nve-miR-48-1_star
                                                              novel-nve-miR-48-1_guide
5'- uuucggcuuccagcaaguuuuuuccuuucccauuauaaaauaauuaauaauguggaaggaaaaaacgugcugcuggggguagaguu   -3'    exp
    .(((.((((((((((.(((((((((((((.((((((..........)))))).)))))))))))))....)))))))))).)))..    reads      mm         sample
    ...............aguuuuuuccuuucccauC....................................................    1          1          seq
    ..............................................auaauguggaaggaaaaaacgug.................    1          0          seq
    ...............................................uaauguggaaggaaaaaacg...................    1          0          seq
    ...............................................uaauguggaaggaaaaaacgG..................    4          1          seq
    ...............................................Gaauguggaaggaaaaaacgu..................    1          1          seq
    ...............................................uaauguggaaggaaaaaacgC..................    6          1          seq
    ...............................................uaauguggaaggaaaaGacgu..................    1          1          seq
    ...............................................uaauguggaaggaaaaaacgA..................    3          1          seq
    ...............................................uaauguggaaggaaaaaacgu..................    17         0          seq
    ...............................................uaauguggaaggaaaaaaUgug.................    1          1          seq
    ...............................................uaauguggaagAaaaaaacgug.................    1          1          seq
    ...............................................uaauguggaaggaaaaaGcgug.................    3          1          seq
    ...............................................uaauguggaaggaaaaaacguU.................    18         1          seq
    ...............................................uGauguggaaggaaaaaacgug.................    1          1          seq
    ...............................................uaauguggaUggaaaaaacgug.................    2          1          seq
    ...............................................uaauguggaaggaaaaaacAug.................    1          1          seq
    ...............................................Aaauguggaaggaaaaaacgug.................    9          1          seq
    ...............................................uaauguggaaggaaaaaacguC.................    1          1          seq
    ...............................................uaauguggaaAgaaaaaacgug.................    1          1          seq
    ...............................................uaauguggaaggGaaaaacgug.................    3          1          seq
    ...............................................uaauguggaGggaaaaaacgug.................    2          1          seq
    ...............................................uaGuguggaaggaaaaaacgug.................    3          1          seq
    ...............................................uaauguAgaaggaaaaaacgug.................    1          1          seq
    ...............................................uaauguggaaggaaGaaacgug.................    3          1          seq
    ...............................................uaauguggaaggaaaaGacgug.................    1          1          seq
    ...............................................uaauguggaaggaaaaaacgGg.................    2          1          seq
    ...............................................uaauguggaaggaaaaaacgug.................    498        0          seq
    ...............................................uaaCguggaaggaaaaaacgug.................    1          1          seq
    ...............................................uaauguggaaggaaaaaacguA.................    94         1          seq
    ...............................................uaauguggGaggaaaaaacgug.................    6          1          seq
    ...............................................uaauguggaaggaaaaaacgCg.................    9          1          seq
    ...............................................uaauguggaaggaaaGaacgug.................    2          1          seq
    ...............................................uaauguggaaggaGaaaacgug.................    3          1          seq
    ...............................................uaauguggaaggaaUaaacgug.................    1          1          seq
```

```
               novel-nve-miR-48-1_star
                                               novel-nve-miR-48-1_guide
uuucggcuuccagcaaguuuuuuccuuucccauuauaaaauaauuaauaauguggaaggaaaaaacgugcugcugggguagaguu

...............................................uaauguggaaggaaaUaacgug................    1     1     seq
...............................................uaauguggaaggaaaaaaAgugc...............    1     1     seq
...............................................uaauguggaaggaaaaaacguCc...............    1     1     seq
...............................................Aaauguggaaggaaaaaacgugc...............    1     1     seq
...............................................uaauguggaaggaaaaaacguUc...............    1     1     seq
...............................................uaauguggaaggaaaaaacgugA...............    7     1     seq
...............................................uaauguggaaggaaaaaacgCgc...............    1     1     seq
...............................................uaauguggaaggGaaaaacgugc...............    1     1     seq
...............................................uaaAguggaaggaaaaaacgugc...............    1     1     seq
...............................................uaauguggaaggaGaaaacgugc...............    1     1     seq
...............................................Caauguggaaggaaaaaacgugc...............    2     1     seq
...............................................uaauguggaaUgaaaaaacgugc...............    1     1     seq
...............................................uaauguggaaggaaaGaacgugc...............    2     1     seq
...............................................uUauguggaaggaaaaaacgugc...............    1     1     seq
...............................................uaGuguggaaggaaaaaacgugc...............    1     1     seq
...............................................uaauguggaaggaaaaaacgugG...............    3     1     seq
...............................................uGauguggaaggaaaaaacgugc...............    1     1     seq
...............................................uaauguggaaggaaaaaacgugU...............    79    1     seq
...............................................uaaCguggaaggaaaaaacgugc...............    2     1     seq
...............................................uaauguggaaggaaaaaacgugc...............    122   0     seq
...............................................uaaugAggaaggaaaaaacgugc...............    1     1     seq
...............................................uaauguggaaggaaaaaacgugAu..............    1     1     seq
...............................................uaauguggaaggaaaaaacgugcA..............    5     1     seq
...............................................uaauguggaaggaaaaaacgugcC..............    5     1     seq
...............................................uaauguggaaggaaaaaGcgugcu..............    2     1     seq
...............................................uaauguggaaggaaaaaacgugcu..............    22    0     seq
...............................................uaauguggaaggaaaaaacgugcuA.............    1     1     seq
...............................................uaauguggaaggaaaaaacgugcuC.............    1     1     seq
................................................aauguggaaggaaaaaac...................    1     0     seq
................................................aauguggaaggaaaaaacA..................    2     1     seq
................................................aauguggaaggaaaaaacg..................    2     0     seq
................................................aauguggaaggaaaaaacgu.................    11    0     seq
................................................aauguggaaggaaaaaacgC.................    6     1     seq
................................................aauguggaaggaaaaaacgG.................    2     1     seq
................................................aauguggaGggaaaaaacgu.................    1     1     seq
................................................aauguggaaggaaaaaaAgug................    1     1     seq
................................................aauguggaaggaaaaaacgCg................    1     1     seq
................................................aauguggaaggaaaaaacguA................    8     1     seq
................................................aauguggaaggaaaaaacguC................    2     1     seq
................................................aauguggaaggaaGaaacgug................    1     1     seq
................................................aauguggaaggaaaaaacgGg................    1     1     seq
................................................aauguggaaggaaaaaacgug................    46    0     seq
................................................aauguggaaggaaaGaacgug................    1     1     seq
................................................aauguggaaggaaaaaaUgug................    1     1     seq
................................................aauguggaaggaaaaaacguU................    2     1     seq
................................................aaugugUaaggaaaaaacgug................    1     1     seq
................................................aauguggaagUaaaaaacgugc...............    1     1     seq
................................................aauguggaaggaaaaaacgugG...............    4     1     seq
................................................aauguggaaggaaaaaacgugc...............    506   0     seq
................................................aaGguggaaggaaaaaacgugc...............    1     1     seq
................................................aauguggaaggaUaaaacgugc...............    1     1     seq
................................................aauguggaaggaaaaaGcgugc...............    1     1     seq
................................................aauguggaaggaaaaaacAugc...............    1     1     seq
................................................aauguggaaggaaaaaacgAgc...............    1     1     seq
................................................aauguggaaggaaaaaCcgugc...............    1     1     seq
................................................aauguggaaggGaaaaacgugc...............    2     1     seq
................................................aauguggaGggaaaaaacgugc...............    3     1     seq
................................................aauguggaaggaaaaaacgugU...............    288   1     seq
................................................aCuguggaaggaaaaaacgugc...............    1     1     seq
................................................Gauguggaaggaaaaaacgugc...............    3     1     seq
................................................aauguggaaggaaaaaacgugA...............    48    1     seq
................................................aaCguggaaggaaaaaacgugc...............    1     1     seq
................................................aauguggaaggaaaaaacgCgc...............    1     1     seq
................................................aauguggGaggaaaaaacgugc...............    3     1     seq
................................................aauguggaaggaaaaaacguAc...............    1     1     seq
................................................aauguggaagAaaaaaacgugc...............    2     1     seq
................................................aauguCgaaggaaaaaacgugc...............    2     1     seq
................................................aaugAggaaggaaaaaacgugc...............    4     1     seq
................................................aauguggaaggaaaGaacgugc...............    1     1     seq
```

novel-nve-miR-48-1_star

novel-nve-miR-48-1_guide

uuucggcuuccagcaaguuuuuuccuuucccauuauaaaauaauuaauaauguggaaggaaaaaacgugcugcuggggguagaguu

....aGuguggaaggaaaaaacgugc................	1	1	seq
....aauguggaaCgaaaaaacgugc................	1	1	seq
....aauguggaaggaaaaaacguCc................	1	1	seq
....aGuguggaaggaaaaaacgugcu...............	1	1	seq
....aauguggaaggaaaaaacgugcA...............	15	1	seq
....aauguAgaaggaaaaaacgugcu...............	1	1	seq
....aaAguggaaggaaaaaacgugcu...............	2	1	seq
....aauguggaaggaaaaaacgugGu...............	1	1	seq
....aauguggaaggaaaaaacgAgcu...............	1	1	seq
....aauguggaUggaaaaaacgugcu...............	1	1	seq
....aauguggaaggaaaaaacgugcC...............	30	1	seq
....aauguggGaggaaaaaacgugcu...............	2	1	seq
....aauguggaaggaaaaaacgugcu...............	113	0	seq
....aauguggaaggaaaaaacgugcG...............	5	1	seq
....aauguggaaggaaaaaacgCgcu...............	1	1	seq
....aauguggaaggaaaaaacgugAu...............	3	1	seq
....aauguggaaggaGaaaacgugcu...............	2	1	seq
....aauguggaagUaaaaaacgugcu...............	1	1	seq
....aaugugUaaggaaaaaacgugcu...............	1	1	seq
....aauguggaaggaaaaaacgugcuU..............	1	1	seq
....auguggaaggaaaaaacgu..................	1	0	seq
....auguggaaggaaaaaacgug.................	7	0	seq
....auguggaaggaaaaaacguA.................	1	1	seq
....auguggaaggaaaaaacguU.................	2	1	seq
....auguggaaggaaaaaacguC.................	1	1	seq
....auguggaaggaaaaaacgugA................	1	1	seq
....auguggaaggaaaaaacgGgc................	1	1	seq
....auguggaaggaaaaaacgugU................	2	1	seq
....aCguggaaggaaaaaacgugc................	1	1	seq
....auguggaaggaaaaaacgugc................	3	0	seq
....aAguggaaggaaaaaacgugc................	1	1	seq
....Cuguggaaggaaaaaacgugcu...............	1	1	seq
....auguggaaggaaaaaacgugcG...............	1	1	seq
....auguggaaggaaaaaacgugcu...............	21	0	seq
....auguggaaggaaaaaacgugcC...............	4	1	seq
....auguggaaggaaaaaacgugcA...............	2	1	seq
....auguggaagAaaaaaacgugcu...............	2	1	seq
....auguggaaggaaaaaacgugcuU..............	2	1	seq
....uguggaaggaaaaaacgug.................	1	0	seq

Provisional ID		novel-nve-miR-48-2
Score total	:	2264.8
Score for star read(s)	:	3.9
Score for read counts	:	2258.1
Score for mfe	:	1.8
Score for randfold	:	1.6
Score for cons. seed	:	-0.6
Total read count	:	4441
Mature read count	:	4402
Loop read count	:	0
Star read count	:	39

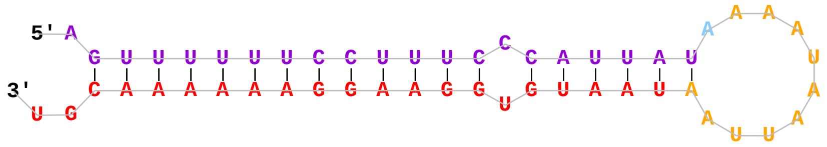

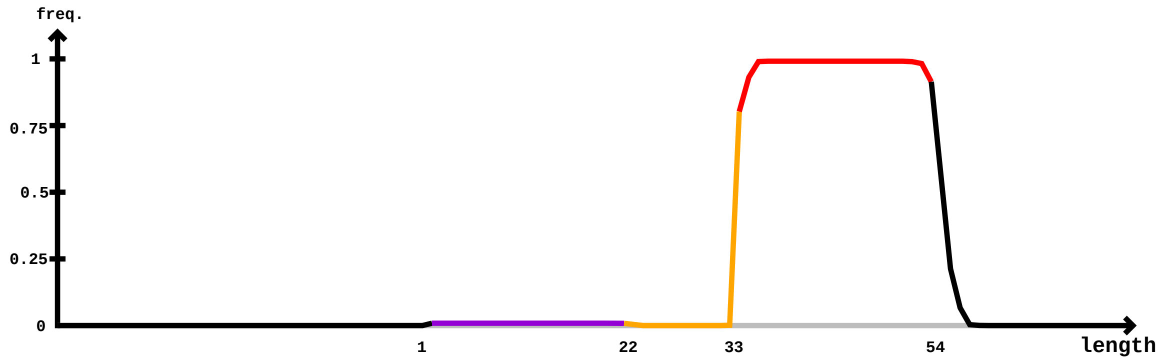


```
                                     Star                          Mature
5'- caacuggccguauucgccuuguuuucggcuuccagcaaguuuuuuccuuucccauuauaaaauaauuaauaauguggaaggaaaaaacgugcugcuggggguagaguuac  -3'   obs
    caacuggccguauucgccuuguuuucggcuuccagcaaguuuuuuccuuucccauuauaaaauaauuaauaauguggaaggaaaaaacgugcugcuggggguagaguuac         exp
    .(((.(((.......)))..)))(((.((((((((((.((((((((((((.((((((.........)))))).)))))))))))))....)))))))))).)))....   reads     mm     sample
    .....................................aguuuuuuccuuucccauC......................................................   1         1      seq
    .....................................aguuuuuuccuuucccauuG.....................................................   1         1      seq
    .....................................aguuuuuuccuuucccauuau....................................................   27        0      seq
    .....................................aguuuCuuccuuucccauuau....................................................   1         1      seq
    .....................................aguuuuuuccuuucccauuaC....................................................   5         1      seq
    .....................................aguuuuuuccuuucccaCuau....................................................   1         1      seq
    .....................................aCuuuuuuccuuucccauuau....................................................   1         1      seq
    .....................................aguuuuuuccuuucccauuaua...................................................   2         0      seq
    ....................................................................auaauguggaaggaaaaaac......................   1         0      seq
    ....................................................................auaauguggaaggaaaaaacU.....................   1         1      seq
    ....................................................................auaauguggaaggaaaaaacgu....................   1         0      seq
    ....................................................................auaauguggaaggaaaaaacguC...................   1         1      seq
    ....................................................................auaauguggaaggaaaaaacgugAu.................   1         1      seq
    .....................................................................uaauguggaaggaaaaaU.......................   1         1      seq
    .....................................................................uaauguggaaggaaaaaa.......................   5         0      seq
    .....................................................................uaauguggaaggaaaaaC.......................   1         1      seq
    .....................................................................uaauguggaaggaaaaaCc......................   1         1      seq
    .....................................................................uaauguggaaggaaaaaac......................   24        0      seq
    .....................................................................uaauguggaaCgaaaaaac......................   1         1      seq
    .....................................................................uaauguggaaggaaaaaaA......................   2         1      seq
    .....................................................................uaauguCgaaggaaaaaacg.....................   2         1      seq
    .....................................................................uaauguggaaggGaaaaacg.....................   1         1      seq
    .....................................................................uaauguggaaggaaaaaacU.....................   8         1      seq
    .....................................................................Caauguggaaggaaaaaacg.....................   1         1      seq
    .....................................................................uaaAguggaaggaaaaaacg.....................   2         1      seq
    .....................................................................uaauCuggaaggaaaaaacg.....................   1         1      seq
    .....................................................................uaauguggaaggaaaaaacC.....................   3         1      seq
    .....................................................................uaauguggaaggaaaaaacg.....................   227       0      seq
    .....................................................................Aaauguggaaggaaaaaacg.....................   3         1      seq
    .....................................................................uaaugGggaaggaaaaaacg.....................   2         1      seq
    .....................................................................uaauguggaaggaaaaaGcg.....................   1         1      seq
    .....................................................................uaauguggaaUgaaaaaacg.....................   1         1      seq
    .....................................................................uaauguggaaggaaaaGacg.....................   1         1      seq
```

Star **Mature**

caacuggccguauucgccuuguuuucggcuuccagcaaguuuuuuccuuucccauuauaaaauaauuaauaauguggaaggaaaaaacgugcugcuggggguagaguuac			
....uaauguggaaggaGaaaacg......................	1	1	seq
....uaauguggaaggaaaaaacA......................	30	1	seq
....uaauguggaagAaaaaaacg......................	1	1	seq
....uaauAuggaaggaaaaaacgu.....................	2	1	seq
....uaauguggaagAaaaaaacgu.....................	7	1	seq
....uaauguggaaggaaaaUacgu.....................	1	1	seq
....uaaugGggaaggaaaaaacgu.....................	1	1	seq
....uaauguggUaggaaaaaacgu.....................	1	1	seq
....uaGuguggaaggaaaaaacgu.....................	4	1	seq
....uaauguggaaggaaaaGacgu.....................	4	1	seq
....uaauguggaaggaaaaaacCu.....................	1	1	seq
....uaauguggaaggaaaGaacgu.....................	5	1	seq
....uaaugCggaaggaaaaaacgu.....................	4	1	seq
....uCauguggaaggaaaaaacgu.....................	1	1	seq
....uaauguggaaggaaaaaaAgu.....................	2	1	seq
....uaaugAggaaggaaaaaacgu.....................	6	1	seq
....Aaauguggaaggaaaaaacgu.....................	22	1	seq
....uaauguggaaggaaaaaacgG.....................	42	1	seq
....uaauguggaaggaaaaaGcgu.....................	4	1	seq
....uaauguggaagUaaaaaacgu.....................	2	1	seq
....uaauguggaaggaaUaaacgu.....................	3	1	seq
....uaauguggaaggaGaaaacgu.....................	3	1	seq
....uaauguggaaggGaaaaacgu.....................	5	1	seq
....uaauguggaaggaaaaaacAu.....................	7	1	seq
....uaauguggaaggaUaaaacgu.....................	2	1	seq
....uaauguggaaggaaaaaacgu.....................	1397	0	seq
....uaauUuggaaggaaaaaacgu.....................	2	1	seq
....uaauguggaaUgaaaaaacgu.....................	1	1	seq
....uaauguggaaggaaaaCacgu.....................	2	1	seq
....uaauguggaaggUaaaaacgu.....................	2	1	seq
....Caauguggaaggaaaaaacgu.....................	4	1	seq
....uGauguggaaggaaaaaacgu.....................	2	1	seq
....uaauguggaaggaaaaaacgA.....................	39	1	seq
....uaaCguggaaggaaaaaacgu.....................	5	1	seq
....uaaAguggaaggaaaaaacgu.....................	4	1	seq
....uaauguggaaggaaaaaCcgu.....................	4	1	seq
....uaauguggaGggaaaaaacgu.....................	8	1	seq
....uaauguggaaggaaaaaacUu.....................	1	1	seq
....uaauguCgaaggaaaaaacgu.....................	1	1	seq
....uaauguggGaggaaaaaacgu.....................	6	1	seq
....Gaauguggaaggaaaaaacgu.....................	4	1	seq
....uaaugugAaaggaaaaaacgu.....................	2	1	seq
....uaauguggaaggaaGaaacgu.....................	6	1	seq
....uaauguggaCggaaaaaacgu.....................	1	1	seq
....uaauguggaaggGaaaaacgug....................	3	1	seq
....Caauguggaaggaaaaaacgug....................	2	1	seq
....uaauguggaagAaaaaaacgug....................	6	1	seq
....uaaugAggaaggaaaaaacgug....................	1	1	seq
....uaauguggaaggaaaaaacUug....................	1	1	seq
....uaauguggUaggaaaaaacgug....................	1	1	seq
....uaauguggaaggaaaaaacAug....................	5	1	seq
....uaauguggaaggaaaaaacgAg....................	1	1	seq
....uaauCuggaaggaaaaaacgug....................	1	1	seq
....uaauguggaaAgaaaaaacgug....................	1	1	seq
....uaauguUgaaggaaaaaacgug....................	1	1	seq
....uaauguggaaggaaaaaacCug....................	1	1	seq
....Aaauguggaaggaaaaaacgug....................	11	1	seq
....uaaugugUaaggaaaaaacgug....................	1	1	seq
....uaauguggaaggaGaaaacgug....................	3	1	seq
....uaauguggaaggaaaaaCcgug....................	3	1	seq
....uaaCguggaaggaaaaaacgug....................	2	1	seq
....uaauguggaaggaaaaGacgug....................	3	1	seq
....uaauguggaaggaaaaaacguC....................	18	1	seq
....uaauguggaaggaaaaaacguU....................	74	1	seq
....uaauguggaaggaaaGaacgug....................	5	1	seq
....uGauguggaaggaaaaaacgug....................	3	1	seq
....uaauguggaaggaaaaaacgGg....................	6	1	seq
....uaauguggaaggaaaaaGcgug....................	3	1	seq
....uaaugCggaaggaaaaaacgug....................	1	1	seq
....uaauguggGaggaaaaaacgug....................	3	1	seq

Star **Mature**

caacuggccguauucgccuuguuuucggcuuccagcaaguuuuuuccuuucccauuauaaaauaauuaauaauguggaaggaaaaaacgugcugcugggggguagaguuac			
....uaauguggaaUgaaaaaacgug....................	1	1	seq
....uaauguAgaaggaaaaaacgug....................	1	1	seq
....uaauguggaaggaaaaaUcgug....................	1	1	seq
....uaauguggaaggaaaaaacgug....................	797	0	seq
....uaauguggaaggaaaaaacguA....................	122	1	seq
....uaauguggaGggaaaaaacgug....................	2	1	seq
....uaauguggaaggUaaaaacgug....................	1	1	seq
....uaauguggaaggaGaaaacgugc...................	1	1	seq
....uaauguggaaggaaaaaacgugU...................	124	1	seq
....uaauguAgaaggaaaaaacgugc...................	1	1	seq
....uaauguggaaggaaaaaacgugA...................	28	1	seq
....uaauguggGaggaaaaaacgugc...................	1	1	seq
....uaauguggaaggaaaaaacAugc...................	1	1	seq
....uaauguggaaggaaaaCacgugc...................	1	1	seq
....uaaugAggaaggaaaaaacgugc...................	1	1	seq
....uaauguggaaggaaaaaacgugG...................	7	1	seq
....uaauguggaaggaaaaaacgugc...................	224	0	seq
....uaauguggaaggUaaaaacgugc...................	1	1	seq
....uaauguggaGggaaaaaacgugc...................	1	1	seq
....uaauguggaaggGaaaaacgugc...................	1	1	seq
....uaauguggaaggaaaaaacguAc...................	1	1	seq
....uaauguggaaggaaaaaacgAgc...................	1	1	seq
....uaauguggUaggaaaaaacgugc...................	1	1	seq
....Caauguggaaggaaaaaacgugc...................	1	1	seq
....Aaauguggaaggaaaaaacgugc...................	2	1	seq
....uaaCguggaaggaaaaaacgugc...................	1	1	seq
....uaGuguggaaggaaaaaacgugc...................	1	1	seq
....uaauguggaagAaaaaaacgugc...................	1	1	seq
....uaauguggaaggaaaGaacgugc...................	1	1	seq
....uaauguggaaggaaaaaacgugcu..................	53	0	seq
....uaauguggaaggaaaaaacgugcG..................	4	1	seq
....uaaugAggaaggaaaaaacgugcu..................	1	1	seq
....uaauguggaaggaaaaaacgugcC..................	13	1	seq
....uaauguggaaggaaaaaacgugUu..................	3	1	seq
....uUauguggaaggaaaaaacgugcu..................	1	1	seq
....uaauguggaaggaaGaaacgugcu..................	1	1	seq
....uaauguggaaggaaaaaacgugAu..................	36	1	seq
....uaauguggaaggaaaaaacgugcA..................	11	1	seq
....uaauguggaaggaaaGaacgugcu..................	1	1	seq
....uaauguggaaggaaaaaacgugcuA.................	2	1	seq
....uaauguggaaggaaaaaacgugAug.................	1	1	seq
....uaauguggaaggaaaaaacgugcuU.................	4	1	seq
....uaauguggaaggaaaaaacgugcug.................	1	0	seq
....uaauguggaaggaaaaaacgugcuUc................	1	1	seq
....Uauguggaaggaaaaaac.......................	1	1	seq
....aauguggaaggaaaaaac.......................	2	0	seq
....aauguggaaggaaaaaacg......................	12	0	seq
....Gauguggaaggaaaaaacg......................	1	1	seq
....aauguggaaggaaaaaacA......................	2	1	seq
....aauguggaagAaaaaaacg......................	1	1	seq
....aauguggaaggaaaaaacgu.....................	89	0	seq
....aauguggaaggaaaaaGcgu.....................	1	1	seq
....aauguggaaggaaGaaacgu.....................	1	1	seq
....aGuguggaaggaaaaaacgu.....................	2	1	seq
....aauCuggaaggaaaaaacgu.....................	1	1	seq
....Uauguggaaggaaaaaacgu.....................	1	1	seq
....aauguggaaggaaaaaacgA.....................	5	1	seq
....aaugAggaaggaaaaaacgu.....................	1	1	seq
....aauguggaaggaaaaaacgG.....................	1	1	seq
....aauguggaGggaaaaaacgu.....................	1	1	seq
....aauguggaaggGaaaaacgu.....................	1	1	seq
....aUuguggaaggaaaaaacgu.....................	1	1	seq
....aauguggaUggaaaaaacgug....................	1	1	seq
....Uauguggaaggaaaaaacgug....................	1	1	seq
....aauguAgaaggaaaaaacgug....................	1	1	seq
....aauguggaaggaaaaaaAgug....................	1	1	seq
....aauguggaaggaaaaaacguU....................	3	1	seq
....aauguggaaggaaaaaacgug....................	131	0	seq
....aauguggGaggaaaaaacgug....................	2	1	seq
....aaugugUaaggaaaaaacgug....................	1	1	seq

Star Mature

```
caacuggccguauucgccuuguuuucggcuuccagcaaguuuuuuccuuucccauuauaaaauaauuaauaauguggaaggaaaaaacgugcugcugggguagaguuac
......................................................................aauguggaagAaaaaaacgug..................	2	1	seq
......................................................................aauguggaaggaaaaGacgug..................	2	1	seq
......................................................................aauguggaGggaaaaaacgug..................	1	1	seq
......................................................................aauguggaaggaaaaaacguA..................	9	1	seq
......................................................................aauguggaaggaaaaaacgugA.................	12	1	seq
......................................................................aauguggaaggaaaaaacgugU.................	41	1	seq
......................................................................aauguggaaggaaaGaacgugc.................	1	1	seq
......................................................................aauguAgaaggaaaaaacgugc.................	1	1	seq
......................................................................aauguggaaggaaaaaacgugc.................	131	0	seq
......................................................................aauguggaaggaaaaaGcgugc.................	1	1	seq
......................................................................aauguggaaggaaaaaacguCc.................	1	1	seq
......................................................................aauguggaaggaaGaaacgugc.................	1	1	seq
......................................................................aGuguggaaggaaaaaacgugc.................	1	1	seq
......................................................................aaugAggaaggaaaaaacgugc.................	1	1	seq
......................................................................aauguggaaggGaaaaacgugc.................	1	1	seq
......................................................................aauguggaaggaaaaaacgugG.................	3	1	seq
......................................................................aauguggaaggaaaaaCcgugc.................	2	1	seq
......................................................................aaAguggaaggaaaaaacgugc.................	1	1	seq
......................................................................aauguggaaggaaaaaacgugcC................	14	1	seq
......................................................................aaAguggaaggaaaaaacgugcu................	1	1	seq
......................................................................aauguggaaggaaaaaacgugcu................	46	0	seq
......................................................................aauguggaaggaaaaGacgugcu................	1	1	seq
......................................................................aauguggaaggaaaaaacgugAu................	4	1	seq
......................................................................aauguggaaggaaaaaacgugcG................	12	1	seq
......................................................................aaCguggaaggaaaaaacgugcu................	1	1	seq
......................................................................Gauguggaaggaaaaaacgugcu................	1	1	seq
......................................................................aauguggaagAaaaaaacgugcu................	1	1	seq
......................................................................aauguggaaggaaaaaacgugcA................	11	1	seq
......................................................................aauguggaaggaaaaaacgugcuA...............	1	1	seq
......................................................................aauguggaaggaaaaaacgugcuUc..............	1	1	seq
.......................................................................auguggaaggaaaaaacg....................	1	0	seq
.......................................................................auguggaaggaaaaGacgu...................	1	1	seq
.......................................................................auguggaaggaaGaaacgu...................	1	1	seq
.......................................................................auCuggaaggaaaaaacgu...................	1	1	seq
.......................................................................auguggaaggaaaGaacgu...................	2	1	seq
.......................................................................auguggaaggaaaaaacgu...................	29	0	seq
.......................................................................auguggaaggaaaaaacgA...................	2	1	seq
.......................................................................auguggaaggUaaaaacgug..................	1	1	seq
.......................................................................auguggaaggaaaaaacgug..................	81	0	seq
.......................................................................Guguggaaggaaaaaacgug..................	1	1	seq
.......................................................................auguggaaggaaGaaacgug..................	1	1	seq
.......................................................................auguggaaggaaaaaacCug..................	1	1	seq
.......................................................................augCggaaggaaaaaacgug..................	1	1	seq
.......................................................................auguggaaggaaaaaacguU..................	3	1	seq
.......................................................................auAuggaaggaaaaaacgug..................	1	1	seq
.......................................................................auguggaaggaaaaaacgGg..................	1	1	seq
.......................................................................augugCaaggaaaaaacgug..................	1	1	seq
.......................................................................auguggaaggaaaaaacguC..................	1	1	seq
.......................................................................auguggaaggaaaaaacguA..................	14	1	seq
.......................................................................auguggaaggaaaaGacgugc.................	1	1	seq
.......................................................................auguggaaggaaaaaacgugU.................	10	1	seq
.......................................................................auguggaaggaaaaaacgugc.................	33	0	seq
.......................................................................auguggaaggaaaaaacgugA.................	4	1	seq
.......................................................................auguggaaggaaaaaacgugG.................	1	1	seq
.......................................................................auguggaaggaaaaaacgugAu................	4	1	seq
.......................................................................auguggaaggaaaaaacgugcu................	36	0	seq
.......................................................................auguggaaggaaaaaacgugcA................	6	1	seq
.......................................................................auguggaaggaaaaaacgugcC................	11	1	seq
.......................................................................auguggaaggaaaaaacAugcu................	1	1	seq
.......................................................................auguggaaggaaaaaacgugcG................	4	1	seq
.......................................................................Guguggaaggaaaaaacgugcu................	1	1	seq
.......................................................................auguggaaggaaaaaacgugcuU...............	3	1	seq
.......................................................................auguggaaggaaaaaacgugcuA...............	1	1	seq
........................................................................uguggaaggaaaaaacgug..................	3	0	seq
........................................................................uguggUaggaaaaaacgug..................	1	1	seq
........................................................................uguggaaggaaaaaacgugU.................	1	1	seq
........................................................................uguggaaggaaaaaacgugcu................	1	0	seq
```

miRBase precursor : novel-nve-miR-49
Total read count : 4251
novel-nve-miR-49_guide read count : 4035
novel-nve-miR-49_star read count : 103
remaining reads : 113

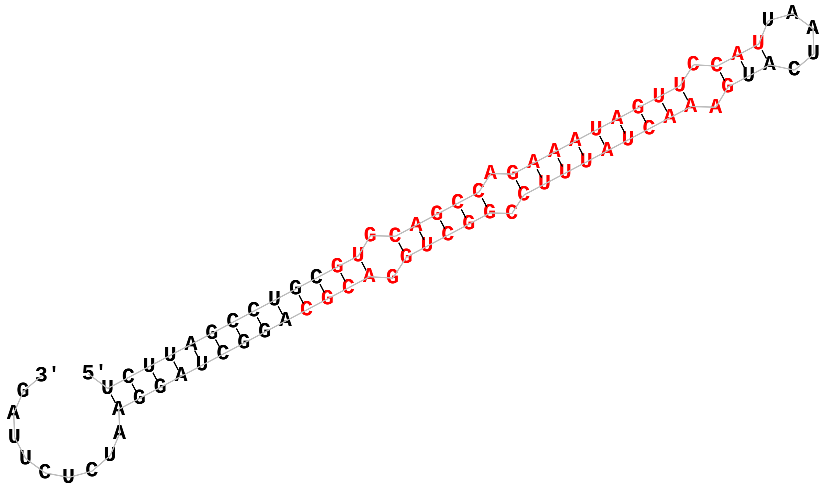

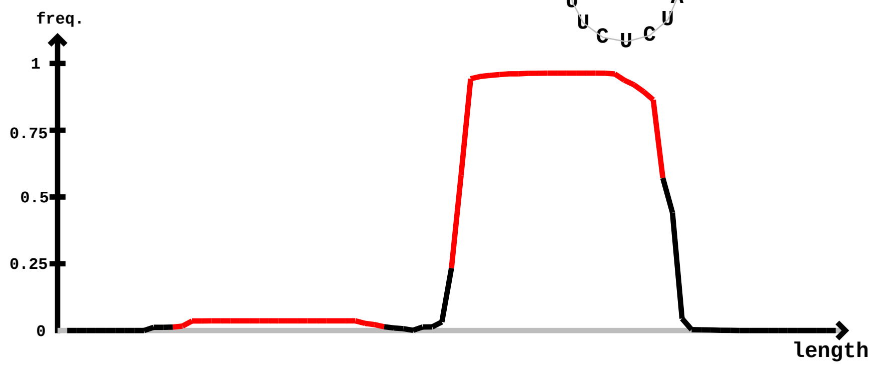

novel-nve-miR-49_star

novel-nve-miR-49_guide

```
5'- ucuuagccugcgugcagccagaaauaguuccauuaaucaugaaacuauuuccggcuggacgcaggcuaggaaucucuuag  -3'    exp
    ((((((((((((.(((((.(((((((((.(((.....))).))))))))).))))).))))))))))))...........    reads      mm         sample
    ........ugcgugcagccGgaaauaguuc..................................................    40         1          seq
    ........ugcgugcagccGgaaauaguucc.................................................    11         1          seq
    ..........cgugcagccGgaaauaguucca................................................    3          1          seq
    ..........cgugcagccGgaaauaguuccau...............................................    1          1          seq
    ...........gugcagccGgaaauaguucc.................................................    5          1          seq
    ...........gugcagccGgaaauaguucca................................................    4          1          seq
    ...........gugcagccagaaauaguuccU................................................    1          1          seq
    ...........gugcagccGgaaauaguuccau...............................................    4          1          seq
    ...........gugcagccGgaaauaguuccauu..............................................    1          1          seq
    ............ugcagccGgaaauaguucc.................................................    4          1          seq
    ............ugcagccGgaaauaguucca................................................    26         1          seq
    ............ugcagccagGaauaguucca................................................    1          1          seq
    ............ugcagccGgaaauaguuccau...............................................    14         1          seq
    ............ugcagccGgaaauaguuccauu..............................................    11         1          seq
    ............ugcagccGgaaauaguuccauua.............................................    26         1          seq
    ..............cagccGgaaauaguuccauua.............................................    2          1          seq
    ................................uuaaucaugaaacuauuuccggcuggacg...................    1          0          seq
    ...................................aucaugaaacuauuuccggcu........................    2          0          seq
    ...................................aucaugaaacuauuuccggcuggacg...................    1          0          seq
    ....................................ucaugaaacuauuuccggU.........................    1          1          seq
    ....................................ucaugaaacuauuuccggcA........................    2          1          seq
    ....................................ucaugaaacuauuuccggcu........................    6          0          seq
    ....................................ucaugaaacuauuuccggcC........................    1          1          seq
    ....................................ucaugaaacuauuuccggcug.......................    9          0          seq
    ....................................ucaugaaaUuauuuccggcug.......................    1          1          seq
    ....................................ucaugaaacuauuuccggcuggaU....................    2          1          seq
    ....................................ucaugaaacuauuuccggcuggac....................    4          0          seq
    ....................................ucaugaaacuauuuccggcuggacg...................    17         0          seq
    ....................................ucaugaaacuauuuccggcuggacU...................    6          1          seq
    ....................................ucaugaaacuauuuccggcuggacA...................    3          1          seq
    .....................................caugaaacuauuuccggcuggacU...................    2          1          seq
    ......................................augaaacuauuuccggcu........................    1          0          seq
    ......................................augaaacuauuuccggcug.......................    9          0          seq
    ......................................augaaacuauuucUggcug.......................    1          1          seq
```

```
           novel-nve-miR-49_star
                                 novel-nve-miR-49_guide
ucuuagccugcgugcagccagaaauaguuccauuaaucaugaaacuauuuccggcuggacgcaggcuaggaaucucuuag

......................................augaaacuauuuccggcuA.......................	3	1	seq
......................................augaaacuGuuuccggcug.......................	1	1	seq
......................................augaaacuauuuccggcGg.......................	1	1	seq
......................................augaaacuauuuccggcugg......................	4	0	seq
......................................augaaacuGuuuccggcugg......................	1	1	seq
......................................augaaacuauuuccggcugU......................	1	1	seq
......................................augaaacuaCuuccggcugg......................	1	1	seq
......................................augaaacuauuuccggcugga.....................	4	0	seq
......................................augaaacuauuuccggcuggG.....................	1	1	seq
......................................augaaacuauuuccggcuggaU....................	2	1	seq
......................................augaaacuauuuccggcuggac....................	3	0	seq
......................................augaaacuauuuccggcuggacA...................	5	1	seq
......................................augaaacuauuuccggcuggacg...................	17	0	seq
......................................augaaacuauuuccggcuggacU...................	20	1	seq
......................................augaaacuauuuccggcuggacC...................	2	1	seq
......................................augaaacuauuAccggcuggacg...................	1	1	seq
.......................................ugaaacuauuuccggcuA.......................	4	1	seq
.......................................ugaaacuauuuccggcug.......................	63	0	seq
.......................................ugaaacuauuuAcggcug.......................	1	1	seq
.......................................ugaaacuauuuUcggcug.......................	4	1	seq
.......................................ugaaacuauuuccggcuU.......................	1	1	seq
.......................................ugaaacuCuuuccggcug.......................	1	1	seq
.......................................ugaaacuauuuccggcuC.......................	1	1	seq
.......................................ugaaacuauuuccAgcugg......................	1	1	seq
.......................................ugaaacuauuuccggcugA......................	7	1	seq
.......................................ugaaacuauuuUcggcugg......................	1	1	seq
.......................................ugaGacuauuuccggcugg......................	1	1	seq
.......................................ugaaGcuauuuccggcugg......................	1	1	seq
.......................................Agaaacuauuuccggcugg......................	4	1	seq
.......................................ugaaacuauuuccggcugU......................	3	1	seq
.......................................ugaaacuauuuccggcuAg......................	1	1	seq
.......................................ugaaacuauuuccggcugg......................	39	0	seq
.......................................ugaaacuauuuccggcugUa.....................	1	1	seq
.......................................ugaaacuauuuUcggcugga.....................	1	1	seq
.......................................ugaaCcuauuuccggcugga.....................	1	1	seq
.......................................ugaaacuauuCccggcugga.....................	1	1	seq
.......................................ugaaacuauuuccggcuggG.....................	4	1	seq
.......................................ugaaacuauuuccAgcugga.....................	1	1	seq
.......................................Agaaacuauuuccggcugga.....................	3	1	seq
.......................................ugaaacuauuuccggcugga.....................	66	0	seq
.......................................ugaaacuauuuccgUcuggac....................	1	1	seq
.......................................ugaaacuauuuUcggcuggac....................	1	1	seq
.......................................ugaaUcuauuuccggcuggac....................	1	1	seq
.......................................ugaaacuauuuccggcuggaA....................	1	1	seq
.......................................Agaaacuauuuccggcuggac....................	1	1	seq
.......................................ugaaacuauuuccggcAggac....................	1	1	seq
.......................................ugaaacuauuuccggcuggac....................	31	0	seq
.......................................ugaaacuauuuccggcuggaU....................	28	1	seq
.......................................ugaaacuauuuccggcuggacA...................	49	1	seq
.......................................ugaaacuauuCccggcuggacg...................	1	1	seq
.......................................ugaaaUuauuuccggcuggacg...................	3	1	seq
.......................................ugaaacuauuucAggcuggacg...................	1	1	seq
.......................................Cgaaacuauuuccggcuggacg...................	2	1	seq
.......................................ugaaacuauuuccggcuggacg...................	274	0	seq
.......................................ugaaacuauuuccggcuAgacg...................	1	1	seq
.......................................ugaaacuauuuccggcuggaUg...................	1	1	seq
.......................................uAaaacuauuuccggcuggacg...................	1	1	seq
.......................................ugaaacCauuuccggcuggacg...................	1	1	seq
.......................................ugaaGcuauuuccggcuggacg...................	2	1	seq
.......................................ugaaacuauuuccggcuggacU...................	107	1	seq
.......................................ugaaacuauuuUcggcuggacg...................	2	1	seq
.......................................ugaaacuauuuAcggcuggacg...................	1	1	seq
.......................................ugaaacuauuuccggcuggacC...................	15	1	seq
.......................................ugCaacuauuuccggcuggacg...................	1	1	seq
.......................................ugaaacuauCuccggcuggacg...................	2	1	seq
.......................................uCaaacuauuuccggcuggacg...................	2	1	seq
.......................................ugaaacuauuuccggcuggaAg...................	1	1	seq
.......................................ugaaacuauAuccggcuggacg...................	1	1	seq
.......................................Agaaacuauuuccggcuggacg...................	3	1	seq
```

```
          novel-nve-miR-49_star
                              novel-nve-miR-49_guide
ucuuagccugcgugcagccagaaauaguuccauuaaucaugaaacuauuuccggcuggacgcaggcuaggaaucucuuag

.......................................ugaaacuauuuccggcuggacgU..................    41     1     seq
.......................................ugaaacuauuuccggcuggacgA..................    13     1     seq
.......................................ugaaacuauuuccggcuggacgc..................    5      0     seq
.......................................Agaaacuauuuccggcuggacgc..................    1      1     seq
.......................................ugaaacuauuuccggcuggacgUa.................    2      1     seq
.......................................ugaaacuauuuccggcuggacgcG.................    2      1     seq
.......................................ugaaacuauuuccggcuggacgcC.................    7      1     seq
.......................................ugaaacuauuuccggcuggacgcU.................    34     1     seq
.......................................ugaaacuauuuccggcuggacgca.................    4      0     seq
.......................................ugaaacuauuuccggcuggacgAa.................    2      1     seq
........................................gaaacuauuuccggcugg......................    6      0     seq
........................................gaaacuauuuccggcugA......................    2      1     seq
........................................gaaacuauuuccggcugga.....................    19     0     seq
........................................gaaacuauuucAggcugga.....................    1      1     seq
........................................gGaacuauuuccggcugga.....................    3      1     seq
........................................gaaacuauuuccggcuggac....................    20     0     seq
........................................gaaacuauuuccggcuggaU....................    6      1     seq
........................................Caaacuauuuccggcuggacg...................    1      1     seq
........................................gaaacuauuuccggcuggGcg...................    1      1     seq
........................................gaaacuauuuccggcuggacU...................    177    1     seq
........................................gaaacAauuuccggcuggacg...................    1      1     seq
........................................gaaacuauuucUggcuggacg...................    1      1     seq
........................................gaaGcuauuuccggcuggacg...................    2      1     seq
........................................gaaacCauuuccggcuggacg...................    1      1     seq
........................................gaaacuauuuccggcuggacA...................    66     1     seq
........................................gaaacuauuuccggcuggacg...................    165    0     seq
........................................gaaacuauuuUcggcuggacg...................    1      1     seq
........................................gaaacuauuAccggcuggacg...................    1      1     seq
........................................gGaacuauuuccggcuggacg...................    2      1     seq
........................................gaaacuauuuccggcAggacg...................    1      1     seq
........................................gaaacuaCuuccggcuggacg...................    1      1     seq
........................................Uaaacuauuuccggcuggacg...................    1      1     seq
........................................gaaacuGuuuccggcuggacg...................    2      1     seq
........................................gaaacuauuuccggcuggacC...................    47     1     seq
........................................gaaacuauAuccggcuggacg...................    1      1     seq
........................................gaaacuauuuccgAcuggacg...................    1      1     seq
........................................gaaacuauuuccggcuggacgG..................    4      1     seq
........................................gaaacuauuuccggcuggacUc..................    1      1     seq
........................................gaaacuauuuccggcCggacgc..................    1      1     seq
........................................gaaacuauuuccggcuggacgA..................    14     1     seq
........................................gaaacuauuuccggcuggacgU..................    51     1     seq
........................................gaaacuauuuccggcuggacgc..................    89     0     seq
........................................gaaacGauuuccggcuggacgc..................    1      1     seq
........................................gaaacuauuuccggcuggCcgc..................    1      1     seq
........................................gaaacCauuuccggcuggacgc..................    1      1     seq
........................................gaaacuauuuccggcuggacgcC.................    93     1     seq
........................................gaaacuauuuccggcuggacgAa.................    1      1     seq
........................................gaGacuauuuccggcuggacgca.................    2      1     seq
........................................gaaacuauuuccggcuggacgca.................    79     0     seq
........................................gaaacuauuuccggcuggacgcU.................    562    1     seq
........................................gaaacuauuuccAgcuggacgca.................    1      1     seq
........................................gaaUcuauuuccggcuggacgca.................    1      1     seq
........................................gGaacuauuuccggcuggacgca.................    1      1     seq
........................................gaaacuauuuccggcuggGcgca.................    2      1     seq
........................................gaaacuauuucUggcuggacgca.................    1      1     seq
........................................gaaacuauuuccggcCggacgca.................    1      1     seq
........................................gaaacCauuuccggcuggacgca.................    1      1     seq
........................................gaaacuauuuccggcuggacgcG.................    24     1     seq
........................................gaaacuauuuccggcuggacgcaC................    4      1     seq
........................................gaaacuauuuccggcuggacgcag................    1      0     seq
........................................gaaacuauuuccggcuggacgcaA................    1      1     seq
........................................gaaacuauuuccggcuggacgcaU................    7      1     seq
........................................gaaacuauuuccggcuggacgcUg................    2      1     seq
.........................................aaacuauuuccggcugga.....................    3      0     seq
.........................................aaacuauuuccggcuggaA....................    1      1     seq
.........................................aaacuauuCccggcuggac....................    2      1     seq
.........................................aaacuauuuccggcuggac....................    20     0     seq
.........................................aaacuauuuccggcuggaU....................    5      1     seq
.........................................aaacuauuuccggcuggGcg...................    2      1     seq
```

```
           novel-nve-miR-49_star
                                        novel-nve-miR-49_guide
ucuuagccugcgugcagccagaaauaguuccauuaaucaugaaacuauuuccggcuggacgcaggcuaggaaucucuuag

.........................................aaacuauuuccggcuggacg...................    154    0    seq
.........................................aaacuauuuccggcCggacg...................    1      1    seq
.........................................aaacuauuuccggcuggacA...................    34     1    seq
.........................................aaacAauuuccggcuggacg...................    1      1    seq
.........................................aaacuauuuccUgcuggacg...................    2      1    seq
.........................................aaacuauuucAggcuggacg...................    1      1    seq
.........................................aaacuauuuccggcuggacU...................    9      1    seq
.........................................aaacuauuuAcggcuggacg...................    1      1    seq
.........................................aaaAuauuuccggcuggacg...................    1      1    seq
.........................................aaacuauuucUggcuggacg...................    1      1    seq
.........................................aaaUuauuuccggcuggacg...................    2      1    seq
.........................................aaacuauuuccggcuggacC...................    2      1    seq
.........................................aaGcuauuuccggcuggacg...................    1      1    seq
.........................................aaacuauuuccgAcuggacg...................    1      1    seq
.........................................aaacuauuuccggcuggacUc..................    1      1    seq
.........................................aaacuauuuccggcCggacgc..................    2      1    seq
.........................................aaacuauuuccAgcuggacgc..................    1      1    seq
.........................................aaaUuauuuccggcuggacgc..................    2      1    seq
.........................................aaacuauuuccggcuggacgA..................    16     1    seq
.........................................aaacuauuuccggAuggacgc..................    2      1    seq
.........................................Gaacuauuuccggcuggacgc..................    1      1    seq
.........................................aaacuauuuccggcuggacgG..................    1      1    seq
.........................................aaacuauuuccggcuggGcgc..................    1      1    seq
.........................................aaacCauuuccggcuggacgc..................    1      1    seq
.........................................Caacuauuuccggcuggacgc..................    1      1    seq
.........................................aaacuGuuuccggcuggacgc..................    1      1    seq
.........................................aaacuauuuccggcuggacAc..................    1      1    seq
.........................................aaacuaCuuccggcuggacgc..................    1      1    seq
.........................................aaacuauuuccggcuAgacgc..................    1      1    seq
.........................................aaacuauuuccggcuggacgU..................    75     1    seq
.........................................aaacuauuuAcggcuggacgc..................    1      1    seq
.........................................aaacAauuuccggcuggacgc..................    1      1    seq
.........................................aaacuauuuccggcuggacgc..................    193    0    seq
.........................................aaacuauuuccggcuggacgcC.................    108    1    seq
.........................................aaacuauuuccggcuggacgUa.................    1      1    seq
.........................................aaacuauuCccggcuggacgca.................    1      1    seq
.........................................aaacuauuuccggcuggacgcG.................    19     1    seq
.........................................aaacuauuuccggcuggacgca.................    106    0    seq
.........................................aaacuauuuccggUuggacgca.................    2      1    seq
.........................................aaaUuauuuccggcuggacgca.................    1      1    seq
.........................................aaacuauuucUggcuggacgca.................    1      1    seq
.........................................aGacuauuuccggcuggacgca.................    1      1    seq
.........................................aaacuauuuccggcuggacgcU.................    593    1    seq
.........................................aaacuauuuccUgcuggacgca.................    1      1    seq
.........................................aaacuauuAccggcuggacgca.................    1      1    seq
.........................................aaacuauuuccggcuggacAca.................    1      1    seq
.........................................aaacuauuuccggcuggaAgca.................    1      1    seq
.........................................aaacuaCuuccggcuggacgca.................    1      1    seq
.........................................aaacuauuuccggcuggacgcUg................    4      1    seq
.........................................aaacuauuuccggcuggacgcaA................    8      1    seq
.........................................aaacuauuuccggcuggacgcaC................    21     1    seq
.........................................aaacuauuuccggcuggacgcaU................    117    1    seq
.........................................aaacuauuuccggcuggacgcaUg...............    2      1    seq
..........................................aacuauuuccggcuggacg...................    3      0    seq
..........................................aacuauuuccggcuggacA...................    1      1    seq
..........................................aacuauuuccggcugCacg...................    5      1    seq
..........................................aacuauuuccggcugCacgc..................    11     1    seq
..........................................aacuauuuccggcuggacgc..................    2      0    seq
..........................................aacuauuuccggcuggacgU..................    2      1    seq
..........................................aacuauuuccggcuggacgA..................    1      1    seq
..........................................aacuauuuccggcuggacgcG.................    1      1    seq
..........................................aacuauuuccggcugCacgca.................    1      1    seq
..........................................aacuauuuccggcuggacgca.................    2      0    seq
..........................................aacuauuuccggcuggacgcU.................    6      1    seq
..........................................aacuauuuccggcuggacgcaU................    1      1    seq
...........................................acuauuuccggcugCacg...................    3      1    seq
...........................................acuauuuccggcuggacC...................    1      1    seq
...........................................acuauuuccggcugCacgc..................    4      1    seq
...........................................acuauuuccggcugUacgc..................    1      1    seq
```

```
          novel-nve-miR-49_star
                             novel-nve-miR-49_guide
ucuuagccugcgugcagccagaaauaguuccauuaaucaugaaacuauuuccggcuggacgcaggcuaggaaucucuuag

...........................................acuauuuccggcuggacgc..................	1	0	seq
...........................................acuauuuccggcugCacgca.................	7	1	seq
............................................cuauuuccggcuggacgc..................	2	0	seq
............................................cuauuuccggcuggacgcU.................	2	1	seq
............................................cuauuuccggcuggacgcC.................	1	1	seq
............................................cuauuuccggcuggacgcG.................	1	1	seq
............................................cuauuuccggcuggacgcaC................	1	1	seq
............................................cuauuuccggcugCacgcag................	1	1	seq
............................................cuauuuccggcuggacgcaU................	4	1	seq
............................................cuauuuccggcugCacgcagg...............	1	1	seq
.............................................uauuuccggcugCacgca.................	4	1	seq
.............................................uauuuccggcuggacgcU.................	3	1	seq
.............................................uauuuccggcuggacgcC.................	1	1	seq
.............................................uauuuccggcugCacgcag................	4	1	seq
...............................................uuuccggcugCacgcaggc..............	2	1	seq
...............................................uAuccggcuggacgcaggcu.............	1	1	seq
...............................................uuuccggcugCacgcaggcu.............	4	1	seq
...............................................uuuccggcugCacgcaggcua............	1	1	seq
................................................uuccggcuggacgcaggcuaU...........	1	1	seq
.................................................uccggcuggacgcaggcuaA...........	2	1	seq
```

Provisional ID		novel-nve-miR-51
Score total	:	45.5
Score for star read(s)	:	3.9
Score for read counts	:	37.3
Score for mfe	:	-0.3
Score for randfold	:	1.6
Score for cons. seed	:	3
Total read count	:	85
Mature read count	:	82
Loop read count	:	0
Star read count	:	3

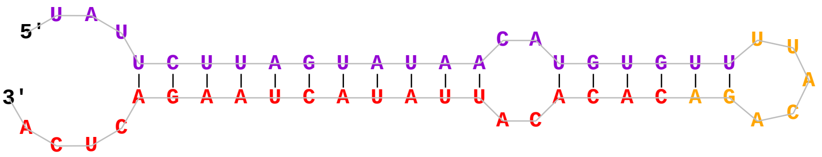

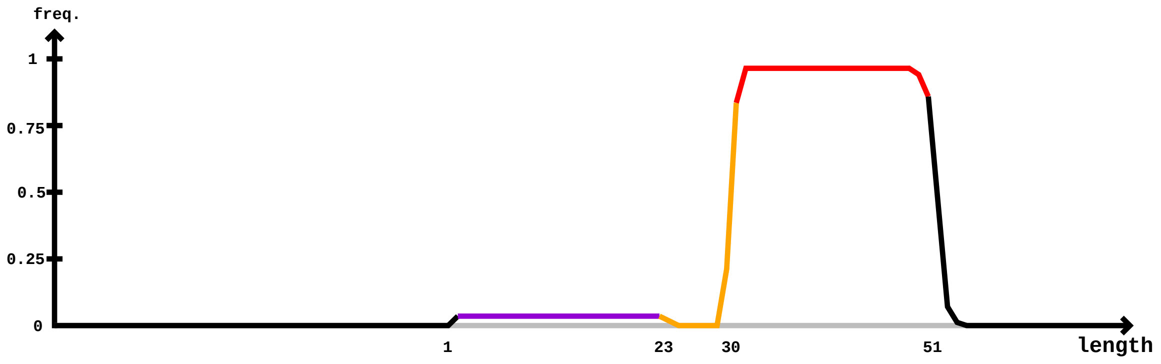

Star Mature

```
5'- gugugcuuuuaaaguccacauuuauaauuaagucuuacaauauucuuaguauaacauguguuuuacagacacacauuauacuaagacucaaaauggcucaauuugaaggu  -3'  obs
    gugugcuuuuaaaguccacauuuauaauuaagucuuacaauauucuuaguauaacauguguuuuacagacacacauuauacuaagacucaaaauggcucaauuugaaggu       exp
    (((..((.....))..))).((((.((((.((((.........(((((((((((..((((((.....))))))..)))))))))))........)))).))))))))...   reads   mm   sample
    ........................................uauucuuaguauaacauguguu................................................   3       0    seq
    ....................................................................acacacauuauacuaagacG......................   1       1    seq
    ....................................................................acacacauuauacuaagacuA.....................   1       1    seq
    ....................................................................acacacauuauacuaagacuc.....................   3       0    seq
    ....................................................................acacacauuauacuaagacucC....................   1       1    seq
    ....................................................................acacacauuauacuaagacucU....................   3       1    seq
    ....................................................................acacacauuauacuaagacucG....................   4       1    seq
    ....................................................................acacacauuauacuaagacuca....................   1       0    seq
    ....................................................................acacacauuauacuaagacucaG...................   1       1    seq
    ....................................................................acacacauuauacuaagacucaa...................   3       0    seq
    .....................................................................cacacauuauacuaagacu......................   1       0    seq
    .....................................................................cacacauuauacuaagacuc.....................   2       0    seq
    .....................................................................cacacauuauacuaagacuU.....................   1       1    seq
    .....................................................................Uacacauuauacuaagacuca....................   1       1    seq
    .....................................................................cacacauuauacuaagacucG....................   15      1    seq
    .....................................................................cacacauuauacuaagacucC....................   2       1    seq
    .....................................................................cacacauuauacuaagacucU....................   7       1    seq
    .....................................................................cacacauuauacuaagacuca....................   12      0    seq
    .....................................................................cacacauuauacuaagacucaU...................   4       1    seq
    .....................................................................cacacauuauacuaagacucaG...................   1       1    seq
    .....................................................................cacacauuauacuaagacucaa...................   5       0    seq
    .....................................................................caUacauuauacuaagacucaa...................   1       1    seq
    .....................................................................cacacauuauacuaagacucUa...................   1       1    seq
    ......................................................................acacauuauacuaagacucG....................   1       1    seq
    ......................................................................acacauuauacuaagacuca....................   2       0    seq
    ......................................................................acacauuauacuaagacucaa...................   1       0    seq
    ......................................................................acacauuauacuaagacucaG...................   1       1    seq
    ......................................................................acacauuauacuaagacucaaG..................   1       1    seq
    ......................................................................acacauuauacuaagacucaaU..................   2       1    seq
    ......................................................................acacauuauacuaagacucaaa..................   2       0    seq
    ......................................................................acacauuauacuaagacucaaaU.................   1       1    seq
```

miRBase precursor : novel-nve-miR-53
Total read count : 230
novel-nve-miR-53_guide read count : 218
novel-nve-miR-53_star read count : 12
remaining reads : 0

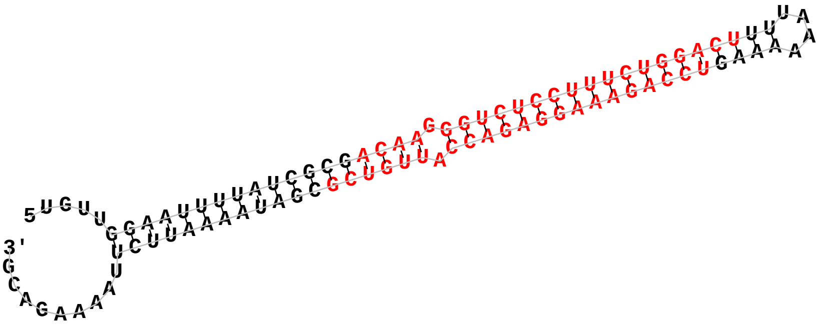

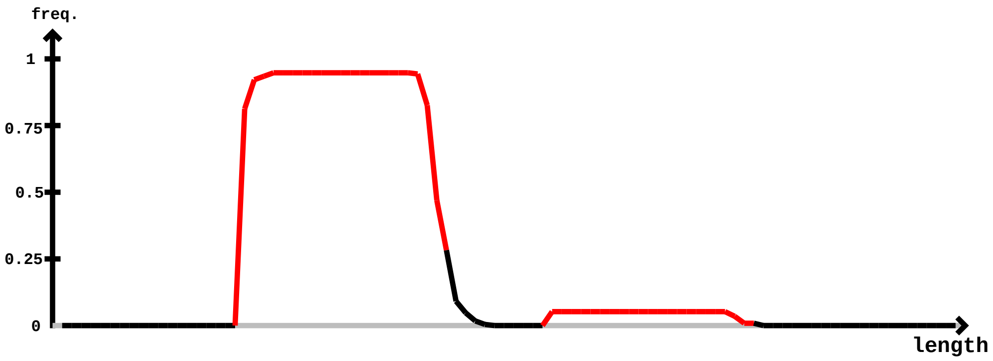


```
                                                    novel-nve-miR-53_star
                      novel-nve-miR-53_guide
5'- uguuggaauuuuaucgcgacaagggucuccuuucuggacuuuuaaaaaaguccagaaaggagaccauugucgcgauaaaaauucuuaaaagacg   -3'   exp
    .(((((((((((((((((((((.(((((((((((((((((((....))))))))))))))))))).))))))))))))))))))).....))).   reads   mm   sample
    ..................acaagggucuccuuucug..........................................................   1   0   seq
    ..................acaagggucuccuuucugA.........................................................   2   1   seq
    ..................acaagggucuccuuucugU.........................................................   2   1   seq
    ..................acaagggucuccuuucugg.........................................................   23   0   seq
    ..................acaagggucuccuuucCgga........................................................   1   1   seq
    ..................acaagggucuccuuucuggU........................................................   1   1   seq
    ..................acaagggucCccuuucugga........................................................   1   1   seq
    ..................acaagggucuccuuucugga........................................................   59   0   seq
    ..................Ccaagggucuccuuucugga........................................................   1   1   seq
    ..................acaagggucuUcuuucugga........................................................   1   1   seq
    ..................acaagggucuccCuucugga........................................................   2   1   seq
    ..................acaagggCcuccuuucugga........................................................   1   1   seq
    ..................acaaggguUuccuuucugga........................................................   1   1   seq
    ..................acaagggucuccuuucuggG........................................................   2   1   seq
    ..................acaagggucuccuuucuggaA.......................................................   2   1   seq
    ..................aUaagggucuccuuucuggac.......................................................   1   1   seq
    ..................acaagggucuccuuucuggaU.......................................................   12   1   seq
    ..................acaagggucuccuuucuggac.......................................................   23   0   seq
    ..................acaagggucAccuuucuggac.......................................................   1   1   seq
    ..................acaagggucuccuuucuggacC......................................................   4   1   seq
    ..................acaagggucuccuuucuggacA......................................................   2   1   seq
    ..................acaagggucuccuuucuggacG......................................................   1   1   seq
    ..................acaaggguUuccuuucuggacu......................................................   1   1   seq
    ..................acaagggucuccuGucuggacu......................................................   1   1   seq
    ..................acaagggucuccuuucuggaAu......................................................   1   1   seq
    ..................acaagggucuccuuucuggacu......................................................   26   0   seq
    ..................acaagggucuccuuucuggacuA.....................................................   4   1   seq
    ..................acaagggucuccuuucuggaUuu.....................................................   2   1   seq
    ..................acaagggucuccuuucuggacuAu....................................................   3   1   seq
    ..................acaagggucuccuuucuggaUuuu....................................................   1   1   seq
    ..................acaagggucuccuuucuggacuuu....................................................   1   0   seq
    ..................acaagggucuccuuucuggacuuAu...................................................   1   1   seq
    ..................acaagggucuccuuucuggacuuuu...................................................   2   0   seq
    ...................caagggucuccuuucuggG........................................................   1   1   seq
```

```
                                                  novel-nve-miR-53_star
                  novel-nve-miR-53_guide
uguuggaauuuuaucgcgacaagggucuccuuucuggacuuuuaaaaaaguccagaaaggagaccauugucgcgauaaaauucuuaaaagacg

...................caagggucuccuuucugga.......................................................   9   0   seq
...................caagggucuccuuucuggaU......................................................   1   1   seq
...................caagggucuccuuucuggac......................................................   2   0   seq
...................caagggucuccuuucuggacu.....................................................   6   0   seq
...................caagggucuccuuucuCgacu.....................................................   1   1   seq
...................caagggucuccuuucuggaUu.....................................................   1   1   seq
...................caagggucuccuuucuggacuu....................................................   3   0   seq
...................caagggucuccuuucuggacuuu...................................................   1   0   seq
....................aagggucuccuuucugga.......................................................   2   0   seq
....................aagggucuccuuucuggacuuC...................................................   1   1   seq
.....................agggucuccuuucuggaU......................................................   1   1   seq
.....................agggucuccuuucuggacuu....................................................   1   0   seq
.....................agggucuccuuucuggacuuuuU.................................................   1   1   seq
..................................................uccagaaaggagaccauug........................   4   0   seq
..................................................uccagaaaggagaccauugG.......................   1   1   seq
..................................................uccagaaaggagGccauugu.......................   1   1   seq
..................................................uccagaaaggagaccauugu.......................   3   0   seq
..................................................uccagaaaggagaccauugC.......................   1   1   seq
..................................................uccagaaaggagaccauugucA.....................   1   1   seq
..................................................uccagaaaggagaccauugucg.....................   1   0   seq
```

miRBase precursor : novel-nve-miR-54
Total read count : 2306
novel-nve-miR-54_guide read count : 2295
novel-nve-miR-54_star read count : 8
remaining reads : 3

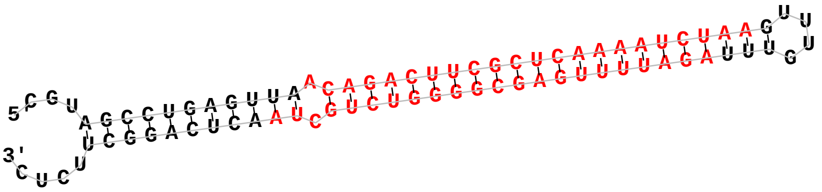

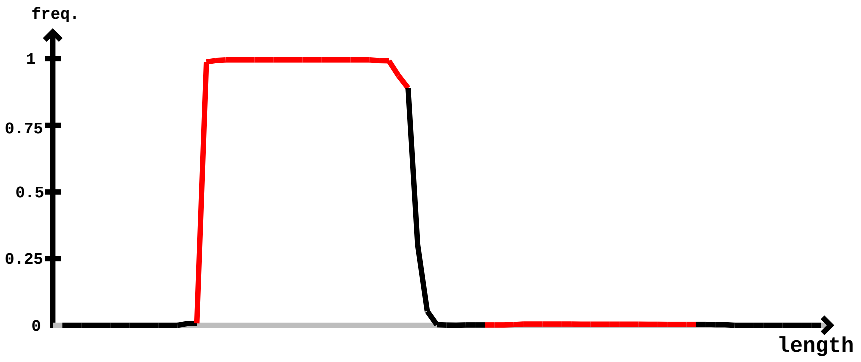


```
                                                  novel-nve-miR-54_star
                      novel-nve-miR-54_guide
5'- cguagccugaguuaacagacuucgcucaaaaucuaaguuuguuuagauuuugagcggggucugcuaacucaggcuucuc   -3'    exp
    .(.((((((((((.(((((((((((((((((((((((....))))))))))))))))))))))).))))))))))).)..    reads      mm         sample
    ............uaacagacuuUgcucaaaau...............................................    2          1          seq
    ............uaacagacuucgcucaaaauc..............................................    1          0          seq
    ............uaacagacuuUgcucaaaaucu.............................................    2          1          seq
    ............uaacGgacuucgcucaaaaucu.............................................    1          1          seq
    ............uaacagacuucgcucaaaaucu.............................................    7          0          seq
    ............uaacagacuucgcucaaaaucA.............................................    1          1          seq
    ............uaacagacuucgcucaaaaucC.............................................    1          1          seq
    .............aacagacuucgcucaaaaucC.............................................    1          1          seq
    .............aacagacuucgcucaaaaucuaa...........................................    1          0          seq
    ..............acagacuucgcucaaaau...............................................    3          0          seq
    ..............acagacuucgcucaaaaC...............................................    1          1          seq
    ..............acagacuucgcucaaaauc..............................................    1          0          seq
    ..............acagacuucgcucaaaaucC.............................................    24         1          seq
    ..............acagacuucgcucaaaaucA.............................................    1          1          seq
    ..............acagacuucgcucaaaauUu.............................................    1          1          seq
    ..............acagacuGcgcucaaaaucu.............................................    1          1          seq
    ..............acagacuCcgcucaaaaucu.............................................    1          1          seq
    ..............acagaAuucgcucaaaaucu.............................................    1          1          seq
    ..............acGgacuucgcucaaaaucu.............................................    1          1          seq
    ..............acagacuucgcucaaaaucu.............................................    81         0          seq
    ..............acagacuucgcucaaaaucG.............................................    4          1          seq
    ..............acagacuucgcucaaGaucua............................................    1          1          seq
    ..............Gcagacuucgcucaaaaucua............................................    1          1          seq
    ..............acagacuCcgcucaaaaucua............................................    2          1          seq
    ..............acagacuucgcucaaaaucuG............................................    1          1          seq
    ..............acagacuuUgcucaaaaucua............................................    1          1          seq
    ..............acagacuuGgcucaaaaucua............................................    1          1          seq
    ..............acagacuucgcucaaaaucua............................................    100        0          seq
    ..............acagacCucgcucaaaaucua............................................    1          1          seq
    ..............acagacuucgcucaaGaucuaa...........................................    2          1          seq
    ..............acagacuGcgcucaaaaucuaa...........................................    1          1          seq
    ..............acaCacuucgcucaaaaucuaa...........................................    1          1          seq
    ..............Gcagacuucgcucaaaaucuaa...........................................    7          1          seq
    ..............acagacuucgAucaaaaucuaa...........................................    5          1          seq
```

```
                                             novel-nve-miR-54_star
              novel-nve-miR-54_guide
cguagccugaguuaacagacuucgcucaaaaucuaaguuuguuuagauuuugagcggggucugcuaacucaggcuucuc

..............acagaAuucgcucaaaaucuaa...........................................    4     1     seq
..............acagUcuucgcucaaaaucuaa...........................................    1     1     seq
..............aAagacuucgcucaaaaucuaa...........................................    2     1     seq
..............acagGcuucgcucaaaaucuaa...........................................    3     1     seq
..............acagacuucgcuAaaaaucuaa...........................................    4     1     seq
..............acagacuucgcucaaaaucuaU...........................................    51    1     seq
..............acagacuucgcucaaUaucuaa...........................................    1     1     seq
..............acagacuucgcucaaaaucuaG...........................................    20    1     seq
..............acagacuucgUucaaaaucuaa...........................................    1     1     seq
..............Ccagacuucgcucaaaaucuaa...........................................    1     1     seq
..............acagacuucgcucGaaaucuaa...........................................    3     1     seq
..............acagacuucgcucaGaaucuaa...........................................    3     1     seq
..............acagacuucgcucaaaGucuaa...........................................    4     1     seq
..............acagacuuUgcucaaaaucuaa...........................................    4     1     seq
..............acagacuucgcucaaaaucuaC...........................................    41    1     seq
..............acagacuucCcucaaaaucuaa...........................................    4     1     seq
..............aUagacuucgcucaaaaucuaa...........................................    3     1     seq
..............acagacGucgcucaaaaucuaa...........................................    1     1     seq
..............Ucagacuucgcucaaaaucuaa...........................................    1     1     seq
..............acaUacuucgcucaaaaucuaa...........................................    2     1     seq
..............acagacuucgcucaaaauUuaa...........................................    8     1     seq
..............acagacuucUcucaaaaucuaa...........................................    2     1     seq
..............acGgacuucgcucaaaaucuaa...........................................    3     1     seq
..............acagacuCcgcucaaaaucuaa...........................................    7     1     seq
..............acagacuucAcucaaaaucuaa...........................................    2     1     seq
..............acagacuucgcCcaaaaucuaa...........................................    5     1     seq
..............acagacAucgcucaaaaucuaa...........................................    4     1     seq
..............acagCcuucgcucaaaaucuaa...........................................    1     1     seq
..............acagacuucgcucaaaaCcuaa...........................................    2     1     seq
..............acUgacuucgcucaaaaucuaa...........................................    1     1     seq
..............acagacuucgcucaaaaucuGa...........................................    4     1     seq
..............acagacuucgcucaaaaucuaa...........................................    1140  0     seq
..............acagacCucgcucaaaaucuaa...........................................    4     1     seq
..............acagacuucgcucaaaaucuaag..........................................    1     0     seq
..............acagacuucgcucaaaaucuaCg..........................................    2     1     seq
..............acagacuucgcucaaaaucuaaA..........................................    46    1     seq
..............acagacuucgcucaaaaucuaaC..........................................    177   1     seq
..............acagacuucgcucaaaaucuaaU..........................................    338   1     seq
..............acagacuucgcucaaaaucuaaUu.........................................    37    1     seq
..............acagacuucgcucaaaaucuaagu.........................................    1     0     seq
..............acagacuucgcucaaaaucuaaAu.........................................    6     1     seq
..............acagacuucgcucaaaaucuaaCu.........................................    67    1     seq
..............acagacuucgcucaaaaucuaaUuu........................................    3     1     seq
..............acagacuucgcucaaaaucuaaUuuu.......................................    1     1     seq
...............cagacuucgcucaaaaucuaC...........................................    1     1     seq
...............cagacuucgcucaaaaucuaa...........................................    1     0     seq
...............cagacuucgcucaaaaucuaaC..........................................    1     1     seq
...............cagacuucgcucaaaaucuaaU..........................................    6     1     seq
...............cagacuucgcucaaaaucuaaCu.........................................    4     1     seq
................agacuucgcucaaaaucuaaC..........................................    3     1     seq
................agacuucgcucaaaaucuaaUu.........................................    1     1     seq
................agacuucgcucaaaaucuaaCu.........................................    1     1     seq
...................................aguuuguuuagauuGuga..........................    1     1     seq
.........................................uuuagauuuugagcggggu...................    1     0     seq
.........................................uuuagauuuugagcgggguUu.................    1     1     seq
..............................................auuuugagcggggucugUuaa............    3     1     seq
...............................................uuuugagcggggucugcuaacu..........    1     0     seq
...............................................uuuugagcggggucugcuaacG..........    1     1     seq
...............................................uuuugagcggggucugUuaacu..........    3     1     seq
```

miRBase precursor : novel-nve-miR-55
Total read count : 1714
novel-nve-miR-55_guide read count : 1502
novel-nve-miR-55_star read count : 211
remaining reads : 1

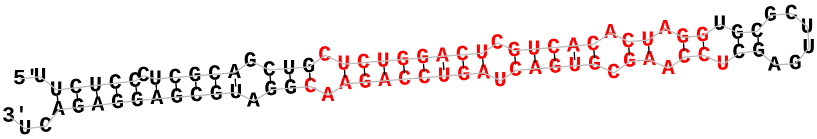

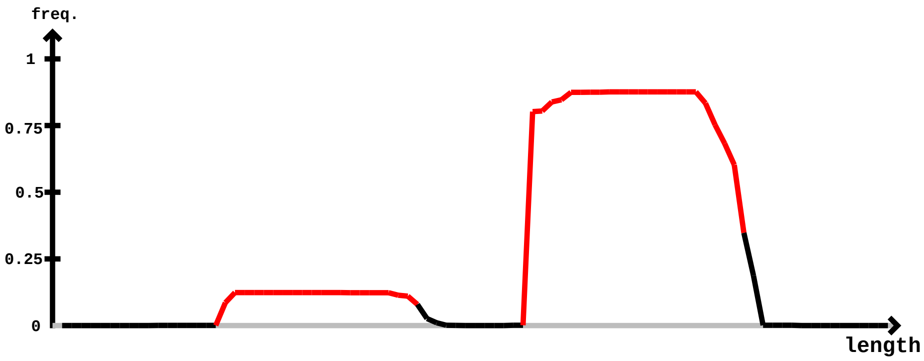


```
                                                                          novel-nve-miR-55_guide
                              novel-nve-miR-55_star
5'- uucucccucgcagcugcucuggacucgucacacuaggugcgcuugagcuccaagcgugacuaguccagaacggaugcgaggagacu   -3'    exp
    .(((((.(((((.(((.((((((((.(((((.((.((.((......)).)).)).))))).)))))))).))).))))))))))..   reads     mm        sample
    .........gcagcCgcucuggacucguc.........................................................   1         1         seq
    ................cucuggacucgucacacC....................................................   3         1         seq
    ................cucuggacucgucacacA....................................................   1         1         seq
    ................cucuggacucgucacacu....................................................   10        0         seq
    ................cucuggacucgucacacG....................................................   2         1         seq
    ................cucuggacucgucacacuU...................................................   1         1         seq
    ................cucuggacucgucacacua...................................................   2         0         seq
    ................cucugAacucgucacacuag..................................................   1         1         seq
    ................cucuggacucgucacacuag..................................................   37        0         seq
    ................cucuggacucgucGcacuag..................................................   1         1         seq
    ................cucCggacucgucacacuag..................................................   1         1         seq
    ................cucuggacucgucacacuaU..................................................   1         1         seq
    ................Uucuggacucgucacacuag..................................................   2         1         seq
    ................cucuggacucgCcacacuag..................................................   1         1         seq
    ................cucuggacucgucacacuaA..................................................   4         1         seq
    ................cucuCgacucgucacacuag..................................................   1         1         seq
    ................cucuggacucgucaUacuagg.................................................   1         1         seq
    ................cuAuggacucgucacacuagg.................................................   1         1         seq
    ................cucuggacucgucacacuagA.................................................   1         1         seq
    ................cAcuggacucgucacacuagg.................................................   1         1         seq
    ................cucuggacucgucacacuagg.................................................   59        0         seq
    ................cucuggacucgucacacuagU.................................................   1         1         seq
    ................cucuggacuAgucacacuagg.................................................   1         1         seq
    ................cucugAacucgucacacuaggu................................................   1         1         seq
    ................cucuggacucgucacacuaggu................................................   4         0         seq
    ................cucuggacucgucacacuaggC................................................   7         1         seq
    ................cucuggacucgucacacuaggCg...............................................   1         1         seq
    .................uUuggacucgucacacua...................................................   2         1         seq
    .................ucuggacucgucacacuG...................................................   1         1         seq
    .................ucuggacucgucacacuag..................................................   3         0         seq
    .................ucuggacucgucacacCag..................................................   1         1         seq
    .................ucugUacucgucacacuagg.................................................   1         1         seq
    .................ucuggacucgucacacuagg.................................................   18        0         seq
    .................ucuggacucgucacacuagA.................................................   3         1         seq
```

novel-nve-miR-55_guide

novel-nve-miR-55_star

uucucccucgcagcugcucuggacucgucacacuaggugcgcuugagcuccaagcgugacuaguccagaacggaugcgaggagacu

.................Ccuggacucgucacacuagg...	1	1	seq
.................ucuggacucgucacacuagU...	2	1	seq
.................ucuCgacucgucacacuagg...	1	1	seq
.................ucuggacucgucacacuaggC..	11	1	seq
.................ucuggacucgucacacuaggu..	4	0	seq
.................ucuggacucgucacacuaggCg...	12	1	seq
.................ucuggacucgucacacuaggug...	3	0	seq
.................ucuggacucgucacacuaggCgc..	1	1	seq
.................ucuggacucgucacacuaggugcg...	1	0	seq
....gcuccaagcgugacuaguccC...................	1	1	seq
....gcCccaagcgugacuagucca...................	1	1	seq
....uccaagUgugacuagucc....................	2	1	seq
....uccaagcgugacuagucc....................	31	0	seq
....uccaagcgugacuagucU....................	28	1	seq
....uccaagcgugacuagucA....................	7	1	seq
....Cccaagcgugacuagucc....................	2	1	seq
....Gccaagcgugacuagucc....................	2	1	seq
....uccaagcgugacuagucG....................	1	1	seq
....uccaagcgugacuaguccG...................	4	1	seq
....uccaagcgugacuaguccU...................	3	1	seq
....Cccaagcgugacuagucca...................	2	1	seq
....Accaagcgugacuagucca...................	3	1	seq
....uUcaagcgugacuagucca...................	1	1	seq
....uccaagcgugacuaguccC...................	5	1	seq
....ucUaagcgugacuagucca...................	1	1	seq
....uccaagcgugacuagucUa...................	1	1	seq
....uccaagcgugacuaguUca...................	2	1	seq
....uccaagcgugacuagucca...................	115	0	seq
....uccaagcgugacuaguccaU..................	3	1	seq
....uccaagcgugacuaguccaC..................	1	1	seq
....uccaagcgugGcuaguccag..................	2	1	seq
....uccaagcCugacuaguccag..................	1	1	seq
....Accaagcgugacuaguccag..................	2	1	seq
....Cccaagcgugacuaguccag..................	1	1	seq
....uccaagcgugacCaguccag..................	1	1	seq
....uccaagcgugacuaguccGg..................	1	1	seq
....uccaagcgugacuGguccag..................	1	1	seq
....uccaagcgugacuagucAag..................	1	1	seq
....uccaagcgugacuaUuccag..................	2	1	seq
....uccaagcgugacuaguccag..................	86	0	seq
....uccaGgcgugacuaguccag..................	1	1	seq
....uccaagcgCgacuaguccag..................	1	1	seq
....uccaagcgugacuagCccag..................	1	1	seq
....uccaagcgugacuaguccaA..................	10	1	seq
....Accaagcgugacuaguccaga.................	3	1	seq
....uccaagcgugacuaguccaga.................	119	0	seq
....Gccaagcgugacuaguccaga.................	1	1	seq
....uccaagcgugacuaguccagG.................	5	1	seq
....uccaagcgugacuaguccagU.................	1	1	seq
....uccaagcgugacuaUuccaga.................	1	1	seq
....uccaagcguAacuaguccaga.................	1	1	seq
....uccaagcgugacuaguccagaa................	375	0	seq
....uccaagcgugacuaguccagGa................	1	1	seq
....uccaagcgugacuaguccagUa................	1	1	seq
....uccaagcgugacuaguccagaG................	7	1	seq
....uccGagcgugacuaguccagaa................	1	1	seq
....uccaagcgugacuagucAagaa................	2	1	seq
....Cccaagcgugacuaguccagaa................	2	1	seq
....uccaaCcgugacuaguccagaa................	1	1	seq
....uccaagcgugacuaguccaUaa................	1	1	seq
....uccaagcgugacuaguccCgaa................	1	1	seq
....uccaagcgugacuaAuccagaa................	1	1	seq
....uccaagcgugCcuaguccagaa................	1	1	seq
....Accaagcgugacuaguccagaa................	7	1	seq
....uccaagcCugacuaguccagaa................	1	1	seq
....uGcaagcgugacuaguccagaa................	1	1	seq
....uccaagcgugacuaguccagaC................	2	1	seq
....ucUaagcgugacuaguccagaa................	3	1	seq
....uccaagcgugacuaUuccagaa................	1	1	seq

```
                                                novel-nve-miR-55_guide
                novel-nve-miR-55_star
uucucccucgcagcugcucuggacucgucacacuaggugcgcuugagcuccaagcgugacuaguccagaacggaugcgaggagacu

................................................uccaagcgCgacuaguccagaa................	1	1	seq
................................................uccaGgcgugacuaguccagaa................	1	1	seq
................................................uccaagcgugacAaguccagaa................	2	1	seq
................................................uccaagcgugGcuaguccagaa................	5	1	seq
................................................uccaagcgugacuaguccagaU................	9	1	seq
................................................Cccaagcgugacuaguccagaac...............	1	1	seq
................................................uccaagcgugacuaguccagaaU...............	134	1	seq
................................................uccaagUgugacuaguccagaac...............	1	1	seq
................................................uccaagcgugacuaguccagaaA...............	17	1	seq
................................................uccaagcgugacuaguccGgaac...............	1	1	seq
................................................uccaagcgugacuagAccagaac...............	1	1	seq
................................................uccaagcgugacuaguccagaac...............	98	0	seq
................................................uccaagcgAgacuaguccagaac...............	1	1	seq
................................................uccaagcgugacuaguccagaaG...............	3	1	seq
................................................uccaagcgugGcuaguccagaac...............	2	1	seq
................................................Accaagcgugacuaguccagaac...............	3	1	seq
................................................uccaagcgugacuaguccagaacU..............	175	1	seq
................................................uccaagcgugacuaguccagaacA..............	21	1	seq
................................................ucAaagcgugacuaguccagaacg..............	1	1	seq
................................................uccaagcgugacuagucUagaacg..............	1	1	seq
................................................uccaagcgugacuaguccagaacg..............	10	0	seq
................................................uccaagcgugacuaguccagaacC..............	19	1	seq
................................................uUcaagcgugacuaguccagaacg..............	1	1	seq
.................................................ccaagcgugacuaguccaU..................	2	1	seq
.................................................ccaagcgugacuaguccaga.................	1	0	seq
.................................................ccaagcgugacuaguccagaaG...............	1	1	seq
..................................................caagcgugacuaguccag..................	3	0	seq
..................................................caagcgugacuaguccaga.................	3	0	seq
..................................................caagcgugacuaguccUgaa................	3	1	seq
..................................................caagcgugacuaguccagaa................	7	0	seq
..................................................caagcgugacuaguccCgaac...............	1	1	seq
..................................................caagcgugacuaguccagaaU...............	2	1	seq
..................................................caagcgugacuaguccagaac...............	4	0	seq
..................................................caagcgugacuaguccagaacU..............	7	1	seq
..................................................caGgcgugacuaguccagaacg..............	1	1	seq
..................................................caagcgugacuaguccagaacA..............	4	1	seq
..................................................caagcgugacuaguccagaacC..............	1	1	seq
..................................................caagcgugGcuaguccagaacg..............	1	1	seq
..................................................Uaagcgugacuaguccagaacg..............	1	1	seq
..................................................caagcgugacuaguccagaacg..............	19	0	seq
..................................................caagcgugacuaguccUgaacg..............	1	1	seq
...................................................aagcgugacuaguccaga.................	2	0	seq
...................................................aagcgugacuaguccaUaacg..............	1	1	seq
...................................................aagcgugacuaguccagaacg..............	9	0	seq
...................................................aagcgugacuaguccagaacU..............	1	1	seq
...................................................aagcgugacuaguccagaacA..............	1	1	seq
....................................................agcgugacuaguccagaaU...............	1	1	seq
....................................................agcgugacuaguccagaac...............	4	0	seq
....................................................Ggcgugacuaguccagaacg..............	1	1	seq
....................................................agcgugacuagCccagaacg..............	1	1	seq
....................................................agcgugacuaguccagaacA..............	8	1	seq
....................................................agcgugacuaguccagaacC..............	1	1	seq
....................................................agcgugacuaguccagaacU..............	11	1	seq
....................................................agcgugacuaguccagaacg..............	20	0	seq
....................................................agcgugacuaguccagaUcg..............	1	1	seq
......................................................cgugacuaguccagaacg..............	1	0	seq
........................................................ugacuaguccagaacggauA..........	1	1	seq
........................................................ugacuaguccagaacggaug..........	1	0	seq
```

miRBase precursor : novel-nve-miR-56
Total read count : 184
novel-nve-miR-56_guide read count : 160
novel-nve-miR-56_star read count : 24
remaining reads : 0

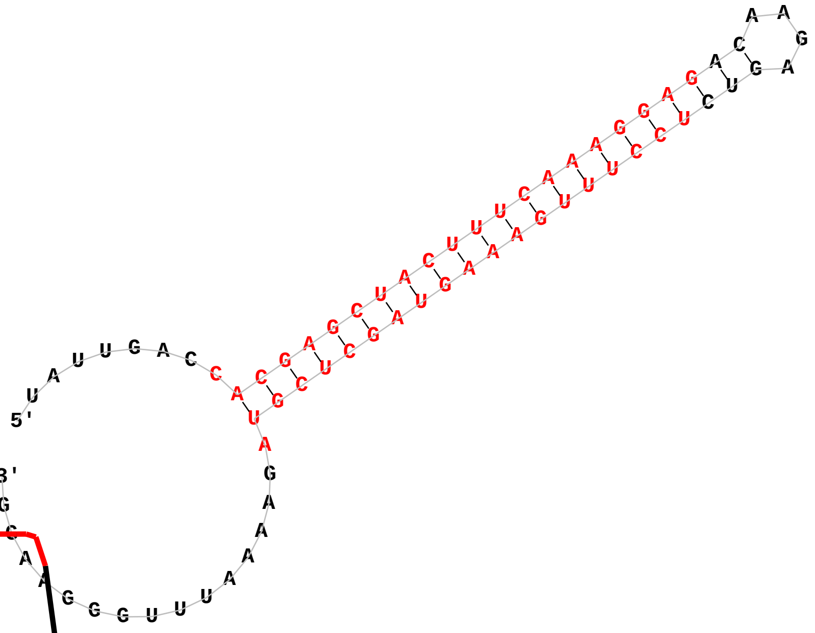

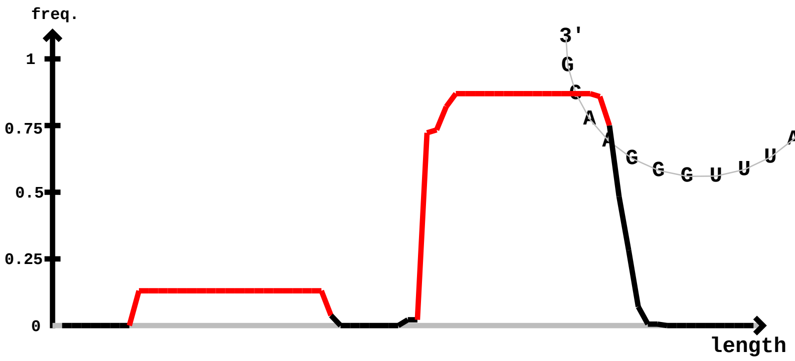


```
        novel-nve-miR-56_star
                                        novel-nve-miR-56_guide
5'- uauugaccacgagcuacuuucaaaggagacaagagucuccuuugaaaguagcucguagaaaauuugggaagg  -3'   exp
    ........((((((((((((((((((((....))))))))))))))))))))).....................   reads    mm    sample
    .......cacgagcuacuuucaaagga..............................................   15       0     seq
    .......cacgUgcuacuuucaaagga..............................................   1        1     seq
    .......cacgagcuacuuCcaaagga..............................................   1        1     seq
    .......cacgagcuacuuucaaaggag.............................................   1        0     seq
    .......cacgagcuacuuucaaaggaC.............................................   2        1     seq
    .......cacgagcuacuuucaaaggaU.............................................   4        1     seq
    ...................................ucuccuuugaaaguagcucg..................   1        0     seq
    ...................................ucuccuuugaaaguagcucgua................   2        0     seq
    ...................................ucuccuuugaaaguagcucguag...............   1        0     seq
    .....................................uccuuugaaaguagcucg..................   1        0     seq
    .....................................uccuuugaaaguagcucgu.................   17       0     seq
    .....................................uccuuugaaaguagcucgC.................   1        1     seq
    .....................................uccuuugaaaguagcucgA.................   2        1     seq
    .....................................uccuuugaaaguGgcucgua................   1        1     seq
    .....................................uccuuugaaaguagcucguG................   12       1     seq
    .....................................uccuuugaaaguagcucgua................   33       0     seq
    .....................................uccuuugaaaguagcucguU................   1        1     seq
    .....................................uccuuugaaaguagcucguaU...............   1        1     seq
    .....................................uccuuugaaaguaUcucguag...............   1        1     seq
    .....................................uccuuugaaaguagcucguag...............   8        0     seq
    .....................................uccuuugaaaguagcucguGg...............   10       1     seq
    .....................................ucAuuugaaaguagcucguaga..............   2        1     seq
    .....................................uccuuugaaaguagcucguGga..............   1        1     seq
    .....................................uccuuugaaaguagcucguaga..............   27       0     seq
    .....................................uccuCugaaaguagcucguaga..............   1        1     seq
    .....................................uccuuugaaaguagcucguagaa.............   10       0     seq
    ......................................Ucuuugaaaguagcucguag...............   1        1     seq
    ......................................ccuuugaaaguagcucguaga..............   1        0     seq
    .......................................cuuugaaaguagcucguag...............   2        0     seq
    .......................................cuuugaaaguagcucguGg...............   7        1     seq
    .......................................cuuugaaaguagcucguaga..............   7        0     seq
    ........................................uuugaaaguagcucguGg...............   6        1     seq
    ........................................uuugaaaguagcucguagaa.............   1        0     seq
    ........................................uuugaaaguagcucguagaU.............   1        1     seq
```

novel-nve-miR-56_star

novel-nve-miR-56_guide

uauugaccacgagcuacuuucaaaggagacaagagucuccuuugaaaguagcucguagaaaauuugggaagg

....uuugaaaguagcucguagaaaa.......... 1 0 seq

miRBase precursor : novel-nve-miR-57
Total read count : 289
novel-nve-miR-57_guide read count : 288
novel-nve-miR-57_star read count : 1
remaining reads : 0

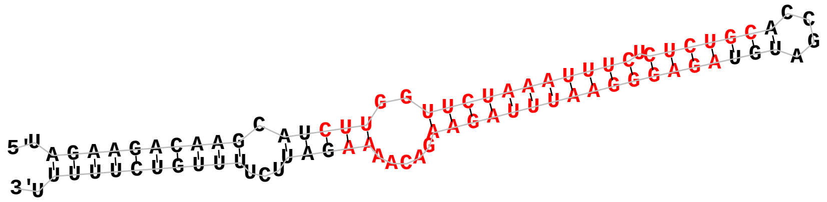

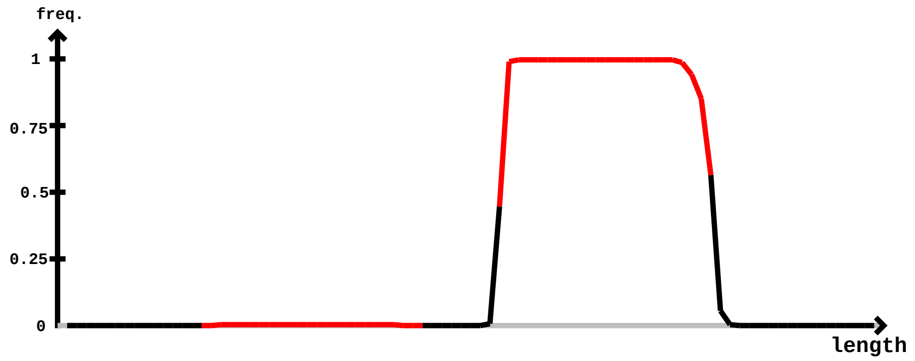


```
                    novel-nve-miR-57_star
                                                novel-nve-miR-57_guide
5'- uagaagacaagcaucuugguucuaaauuucucucugcaccgauguagagggaauuuagaagacaaaagauucuuuugucuuuuu   -3'    exp
    .((((((((((.(((((..(((((((((((.(((((((....)))))))))))))))))).....)))))...)))))))))).    reads     mm     sample
    ...............uugguucuaaauuucucuA..................................................    1         1      seq
    ...........................................guagagggaauuuagaagacaa...................    1         0      seq
    ...........................................guagagggaauuuagaagacaaaa.................    1         0      seq
    ............................................uagagggaauuuagaagac.....................    1         0      seq
    ............................................uagagggaaCuuagaagaca....................    1         1      seq
    ............................................uagagggaauuuagaagaca....................    11        0      seq
    ............................................uagagggaauuuagaagacaU...................    1         1      seq
    ............................................uagagggaauuuagaagacaC...................    1         1      seq
    ............................................uagagggaauuuagaagacaa...................    13        0      seq
    ............................................uGgagggaauuuagaagacaa...................    1         1      seq
    ............................................uagaggUaauuuagaagacaa...................    1         1      seq
    ............................................uagagggaauuuagaagacaaC..................    3         1      seq
    ............................................uagagggaauuuaAaagacaaa..................    1         1      seq
    ............................................uagagggaauuuagaagacaaU..................    6         1      seq
    ............................................uagagggaauuuagaagacaGa..................    1         1      seq
    ............................................uagagggaauuuagaagacaaa..................    39        0      seq
    ............................................uagagggaauuuagaagacaaaC.................    2         1      seq
    ............................................uagagggaauuuagaagacaaaU.................    9         1      seq
    ............................................uagagggaauuuagaagacaaaa.................    29        0      seq
    ............................................uagagggaauuuagaagacaaaaU................    6         1      seq
    ............................................uagagggaauuuagaagacaaaaA................    1         1      seq
    .............................................agagggaauuuagaagac.....................    1         0      seq
    .............................................agagggUauuuagaagac.....................    1         1      seq
    .............................................agagggaauuuagaagaca....................    1         0      seq
    .............................................agagggaauuuagaagacaa...................    4         0      seq
    .............................................agagggaauuuaUaagacaa...................    2         1      seq
    .............................................agagggaauuuagaagacaaU..................    1         1      seq
    .............................................agagggaauuuagaagacaaa..................    27        0      seq
    .............................................agagggaaCuuagaagacaaa..................    1         1      seq
    .............................................Ggagggaauuuagaagacaaa..................    1         1      seq
    .............................................agagggaauuuagaaUacaaa..................    1         1      seq
    .............................................agGgggaauuuagaagacaaa..................    1         1      seq
    .............................................agagggaauuuagaagacaGa..................    1         1      seq
    .............................................agagggaauuuagaagacaaaa.................    80        0      seq
```

```
              novel-nve-miR-57_star
                                             novel-nve-miR-57_guide
uagaagacaagcaucuugguucuaaauuucucucugcaccgauguagagggaauuuagaagacaaaagauucuuuugucuuuuu

.............................................agaggCaauuuagaagacaaaa.................    1    1    seq
.............................................agagggaauuuagaagacaaCa.................    1    1    seq
.............................................agagggaauuuagGagacaaaa.................    1    1    seq
.............................................agagggaauuuagaagacGaaa.................    1    1    seq
.............................................agagggaauuuagaagacaaaU.................    10   1    seq
.............................................agagggGauuuagaagacaaaa.................    1    1    seq
.............................................Ggagggaauuuagaagacaaaa.................    2    1    seq
.............................................agagggaauuuagaagacaaaC.................    9    1    seq
.............................................agagggaauuuagaagacaaaaU................    8    1    seq
.............................................agagggaauuuagaagacaaaagG...............    1    1    seq
..............................................gagggaauuuagaagacaa...................    2    0    seq
```

miRBase precursor : novel-nve-miR-58
Total read count : 373
novel-nve-miR-58_guide read count : 371
novel-nve-miR-58_star read count : 2
remaining reads : 0

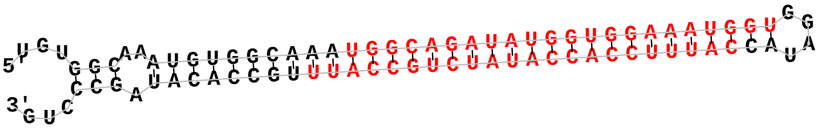

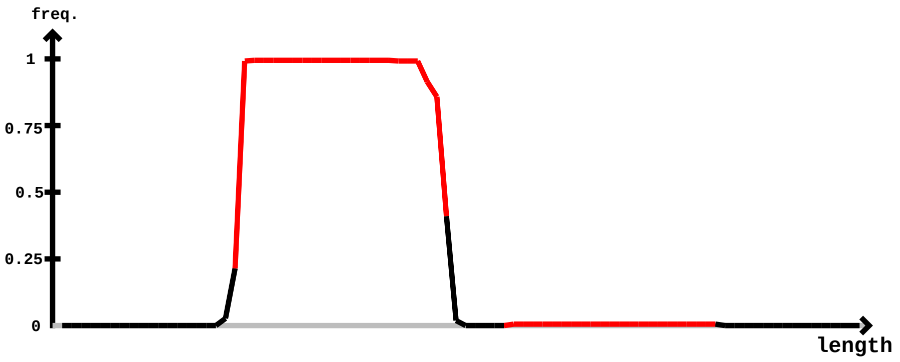

novel-nve-miR-58_guide

novel-nve-miR-58_star

```
5'- uguggcaaauguggcaaauggcagauaugguggaaaugguggauaccauuuccaccauaucugccauuugccacauagcccug  -3'    exp
    ...(((..((((((((((((((((((((((((((((((((....)))))))))))))))))))))))))))))))).)))...   reads     mm        sample
    ................aauggcagauauggugga.................................................   1         0         seq
    ................aauggcagauaugguggaaau..............................................   1         0         seq
    ................aauggcagauaugguggaaaug.............................................   3         0         seq
    ................aauggcagauaugguggaaaugA............................................   1         1         seq
    ................aauggcagauaugguggaaaugg............................................   4         0         seq
    .................auggcagauaugguggaaau..............................................   10        0         seq
    .................auggcagauaugguggaaaug.............................................   2         0         seq
    .................Guggcagauaugguggaaaugg............................................   1         1         seq
    .................auggcagauaugguggaaaugg............................................   47        0         seq
    .................auggAagauaugguggaaaugg............................................   1         1         seq
    .................auggUagauaugguggaaaugg............................................   1         1         seq
    .................auggcagauauggugGaaugg.............................................   2         1         seq
    .................auggcagauaugguggaaaugA............................................   3         1         seq
    .................auggcagauaCgguggaaaugg............................................   1         1         seq
    .................auggcagauaugguggaaGugg............................................   1         1         seq
    .................auggcagauaugguggaaauggu...........................................   1         0         seq
    ..................uggcagauaugguggaaaC..............................................   2         1         seq
    ..................uggUagauaugguggaaau..............................................   1         1         seq
    ..................uggcagaGaugguggaaau..............................................   1         1         seq
    ..................uggcagauaugguggaaau..............................................   14        0         seq
    ..................uggcagauaugguggaaaug.............................................   13        0         seq
    ..................uggcagauaugguggaaauA.............................................   3         1         seq
    ..................uggcagauauUguggaaaugg............................................   1         1         seq
    ..................uggcagauaugguggaaaugC............................................   2         1         seq
    ..................ugUcagauaugguggaaaugg............................................   1         1         seq
    ..................uggcagUuaugguggaaaugg............................................   1         1         seq
    ..................uggcagauauggCggaaaugg............................................   1         1         seq
    ..................uggcagauaugguggaaaugA............................................   11        1         seq
    ..................uggcagauaugguggaaaugU............................................   4         1         seq
    ..................uggcagauaugguCgaaaugg............................................   2         1         seq
    ..................uUgcagauaugguggaaaugg............................................   1         1         seq
    ..................uggcagauaugguggaaaugg............................................   78        0         seq
    ..................uggcagaCaugguggaaaugg............................................   2         1         seq
    ..................uggcaAauaugguggaaaugg............................................   1         1         seq
```

```
                  novel-nve-miR-58_guide
                                              novel-nve-miR-58_star
uguggcaaauguggcaaauggcagauaugguggaaaugguggauaccauuuccaccauaucugccauuugccacauagcccug

..................uggcagGuaugguggaaauggu...........................................    1      1      seq
..................uggcagauaugguggaaauggu...........................................    116    0      seq
..................Gggcagauaugguggaaauggu...........................................    2      1      seq
..................uggcagauaugguggaaUuggu...........................................    1      1      seq
..................uCgcagauaugguggaaauggu...........................................    1      1      seq
..................uggcagauaugguggaaauggG...........................................    2      1      seq
..................Aggcagauaugguggaaauggu...........................................    1      1      seq
..................uggcagauaugguggaaauggA...........................................    10     1      seq
..................uggcagauaugguggaaauggC...........................................    10     1      seq
..................uggcagauaugguggaaaugguC..........................................    1      1      seq
..................uggcagauaugguggaaaugguU..........................................    6      1      seq
...................Ugcagauaugguggaaauggu...........................................    1      1      seq
..............................................cauuuccaccauaucugccaCu...............    2      1      seq
```

miRBase precursor : novel-nve-miR-59
Total read count : 1378
novel-nve-miR-59_guide read count : 1332
novel-nve-miR-59_star read count : 22
remaining reads : 24

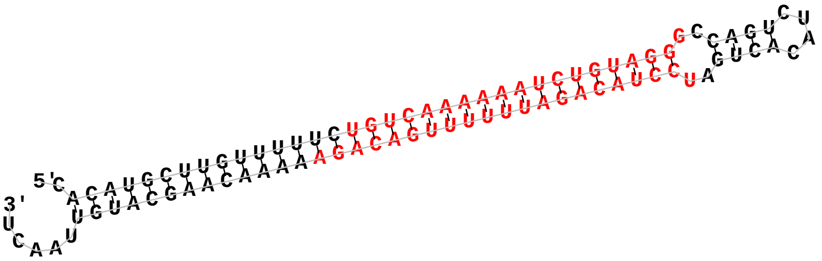

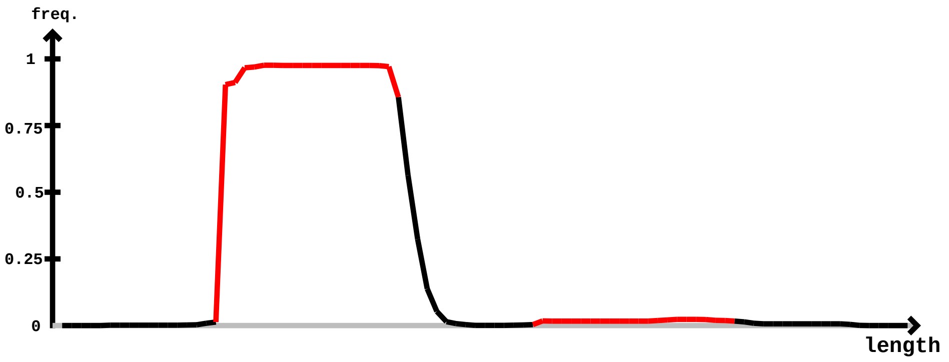


```
                  novel-nve-miR-59_guide
                                                 novel-nve-miR-59_star
5'- cacaugcuuguuuuucugucaaaaaaucuguagggccagucuacacugauccuacagauuuuuugacagaaaaacaagcauguuaacu   -3'    exp
    .(((((((((((((((((((((((((((((((((..((((....))))..))))))))))))))))))))))))))))))))).....    reads    mm    sample
    ....ugcuuguuuuucugucaa..................................................................    2    0    seq
    ............uuucugucaaaaaaucugua........................................................    1    0    seq
    .............uucugucaaaaaaucuguagg......................................................    1    0    seq
    ..............ucugucaaaaaaucuguag.......................................................    4    0    seq
    ..............ucugucaaaaaaucuguaA.......................................................    1    1    seq
    ..............ucugucaaaaaaucuguagg......................................................    3    0    seq
    ...............cugucaaaaaaucuguagg......................................................    3    0    seq
    ...............cugucaaaaaaucuguagA......................................................    2    1    seq
    ...............cugucaaaaaaucuguaggg.....................................................    1    0    seq
    ................ugucaaaaaaucuguagA......................................................    12    1    seq
    ................Ggucaaaaaaucuguagg......................................................    1    1    seq
    ................ugucaaaaaaucuguUgg......................................................    1    1    seq
    ................ugucaaaaaaCcuguagg......................................................    1    1    seq
    ................ugucaaaGaaucuguagg......................................................    5    1    seq
    ................ugAcaaaaaaucuguagg......................................................    1    1    seq
    ................ugucaaaaaaucuguGgg......................................................    2    1    seq
    ................ugucGaaaaaucuguagg......................................................    1    1    seq
    ................ugucaaaaaauUuguagg......................................................    1    1    seq
    ................ugucaaaaaaucuguagg......................................................    117    0    seq
    ................uAucaaaaaaucuguagg......................................................    1    1    seq
    ................ugucaaaaaaucuguagU......................................................    3    1    seq
    ................ugucaaaaGaucuguagg......................................................    2    1    seq
    ................Agucaaaaaaucuguagg......................................................    1    1    seq
    ................ugucaaaGaaucuguaggg.....................................................    9    1    seq
    ................ugucaGaaaaucuguaggg.....................................................    2    1    seq
    ................ugucaaGaaaucuguaggg.....................................................    2    1    seq
    ................uguUaaaaaaucuguaggg.....................................................    4    1    seq
    ................ugucaaaaaaucCguaggg.....................................................    4    1    seq
    ................ugucGaaaaaucuguaggg.....................................................    2    1    seq
    ................Ggucaaaaaaucuguaggg.....................................................    2    1    seq
    ................ugucaaaaaaucuguGggg.....................................................    1    1    seq
    ................ugucaaaUaaucuguaggg.....................................................    1    1    seq
    ................ugucaaaaaaucuguaggU.....................................................    16    1    seq
    ................ugucaaaaaauUuguaggg.....................................................    3    1    seq
```

```
                novel-nve-miR-59_guide
                                                 novel-nve-miR-59_star
cacaugcuuguuuuucugucaaaaaaucuguagggccagucuacacugauccuacagauuuuuugacagaaaaacaagcauguuaacu

................ugucaaaaaaucuguaggg.....................................................    305    0    seq
................ugucaaaaaaCcuguaggg.....................................................    2      1    seq
................Agucaaaaaaucuguaggg.....................................................    4      1    seq
................ugucCaaaaaucuguaggg.....................................................    2      1    seq
................ugucaaaaaGucuguaggg.....................................................    2      1    seq
................ugucaaaaaaucuguaggC.....................................................    1      1    seq
................ugucaaaCaaucuguaggg.....................................................    1      1    seq
................ugucaaaaaaucuUuaggg.....................................................    2      1    seq
................ugucaaaaGaucuguaggg.....................................................    5      1    seq
................Cgucaaaaaaucuguaggg.....................................................    1      1    seq
................ugucUaaaaaucuguaggg.....................................................    1      1    seq
................ugucaaaaaaucuguagCg.....................................................    1      1    seq
................ugucaaaaaaucuguaggA.....................................................    29     1    seq
................ugucaaaaaaucCguagggc....................................................    1      1    seq
................ugucaaaaaaucugGagggc....................................................    1      1    seq
................Agucaaaaaaucuguagggc....................................................    4      1    seq
................ugucaaaaaGucuguagggc....................................................    2      1    seq
................ugucaaaaaaucuguaAggc....................................................    1      1    seq
................ugucaUaaaaucuguagggc....................................................    1      1    seq
................ugucaaaaaaucuguagggU....................................................    62     1    seq
................ugucaaaaaaucuguagggA....................................................    21     1    seq
................uguUaaaaaaucuguagggc....................................................    1      1    seq
................ugAcaaaaaaucuguagggc....................................................    1      1    seq
................ugucaaaaGaucuguagggc....................................................    6      1    seq
................Cgucaaaaaaucuguagggc....................................................    2      1    seq
................ugucaaaaaaucuguagggc....................................................    190    0    seq
................ugucaaaaaauAuguagggc....................................................    1      1    seq
................ugucaaaaaaucuguaUggc....................................................    1      1    seq
................ugucaaaGaaucuguagggc....................................................    5      1    seq
................ugucaaaUaaucuguagggc....................................................    1      1    seq
................ugucaaaaaaucuguagggG....................................................    5      1    seq
................ugucaGaaaaucuguagggc....................................................    2      1    seq
................ugucaaaaaauUuguagggc....................................................    1      1    seq
................ugucaaaaGaucuguagggcc...................................................    2      1    seq
................ugCcaaaaaaucuguagggcc...................................................    1      1    seq
................ugucaaaaaaucuguGgggcc...................................................    1      1    seq
................ugucaaaaaauAuguagggcc...................................................    1      1    seq
................ugucaaaaaaucuguagggcc...................................................    117    0    seq
................uAucaaaaaaucuguagggcc...................................................    1      1    seq
................ugucaaaaaaucuguagggcG...................................................    2      1    seq
................ugucaaGaaaucuguagggcc...................................................    1      1    seq
................ugucaaaaaaucuguagggcA...................................................    27     1    seq
................ugucaGaaaaucuguagggcc...................................................    1      1    seq
................ugucaaaaaaucuguagggcU...................................................    70     1    seq
................Agucaaaaaaucuguagggcc...................................................    2      1    seq
................ugucaaaaaaucuguagggccU..................................................    26     1    seq
................ugucaaaaaaucuguagggcUa..................................................    1      1    seq
................ugAcaaaaaaucuguagggcca..................................................    1      1    seq
................Agucaaaaaaucuguagggcca..................................................    3      1    seq
................ugucaaaaaaucuguagggUca..................................................    2      1    seq
................ugucaaUaaaucuguagggcca..................................................    1      1    seq
................ugCcaaaaaaucuguagggcca..................................................    3      1    seq
................ugucaaaaaaucuguagggccC..................................................    9      1    seq
................ugucaaaaaaucuguaggUcca..................................................    1      1    seq
................ugucaaaaaaucuguagggccG..................................................    3      1    seq
................ugucaaaaaaucuguagggcca..................................................    57     0    seq
................ugucaaaaaaucuguagggccaU.................................................    8      1    seq
................ugucaaaaaaucuguagggccag.................................................    16     0    seq
................ugucaaaaaGucuguagggccag.................................................    2      1    seq
................ugucaaaaaaucuguagggccaC.................................................    2      1    seq
................ugucaaaaaaucuguagggccaCu................................................    1      1    seq
................ugucaaaaaaucuguagggccaUu................................................    5      1    seq
.................gAcaaaaaaucuguagggc....................................................    1      1    seq
.................gucaaaaaaucuguagggcc...................................................    2      0    seq
.................gucaaaaaaucuguagggcU...................................................    2      1    seq
.................gucaaaaaaucCguagggcca..................................................    1      1    seq
.................gucaaaaaaucuguagggcca..................................................    2      0    seq
.................gucaaaaaaucAguagggcca..................................................    1      1    seq
.................gucaaaaaaucuguagggccU..................................................    1      1    seq
```

novel-nve-miR-59_guide

novel-nve-miR-59_star

```
cacaugcuuguuuuucugucaaaaaaucuguagggccagucuacacugauccuacagauuuuuugacagaaaaacaagcauguuaacu

.................gucaaaaaaucuguagggccag.................................................	1	0	seq
..................ucaaaaaaucuguagggU....................................................	11	1	seq
..................Gcaaaaaaucuguagggc....................................................	1	1	seq
..................ucaaaaaaucuguagggc....................................................	8	0	seq
..................ucaaaaaaucuguagggcc...................................................	17	0	seq
..................ucaaaaaaucuguaggAcc...................................................	1	1	seq
..................ucaaaaaaucuguagggcA...................................................	1	1	seq
..................ucaaaaaaucuguagggcU...................................................	6	1	seq
..................ucaaaaaaucuguagggcca..................................................	3	0	seq
..................ucaaaGaaucuguagggccag.................................................	1	1	seq
..................ucaaaaaaucuguagggccag.................................................	15	0	seq
..................Acaaaaaaucuguagggccag.................................................	1	1	seq
..................ucaaaaaaucuguagggccaA.................................................	1	1	seq
..................ucaaaaaaucuguagggccaAu................................................	1	1	seq
..................ucaaaGaaucuguagggccagu................................................	3	1	seq
..................ucaaaaaaucuguagggccaguc...............................................	3	0	seq
..................ucaaaaaaucuguagggccagucu..............................................	3	0	seq
...................caaaaaaucuguagggcc...................................................	2	0	seq
...................caaaaaaucuguagggcca..................................................	1	0	seq
...................caaaaaaucuguagggccag.................................................	1	0	seq
....................aaaaaaucuguagggcca..................................................	1	0	seq
....................aaaaaaucuguagggccag.................................................	5	0	seq
....................aaaaaaucuguagggccaguU...............................................	2	1	seq
....................aaaaaaucuguagggccagucu..............................................	1	0	seq
......................aaaaucuguagggccagucuacacugau......................................	1	0	seq
..............................................ugauccuacagauuuuuuga......................	1	0	seq
...............................................gauccuacagauuuuuugacag...................	1	0	seq
................................................auccuacagauuuuuugac.....................	2	0	seq
.................................................uccuacagauuuuuugac.....................	2	0	seq
.................................................uccuacagauuuuuugaca....................	1	0	seq
.................................................uccuacagauuuuuugacag...................	1	0	seq
.................................................uccuacagauuuuuugacaA...................	1	1	seq
.................................................ucUuacagauuuuuugacaga..................	1	1	seq
.................................................uccuacagauuuuuugacagC..................	1	1	seq
.................................................uccuacagauuuuuugacaCa..................	2	1	seq
.................................................uccuacagauuuuuugacagCa.................	1	1	seq
.................................................uccuacagauuuuuugacaUaa.................	1	1	seq
.................................................uccuacagauuuuuugacagaC.................	1	1	seq
.................................................uccuacagauuuuuugacagaa.................	4	0	seq
.................................................uccuacagauuuuuugacagaaU................	2	1	seq
.................................................uccuacagauuuuuugacagaaa................	1	0	seq
.............................................................uuugacagaaaaacaagcaug......	1	0	seq
.............................................................uuugacagaaaaacaagcGug......	1	1	seq
.............................................................uuugacagaaaaacaagcauA......	1	1	seq
..............................................................uugacagaaaaacaagcaug......	2	0	seq
..............................................................uugacagaaaaacaagcaugG.....	1	1	seq
...............................................................ugacagaaaaacaagcau.......	3	0	seq
```

miRBase precursor : novel-nve-miR-62
Total read count : 182
novel-nve-miR-62_guide read count : 175
novel-nve-miR-62_star read count : 7
remaining reads : 0

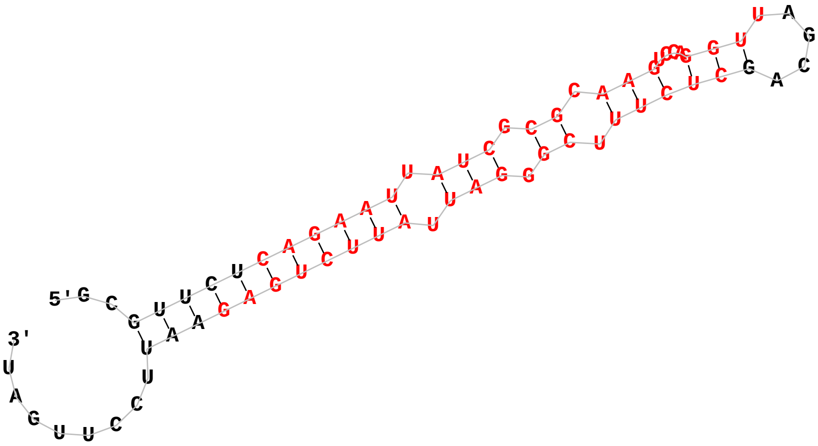

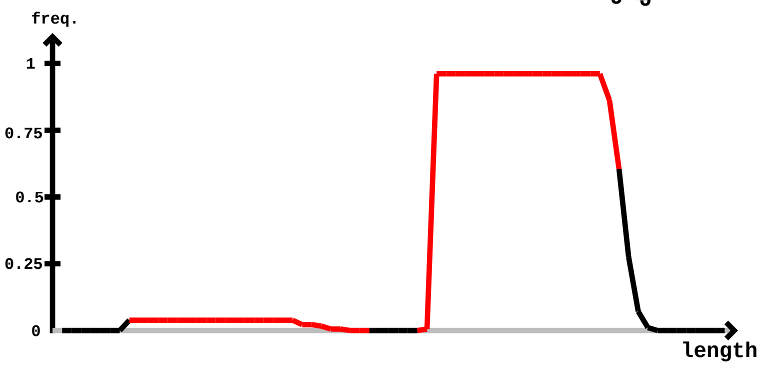


```
                                    novel-nve-miR-62_guide
          novel-nve-miR-62_star
5'- gcguucucagaauuaucgcgcaaguccagguuagcagcucuuucgggauuauucugagaauuccuugau   -3'    exp
    ..(((((((((((.(((.((.(((....(((.....)))))).)).))).)))))))))))........    reads     mm        sample
    ......ucagaauuaucgcgcaag.............................................    3         0         seq
    ......ucagaauuaucgcgcaaguc...........................................    1         0         seq
    ......ucagaauuaucgcgcaagucc..........................................    1         0         seq
    ......ucagaauuaucgcgcaagucU..........................................    1         1         seq
    ......ucagaauuaucgcgcaaguccaC........................................    1         1         seq
    .....................................cucuuucgggauuauucugag...........    1         0         seq
    ......................................uAuuucgggauuauucug.............    1         1         seq
    ......................................ucuuucgggauuauucuA.............    1         1         seq
    ......................................Acuuucgggauuauucug.............    1         1         seq
    ......................................ucuuucgggauuauucug.............    15        0         seq
    ......................................ucuuucgggauuauucuga............    45        0         seq
    ......................................ucuuucgggauuauucugU............    1         1         seq
    ......................................Ccuuucgggauuauucuga............    1         1         seq
    ......................................Acuuucgggauuauucugag...........    2         1         seq
    ......................................ucuuucgggauuauucugUg...........    1         1         seq
    ......................................ucuuucgggauuauucugaC...........    1         1         seq
    ......................................ucuuucgggauuauucugaA...........    3         1         seq
    ......................................ucuuucgggauuauucugag...........    51        0         seq
    ......................................ucuuucgggauuauucugaU...........    1         1         seq
    ......................................ucuuucgggauuGuucugaga..........    1         1         seq
    ......................................ucuuucgggauuauucugaga..........    34        0         seq
    ......................................ucuuucgggauuauucugagG..........    1         1         seq
    ......................................ucuuucgggGuuauucugaga..........    1         1         seq
    ......................................ucuuucgggauuauucugagaU.........    2         1         seq
    ......................................ucuuucgggauuauucugagaC.........    1         1         seq
    ......................................ucCuucgggauuauucugagaa.........    1         1         seq
    ......................................ucuuucgggauuauucugagaa.........    6         0         seq
    ......................................ucuuucgggauuauucugagaG.........    1         1         seq
    ......................................ucuuucgggauuauucugagaCu........    1         1         seq
    ......................................ucuuucgggauuauucugagaUu........    1         1         seq
```

miRBase precursor : novel-nve-miR-63
Total read count : 190
novel-nve-miR-63_guide read count : 186
novel-nve-miR-63_star read count : 0
remaining reads : 4

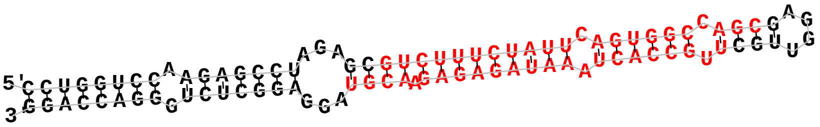

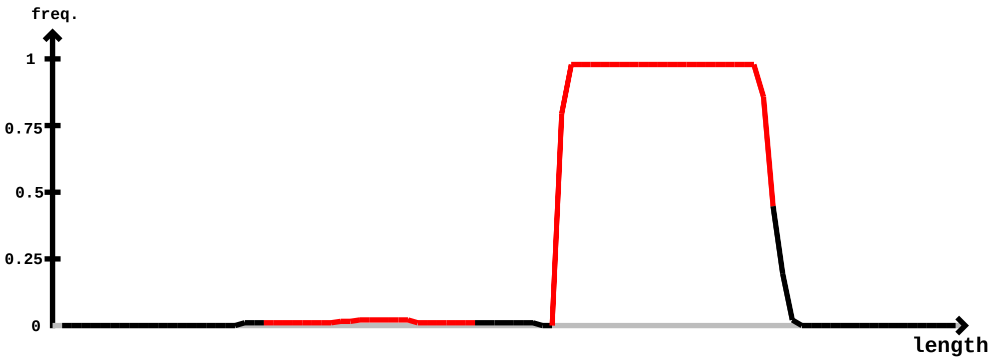


```
                         novel-nve-miR-63_star
                                                  novel-nve-miR-63_guide
5'- ccugguccaagagccuagagcgucuuucuauucaguggccagcgagguugcuugccacuaaauagagagaacguaggaggcucugggaccagg  -3'   exp
    ((((((((.(((((((...(((((((((((((.((((((.((((....)))).)))))).))))))))).))))...))))))).))))))))   reads   mm   sample
    ..................agcgucuCucuauucagu.........................................................   2       1    seq
    ............................uauuUaguggccagcgagguu............................................   1       1    seq
    ..............................uuUaguggccagcgagguu............................................   1       1    seq
    ...................................................uugccacuaaauagagGgaac.....................   1       1    seq
    ...................................................uugccacuaaauagagagaac.....................   18      0    seq
    ...................................................uugccacuaaauagagagaaU.....................   4       1    seq
    ...................................................uugccacuaaauagagagaacC....................   7       1    seq
    ...................................................uugccacuaaauagagagaacg....................   14      0    seq
    ...................................................uugccacuaaauagagagaacA....................   5       1    seq
    ...................................................uugccacuaaauagagagaacU....................   51      1    seq
    ...................................................uugcUacuaaauagagagaacg....................   1       1    seq
    ...................................................uugccacuaaauagagagaacgu...................   23      0    seq
    ...................................................uugccacuaaauagagagGacgu...................   1       1    seq
    ...................................................uugccacuaaauagagagaacgC...................   1       1    seq
    ...................................................uugccacuaaauagagUgaacgu...................   1       1    seq
    ...................................................uugccacuaaGuagagagaacgu...................   1       1    seq
    ...................................................uugccacuaaauagagagaacUu...................   3       1    seq
    ...................................................uugccacuaaauagagagaacgA...................   1       1    seq
    ...................................................uugccacuaaauagagagaacgua..................   6       0    seq
    ...................................................uugccacuaaauagagagaacguU..................   9       1    seq
    ...................................................uugccacuaaauagagagaacguaU.................   1       1    seq
    ...................................................uugccacuaaauagagagaacguaC.................   1       1    seq
    ...................................................uugccacuaaauagagagaacguag.................   2       0    seq
    ....................................................ugccacuaaauagagagaacgu...................   10      0    seq
    ....................................................ugccacuaaauagagagaacgC...................   2       1    seq
    ....................................................ugccacuaaauagagagaacUu...................   4       1    seq
    ....................................................ugccacuaaauagagagaacgA...................   1       1    seq
    ....................................................ugccacuaaauagagagaacgua..................   9       0    seq
    ....................................................ugccacuaaauagagagaacguU..................   7       1    seq
    ....................................................ugccacuaaauagagagaacguC..................   1       1    seq
    ....................................................uAccacuaaauagagagaacgua..................   1       1    seq
```

miRBase precursor : novel-nve-miR-66
Total read count : 334
novel-nve-miR-66_guide read count : 265
novel-nve-miR-66_star read count : 69
remaining reads : 0

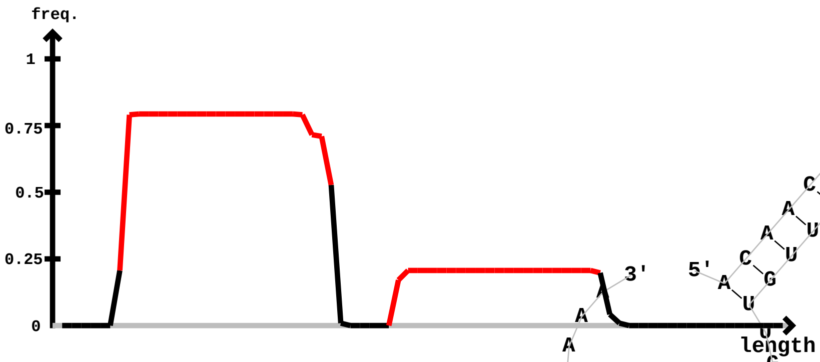

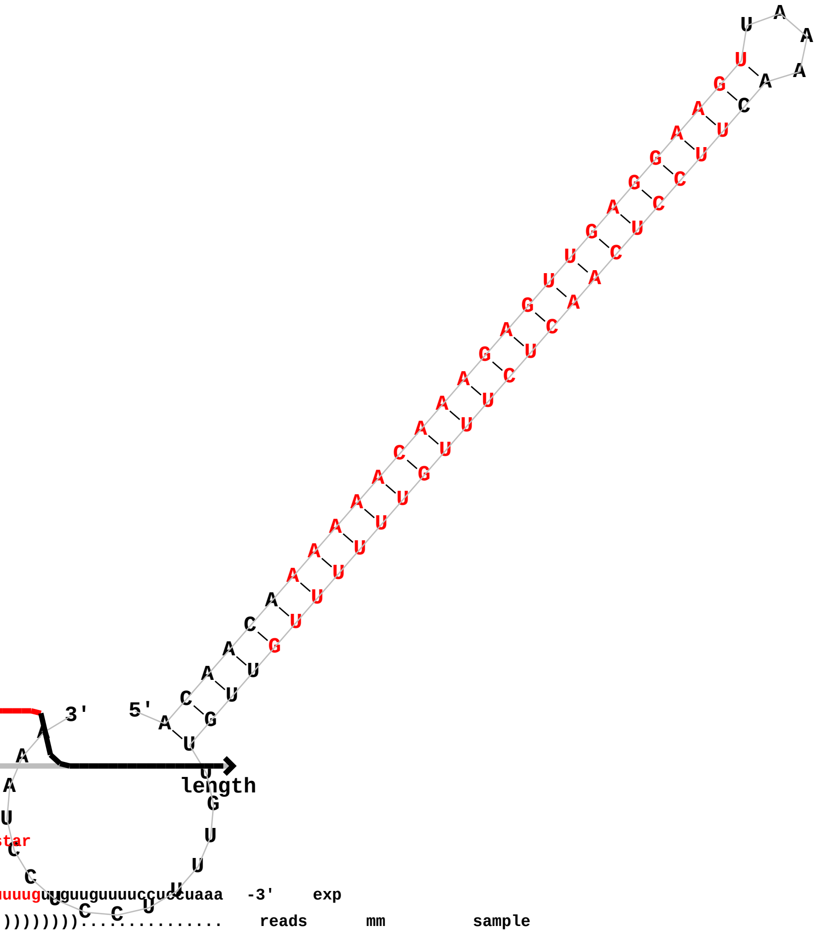

novel-nve-miR-66_star

novel-nve-miR-66_guide

```
5'- acaacaaaaaaacaaagaguugaggaaguuaaaacuuccucaacucuuuguuuuuuguuguuguuuuccuccuaaa   -3'    exp
    (((((((((((((((((((((((((((....)))))))))))))))))))))))))))..................    reads      mm         sample
    .....aaaaaacaaagaguugagga...................................................    4          0          seq
    .....aGaaaacaaagaguugagga...................................................    1          1          seq
    .....aaaaaacaaagaguugaggaa..................................................    1          0          seq
    .....aaaaaacaaagaguugaggaaU.................................................    11         1          seq
    .....aaaaaacaaagaguugGggaag.................................................    1          1          seq
    .....aaaaaacaaagaguugaggaag.................................................    17         0          seq
    .....aaaaaacaaagaguugaggaaA.................................................    2          1          seq
    .....aaaaaacaaagaguugaggaaC.................................................    5          1          seq
    .....aaaaaacaaagGguugaggaag.................................................    1          1          seq
    .....aaaaaacaaagaguugaggaGg.................................................    1          1          seq
    .....aaaaaacaaagaguugaggaaUu................................................    4          1          seq
    .....aaaaaacaaagUguugaggaagu................................................    1          1          seq
    .....aaaaaacaGagaguugaggaagu................................................    2          1          seq
    .....aGaaaacaaagaguugaggaagu................................................    1          1          seq
    .....aaaaaacaaagaguugaggaagC................................................    6          1          seq
    .....aaaaaacaaagaguugaggaagA................................................    1          1          seq
    .....aaaaaacaaagaguugaggaagu................................................    7          0          seq
    .....aaaaaacaaagaguugaggaaguu...............................................    2          0          seq
    .....aaaaaacaaagaguugaggaaguC...............................................    1          1          seq
    ......aaaaacaaagaguugagA....................................................    1          1          seq
    ......aaaaacaaagagCugagga...................................................    1          1          seq
    ......aaaaacaaagaguugagga...................................................    18         0          seq
    ......aaaaacaaagaguugaggG...................................................    1          1          seq
    ......aaaaacaaagaguugaggaa..................................................    1          0          seq
    ......aaaaacaaagaguugaggaag.................................................    21         0          seq
    ......aaaaacaaagaguugaggaaA.................................................    1          1          seq
    ......aaaaacaaagagGugaggaag.................................................    1          1          seq
    ......aaaaacaaagaguCgaggaagu................................................    1          1          seq
    ......aaaaacaaagaguugaggaagA................................................    4          1          seq
    ......aaaaacaaagaguugaUgaagu................................................    1          1          seq
    ......aaaaacaaagaguugaggaagC................................................    17         1          seq
    ......aGaaacaaagaguugaggaagu................................................    2          1          seq
    ......aaaaacaaGgaguugaggaagu................................................    1          1          seq
    ......aaaaacaaagaguugaggaagG................................................    1          1          seq
```

```
                                  novel-nve-miR-66_star
      novel-nve-miR-66_guide
acaacaaaaaacaaagaguugaggaaguuaaaacuuccucaacucuuuguuuuuuguuguuguuuuccuccuaaa

......aaaaGcaaagaguugaggaagu...............................................   2    1    seq
......aaaaacaaagaguugGggaagu...............................................   2    1    seq
......aaaaacaaagaguugaggaGgu...............................................   2    1    seq
......aaaaacaaagaguuUaggaagu...............................................   1    1    seq
......aaaaacaaagaguuCaggaagu...............................................   1    1    seq
......aaaaacUaagaguugaggaagu...............................................   1    1    seq
......aaaaacaaagaguugaggaagu...............................................   110  0    seq
......aaaaacaGagaguugaggaagu...............................................   4    1    seq
.......aaaacaaagaguugaggaagu...............................................   1    0    seq
..................................uuccucaacucuuuguuuuuA....................   1    1    seq
..................................uuccucaacucuuuguuuuuuA...................   9    1    seq
..................................uuccuUaacucuuuguuuuuug...................   1    1    seq
..................................uuccucaacucCuuguuuuuug...................   1    1    seq
..................................uuccucaacucuuuguuuuuCg...................   1    1    seq
..................................uuccucaacucuuuguuuuuug...................   36   0    seq
..................................uuccucaacucuuuguuCuuug...................   1    1    seq
..................................uuccucGacucuuuguuuuuugu..................   3    1    seq
..................................uuccucaacucuuuguuuuuugu..................   1    0    seq
..................................uuccucaacucuuuguuuuuuguA.................   3    1    seq
...................................uccucaacucuuuguuuuuu....................   2    0    seq
...................................uccucaacucuuuguuuuuug...................   3    0    seq
...................................uccucaacucuuuguuuuuugu..................   4    0    seq
...................................uccucaacucuuuguuuuuugC..................   2    1    seq
...................................uccucaacucuuuguuuuuugA..................   1    1    seq
```

miRBase precursor : novel-nve-miR-68
Total read count : 1570
novel-nve-miR-68_guide read count : 21
novel-nve-miR-68_star read count : 1549
remaining reads : 0

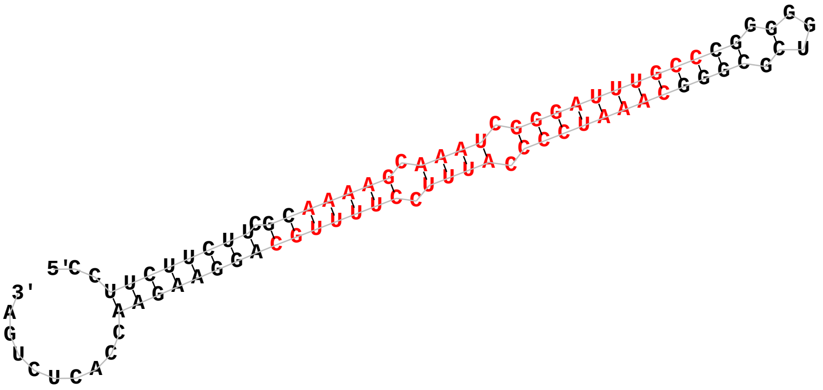

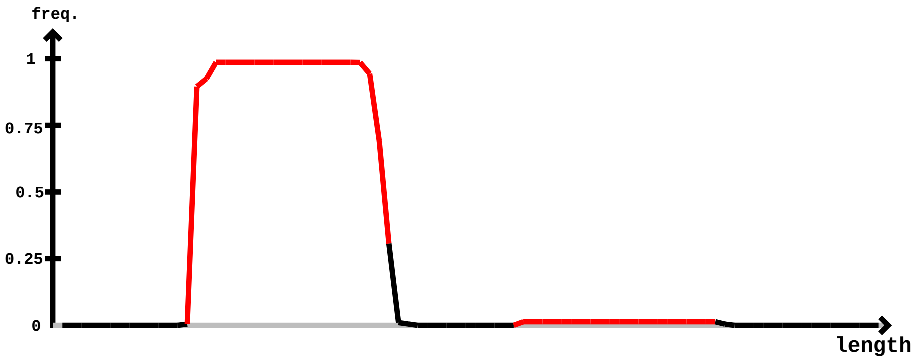


```
                                                     novel-nve-miR-68_guide
             novel-nve-miR-68_star
5'- ccuucuucuucgcaaaagcaaaucgggauuugcccgggggucgcgggcaaauccccauuuccuuuugcaggaagaaccacucuga  -3'    exp
    ..((((((((.(((((((.((((.((((((((((((.(...).)))))))))))).)))).))))))))))))))).........    reads      mm         sample
    ............caaaagGaaaucgggauu.......................................................    1          1          seq
    ............caaaagGaaaucgggauuugc....................................................    1          1          seq
    ............caaaagGaaaucgggauuugcc...................................................    5          1          seq
    .............aaaagGaaaucgggauu.......................................................    66         1          seq
    .............aaaagcaaaucgggauuug.....................................................    2          0          seq
    .............aaaagGaaaucgggauuug.....................................................    386        1          seq
    .............aaaagcaaaucgggauuugc....................................................    1          0          seq
    .............aaaagGaaaucgggauuugc....................................................    522        1          seq
    .............aaaagAaaaucgggauuugcc...................................................    1          1          seq
    .............aaaagGaaaucgggauuugcc...................................................    412        1          seq
    .............aaaagcaaaucgggauuugcc...................................................    1          0          seq
    .............aaaagGaaaucgggauuugccc..................................................    7          1          seq
    ..............aaagGaaaucgggauuug.....................................................    9          1          seq
    ..............aaagGaaaucgggauuugc....................................................    19         1          seq
    ..............aaagGaaaucgggauuugcc...................................................    16         1          seq
    ..............aaagGaaaucgggauuugcccg.................................................    2          1          seq
    ...............aagAaaaucgggauuugc....................................................    3          1          seq
    ...............aagGaaaucgggauuugc....................................................    58         1          seq
    ...............aagGaaaucgggauuugcc...................................................    29         1          seq
    ...............aagAaaaucgggauuugcc...................................................    1          1          seq
    ...............aagGaaaucgggauuugccc..................................................    1          1          seq
    ...............aagGaaaucgggauuugcccg.................................................    6          1          seq
    ...............................................caaauccccauuuccuuuugU.................    3          1          seq
    ...............................................caaauccccauuuccuuuugc.................    9          0          seq
    ...............................................caaauccccauuuccCuuugc.................    1          1          seq
    ...............................................caaauccccauuuccuuuugA.................    1          1          seq
    ...............................................caaauccccauuucUuuuugca................    1          1          seq
    ...............................................caaauccccauuuccuuuugcU................    3          1          seq
    ...............................................caaauccccauuuccuuuugca................    3          0          seq
```

miRBase precursor : novel-nve-miR-69
Total read count : 468
novel-nve-miR-69_guide read count : 468
novel-nve-miR-69_star read count : 0
remaining reads : 0

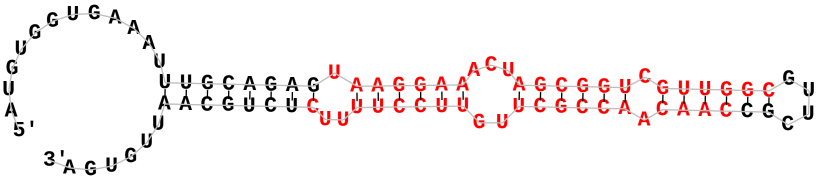

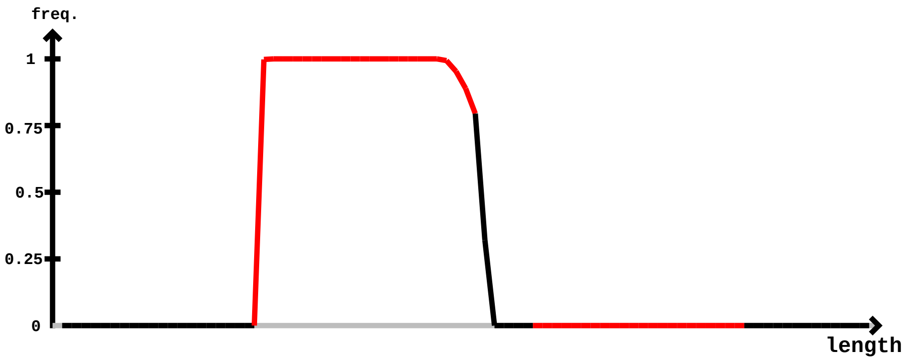


```
                                 novel-nve-miR-69_guide
                                                       novel-nve-miR-69_star
5'- auguggugaaauuugcagaguaaggaaacuagcggucguuggcguucgccaacaaccgcuuguuccuuuucucugcaauuguga  -3'   exp
    ............((((((((.((((((...((((((.((((((....)))))).))))))..))))))..))))))))......    reads   mm   sample
    ....................uaaggaaacuagcggucgu.............................................    3       0    seq
    ....................uaaggaaacuagcggucguu............................................    16      0    seq
    ....................uaaggaaacuagcggucguC............................................    3       1    seq
    ....................uaaggaaacuagcggucguuU...........................................    1       1    seq
    ....................uaaggaaacuagcggucguug...........................................    25      0    seq
    ....................uaaggaaacuagcggucguuA...........................................    2       1    seq
    ....................uaaggaaacuagcAgucguug...........................................    2       1    seq
    ....................uaaggaaacuagcggucguugg..........................................    35      0    seq
    ....................uaaggaaacuagcggucguugA..........................................    4       1    seq
    ....................uaaggaaacuagcggucguugU..........................................    2       1    seq
    ....................uaaggaaacuagcggucguuAg..........................................    1       1    seq
    ....................uaaggaaacuagcggucguGgg..........................................    1       1    seq
    ....................uaaggaaacuagcggucguugC..........................................    1       1    seq
    ....................uaaggaaacuagcggucguuAgc.........................................    2       1    seq
    ....................uaaggaaacuGgcggucguuggc.........................................    1       1    seq
    ....................uaaggaaacuagcggucguuggU.........................................    56      1    seq
    ....................uaaggaaacuagcggucguuggG.........................................    1       1    seq
    ....................uaaggaaacuagcggucguuggA.........................................    9       1    seq
    ....................uaaggUaacuagcggucguuggc.........................................    1       1    seq
    ....................Aaaggaaacuagcggucguuggc.........................................    1       1    seq
    ....................uaaggaaacuagcggucguuggc.........................................    138     0    seq
    ....................uaaggGaacuagcggucguuggc.........................................    4       1    seq
    ....................uGaggaaacuagcggucguuggc.........................................    2       1    seq
    ....................uaaggaaaUuagcggucguuggc.........................................    1       1    seq
    ....................Gaaggaaacuagcggucguuggc.........................................    1       1    seq
    ....................uaaggaaacuagcgAucguuggc.........................................    1       1    seq
    ....................uaGggaaacuagcggucguuggc.........................................    1       1    seq
    ....................Caaggaaacuagcggucguuggc.........................................    2       1    seq
    ....................uaaggaaacuagcggucguuggcg........................................    6       0    seq
    ....................uaaggaaacuagcggucguuggcU........................................    109     1    seq
    ....................uaaggaaacuagcggucguuggcA........................................    20      1    seq
    ....................uaaggaaacuagcggucguuggcC........................................    14      1    seq
    ....................uGaggaaacuagcggucguuggcg........................................    1       1    seq
    .....................aaggaaacuagcggucguuggc.........................................    1       0    seq
```

novel-nve-miR-69_guide

novel-nve-miR-69_star

auguggugaaauuugcagaguaaggaaacuagcggucguuggcguucgccaacaaccgcuuguuccuuuucucugcaauuguga

Provisional ID		novel-nve-miR-71
Score total	:	5.7
Score for star read(s)	:	-1.3
Score for read counts	:	0
Score for mfe	:	2.4
Score for randfold	:	1.6
Score for cons. seed	:	3
Total read count	:	1082
Mature read count	:	1080
Loop read count	:	0
Star read count	:	2

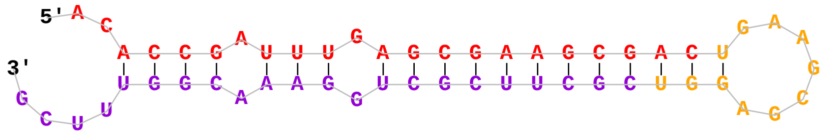

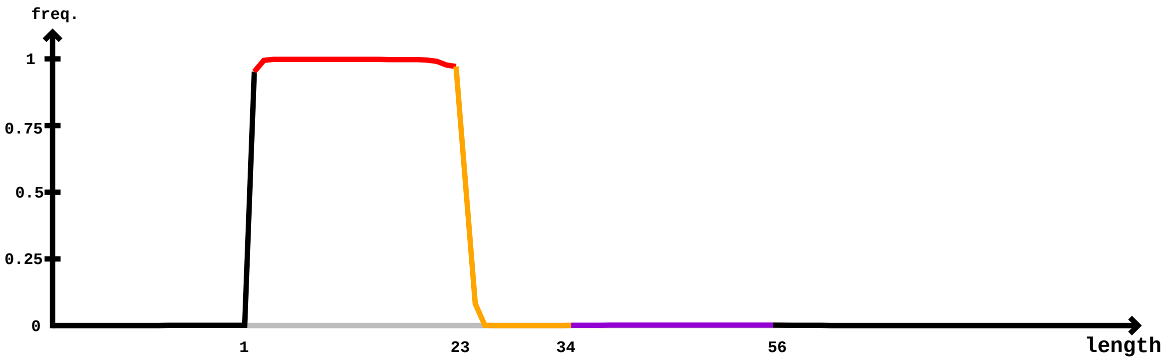

Mature Star

```
5'- ggcuccgagcuucgcggcgacaccgauuugagcgaagcgacugaagcgaggucgcuucgcuggaaacgguuucgccucggagccucauuaagaaccuucgauuuggucacu  -3'  obs
    ggcuccgagcuucgcggcgacaccgauuugagcgaagcgacugaagcgaggucgcuucgcuggaaacgguuucgccucggagccucauuaagaaccuucgauuuggucacu       exp
    ((((((((.......(((((.((((.(((.((((((((((((.......)))))))))))).))).)))).))))))))))))).......((.((........))))...   reads  mm  sample
    ..........uucgcggcgacaGcgauuugagc..............................................................................   1      1   seq
    ...................acaccgauuugagcgaag..........................................................................   2      0   seq
    ...................acaccgauuugagcgaagc.........................................................................   3      0   seq
    ...................acaccgauuugagcgaagU.........................................................................   1      1   seq
    ...................Gcaccgauuugagcgaagcg........................................................................   1      1   seq
    ...................acaccgauuugagcgaagcA........................................................................   1      1   seq
    ...................acaccgauuugaAcgaagcg........................................................................   1      1   seq
    ...................acaccgauuugagcgaagcg........................................................................   12     0   seq
    ...................acaccgauuugagcgaagcga.......................................................................   2      0   seq
    ...................Ccaccgauuugagcgaagcgac......................................................................   1      1   seq
    ...................acaccgauuugaCcgaagcgac......................................................................   1      1   seq
    ...................Ucaccgauuugagcgaagcgac......................................................................   1      1   seq
    ...................acaccgauuugagcgGagcgac......................................................................   1      1   seq
    ...................acaAcgauuugagcgaagcgac......................................................................   2      1   seq
    ...................acGccgauuugagcgaagcgac......................................................................   4      1   seq
    ...................aAaccgauuugagcgaagcgac......................................................................   1      1   seq
    ...................acaccgaAuugagcgaagcgac......................................................................   1      1   seq
    ...................acaccgUuuugagcgaagcgac......................................................................   1      1   seq
    ...................acaccgauuugagcgaagcgaA......................................................................   1      1   seq
    ...................acaccgGuuugagcgaagcgac......................................................................   1      1   seq
    ...................Gcaccgauuugagcgaagcgac......................................................................   8      1   seq
    ...................aUaccgauuugagcgaagcgac......................................................................   1      1   seq
    ...................acaccgaCuugagcgaagcgac......................................................................   2      1   seq
    ...................acaccgauuugagcgaagcgac......................................................................   507    0   seq
    ...................acaccgauuugagcgaagcgGc......................................................................   2      1   seq
    ...................acaccAauuugagcgaagcgac......................................................................   5      1   seq
    ...................acaccgauAugagcgaagcgac......................................................................   1      1   seq
    ...................acaccgauuugGgcgaagcgac......................................................................   2      1   seq
    ...................acaccgauuugagcgaagUgac......................................................................   9      1   seq
    ...................acaccgauCugagcgaagcgac......................................................................   2      1   seq
    ...................acaccgauuugagUgaagcgac......................................................................   1      1   seq
    ...................acaccgauuCgagcgaagcgac......................................................................   1      1   seq
    ...................acaccgauuugagcgaagcgaU......................................................................   28     1   seq
```

```
                   Mature                          Star
ggcuccgagcuucgcggcgacaccgauuugagcgaagcgacugaagcgaggucgcuucgcuggaaacgguuucgccucggagccucauuaagaaccuucgauuuggucacu
...................acaccAauuugagcgaagcgacu.....................................................................	1	1	seq
...................acaccgauuugagcgaagcgacu.....................................................................	250	0	seq
...................acaccgauuugGgcgaagcgacu.....................................................................	1	1	seq
...................acaccgauuugagcgaagcgaAu.....................................................................	1	1	seq
...................acaccgauuugagcgaagcgacA.....................................................................	15	1	seq
...................acaccgaAuugagcgaagcgacu.....................................................................	1	1	seq
...................acaccgauuugagcgGagcgacu.....................................................................	1	1	seq
...................acGccgauuugagcgaagcgacu.....................................................................	2	1	seq
...................acaccgauuugagcgaagcgaUu.....................................................................	1	1	seq
...................acaccgauuugagcgaagcgacC.....................................................................	41	1	seq
...................Gcaccgauuugagcgaagcgacu.....................................................................	7	1	seq
...................acacAgauuugagcgaagcgacu.....................................................................	1	1	seq
...................acaccgaCuugagcgaagcgacu.....................................................................	1	1	seq
...................acaccgauCugagcgaagcgacu.....................................................................	1	1	seq
...................Ccaccgauuugagcgaagcgacu.....................................................................	1	1	seq
...................acaccgauuugagUgaagcgacu.....................................................................	4	1	seq
...................acaccgauuCgagcgaagcgacu.....................................................................	1	1	seq
...................acaccgauuugagcgaagcgacG.....................................................................	1	1	seq
...................acaccgauuugagcgaagcgGcu.....................................................................	4	1	seq
...................acaccgauuugagcgaagcgacuA....................................................................	2	1	seq
...................acaccgauuugagcgaagcgacuU....................................................................	76	1	seq
...................acaccgauuugagcgaagcgacug....................................................................	2	0	seq
...................acaccgauuugagcgaagcgacuC....................................................................	7	1	seq
...................acaccgauuugagcgaagcgacuUa...................................................................	1	1	seq
....................caccgauuugagcgaagc.........................................................................	1	0	seq
....................caccgauuugagcgaagcga.......................................................................	4	0	seq
....................caccgauuugagcgaagcgac......................................................................	16	0	seq
....................caccgauuugagcgaagcgaU......................................................................	1	1	seq
....................caccgauuugagcgaagcgacu.....................................................................	22	0	seq
....................caccgauuugagcgaagcgacC.....................................................................	2	1	seq
.....................accgauuugagcgaagcgacu.....................................................................	1	0	seq
.....................accgauuugagcgaGgcgacu.....................................................................	1	1	seq
.....................accgauuugagcgaagcgacG.....................................................................	1	1	seq
.....................accgauuugagcgaagcgacuA....................................................................	1	1	seq
....................................................cgcuucgcuggaaacgguuucg.....................................	1	0	seq
........................................................ucgcuggaaacgguuucgccucC................................	1	1	seq
```

miRBase precursor : novel-nve-miR-72
Total read count : 69
novel-nve-miR-72_guide read count : 62
novel-nve-miR-72_star read count : 7
remaining reads : 0

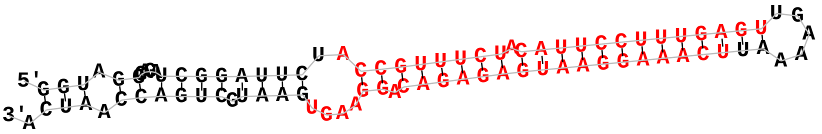

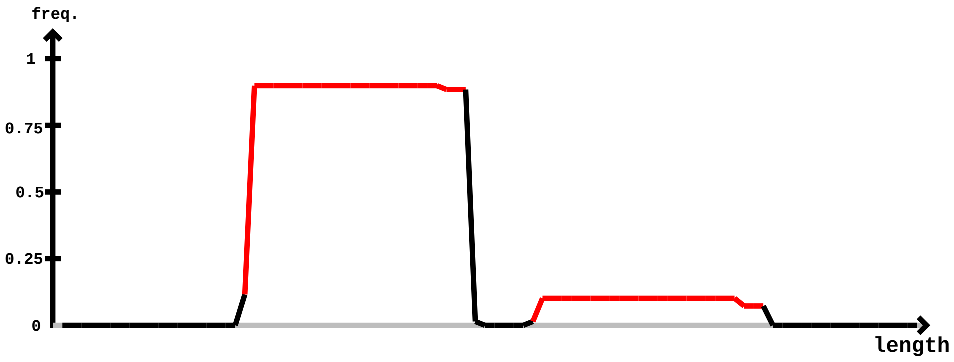

novel-nve-miR-72_guide
novel-nve-miR-72_star

```
5'- gguagggagaucggauucuaccguuucuacauuccuuugaguugaaaauucaaaggaaugagagacaggaagugaaugcugaccaauca   -3'    exp
    (((.((....((((((((..((((((((.(((((((((((((.....)))))))))))))))))))).))....)))).)))))).))).    reads    mm    sample
    ..................uaccguuucuacauuccuuug..................................................    1        0     seq
    ..................uaccguuucuacauuccuuugagu...............................................    5        0     seq
    ..................uaccguuucuacauuccuuugagC...............................................    2        1     seq
    ...................Gccguuucuacauuccuuugagu...............................................    1        1     seq
    ...................accguuucuacauuccuuugagA...............................................    1        1     seq
    ...................accguuucuacauuccuuugagu...............................................    32       0     seq
    ...................accguuucuacauuccCuugagu...............................................    3        1     seq
    ...................accguuucuaAauuccuuugagu...............................................    1        1     seq
    ...................aUcguuucuacauuccuuugagu...............................................    1        1     seq
    ...................accguuucuacauuccuuugagG...............................................    1        1     seq
    ...................accguuucuacauuccuuugagC...............................................    12       1     seq
    ...................accguuucuacauuccuuugGgu...............................................    1        1     seq
    ...................accguuucuacauuccuuugaguG..............................................    1        1     seq
    ................................................uucaaaggaaugagagacaggU...................    1        1     seq
    .................................................ucaaaggaaugagagacaggU...................    1        1     seq
    .................................................ucaaaggaaugagagacaggaagG................    1        1     seq
    .................................................ucaaaggaaugagagacaggaaUu................    1        1     seq
    .................................................ucaaaggaaugagagacaggaagu................    3        0     seq
```

miRBase precursor : novel-nve-miR-73
Total read count : 270
novel-nve-miR-73_guide read count : 259
novel-nve-miR-73_star read count : 6
remaining reads : 5

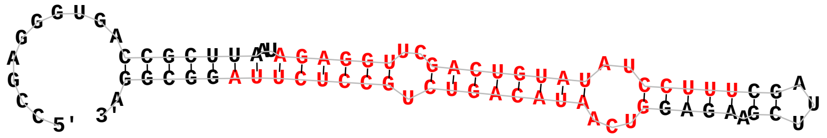

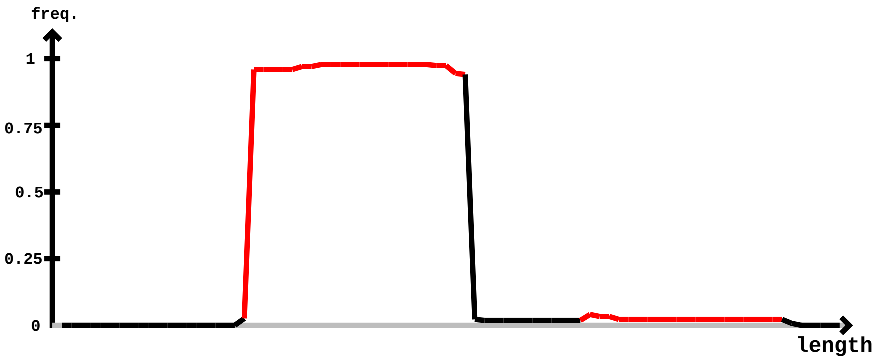


```
                                                        novel-nve-miR-73_star
                       novel-nve-miR-73_guide
5'- ccgagggugaccgcuuaauagagguucgacuguauauccuuucgauucgaagaggucaauacagucugccucuuaggcgga   -3'    exp
    ..........(((((((..((((((..((((((((..(((((((...)).)))))...)))))))).))))))))))))).    reads   mm   sample
    ..................uagagguucgacuguauauccu.........................................    1       0    seq
    ..................uagagguucgacuguauauccC.........................................    1       1    seq
    ..................uagagguucgacuguauauccuuu.......................................    5       0    seq
    ...................agagguucgacuguauauc...........................................    1       0    seq
    ...................agagguucgacuguauauccA.........................................    1       1    seq
    ...................agagguucgacuguauauccu.........................................    5       0    seq
    ...................agagguucgacuguauauccuu........................................    1       0    seq
    ...................agagguucgGcuguauauccuuu.......................................    2       1    seq
    ...................agagguucgacAguauauccuuu.......................................    2       1    seq
    ...................agagguucgacuguauaucUuuu.......................................    2       1    seq
    ...................agagguucgacuguauauccuuA.......................................    6       1    seq
    ...................agagguCcgacuguauauccuuu.......................................    1       1    seq
    ...................agagguucgacuguauauccuuu.......................................    195     0    seq
    ...................agagAuucgacuguauauccuuu.......................................    1       1    seq
    ...................agagguuUgacuguauauccuuu.......................................    3       1    seq
    ...................agagguucgacuguauaCccuuu.......................................    1       1    seq
    ...................agagguucgacuguauauccuuG.......................................    1       1    seq
    ...................Ggagguucgacuguauauccuuu.......................................    4       1    seq
    ...................agagguucgacuguauauccuCu.......................................    1       1    seq
    ...................agGgguucgacuguauauccuuu.......................................    1       1    seq
    ...................agagguucUacuguauauccuuu.......................................    1       1    seq
    ...................agaggCucgacuguauauccuuu.......................................    1       1    seq
    ...................agagguucgacuguauauccuuC.......................................    20      1    seq
    ...................agaggGucgacuguauauccuuu.......................................    1       1    seq
    ...................agagguucgacuguauauccuuuU......................................    1       1    seq
    ........................uucgacuguauauccuuucgauucgaagagguc........................    3       0    seq
    ..........................cgacuguauauccuuucgauucgaagagU..........................    1       1    seq
    ..........................cgacuguauauccuuCcgauucgaagagg..........................    1       1    seq
    ......................................................gucaauacagucugccucuuU......    3       1    seq
    ......................................................gucaauacagucugccucuua......    1       0    seq
    ......................................................gucaauacagucugccucuuaU.....    2       1    seq
```

miRBase precursor : novel-nve-miR-75
Total read count : 246
novel-nve-miR-75_guide read count : 245
novel-nve-miR-75_star read count : 1
remaining reads : 0

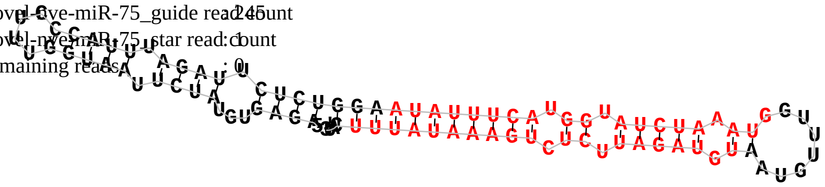

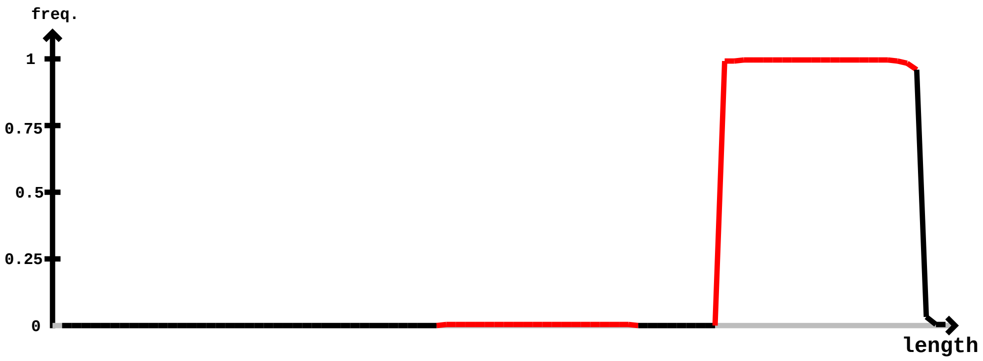


```
                                                            novel-nve-miR-75_star
                                                                                      novel-nve-miR-75_guide
5'- agaguguaucuuaaugguuucccauuuagauucucuggaauauuucaugguaucuaaaugguuguaauguagauucucugaaauauuucau  -3'    exp
    ((((...((((.(((((....))))).)))).))))(((((((((((.((.(((((.((........)).))))).)).))))))))))).. reads  mm   sample
    .......................................auauuucaugguaucuaaau................................ 1      0    seq
    ...................................................................uguagauucucugaaaua...... 1      0    seq
    ...................................................................uguagauucucugaaauaG..... 1      1    seq
    ...................................................................uguagauucucugaaauau..... 1      0    seq
    ...................................................................uguagauucucugaaauauu.... 5      0    seq
    ...................................................................uguagauucucugaaauauC.... 1      1    seq
    ...................................................................Aguagauucucugaaauauuu... 1      1    seq
    ...................................................................uguagauucucugaaauauuu... 188    0    seq
    ...................................................................ugCagauucucugaaauauuu... 1      1    seq
    ...................................................................uguagauuAucugaaauauuu... 1      1    seq
    ...................................................................uguagGuucucugaaauauuu... 1      1    seq
    ...................................................................uguagauucucugaaauauCu... 2      1    seq
    ...................................................................uguagauucuUugaaauauuu... 1      1    seq
    ...................................................................uguagauucAcugaaauauuu... 1      1    seq
    ...................................................................uguagauucucugaaGuauuu... 3      1    seq
    ...................................................................uguagauucucugaUauauuu... 1      1    seq
    ...................................................................uguagauCcucugaaauauuu... 1      1    seq
    ...................................................................uguagauucucugaaauauuA... 2      1    seq
    ...................................................................uguagauucucugaaauauuG... 1      1    seq
    ...................................................................uguagauucucugaaauauuC... 24     1    seq
    ...................................................................uguagauucucugaaauauuuA.. 4      1    seq
    ...................................................................uguagauucucugaaauauuuU.. 2      1    seq
    ...................................................................uguagauucucugaaauauuuc.. 1      0    seq
    .....................................................................uagauucucugaaauauuucau 1      0    seq
```

miRBase precursor : novel-nve-miR-76
Total read count : 69
novel-nve-miR-76_guide read count : 68
novel-nve-miR-76_star read count : 1
remaining reads : 0

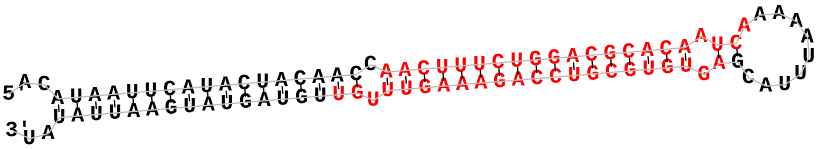

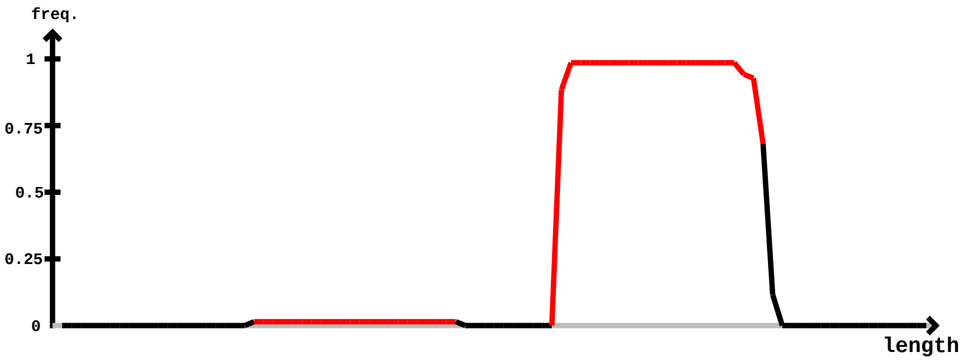


```
                                                    novel-nve-miR-76_guide
                          novel-nve-miR-76_star
5'- acauaauucauacuacaaccaacuuucuggacgcacaaucaaaaauuuacgagugugcguccagaaaguuuguuguaguaugaauuauau   -3'    exp
    ..(((((((((((((((((.(((((((((((((((((.((..........)).))))))))))))))))).)))))))))))))))))..     reads    mm    sample
    ...................caacuuucuggacgcacaauca.................................................     1        0     seq
    ...................................................aUugugcguccagaaaguuug..................     1        1     seq
    ...................................................agugugcguccagaaaguuug..................     15       0     seq
    ...................................................agugugcAuccagaaaguuug..................     1        1     seq
    ...................................................agugugcguccagaaGguuugu.................     1        1     seq
    ...................................................agugugcguccagaaaguuugG.................     1        1     seq
    ...................................................agugugcguccagaaaguuugC.................     4        1     seq
    ...................................................agugugcguccUgaaaguuugu.................     1        1     seq
    ...................................................agugugcgucUagaaaguuugu.................     1        1     seq
    ...................................................agugugcguccagaaaguuugA.................     1        1     seq
    ...................................................agugugcguccagaaaguuugu.................     29       0     seq
    ...................................................agCgugcguccagaaaguuugu.................     1        1     seq
    ...................................................agugugcguccagaaaguuuguu................     5        0     seq
    ....................................................Uugugcguccagaaagu.....................     3        1     seq
    ....................................................Uugugcguccagaaaguu....................     1        1     seq
    ....................................................gugugcguccagaaaguuuguC................     1        1     seq
    ....................................................gugugcguccagaaaguuuguA................     2        1     seq
```

miRBase precursor : novel-nve-miR-79
Total read count : 298
novel-nve-miR-79_guide read count : 294
novel-nve-miR-79_star read count : 3
remaining reads : 1

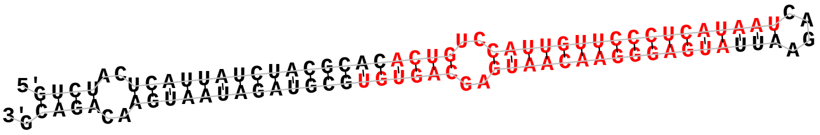

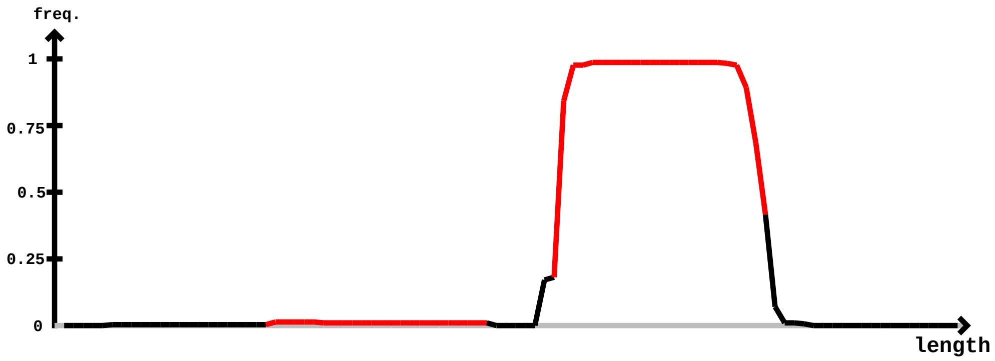

novel-nve-miR-79_star

novel-nve-miR-79_guide

5'- gucuacucauuaucuacgcacacuguccauuguucccucauaaucagaauuaugagggaacaaugagcagugugcguagauaaugaacagacg -3'	exp		
((((..(((((((((((((((((((..(((((((((((((((((....)))))))))))))))))..)))))))))))))))))))..)))).	reads	mm	sample
....acucauuaucuacgcacacugu...	1	0	seq
.....................acuguccauuguucccucauaau...	3	0	seq
....uuaugagggaacaaugagc.........................	1	0	seq
....uuaugagggaacaaugagcC........................	1	1	seq
....uuaugagggaacaaugagcGg.......................	1	1	seq
....uuaugagggaacaaugagcag.......................	4	0	seq
....uuaugagggaacaaugagcagu......................	33	0	seq
....uuaugagggaacaaugagcagC......................	2	1	seq
....uCaugagggaacaaugagcagu......................	1	1	seq
....uAaugagggaacaaugagcagu......................	1	1	seq
....uuaugGgggaacaaugagcagu......................	1	1	seq
....uuaugagggaaAaaugagcagu......................	1	1	seq
....uuaugagggaacaaugagcagA......................	2	1	seq
....uuaugagggaacaaugagcagug.....................	1	0	seq
....uuaugagggaacaaugagcaguU.....................	2	1	seq
....Aaugagggaacaaugagcagu......................	1	1	seq
....uaugagggaacaaugagcagug.....................	2	0	seq
....augaggUaacaaugagca........................	1	1	seq
....augaCggaacaaugagcag.......................	1	1	seq
....augagggaacaaugagcag.......................	13	0	seq
....augagggaacaaugagcaA.......................	1	1	seq
....augagggaacaaugagcGgu......................	1	1	seq
....augagggaacaaugagcagC......................	2	1	seq
....augagggaaGaaugagcagu......................	1	1	seq
....augagggaacaaugagcagu......................	12	0	seq
....augagggaacaaugagUagu......................	1	1	seq
....augagggaacaaugagUagug.....................	2	1	seq
....augagggaaUaaugagcagug.....................	1	1	seq
....augagggaacaaugagcaguA.....................	5	1	seq
....augagggaacaaCgagcagug.....................	1	1	seq
....augagggGacaaugagcagug.....................	1	1	seq
....augagggaacaaugGgcagug.....................	1	1	seq
....augagggaacaaugagcagug.....................	48	0	seq
....augagUgaacaaugagcagug.....................	1	1	seq

```
                     novel-nve-miR-79_star
                                                   novel-nve-miR-79_guide
gucuacucauuaucuacgcacacuguccauuguucccucauaaucagaauuaugagggaacaaugagcagugugcguagauaaugaacagacg

...................................................augagggaacaaugagcaguU.....................    2     1     seq
...................................................auCagggaacaaugagcagug.....................    1     1     seq
...................................................augagggaacaaugagcagGg.....................    1     1     seq
...................................................augagggaacaaugagcUgugu....................    1     1     seq
...................................................augagggaacaaugagcagugu....................    71    0     seq
...................................................augagggaacaaAgagcagugu....................    1     1     seq
...................................................auAagggaacaaugagcagugu....................    1     1     seq
...................................................augagggaacaaugagcagugC....................    9     1     seq
...................................................augagggaacGaugagcagugu....................    1     1     seq
...................................................augagggaacaGugagcagugu....................    1     1     seq
...................................................augagggaacaaugagcagGgu....................    1     1     seq
...................................................augagggaacaaugagcagugA....................    1     1     seq
...................................................augagggaacaaugagcagugG....................    1     1     seq
...................................................augagggaacaaugagcagugug...................    1     0     seq
...................................................augagggaacaaugagcaguguC...................    2     1     seq
...................................................augGgggaacaaugagcagugug...................    1     1     seq
...................................................augagggaacaaugagcaguguU...................    8     1     seq
....................................................ugagggaacaaugagcag.......................    5     0     seq
....................................................ugagggaacaaugagcagu......................    3     0     seq
....................................................ugagggaacaaugagcagug.....................    10    0     seq
....................................................ugagggaacaaugagcaguA.....................    1     1     seq
....................................................ugagggaacaaugagcagugu....................    15    0     seq
....................................................Agagggaacaaugagcagugug...................    1     1     seq
....................................................ugagggaacaaugagcaguguU...................    1     1     seq
....................................................ugagggaacaaugagcagugug...................    3     0     seq
....................................................ugagggaacaaugagcaguguC...................    1     1     seq
......................................................agggaacaaugagcagugugcA.................    1     1     seq
......................................................agggaacaaugagcagugugcgC................    1     1     seq
......................................................agggaacaaugagcagugugcgu................    1     0     seq
```

miRBase precursor : novel-nve-miR-83
Total read count : 312
novel-nve-miR-83_guide read count : 213
novel-nve-miR-83_star read count : 98
remaining reads : 1

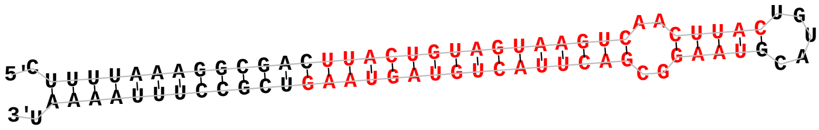

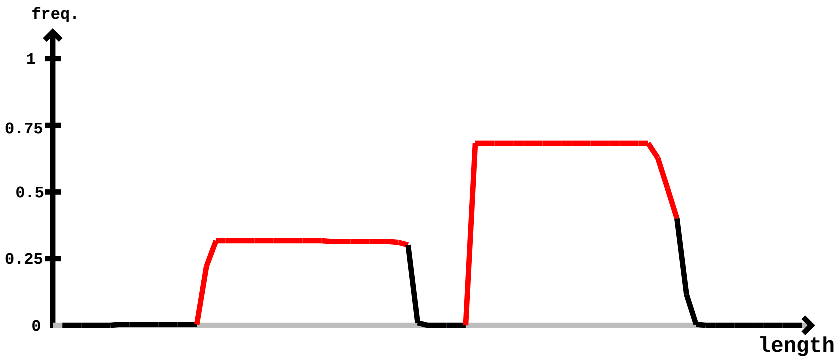

novel-nve-miR-83_star

novel-nve-miR-83_guide

```
5'- cuuuuaaaggcgacuuacuguaguaagucaacuuacuguacguaaggcgacuuacuguaguaagucgccuuuaaaau   -3'    exp
    .((((((((((((((((((((((((((((..(((((.....)))))..)))))))))))))))))))))))))))).    reads      mm         sample
    .....aaaggcgacuuacuguaguaag..................................................    1          0          seq
    ..............uuacuguaguaagucaacuu...........................................    1          0          seq
    ..............uuacuguaguaagucaacuua..........................................    1          0          seq
    ..............uuacuguagAaagucaacuuac.........................................    1          1          seq
    ..............uuacuguaguaagucaacuuaA.........................................    1          1          seq
    ..............uuacuguaguaagucaacuuaU.........................................    3          1          seq
    ..............uuacuguaguGagucaacuuac.........................................    1          1          seq
    ..............uuacuguaguaagucaacuuac.........................................    59         0          seq
    ..............uAacuguaguaagucaacuuacu........................................    1          1          seq
    ...............uacuguaguaagucaacuua..........................................    1          0          seq
    ...............Cacuguaguaagucaacuua..........................................    1          1          seq
    ...............uacuguaguaagucaacuuaU.........................................    2          1          seq
    ...............Aacuguaguaagucaacuuac.........................................    1          1          seq
    ...............uacuguaguaagucaCcuuac.........................................    1          1          seq
    ...............uacuguaguaagucaacuuac.........................................    22         0          seq
    ...............uacuguaguaagucaacuuacA........................................    1          1          seq
    ...............uacuguaguaagucaacuuacu........................................    1          0          seq
    ..........................................uaaggcgacuuacuguagC................    3          1          seq
    ..........................................uaaggcgacuuacuguagu................    12         0          seq
    ..........................................uaaggcgaUuuacuguagu................    1          1          seq
    ..........................................uaaggcgacuuacuguagG................    1          1          seq
    ..........................................uaaggcgacuuaAuguagua...............    1          1          seq
    ..........................................uaaggcgacuuacuguGgua...............    1          1          seq
    ..........................................uaaggcgGcuuacuguagua...............    1          1          seq
    ..........................................uaaggcgacuuacuguaguG...............    2          1          seq
    ..........................................uaaggcgacuuacuguagCa...............    1          1          seq
    ..........................................uaaggcgacuuacuguagua...............    29         0          seq
    ..........................................uaaUgcgacuuacuguaguaa..............    1          1          seq
    ..........................................uaaggcgacCuacuguaguaa..............    1          1          seq
    ..........................................uaaggcgacuuacuguaguaa..............    33         0          seq
    ..........................................uaaggcgacuuacuguaguaG..............    1          1          seq
    ..........................................uaaggcgacuuacuguaguaag.............    33         0          seq
    ..........................................uaaggAgacuuacuguaguaag.............    1          1          seq
    ..........................................uaaggcgacuuacuguaguaaA.............    6          1          seq
```

```
              novel-nve-miR-83_star
                                          novel-nve-miR-83_guide
cuuuuaaaggcgacuuacuguaguaagucaacuuacuguacguaaggcgacuuacuguaguaagucgccuuuaaaau

..........................................uaaggUgacuuacuguaguaag.............    1          1          seq
..........................................uaaggcgacuuacuguaguaaU.............    35         1          seq
..........................................uaaggcgacuuacCguaguaag.............    1          1          seq
..........................................uaaggcgacuuacuguaguaaC.............    12         1          seq
..........................................uaaggcgacuuacuguaguaagA............    3          1          seq
..........................................uaaggcgacuuacuguaguaaUu............    14         1          seq
..........................................uaaggcgacuuacuguaguaaAu............    1          1          seq
..........................................uaaggcgacuuacuguaguaagu............    10         0          seq
..........................................uaaggcgacuuacuguaguaaCu............    7          1          seq
..........................................uaaggcgacuuacuguaguaaguU...........    1          1          seq
```

miRBase precursor : novel-nve-miR-84
Total read count : 118
novel-nve-miR-84_guide read count : 90
novel-nve-miR-84_star read count : 24
remaining reads : 4

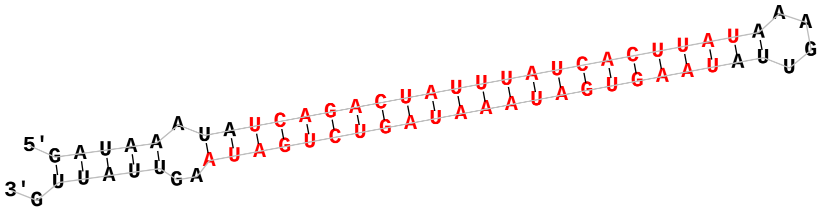

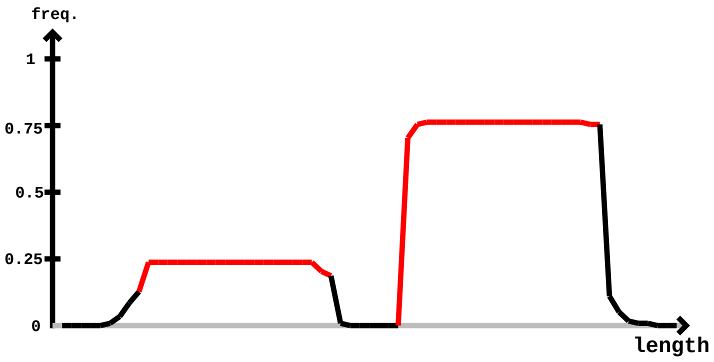

novel-nve-miR-84_guide
novel-nve-miR-84_star

```
5'- gauaaauaucagacuauuuaucacuuauaaaguuauaagugauaaaauagucugauaaguuauug   -3'    exp
    (((((.(((((((((((((((((((((((....))))))))))))))))))))))))..))))).   reads   mm   sample
    ....aauaucagacuauuuaucacuu.......................................   1       0    seq
    .....auaucagacuauuuaAcacuuau.....................................   1       1    seq
    .....Uuaucagacuauuuaucacuuau.....................................   1       1    seq
    .....auaucagacuauuuaucacuuauU....................................   1       1    seq
    ......uaucagacuauuuaucacuuau.....................................   6       0    seq
    .......auAagacuauuuaucacuu.......................................   1       1    seq
    .......aucagacuauuuaucacuu.......................................   1       0    seq
    .......aucagacuauuuaucacuuU......................................   1       1    seq
    .......aucagacuauuuaucacuuau.....................................   2       0    seq
    ........ucagacuauuuaucacuu.......................................   1       0    seq
    ........ucagacuauuuaucacuuU......................................   1       1    seq
    ........ucagacuauuuaucacuuaA.....................................   1       1    seq
    ........ucagacuauuuaucacuuaC.....................................   1       1    seq
    ........ucagacuauuuaucacuuau.....................................   9       0    seq
    ...................................uaagugauaaaauagucugC..........   1       1    seq
    ...................................uaagugauaaaauagucugauC........   4       1    seq
    ...................................uaagugauaaaauagucugauU........   51      1    seq
    ...................................uaagugauaaaauagucugaua........   14      0    seq
    ...................................uaagugauaaaauagucugauG........   1       1    seq
    ...................................uaagugauaaaauagucugauaU.......   1       1    seq
    ...................................uaagugauaaaauagucugauaa.......   4       0    seq
    ...................................uaagugauaaaauagucugauCa.......   2       1    seq
    ...................................uaagugauaaaauagucugauaaA......   1       1    seq
    ...................................uaagugauaaaauagucugauaag......   3       0    seq
    ...................................uaagugauaaaauagucugauaaguuU...   1       1    seq
    ....................................aagugauaaaauagucugauU........   2       1    seq
    ....................................aagugauaaaauagucugaua........   4       0    seq
    .....................................agugauaaaauagucugauaagu.....   1       0    seq
```

miRBase precursor : novel-nve-miR-85
Total read count : 391
novel-nve-miR-85_guide read count : 350
novel-nve-miR-85_star read count : 28
remaining reads : 13

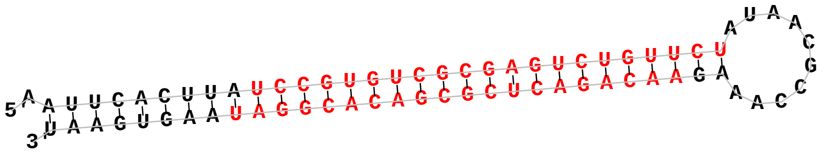

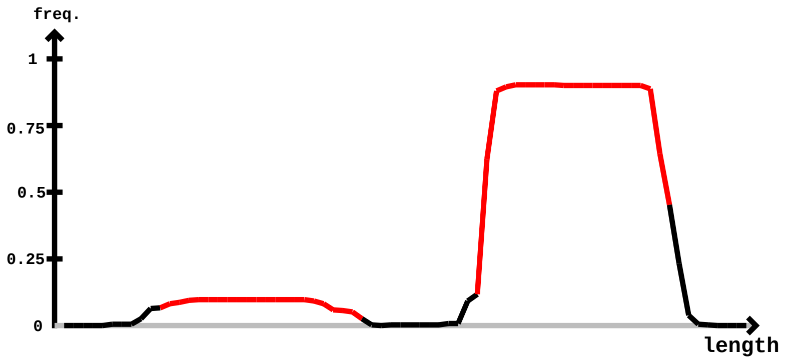

novel-nve-miR-85_star

novel-nve-miR-85_guide

5'- aauucacuuauccgugucgcgagucuguucuauaacgccaaagaacagacucgcgacacggauaagugaau -3'	exp		
.((((((((((((((((((((((((((((((..........))))))))))))))))))))))))))))))	reads	mm	sample
....caUuuauccgugucgcgaguc..	2	1	seq
.......uuauccgugucgcgagucu...	1	0	seq
.......uuauccgugucgcgagucC...	1	1	seq
.......uuauccgugucgcgagucug..	3	0	seq
.......uuauccgugucgUgagucug..	1	1	seq
.......uuauccgugucgcgaCucug..	1	1	seq
.......uuauccgugucgcgagucuguucu..	1	0	seq
........uauccgugucgcgagucu...	2	0	seq
........uUuccgugucgcgagucug..	2	1	seq
........uauccguguAgcgagucug..	1	1	seq
........uauccgugucgcgagucuA..	1	1	seq
........uauccguguAgcgagucugu...	1	1	seq
........uauccguguAgcgagucuguuc...	6	1	seq
........uauccgugucgcgagucuguuc...	1	0	seq
........uauccguguAgcgagucuguucu..	1	1	seq
.........auccgugucgcgagucuguuA...	1	1	seq
..........uccgugucgcgagucuguu..	2	0	seq
..........uccgugucgcgagucuguCcu..	1	1	seq
..........Accgugucgcgagucuguucu..	1	1	seq
..........uccgugucgcgagucuguucu..	2	0	seq
...........ccgugucgcgagucuguuc...	2	0	seq
............cgugucgcgagucuguucu..	1	0	seq
............cgugucgcAagucuguucu..	2	1	seq
.............gugucgcgagucuguucuU.......................................	1	1	seq
.................................aacgcUaaagaacagacu....................	1	1	seq
....aaagaacagacucgcgacacU...........	1	1	seq
....aaagaacagacucgcgacacC...........	1	1	seq
....agaacagacuUgcgacacg...........	1	1	seq
....agaacagacucgcgacacA...........	1	1	seq
....agaacagacucgcgacacg...........	1	0	seq
....agaacagacucgcgacacgg..........	15	0	seq
....agaacagacucgcgacacgU..........	1	1	seq
....agaacagacucgcgacacgA..........	4	1	seq
....agaacGgacucgcgacacgg..........	1	1	seq

```
          novel-nve-miR-85_star
                                         novel-nve-miR-85_guide
aauucacuuauccgugucgcgagucuguucuauaacgccaaagaacagacucgcgacacggauaagugaau

.........................................agaacagacucgAgacacgg..........    1    1    seq
.........................................agaacagacucgcgacacggC.........    1    1    seq
.........................................agaacagacucgcgacacggU.........    2    1    seq
.........................................Ugaacagacucgcgacacgga.........    1    1    seq
.........................................agaacagacucgcgacacggG.........    1    1    seq
.........................................agaacagacucgcgacacgga.........    3    0    seq
..........................................gaacagacuUgcgacacgg..........    1    1    seq
..........................................Aaacagacucgcgacacgg..........    1    1    seq
..........................................gaacagacucgcgacacgg..........    1    0    seq
..........................................gaacagacucgcgacacgC..........    1    1    seq
..........................................gaacagacucgcgacacgga.........    2    0    seq
..........................................gaacagacucgcgacacggU.........    2    1    seq
..........................................gaacagacucgcgacacggau........    1    0    seq
..........................................gaacagacucgcgacacggUu........    1    1    seq
...........................................Gacagacucgcgacacgg..........    1    1    seq
...........................................aacagaUucgcgacacgg..........    1    1    seq
...........................................aacagacucgcgacacgU..........    6    1    seq
...........................................aacagacucgcAacacgg..........    1    1    seq
...........................................aacagacucgcgacacgg..........    52   0    seq
...........................................aacagacucgcgacacCg..........    1    1    seq
...........................................aacagacucgcgacacgA..........    4    1    seq
...........................................aacagGcucgcgacacgg..........    1    1    seq
...........................................aacagacCcgcgacacgg..........    1    1    seq
...........................................aacGgacucgcgacacgg..........    1    1    seq
...........................................aacagacucgcgacacggC.........    1    1    seq
...........................................Cacagacucgcgacacgga.........    1    1    seq
...........................................aacaAacucgcgacacgga.........    1    1    seq
...........................................aacagacucgcgacacggU.........    10   1    seq
...........................................aacagacucgcgacacgga.........    31   0    seq
...........................................aacagacucgcgacacggG.........    3    1    seq
...........................................aacagacucgcgacacggaA........    2    1    seq
...........................................Gacagacucgcgacacggau........    1    1    seq
...........................................aacagacucgUgacacggau........    1    1    seq
...........................................aacagGcucgcgacacggau........    1    1    seq
...........................................aacaAacucgcgacacggau........    1    1    seq
...........................................aacagacucgcgacacggaC........    3    1    seq
...........................................aacagacucgcgacacggUu........    4    1    seq
...........................................aacagacucgcgacacggau........    35   0    seq
...........................................aacagacuUgcgacacggau........    1    1    seq
...........................................aacagacucgcgacacggauC.......    3    1    seq
...........................................aacagacucgcgacacggaCa.......    1    1    seq
...........................................aacagacuUgcgacacggaua.......    2    1    seq
...........................................aaUagacucgcgacacggaua.......    1    1    seq
...........................................aacagacucgcgacacggauU.......    24   1    seq
...........................................aacagacucgcgacacggaua.......    2    0    seq
............................................acagacAcgcgacacgga.........    1    1    seq
............................................aUagacucgcgacacgga.........    1    1    seq
............................................acagacucgcgacacgga.........    12   0    seq
............................................acagacucgcgacacggU.........    1    1    seq
............................................acagacucgcgacaUgga.........    1    1    seq
............................................acagacucgcgacGcggau........    1    1    seq
............................................acagacucgcgacacggaC........    1    1    seq
............................................acagacucgcgUcacggau........    1    1    seq
............................................acagacuAgcgacacggau........    1    1    seq
............................................acagacuUgcgacacggau........    3    1    seq
............................................acagacucgcgacacggaG........    2    1    seq
............................................acagacucgcgacacggCu........    2    1    seq
............................................acagacucgcgacacggau........    22   0    seq
............................................acagacucgcgacacAgaua.......    1    1    seq
............................................acagaUucgcgacacggaua.......    1    1    seq
............................................acagacucgcgacacggauU.......    11   1    seq
............................................acagacucgcgacacggauG.......    1    1    seq
............................................acagacucgcgacacggaua.......    24   0    seq
............................................acagacucgcgGcacggaua.......    1    1    seq
............................................acagacucgcgacacggauaa......    4    0    seq
............................................acagacucgcgacacggauaU......    6    1    seq
............................................acagacucgcgacacggauaG......    1    1    seq
............................................acagacucgcgacacggauaUg.....    1    1    seq
.............................................cagacucgcgacacggau........    2    0    seq
```

```
          novel-nve-miR-85_star
                                           novel-nve-miR-85_guide
aauucacuuauccgugucgcgagucuguucuauaacgccaaagaacagacucgcgacacggauaagugaau

.............................................cagacucgcgacacggaua.......    1          0          seq
.............................................cagacucgcgacacggauU.......    1          1          seq
.............................................Uagacucgcgacacggauaa......    1          1          seq
.............................................cagacucgcgacacggauUa......    1          1          seq
..............................................agacucgcgacGcggaua.......    2          1          seq
..............................................agacucgcgacacggauaaCu....    1          1          seq
```

miRBase precursor : novel-nve-miR-86
Total read count : 154
novel-nve-miR-86_guide read count : 126
novel-nve-miR-86_star read count : 21
remaining reads : 7

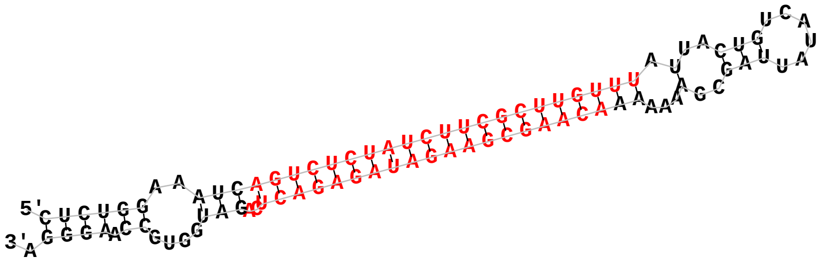

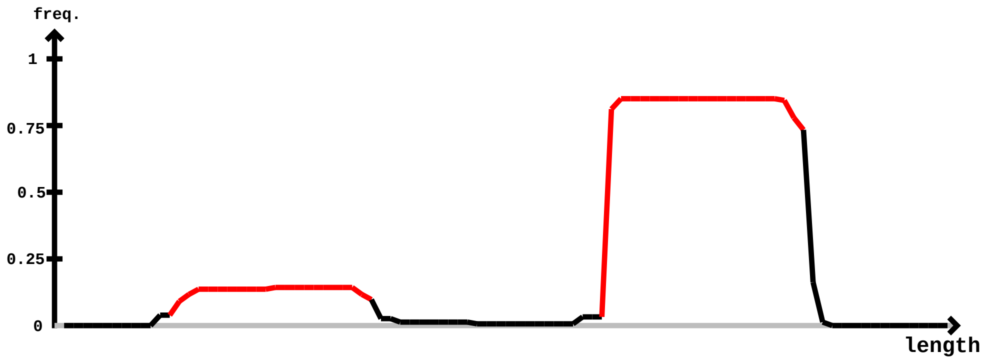


```
                                                                              novel-nve-miR-86_guide
               novel-nve-miR-86_star
5'- cucuggaaaucagucucuaucuucgcuuguuuauuacugucauauuagcgaaaaaaacaagcgaagauagagacucagauggugccaaggga  -3'  exp
    (((((..(((((((((((((((((((((((((.(..(((......)))..)...)))))))))))))))))))))..)))....)).)))).   reads   mm   sample
    .........uUagucucuaucuucgcuugu..............................................................   1       1    seq
    .........ucagucucuaucuucgcuugu..............................................................   2       0    seq
    .........ucagucucuaucuucgcuuguu.............................................................   3       0    seq
    ...........agucucuaucuucgcuugu..............................................................   1       0    seq
    ...........agucucuaucuucgcuuguuC............................................................   1       1    seq
    ...........agucucuaucuucgcCuguuu............................................................   1       1    seq
    ...........agucucuaucuucgcuuguuu............................................................   5       0    seq
    ............gucucuaucuucgcuuguuu............................................................   3       0    seq
    ............gucucuaucuucgcuuguCu............................................................   1       1    seq
    .............ucucuaucuucgcuuguuua...........................................................   1       0    seq
    .............ucucuaucuucgcuuguuuau..........................................................   1       0    seq
    .............ucucuaucuucgcuuguuuaC..........................................................   1       1    seq
    .....................uucgcuuguuuauuacuguca..................................................   1       0    seq
    .................................uuacugucauauuagcgaaU.......................................   1       1    seq
    .....................................................aaaacaagcgaagauagagac..................   1       0    seq
    .....................................................Uaaacaagcgaagauagagacu.................   3       1    seq
    .....................................................aaaacaagcgaagauagagacu.................   1       0    seq
    ........................................................acaagcgaagauagagacu.................   3       0    seq
    ........................................................acaagcgaagauagagacG.................   1       1    seq
    ........................................................acaagcgaagauagagacC.................   1       1    seq
    ........................................................acaagcgaagGuagagacu.................   1       1    seq
    ........................................................acaagcgaagauagagacuG................   1       1    seq
    ........................................................acaagcgaagauagagacuU................   3       1    seq
    ........................................................acaagcgaagauagagacuc................   2       0    seq
    ........................................................acaagcgaagauagagacGc................   1       1    seq
    ........................................................acaagcgaagauagagacuGa...............   4       1    seq
    ........................................................acaagcgaagauagagacucU...............   41      1    seq
    ........................................................acaagcgaagauagGgacuca...............   1       1    seq
    ........................................................acaagcgaagauagagacuca...............   28      0    seq
    ........................................................acaagcgaagauaCagacuca...............   1       1    seq
    ........................................................acaagcgaagauagagacucC...............   9       1    seq
    ........................................................acaagcgaagauagagacucG...............   3       1    seq
    ........................................................acaagcgaagauagagacucaU..............   7       1    seq
    ........................................................acaagcgaagauagagacucaA..............   3       1    seq
```

```
                                                       novel-nve-miR-86_guide
           novel-nve-miR-86_star
cucuggaaaucagucucuaucuucgcuuguuuauuacugucauauuagcgaaaaaaacaagcgaagauagagacucagauggugccaaggga

........................................................acaagcgGagauagagacucag..............    1           1           seq
........................................................acaagcgaagauagagacucaC..............    2           1           seq
........................................................acaagcgaagauagagacucag..............    7           0           seq
.........................................................caagcgaagauagagacuca...............    1           0           seq
.........................................................caagcgaagauagagacucaU..............    1           1           seq
.........................................................caagcgaagauagagacucag..............    2           0           seq
.........................................................caagcgaagauagagacucUga.............    2           1           seq
```

Provisional ID		novel-nve-miR-87
Score total	:	72.5
Score for star read(s)	:	3.9
Score for read counts	:	64.9
Score for mfe	:	2.7
Score for randfold	:	1.6
Score for cons. seed	:	-0.6
Total read count	:	139
Mature read count	:	107
Loop read count	:	0
Star read count	:	32

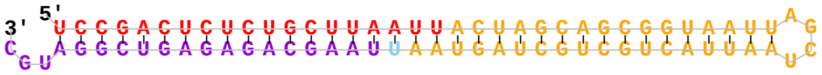

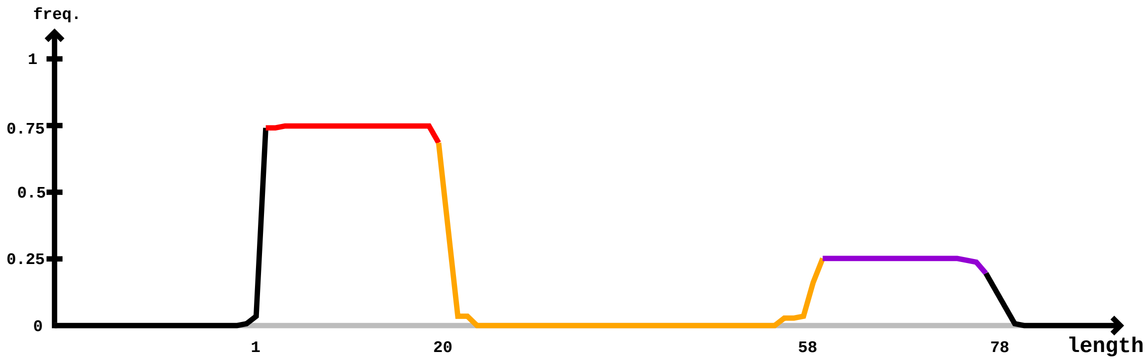

Mature   Star

```
5'- cuacgcuuucuauugaaacauccgacucucugcuuaauuacuagcagcgguaauuagcuaauuacugcugcuaguaauuaagcagagagucggaugcuucaacaacaau   -3'   obs
    cuacgcuuucuauugaaacauccgacucucugcuuaauuacuagcagcgguaauuagcuaauuacugcugcuaguaauuaagcagagagucggaugcuucaacaacaau         exp
    ............(((((.(((((((((((((((((((((((((((((((((((((....))))))))))))))))))))))))))))))))))))).)))))).......   reads   mm   sample
    ..................cauccgacucucugcuuaau.......................................................................   1       0    seq
    ...................auccgacucucugcuuaauu......................................................................   4       0    seq
    ....................uccgacucucugcuuaau.......................................................................   6       0    seq
    ....................uccgacucucugcuuaaC.......................................................................   2       1    seq
    ....................uccgacucucugcuCaauu......................................................................   1       1    seq
    ....................uccgacucucugcuuaauC......................................................................   11      1    seq
    ....................uccgacucucugcuuaaCu......................................................................   2       1    seq
    ....................uccgacucucugcuuaauu......................................................................   66      0    seq
    ....................uccgacucucugcuuaauua.....................................................................   3       0    seq
    ....................uccgacucucugcuuaauuU.....................................................................   6       1    seq
    ....................Accgacucucugcuuaauuacu...................................................................   1       1    seq
    ....................uccgacucucugcuuaauuacu...................................................................   2       0    seq
    ....................uccgacucucugcuuaauuacC...................................................................   1       1    seq
    ......................cgacucucugcuuaauuacu...................................................................   1       0    seq
    ..........................................................................uaauuaagcagagagucgg................   1       0    seq
    ..........................................................................uaauuaagcagagagucggU...............   1       1    seq
    ..........................................................................uaauuaagcagagagucggau..............   1       0    seq
    ..........................................................................uaauuaagcagagagucggauA.............   1       1    seq
    ............................................................................auuaagcagagagucggau..............   1       0    seq
    .............................................................................uuaagcagagagucggau..............   3       0    seq
    .............................................................................uuaagcagagagucggaA..............   1       1    seq
    .............................................................................uuaagcagagagucggauA.............   1       1    seq
    .............................................................................uuaagcagagagucggauU.............   5       1    seq
    .............................................................................uuaagcagagagucggaug.............   4       0    seq
    .............................................................................uuaagcagagagucggaugA............   1       1    seq
    .............................................................................uuaagcagagagucggauAc............   1       1    seq
    .............................................................................uuaagcagagagucggaugc............   1       0    seq
    .............................................................................uuaagcagagagucggaugcC...........   1       1    seq
    ..............................................................................uaagcagagagucAgaug.............   1       1    seq
    ..............................................................................uaagcagagagucggaugA............   3       1    seq
    ..............................................................................uaagcagagUgucggaugc............   1       1    seq
    ..............................................................................uaagcagagagucggaugc............   5       0    seq
    ..............................................................................uaagcagagagucggauUc............   1       1    seq
```

```
                   Mature                                                       Star
cuacgcuuucuauugaaacauccgacucucugcuuaauuacuagcagcgguaauuagcuaauuacugcugcuaguaauuaagcagagagucggaugcuucaacaacaau
..............................................................................uaagcagaUagucggaugc............    1          1          seq
..............................................................................uaagcagagagucggaugcGu...........    1          1          seq
```

miRBase precursor : novel-nve-miR-88
Total read count : 180
novel-nve-miR-88_guide read count : 162
novel-nve-miR-88_star read count : 18
remaining reads : 0

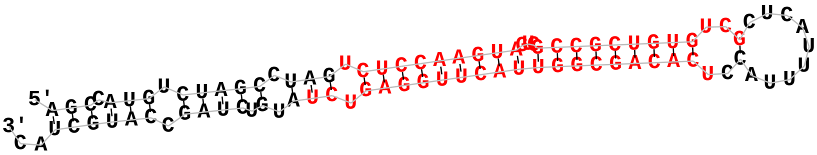

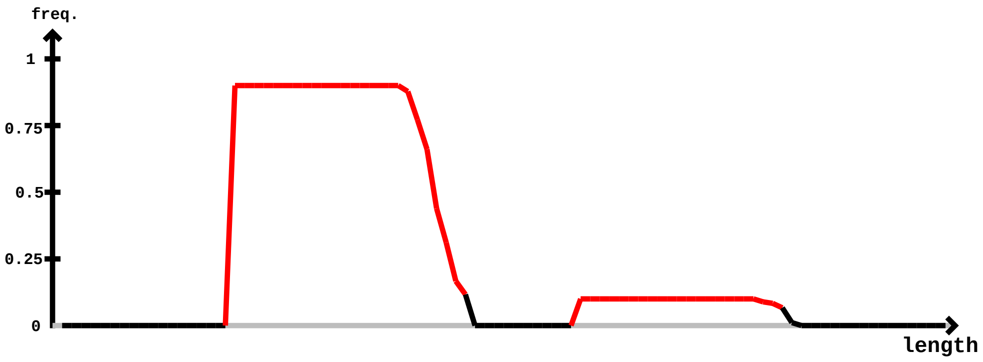


```
                                                                   novel-nve-miR-88_star
                novel-nve-miR-88_guide
5'- agccaugucuagccuagucuccaaguagucgccgcugugucgcucauuuuaccucacagcgguuacuuggagucuaugucuagccaugcuac   -3'   exp
    (((.(((.(((((.(((.(((((((((...((((((((..(.........)..))))))))))))))))).))).).)))).))))))..   reads   mm   sample
    .................ucuccaaguagucgccgU.........................................................   2       1    seq
    .................ucuccaaguagucgccgc.........................................................   1       0    seq
    .................ucuccaaguagucgcUgc.........................................................   1       1    seq
    .................ucuccaaguagucgccgcC........................................................   2       1    seq
    .................ucuccaaguagucgccgcG........................................................   2       1    seq
    .................ucuccaaguagucgccgcA........................................................   3       1    seq
    .................ucuccaaguagucgccgcu........................................................   12      0    seq
    .................ucuccaaguagucgccgcug.......................................................   17      0    seq
    .................Acuccaaguagucgccgcug.......................................................   1       1    seq
    .................ucuccaaguagucgccgcuA.......................................................   2       1    seq
    .................ucuccaaguagucgccgcugA......................................................   1       1    seq
    .................ucuccaaguagucgccgcugG......................................................   1       1    seq
    .................ucuccaaguagucgcAgcugu......................................................   1       1    seq
    .................ucuccaaguagucgccgcugC......................................................   6       1    seq
    .................ucuccaaguagucgAcgcugu......................................................   1       1    seq
    .................ucuccaaguagucgccgcugu......................................................   29      0    seq
    .................Gcuccaaguagucgccgcugu......................................................   1       1    seq
    .................ucuccaaguagucgccgcuguC.....................................................   1       1    seq
    .................ucuccaaguagucgccgcuguU.....................................................   3       1    seq
    .................ucuccaaguagucgccgcugug.....................................................   16      0    seq
    .................ucuccaaguagucgccgcuguA.....................................................   3       1    seq
    .................ucuccaaguagucgccgcuguUu....................................................   1       1    seq
    .................ucuccaaguagucgccgcugugC....................................................   3       1    seq
    .................ucuccaaguagucgccgcugugA....................................................   1       1    seq
    .................ucuccaaguagucgccgcuguAu....................................................   1       1    seq
    .................Acuccaaguagucgccgcugugu....................................................   1       1    seq
    .................ucuccaaguagucgccgcugugu....................................................   19      0    seq
    .................ucuccaaguagucgccgcuguguG...................................................   1       1    seq
    .................ucuccaaguagucgccgcuguguU...................................................   3       1    seq
    .................ucuccaaguagucgccgcuguguc...................................................   4       0    seq
    .................ucuccaaguagucgGcgcuguguc...................................................   1       1    seq
    .................ucuccaagAagucgccgcugugucg..................................................   1       1    seq
    .................ucuccaaguagucgccgcugugucU..................................................   4       1    seq
    .................ucuccaaguagucgccgcugugucg..................................................   13      0    seq
```

```
                                                    novel-nve-miR-88_star
                 novel-nve-miR-88_guide
agccaugucuagccuagucuccaaguagucgccgcugugucgcucauuuuaccucacagcgguuacuuggagucuaugucuagccaugcuac

.................ucuccaaguagucgccgcugugucA.................................................    3    1    seq
.....................................................ucacagcgguuacuuggaU....................    1    1    seq
.....................................................ucacagcgguuacuuggag....................    1    0    seq
.....................................................ucacagcgguuacuuggagu...................    1    0    seq
.....................................................ucacagcgguuacuuggaguU..................    1    1    seq
.....................................................ucacagcgguuacuuggaguc..................    2    0    seq
.....................................................ucacagcgguuacuuggagucu.................    9    0    seq
.....................................................ucacagcgguuacuuggagucA.................    1    1    seq
.....................................................ucacagcgguuacuuggagucuU................    2    1    seq
```

miRBase precursor : novel-nve-miR-89
Total read count : 1076
novel-nve-miR-89_guide read count : 988
novel-nve-miR-89_star read count : 82
remaining reads : 6

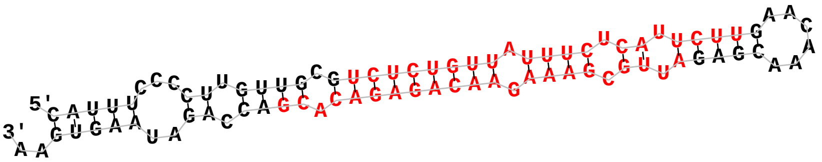

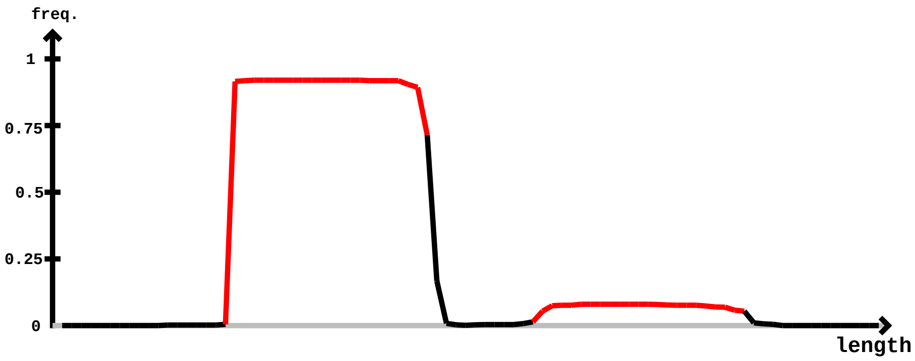

novel-nve-miR-89_guide

novel-nve-miR-89_star

```
5'- cauuuccccuuguugcgucucuguuauuucucauucuugaacaaacgagauugcgaaagaacagagacacgaccagauaagugaa   -3'    exp
    (((((...((.((((.(((((((((.((((.((.(((((......))))).)).)))).))))))))).)))).))..)))))..    reads      mm         sample
    ..........uguugcgucucuguuauuucu......................................................    2          0          seq
    ................gucucuguuauuucucauucuu...............................................    1          0          seq
    ................gucucuguuauuucucauucuG...............................................    1          1          seq
    ................Cucucuguuauuucucauucuu...............................................    1          1          seq
    .................ucucuguuauuucucauC..................................................    4          1          seq
    .................ucucuguuauuuGucauu..................................................    1          1          seq
    .................ucucuguuauuucucauu..................................................    10         0          seq
    .................ucucuguuauuucucauuU.................................................    2          1          seq
    .................ucucuguuauuucucauuc.................................................    10         0          seq
    .................ucucugCuauuucucauucu................................................    1          1          seq
    .................ucucuguuauuucucGuucu................................................    1          1          seq
    .................ucucuguuauuucucauucA................................................    6          1          seq
    .................ucucuguuauuucCcauucu................................................    2          1          seq
    .................ucucuguuauuucucauucC................................................    41         1          seq
    .................ucucuguuauuuAucauucu................................................    1          1          seq
    .................ucucuguAauuucucauucu................................................    1          1          seq
    .................ucucuguCauuucucauucu................................................    1          1          seq
    .................ucucCguuauuucucauucu................................................    1          1          seq
    .................ucucuguuauuucucauucu................................................    131        0          seq
    .................Ccucuguuauuucucauucu................................................    1          1          seq
    .................ucucuguuauuucuAauucu................................................    1          1          seq
    .................ucucuguuauCucucauucu................................................    2          1          seq
    .................ucAcuguuauuucucauucu................................................    1          1          seq
    .................ucucuguuauuucucauucG................................................    1          1          seq
    .................ucucuguuauuucucauucuC...............................................    83         1          seq
    .................ucucuguuaGuucucauucuu...............................................    1          1          seq
    .................ucucuguuaCuucucauucuu...............................................    3          1          seq
    .................ucuAuguuauuucucauucuu...............................................    1          1          seq
    .................ucucuguuGuuucucauucuu...............................................    2          1          seq
    .................Ccucuguuauuucucauucuu...............................................    3          1          seq
    .................ucucuguuCuuucucauucuu...............................................    1          1          seq
    .................ucCcuguuauuucucauucuu...............................................    1          1          seq
    .................ucucuCuuauuucucauucuu...............................................    1          1          seq
    .................uUucuguuauuucucauucuu...............................................    2          1          seq
```

novel-nve-miR-89_guide

novel-nve-miR-89_star

cauuuccccuuguugcgucucuguuauuucucauucuugaacaaacgagauugcgaaagaacagagacacgaccagauaagugaa

.................ucAcuguuauuucucauucuu...	1	1	seq
.................ucucuguuauuCcucauucuu...	2	1	seq
.................ucucuguuauCucucauucuu...	1	1	seq
.................ucucCguuauuucucauucuu...	1	1	seq
.................ucucugCuauuucucauucuu...	1	1	seq
.................ucucuguuauuucucGuucuu...	3	1	seq
.................ucucuguuauuucucauucuG...	3	1	seq
.................ucucuUuuauuucucauucuu...	1	1	seq
.................ucucugAuauuucucauucuu...	1	1	seq
.................ucucuguuauuucuUauucuu...	1	1	seq
.................ucucGguuauuucucauucuu...	1	1	seq
.................Acucuguuauuucucauucuu...	3	1	seq
.................uAucuguuauuucucauucuu...	1	1	seq
.................ucucuguuauuAcucauucuu...	2	1	seq
.................ucucuguuauuucAcauucuu...	1	1	seq
.................ucucuguuauuucucauucuu...	450	0	seq
.................ucucuguuauuucucauuAuu...	2	1	seq
.................ucucuguuauuucucauucCu...	1	1	seq
.................ucucuguuauuucucauucuA...	8	1	seq
.................ucucuguuauuucucaCucuu...	1	1	seq
.................ucucuguuauuucucauucuug..	129	0	seq
.................ucucuguuauuucAcauucuug..	1	1	seq
.................ucucuguuauuCcucauucuug..	1	1	seq
.................ucucuguuauuucucauucuuA..	9	1	seq
.................Acucuguuauuucucauucuug..	1	1	seq
.................ucucuguuauuucucauucuuC..	3	1	seq
.................ucucuguuauuucucauCcuug..	1	1	seq
.................ucucuguuauuucucauucuuU..	21	1	seq
.................ucucuguuauuuUucauucuug..	1	1	seq
.................Ccucuguuauuucucauucuug..	2	1	seq
.................ucucugAuauuucucauucuug..	1	1	seq
.................ucucCguuauuucucauucuug..	1	1	seq
.................ucucuguuauuucucauucuuUa...	2	1	seq
.................ucucuguuauuucucauucuugC...	1	1	seq
.................ucucuguuauuucucauucuugU...	2	1	seq
.................ucucuguuauuucucauucuugaU..	1	1	seq
.................ucucuguuauuucucauucuugaC..	1	1	seq
..................cucuguuauuucucauucuu...	3	0	seq
...................ucuguuauuucucauucuuUa...	1	1	seq
...................ucugCuauuucucauucuugaa..	1	1	seq
....caaacgagaCugcgaaagaa........................	1	1	seq
....aaacgagaCugcgaaagaac.......................	2	1	seq
....aacgagaCugcgaaagaaca......................	1	1	seq
....agauugcgaaagaacagag...................	2	0	seq
....agauugcgaaagaacagaga..................	1	0	seq
....Ggauugcgaaagaacagagaca................	1	1	seq
....gauugcgaaagaacagag...................	1	0	seq
....gauugcgaaagaacagagacacA..............	3	1	seq
....gauugcgaaagaacagagacacU..............	3	1	seq
....auugcgaaagaacagaga..................	3	0	seq
....auugcgaaagaacagagac.................	1	0	seq
....auugcgaaagaacagagaca................	7	0	seq
....auugcgaaagaacagagacac...............	1	0	seq
....auugcgaaagaacagagCcac...............	1	1	seq
....auugcgaaagaacagagacaU...............	1	1	seq
....auugcgaaagaacagagacacC..............	5	1	seq
....auugcgaaagaacagagacacA..............	5	1	seq
....auugcgaaagaacagagacacU..............	19	1	seq
....uugcgaaagaacagagaca................	4	0	seq
....uugcgaaagaacagagGcac...............	1	1	seq
....uugcgaaagaacagagacacA..............	1	1	seq
....uugcgaaagaacagagacacC..............	1	1	seq
....uugcgaaagaacagagacacU..............	8	1	seq
....uugcgaaagaacagagacacga.............	1	0	seq
....uugcgaaagaGcagagacacga.............	1	1	seq
....uugcgaaagaacagagacacgacc...........	1	0	seq
....uugcgaaagaacagagacacgacG...........	1	1	seq
....uugcgaaagaacagagacacgacU...........	3	1	seq
....ugcgaaagaacagagacacC..............	1	1	seq

```
                 novel-nve-miR-89_guide
                                                 novel-nve-miR-89_star
cauuuccccuuguugcgucucuguuauuucucauucuugaacaaacgagauugcgaaagaacagagacacgaccagauaagugaa

...................................................ugcgaaagaacagagacacU..............    1          1          seq
.....................................................cgaaagaacagagacGcga.............    2          1          seq
.....................................................cgaaagaacagagacGcgac............    2          1          seq
```

miRBase precursor : novel-nve-miR-91
Total read count : 174
novel-nve-miR-91_guide read count : 159
novel-nve-miR-91_star read count : 15
remaining reads : 0

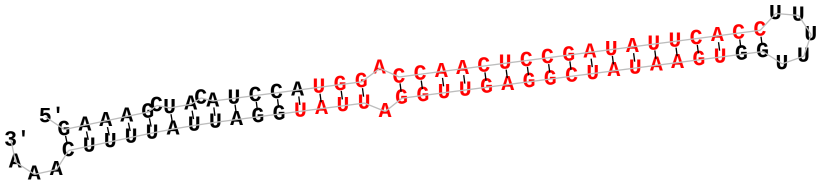

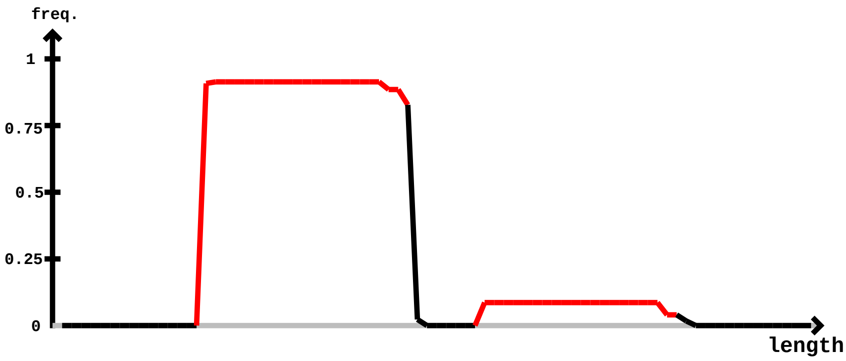


```
                        novel-nve-miR-91_guide
                                            novel-nve-miR-91_star
5'- gaaagcuacauccauggaccaacuccgauauucaccuuuuuggugaauaucggaguuggauuauggauuauuuucaaa   -3'    exp
    (((((.((.((((((((.((((((((((((((((((.....)))))))))))))))))).)))))))))))))))...     reads      mm         sample
    ..............Aggaccaacuccgauauuc.............................................     1          1          seq
    ..............uggaccaacuccgauauuc.............................................     4          0          seq
    ..............uggaccaacuccgauauucaU...........................................     1          1          seq
    ..............uggaccaacuccgauauucac...........................................     9          0          seq
    ..............uggaccaacuccgauauucacA..........................................     13         1          seq
    ..............uggaccaacuccgauauucacU..........................................     37         1          seq
    ..............uggacAaacuccgauauucacc..........................................     1          1          seq
    ..............uggaccaacuccgauauucacc..........................................     86         0          seq
    ..............uggaccaacuccgauauCcacc..........................................     2          1          seq
    ..............uggaccaacuccgauauucaccu.........................................     2          0          seq
    ..............uggaccaacuccgauauucaccC.........................................     1          1          seq
    ..............uggaccaacuccgauauucacAu.........................................     1          1          seq
    ...............Ugaccaacuccgauauucacc..........................................     1          1          seq
    ...........................................ugaauaucggaguuggauu................     8          0          seq
    ...........................................ugaauaucggaguuggauuaA..............     3          1          seq
    ...........................................ugaauaucggaguuggauuaG..............     1          1          seq
    ...........................................ugaauaucggaguuggauuaug.............     3          0          seq
```

miRBase precursor : novel-nve-miR-92
Total read count : 241
novel-nve-miR-92_guide read count : 233
novel-nve-miR-92_star read count : 6
remaining reads : 2

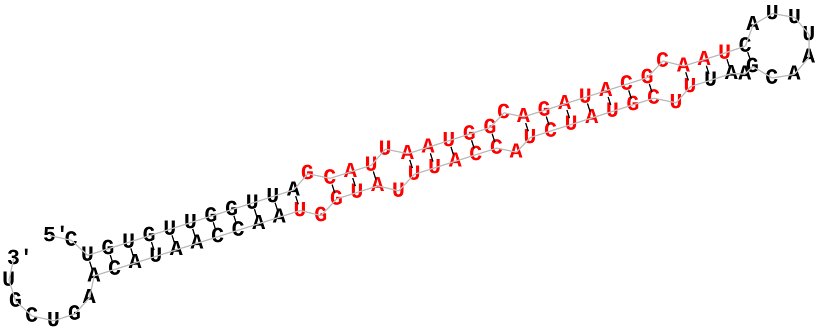

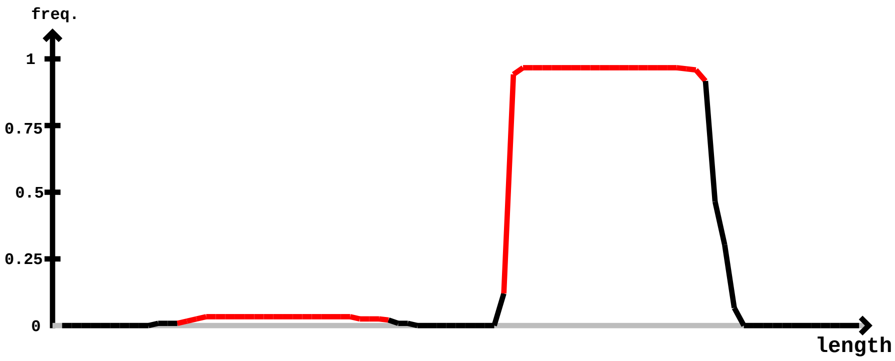

novel-nve-miR-92_star

novel-nve-miR-92_guide

5'- cuguguugguuagcauuaauggcagauacgcaaucauuuaacgaauuucguaucuaccauuuaugguaaccaauacaagucgu -3'	exp		
.((((((((((.(((.(((((.(((((((.((((.......).))).))))))).))))).))).))))))))))......	reads	mm	sample
.........uuagcauuaauggcagauacg...	2	0	seq
............gcauuaauggcagauacgcaau...	1	0	seq
............gcauuaauggcagauacgcaaA...	1	1	seq
.............cauuaauggcagauacgcaa..	1	0	seq
.............cauuaauggcagauacgcaaC...	1	1	seq
..............auuaauggcagauacgcaaucG...	1	1	seq
..............auuaauggcagauacgcaauca...	1	0	seq
....uuucguaucuaccauuuau...................	1	0	seq
....uuucguaucuaccauuuaug..................	1	0	seq
....uuucguaucuaccauuuaugg.................	2	0	seq
....uuucguaucuaccauuuauggu................	12	0	seq
....uuucguaucuGccauuuauggu................	1	1	seq
....uuucguaucuaccauuuauggC................	7	1	seq
....uuucguaucuaccauuuauggG................	1	1	seq
....uuucguaucuaccauuuaugguC...............	1	1	seq
....uuucguaucuaccauuuaugguU...............	2	1	seq
....uuucguaucuaccauuuaugguUa..............	1	1	seq
....uucguaucuaccauuuaugg.................	7	0	seq
....uucguaucuaccauuuaugA.................	1	1	seq
....uucguaucuGccauuuauggu................	8	1	seq
....uucguaucAaccauuuauggu................	1	1	seq
....uucguaucuaccauuuauggu................	65	0	seq
....uucguaucuaccauuuauggA................	1	1	seq
....uucguaucuaccauuuauggC................	11	1	seq
....uucguGucuaccauuuauggu................	1	1	seq
....uucguaucuGccauuuauggua...............	2	1	seq
....uucguaucuaccauuuaugguU...............	19	1	seq
....Aucguaucuaccauuuauggua...............	1	1	seq
....uucguaucuaccauuuauggua...............	10	0	seq
....uucguaucuaccauuuaugguC...............	4	1	seq
....Aucguaucuaccauuuaugguaa..............	1	1	seq
....uucguaucuaccauuuaugguaa..............	27	0	seq
....uucguaucuaccauuuaugguaC..............	5	1	seq
....uucguaucuGccauuuaugguaa..............	4	1	seq

```
            novel-nve-miR-92_star
                                              novel-nve-miR-92_guide
cuguguugguuagcauuaauggcagauacgcaaucauuuaacgaauuucguaucuaccauuuaugguaaccaauacaagucgu

..............................................uucguaucuaccauuuaugguaU..............    14    1    seq
..............................................uucguaucuaccauuuaugguaaA.............    2     1    seq
..............................................uucguaucuaccauuuaugguaac.............    2     0    seq
..............................................uucguaucuaccauuuaugguaaU.............    12    1    seq
...............................................ucguaucuaccauuuauggu................    1     0    seq
...............................................ucguaucuaccauuuaugguaa..............    5     0    seq
```

miRBase precursor : novel-nve-miR-94
Total read count : 79
novel-nve-miR-94_guide read count : 79
novel-nve-miR-94_star read count : 0
remaining reads : 0

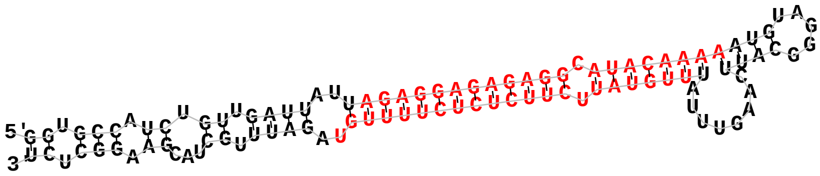

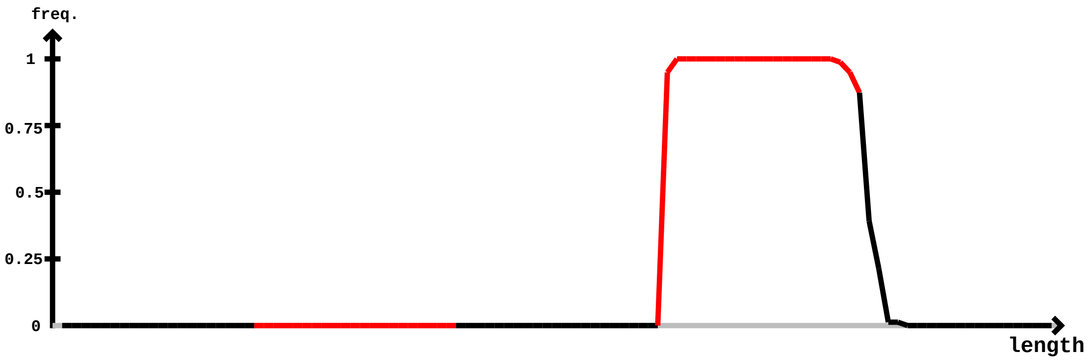

novel-nve-miR-94_star

novel-nve-miR-94_guide

```
5'- ggugccaucuguugauuauuagaggagagaggcauacaaaaauguagggcauucaaguuuauuuguauucuucucucuuuuguagauuugcuacgaaggcucu   -3'    exp
    ((.(((.((.((.((((..((((((((((((.((((((((((.....)))).......))))))).)))))))))))))..)))).))...)).))).))    reads     mm      sample
    ..............................................................uuguauucuucucucuuC.......................    1         1       seq
    ..............................................................uuguauucuucucucuuuu......................    3         0       seq
    ..............................................................uuguauucuucucucuuuug.....................    5         0       seq
    ..............................................................uuguauucuucucucuuuuA.....................    1         1       seq
    ..............................................................uuguauucuucucucuuuugC....................    9         1       seq
    ..............................................................uuguauucuucucucuuuugG....................    1         1       seq
    ..............................................................uAguauucuucucucuuuugu....................    1         1       seq
    ..............................................................uuguauucuucucucuuuugu....................    25        0       seq
    ..............................................................uuguauucuucucAcuuuugu....................    1         1       seq
    ..............................................................uuguauucuucucucuuuugA....................    1         1       seq
    ..............................................................uuguauucuucucucuuuuguC...................    1         1       seq
    ..............................................................uuguauucuucucucuuuuCgua..................    1         1       seq
    ..............................................................uuguauucuucucucuuuugua..................    4         0       seq
    ..............................................................uuguauucuucucucuuuuguU...................    6         1       seq
    ..............................................................uuguauucuucucucuuuuguaA.................    2         1       seq
    ..............................................................uuguauucuucucucuuuuguaC.................    1         1       seq
    ..............................................................uuguauucuucucucuuuuguag.................    2         0       seq
    ..............................................................uuguauucuucucucuuuuguaU.................    9         1       seq
    ..............................................................uuguauucuucucucuuuuguaUau...............    1         1       seq
    ...............................................................uguauucuucucucuuuuguU...................    1         1       seq
    ...............................................................uguauucuucucucuuuugua...................    1         0       seq
    ...............................................................uguauucuucucucuuuuguaU..................    2         1       seq
```

miRBase precursor : novel-nve-miR-96
Total read count : 2519
novel-nve-miR-96_guide read count : 2517
novel-nve-miR-96_star read count : 2
remaining reads : 0

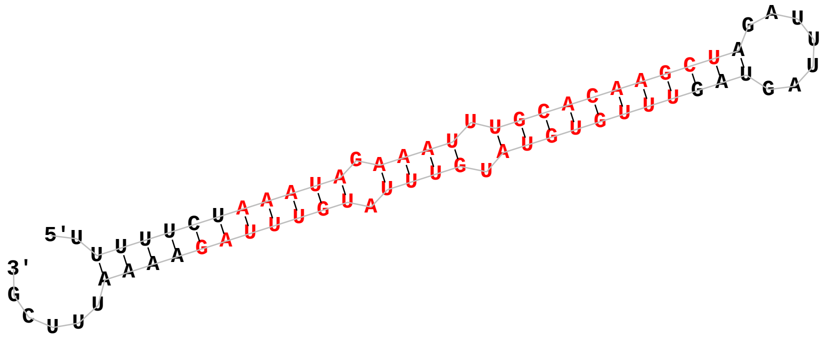

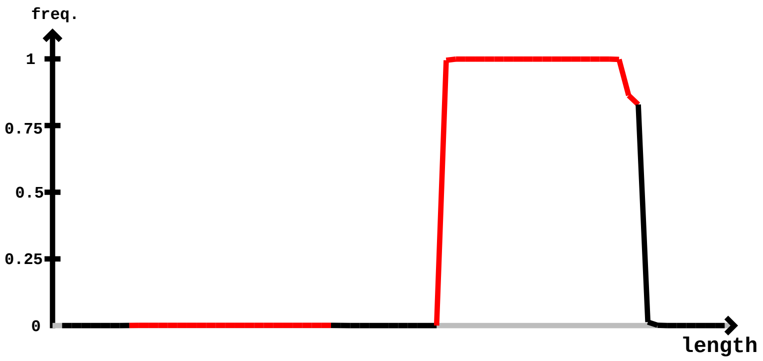


```
                                          novel-nve-miR-96_guide
       novel-nve-miR-96_star
5'- uuuuucuaaauagaaauuugcacaagcuagauuuaguaguuuguguauguuuauguuuagaaaauuucg   -3'    exp
    .((((((((((.((((.((((((((((((.......)))))))))))).)))).))))))))))).....     reads      mm         sample
    ......uaaauagaaauuugcacaagcu.........................................     1          0          seq
    .......aaauagaaauuugcacaagcuU........................................     1          1          seq
    .......................................uuuguguauguuuCuguu............     3          1          seq
    .......................................uuuguguauguuuauguuu...........     3          0          seq
    .......................................uuuguguauguuuauguuC...........     1          1          seq
    .......................................uuuguguauguuuCuguuu...........     336        1          seq
    .......................................uuuguguauguuuCuguuua..........     86         1          seq
    .......................................uuuguguauguuuUuguuuag.........     1          1          seq
    .......................................uuuguguauAuuuauguuuag.........     1          1          seq
    .......................................uuuguguauguuuCuguuuag.........     2025       1          seq
    .......................................uuuguguauguuuauguuuaA.........     1          1          seq
    .......................................uuuguguauguuuauguuuag.........     16         0          seq
    .......................................uuuguguauguuuauguuuaga........     1          0          seq
    .......................................uuuguguauguuuCuguuuaga........     29         1          seq
    .......................................uuuguguauguuuCuguuuagaa.......     2          1          seq
    .......................................uuuguguauguuuauguuuagUa.......     1          1          seq
    ........................................uuguguauguuuCuguuuag.........     11         1          seq
```

miRBase precursor : novel-nve-miR-102-1
Total read count : 229
novel-nve-miR-102-1_guide read count : 207
novel-nve-miR-102-1_star read count : 21
remaining reads : 1

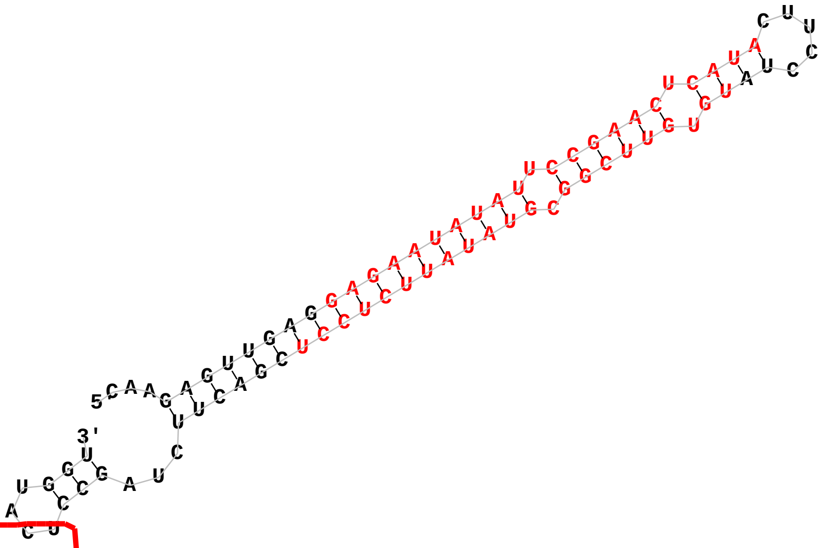

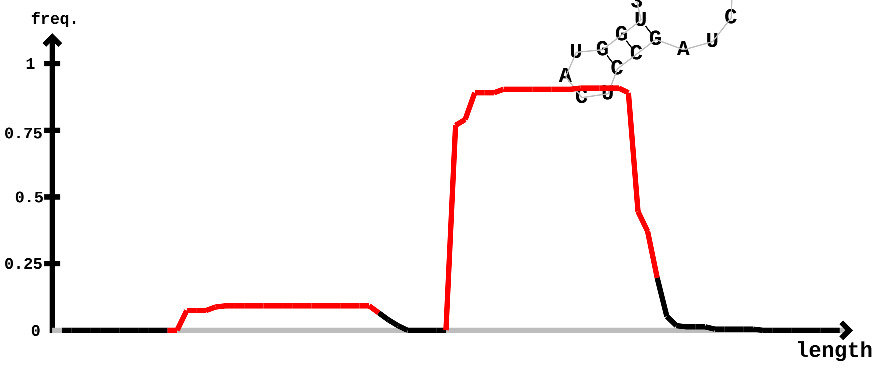

novel-nve-miR-102-1_guide

novel-nve-miR-102-1_star

5'- caagaguugaggagaauauauuccgaacucauacuuccuauguguucggcguauauucuccucgacuucuagccucauggu -3' exp

...((((((((((((((((((.((((((.((((.....)))).)))))).))))))))))))))))))...(((....)))	reads	mm	sample
............agaauauauuccgaacucaC...	1	1	seq
............agaauauauuccgaacucau...	4	0	seq
............agaGuauauuccgaacucau...	1	1	seq
............agaauauauuccgaacucaua..	4	0	seq
............agaauauauuccgaacucauU..	2	1	seq
............agaauauauuccgaacucauaU...	4	1	seq
............Ggaauauauuccgaacucauac...	1	1	seq
...............auauauuccgaacucauacu..	2	0	seq
...............auauauuccgaacucauacC..	1	1	seq
................uauauuccgaacucauaUu..	1	1	seq
....uguguucggcguauauuc.......................	3	0	seq
....uguguucggcguauauuU.......................	1	1	seq
....uguguucggcguauauucC......................	15	1	seq
....uguguucggcguauauucA......................	1	1	seq
....uguguucggcguauauucG......................	13	1	seq
....ugCguucggcguauauucu......................	2	1	seq
....uguguucggcguauauucu......................	65	0	seq
....Gguguucggcguauauucu......................	1	1	seq
....uguguucggcguauauuUu......................	2	1	seq
....uguguucggcguauUuucu......................	1	1	seq
....uguguucggcguauauucAc.....................	1	1	seq
....uguguucggcguauauucuc.....................	8	0	seq
....uguguucggcguauauucuU.....................	2	1	seq
....uguguucggcguauauucucG....................	2	1	seq
....uguguucggcguauauucucc....................	17	0	seq
....uguguucggcguauauucucA....................	5	1	seq
....uguguucggcguauauucucU....................	9	1	seq
....uguguucggcguUuauucucc....................	1	1	seq
....uguguucggcguauauucuAc....................	2	1	seq
....uguguucggcguauauucuccG...................	3	1	seq
....ugugCucggcguauauucuccu...................	1	1	seq
....uguguucggcguauauCcuccu...................	1	1	seq
....uguguucggcguauauucuccC...................	2	1	seq
....uguguucggcguauauucuccA...................	2	1	seq

novel-nve-miR-102-1_guide

novel-nve-miR-102-1_star

caagaguugaggagaauauauuccgaacucauacuuccuauguguucggcguauauucuccucgacuucuagccucauggu

....uguguucggcguauauucuccu...................	15	0	seq
....uguguucggcguauauucuccuU..................	1	1	seq
....guguucggcguauauucu......................	2	0	seq
....guguucggcguauauucucc....................	1	0	seq
....guguucggcAuauauucucc....................	1	1	seq
....guguucggcguauauucuccuU..................	1	1	seq
....uguucggcguauauucuc.....................	3	0	seq
....uguucggcguauauucuU.....................	2	1	seq
....uguucggcguauauucuA.....................	1	1	seq
....uguucggcguauauucucU....................	2	1	seq
....uguucggcguauauucuccA...................	3	1	seq
....uguucggcguauauucuccu...................	2	0	seq
....uguucggcguauauucuccC...................	2	1	seq
....uguucggcguauauucuccG...................	1	1	seq
....uguucggcguauauucuAcu...................	1	1	seq
....uguucggcguauauucuccuc..................	1	0	seq
....uguucggcguauauucuccuU..................	5	1	seq
....ucggcAuauauucuccucg.................	1	1	seq
....ucggcguauauucuccucgacu..............	2	0	seq
....uauucuccucgacuucuCg.........	1	1	seq

miRBase precursor : novel-nve-miR-103-1
Total read count : 2972
novel-nve-miR-103-1_guide read count : 2932
novel-nve-miR-103-1_star read count : 19
remaining reads : 21

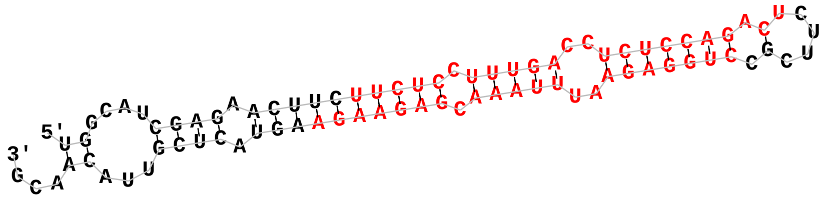

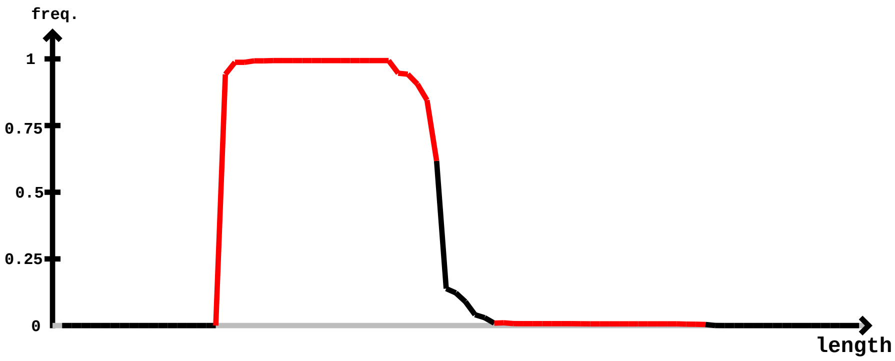


```
                             novel-nve-miR-103-1_guide
                                                           novel-nve-miR-103-1_star
5'- uggcaucgagaacuucuucuccuuugaccucuccagacucuucgccuggagaauuuaaacgagaagaaguacucguuacaacg   -3'    exp
    ((....((((.((((((((((.(((((..(((((((.(.....).)))))))..))))).)))))))))).))))...))...   reads      mm         sample
    ................uuUuccuuugaccucucc.................................................   1          1          seq
    ................uuGuccuuugaccucucc.................................................   1          1          seq
    ................uucuccuuugaccucucA.................................................   13         1          seq
    ................uCcuccuuugaccucucc.................................................   1          1          seq
    ................uucuccuuugaccucCcc.................................................   1          1          seq
    ................uucuccuuugacUucucc.................................................   28         1          seq
    ................uucuccuAugaccucucc.................................................   2          1          seq
    ................uucuccuuugaccucucU.................................................   23         1          seq
    ................uucuccuCugaccucucc.................................................   1          1          seq
    ................Cucuccuuugaccucucc.................................................   1          1          seq
    ................uucuccuuugaUcucucc.................................................   6          1          seq
    ................uucuccuuugaccucucc.................................................   58         0          seq
    ................uucuccuuugCccucucc.................................................   2          1          seq
    ................uucuccuuugGccucucc.................................................   2          1          seq
    ................uucuccuuugaccucuUc.................................................   1          1          seq
    ................uucuccuuugaccuUucca................................................   1          1          seq
    ................uucuccuuugaccucuccC................................................   1          1          seq
    ................uucuccuuugaccucucca................................................   9          0          seq
    ................uucuccuuugaccucucUag...............................................   3          1          seq
    ................uucucUuuugaccucuccag...............................................   1          1          seq
    ................uucuccuuugaUcucuccag...............................................   9          1          seq
    ................uucuccuuugacUucuccag...............................................   11         1          seq
    ................uucuUcuuugaccucuccag...............................................   1          1          seq
    ................uucuccuuAgaccucuccag...............................................   1          1          seq
    ................uucuccuuuCaccucuccag...............................................   1          1          seq
    ................uuUuccuuugaccucuccag...............................................   1          1          seq
    ................uucuccuuugaccucuccaU...............................................   4          1          seq
    ................uucuccuuugacAucuccag...............................................   1          1          seq
    ................uucuccuuugaccucuUcag...............................................   2          1          seq
    ................uucuccuuugaccucuccaA...............................................   9          1          seq
    ................uucuccuuugaccucuccaC...............................................   1          1          seq
    ................uAcuccuuugaccucuccag...............................................   1          1          seq
    ................uucuccuuugaccucuccag...............................................   63         0          seq
    ................uucuccuuugaccucuccaga..............................................   92         0          seq
```

```
                novel-nve-miR-103-1_guide
                                             novel-nve-miR-103-1_star
uggcaucgagaacuucuucuccuuugaccucuccagacucuucgccuggagaauuuaaacgagaagaaguacucguuacaacg

................uucuccuuugaccucCccaga..............................................    1          1          seq
................uucuccuuugaccCcuccaga..............................................    1          1          seq
................uucuUcuuugaccucuccaga..............................................    3          1          seq
................uucuccuuugaccucuUcaga..............................................    2          1          seq
................uucucUuuugaccucuccaga..............................................    1          1          seq
................uucuccuuugacAucuccaga..............................................    1          1          seq
................uucuccGuugaccucuccaga..............................................    1          1          seq
................uucuccuuugaccucuccagU..............................................    2          1          seq
................uucuccuuugacUucuccaga..............................................    60         1          seq
................uucuccuAugaccucuccaga..............................................    1          1          seq
................uucuccuuugaUcucuccaga..............................................    5          1          seq
................uucuccCuugaccucuccaga..............................................    1          1          seq
................uucuccuuugGccucuccaga..............................................    2          1          seq
................uucuccuuugaccucuccagG..............................................    5          1          seq
................uucuccuuugaccucuGcagac.............................................    1          1          seq
................uucuccuuugaccucucUagac.............................................    3          1          seq
................uucuccuuCgaccucuccagac.............................................    1          1          seq
................uucuccuAugaccucuccagac.............................................    1          1          seq
................uucuccuuugaccucuccagac.............................................    363        0          seq
................uucucUuuugaccucuccagac.............................................    1          1          seq
................uucCccuuugaccucuccagac.............................................    2          1          seq
................Cucuccuuugaccucuccagac.............................................    4          1          seq
................uucuccuuugaccucuccagaA.............................................    11         1          seq
................uucuccuuuCaccucuccagac.............................................    1          1          seq
................uCcuccuuugaccucuccagac.............................................    2          1          seq
................uucuccuuugaccucuccagaU.............................................    66         1          seq
................uucuccuuugaccucuccGgac.............................................    1          1          seq
................uuUuccuuugaccucuccagac.............................................    4          1          seq
................uucuccuuugacUucuccagac.............................................    111        1          seq
................uucuAcuuugaccucuccagac.............................................    1          1          seq
................uucuccuuugaccCcuccagac.............................................    3          1          seq
................uucuccuuugaccucuccagGc.............................................    5          1          seq
................uucuccuuugaccucucAagac.............................................    2          1          seq
................uucuccuuugaUcucuccagac.............................................    49         1          seq
................uucuccuCugaccucuccagac.............................................    1          1          seq
................uucuccuuugCccucuccagac.............................................    1          1          seq
................uuAuccuuugaccucuccagac.............................................    1          1          seq
................uucuccuuugaAcucuccagac.............................................    1          1          seq
................uAcuccuuugaccucuccagac.............................................    1          1          seq
................uucuUcuuugaccucuccagac.............................................    9          1          seq
................uucuccuuugaccucuUcagac.............................................    9          1          seq
................uucucAuuugaccucuccagac.............................................    1          1          seq
................Aucuccuuugaccucuccagac.............................................    3          1          seq
................uucuccuuugaccucCccagac.............................................    4          1          seq
................Gucuccuuugaccucuccagac.............................................    1          1          seq
................uucuccCuugaccucuccagac.............................................    2          1          seq
................uucuccuuugaccuUuccagac.............................................    1          1          seq
................uGcuccuuugaccucuccagacu............................................    1          1          seq
................Aucuccuuugaccucuccagacu............................................    3          1          seq
................uucuccuuugaccucuccagacu............................................    754        0          seq
................uucuccuuugaccucuccagacG............................................    22         1          seq
................uucuccuuugaccucucGagacu............................................    4          1          seq
................uAcuccuuugaccucuccagacu............................................    4          1          seq
................uucuccuuugaUcucuccagacu............................................    43         1          seq
................uucuccuuugacUucuccagacu............................................    206        1          seq
................uucuccuuugaccucuccUgacu............................................    1          1          seq
................uucuccuuugaccucuccGgacu............................................    7          1          seq
................Gucuccuuugaccucuccagacu............................................    2          1          seq
................uucuccAuugaccucuccagacu............................................    2          1          seq
................uucuccuuugaccucuccagacC............................................    119        1          seq
................uucuccuuugaccucuccagaGu............................................    1          1          seq
................uucuccuuCgaccucuccagacu............................................    6          1          seq
................uucucUuuugaccucuccagacu............................................    3          1          seq
................uucuccuuugaccucuccagaUu............................................    2          1          seq
................uucuccuuugaAcucuccagacu............................................    1          1          seq
................uucuccuuugGccucuccagacu............................................    1          1          seq
................uucuccuuugaccCcuccagacu............................................    7          1          seq
................uucuccuuugaGcucuccagacu............................................    1          1          seq
................uucuccuAugaccucuccagacu............................................    2          1          seq
```

```
                novel-nve-miR-103-1_guide
                                             novel-nve-miR-103-1_star
uggcaucgagaacuucuucuccuuugaccucuccagacucuucgccuggagaauuuaaacgagaagaaguacucguuacaacg

................uucCccuuugaccucuccagacu............................................   1    1    seq
................uuUuccuuugaccucuccagacu............................................   5    1    seq
................uucuccuuugaccucuUcagacu............................................   20   1    seq
................uucuccuuugacAucuccagacu............................................   1    1    seq
................uucuccuCugaccucuccagacu............................................   2    1    seq
................uucuccuuugaccucuGcagacu............................................   1    1    seq
................Cucuccuuugaccucuccagacu............................................   4    1    seq
................uucuccuuugaccucuccCgacu............................................   1    1    seq
................uCcuccuuugaccucuccagacu............................................   4    1    seq
................uucuccGuugaccucuccagacu............................................   1    1    seq
................uucuccuuugaccucucAagacu............................................   2    1    seq
................uucuccuuugaccucCccagacu............................................   3    1    seq
................uucuccuuugaccucuccagaAu............................................   1    1    seq
................uucuUcuuugaccucuccagacu............................................   7    1    seq
................uucuccuuugaccucuccagacA............................................   93   1    seq
................uucuccuuugaccucuccagGcu............................................   1    1    seq
................uucuccuuAgaccucuccagacu............................................   1    1    seq
................uucuccuuugaccuGuccagacu............................................   1    1    seq
................uucuccuuugaccucuccagCcu............................................   1    1    seq
................uucuccuuugaccucucUagacu............................................   20   1    seq
................uucuccCuugaccucuccagacu............................................   4    1    seq
................uucucAuuugaccucuccagacu............................................   4    1    seq
................uucuccuuugaccuUuccagacu............................................   7    1    seq
................uucuccuuugacUucuccagacuc...........................................   2    1    seq
................uuGuccuuugaccucuccagacuc...........................................   1    1    seq
................uucuccuuugaccucucGagacuc...........................................   1    1    seq
................uucuccuuugaccucuccagacuU...........................................   15   1    seq
................uucuccuuugaccucuccagacuA...........................................   10   1    seq
................uucuccuuugaccucuccagacuG...........................................   1    1    seq
................uucuccuuugaccucuccagacuc...........................................   9    0    seq
................uucuccuuugaccucuccagacuAu..........................................   37   1    seq
................uucuccuuugaAcucuccagacucu..........................................   1    1    seq
................uucuccuuugaccucuccagacucC..........................................   2    1    seq
................uucuccuuugaccucuccagacucu..........................................   23   0    seq
................uucuccuuugaccucuccagacuUu..........................................   2    1    seq
................uucuccuuugaUcucuccagacucu..........................................   2    1    seq
................uucuccuuugaccucuUcagacucu..........................................   1    1    seq
................uucuccuuugacUucuccagacucu..........................................   3    1    seq
................uCcuccuuugaccucuccagacucuu.........................................   1    1    seq
................uucuAcuuugaccucuccagacucuu.........................................   1    1    seq
................uucuccuuugaccucuccagacucuC.........................................   14   1    seq
................uucuccuuugacUucuccagacucuu.........................................   9    1    seq
................uucuccuuugaccucuccagacuAuu.........................................   1    1    seq
................uucuccuuugaccucuccagacucuu.........................................   80   0    seq
................uucuccuCugaccucuccagacucuu.........................................   1    1    seq
................uucuccuuugaccucuccagacuUuu.........................................   6    1    seq
................uucuccuuugaccucuccagacucGu.........................................   1    1    seq
................uucuccuuugaccucuGcagacucuu.........................................   1    1    seq
................Cucuccuuugaccucuccagacucuu.........................................   1    1    seq
................uucuccuuugaccucuccagacucuA.........................................   1    1    seq
................Aucuccuuugaccucuccagacucuu.........................................   2    1    seq
................uuUuccuuugaccucuccagacucuu.........................................   1    1    seq
................uucuccuuugaccucuccGgacucuu.........................................   1    1    seq
................uucuccuuugaUcucuccagacucuu.........................................   5    1    seq
................uucuccuuugacUucuccagacucuuc........................................   4    1    seq
................uucuccuuugaccucuccagacucuuU........................................   7    1    seq
................uucuccuuugaccucuccagacucuuA........................................   2    1    seq
................uucuccuuugaccucuccagacuUuuc........................................   1    1    seq
................uucuccuuugaccucuccagacucuuc........................................   8    0    seq
................uucuccuuugaccucuccagacucuucU.......................................   3    1    seq
................uucuccuuugacUucuccagacucuucg.......................................   5    1    seq
................uucuccuuugaUcucuccagacucuucg.......................................   2    1    seq
................uucuccuuugaccucuccagacucuuAg.......................................   1    1    seq
................uucuccuAugaccucuccagacucuucg.......................................   1    1    seq
................uucuccuuugaccucuccagacucuuUg.......................................   1    1    seq
................uucuccuuugaccucucAagacucuucg.......................................   1    1    seq
................uuGuccuuugaccucuccagacucuucg.......................................   1    1    seq
................uucuccuuugacAucuccagacucuucg.......................................   1    1    seq
................uucuccuuugaccucuccagacucuucA.......................................   8    1    seq
```

```
                novel-nve-miR-103-1_guide
                                             novel-nve-miR-103-1_star
uggcaucgagaacuucuucuccuuugaccucuccagacucuucgccuggagaauuuaaacgagaagaaguacucguuacaacg

................uucuccuuugGccucuccagacucuucg.......................................   1    1    seq
................uucuccuuugaccucuccagacucuucg.......................................   19   0    seq
................uucuccuuugacUucuccagacucuucgc......................................   1    1    seq
................uucuccuuugaccucuccagacucuucgc......................................   2    0    seq
................uucuccuuugaccucuccagacucuucgA......................................   1    1    seq
................uucuccuuugaccucuccagacucuucgU......................................   1    1    seq
................uucuccuuugaccucuccagacucuucgcU.....................................   8    1    seq
................uucuccuuugaccucuccagacucuucgcA.....................................   1    1    seq
................uucuccuuugaccucuccagacucuucgcUu....................................   1    1    seq
.................ucuccuuugaUcucuccag...............................................   1    1    seq
.................ucuccuuugaccucuccagac.............................................   4    0    seq
.................uUuccuuugaccucuccagac.............................................   3    1    seq
.................ucuccuuugacUucuccagacu............................................   7    1    seq
.................Acuccuuugaccucuccagacu............................................   1    1    seq
.................ucuccuuugaccucuUcagacu............................................   1    1    seq
.................uUuccuuugaccucuccagacu............................................   3    1    seq
.................ucuccuuugaccucucUagacu............................................   2    1    seq
.................ucuccuuugaccucuccagacA............................................   2    1    seq
.................ucuccuuugaccGcuccagacu............................................   1    1    seq
.................ucuccuuugaccucuccagacu............................................   15   0    seq
.................ucuccuuugaccucuccagacC............................................   4    1    seq
.................ucucUuuugaccucuccagacu............................................   5    1    seq
.................ucuccuuugaccucuccUgacu............................................   1    1    seq
.................ucuccuuugaccucuccagacuU...........................................   2    1    seq
.................ucuccuuugaccucuccagacuG...........................................   1    1    seq
.................ucuccuuugaccucuccagacuA...........................................   3    1    seq
.................ucuccuuugaccucuccagacuc...........................................   3    0    seq
.................ucuccuuugaccucuccagacucA..........................................   1    1    seq
.................ucuccuuugacUucuccagacucu..........................................   1    1    seq
.................ucuccuuugaccuUuccagacucu..........................................   2    1    seq
.................ucCccuuugaccucuccagacucu..........................................   1    1    seq
.................ucuccuuugaccucuccagacucC..........................................   5    1    seq
.................uUuccuuugaccucuccagacucu..........................................   1    1    seq
.................ucuccuuugaccucuccagacucu..........................................   10   0    seq
.................ucuccuuugaUcucuccagacucu..........................................   4    1    seq
.................ucuccuuugacUucuccagacucuu.........................................   2    1    seq
.................ucuccuuugaccucuccagacucuu.........................................   9    0    seq
.................ucuccuuugaccucuccagacucuG.........................................   1    1    seq
.................ucuccuuugaUcucuccagacucuu.........................................   4    1    seq
.................ucuccuuugaUcucuccagacucuuc........................................   2    1    seq
.................ucuccuuAgaccucuccagacucuuc........................................   1    1    seq
.................ucuccuuugaccucuccagacucuuc........................................   7    0    seq
.................ucuccuuugaccucuccagacucuuU........................................   1    1    seq
.................ucuccuuugaccCcuccagacucuucg.......................................   1    1    seq
.................ucuccuuugaUcucuccagacucuucg.......................................   7    1    seq
.................ucuccuuugaccucuccagacucuucA.......................................   1    1    seq
.................ucuccuuugaccucuccagacucuucC.......................................   1    1    seq
.................ucuccuuugaccucuccagacucuucg.......................................   5    0    seq
.................uUuccuuugaccucuccagacucuucg.......................................   1    1    seq
.................ucuccuuCgaccucuccagacucuucg.......................................   1    1    seq
.................ucuccuuugaccucuccagacucuucU.......................................   4    1    seq
.................ucuccuuugaccucuccagacucuucgUcuggagaa..............................   1    1    seq
.................ucuccuuugaccucuccagacucuucgccuggagaau.............................   1    0    seq
...................uccuuugaccuUuccaga..............................................   1    1    seq
...................uccuuugaccucuccagac.............................................   2    0    seq
...................uccuuugaccucuccagaU.............................................   1    1    seq
...................uccuuugacUucuccagac.............................................   1    1    seq
...................uccuuugaccucuccagacu............................................   3    0    seq
...................uccuuugaccucuccagacucu..........................................   1    0    seq
...................uccuuugaccucuccagacucA..........................................   1    1    seq
...................uccuuugaccucuccagacucuucg.......................................   2    0    seq
...................uccuuugaccucuccagacucuucgU......................................   4    1    seq
.....................cuuugaccucuccagacucuu.........................................   3    0    seq
............................................ccuggagaauuuaaacgaga...................   2    0    seq
............................................ccuggagaauuuaaacgagaa..................   1    0    seq
............................................ccuggagaauuuaaacgagaag.................   2    0    seq
............................................ccuggagGauuuaaacgagaag.................   1    1    seq
.............................................cuggagaauuuaaacgaga...................   1    0    seq
.............................................cuggagaauuuaaacgagGaga................   1    1    seq
```

```
                novel-nve-miR-103-1_guide
                                             novel-nve-miR-103-1_star
uggcaucgagaacuucuucuccuuugaccucuccagacucuucgccuggagaauuuaaacgagaagaaguacucguuacaacg

.............................................cuggagaGuuuaaacgagaaga................    1          1          seq
.............................................cuggagaauuuaaacgagaagG................    1          1          seq
.............................................cuggagaauuuaaacgagaaga................    7          0          seq
.............................................cuggagaauuuaaacgagaagU................    1          1          seq
.............................................cuggagaauuuaaacgagaagaC...............    1          1          seq
```

Starless novel *Nematostella* miRNAs

miRBase precursor : novel-nve-miR-34
Total read count : 2101
novel-nve-miR-34_guide read count : 2101
novel-nve-miR-34_star read count : 0
remaining reads : 0

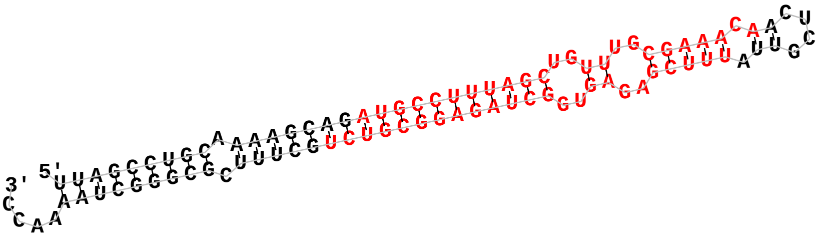

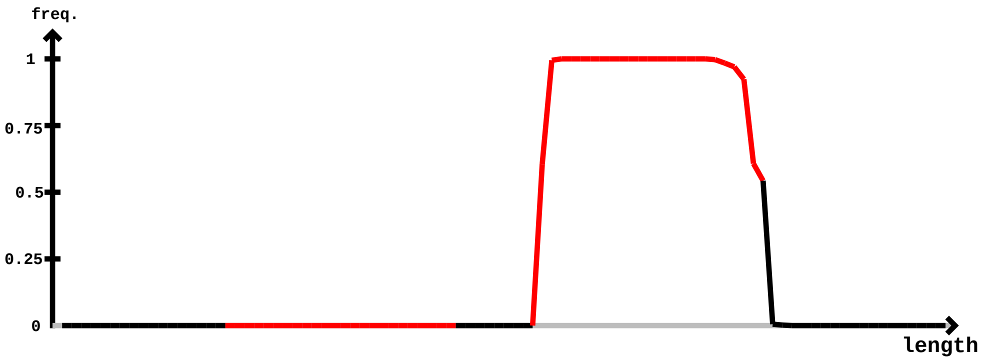

novel-nve-miR-34_guide

novel-nve-miR-34_star

5'- uuagccugcaaaagcagaugccuuuagcuguuugcgaaacaacucguuauuucgagaguggcuagaggcgucugcuuucgcgggcuaaaacc -3' exp

((((((((.((((((((((((((((((..((..(((((.((....)).)))))..))..)))))))))))))))))).)))))))))....	reads	mm	sample
....uuucgagaguggcuagaA.........................	1	1	seq
....uuucgagaguggcuagag.........................	4	0	seq
....uuCcgagaguggcuagag.........................	1	1	seq
....uuucgagaguggcuagagg........................	11	0	seq
....uuucgagaguggcuagagA........................	1	1	seq
....uuucgagaguggcuGgagg........................	2	1	seq
....uuucgagaguggcuagaggU.......................	5	1	seq
....uuucgagaguggcuagaggc.......................	11	0	seq
....uuucgagaguggcuagaggA.......................	1	1	seq
....uuucgagaguggcuagaggcA......................	10	1	seq
....Cuucgagaguggcuagaggcg......................	2	1	seq
....uuucUagaguggcuagaggcg......................	1	1	seq
....uuucgagaguggcuagaggcg......................	35	0	seq
....uuucgagaguggcuagaggcgA.....................	10	1	seq
....uuucgaAaguggcuagaggcgu.....................	1	1	seq
....uuucgagaguggcuagaggcgG.....................	6	1	seq
....uGucgagaguggcuagaggcgu.....................	1	1	seq
....uuucgagaguggcuagaggcgC.....................	45	1	seq
....uuuUgagaguggcuagaggcgu.....................	2	1	seq
....uuucgagGguggcuagaggcgu.....................	3	1	seq
....uuucgagagugUcuagaggcgu.....................	3	1	seq
....uuucgagaguggcuGgaggcgu.....................	3	1	seq
....Auucgagaguggcuagaggcgu.....................	1	1	seq
....uuucgagaguggcuagGggcgu.....................	5	1	seq
....uuuAgagaguggcuagaggcgu.....................	1	1	seq
....uAucgagaguggcuagaggcgu.....................	3	1	seq
....uuucgagaguggUuagaggcgu.....................	1	1	seq
....uuucgagaguggcuagaggcgu.....................	275	0	seq
....uuucgagaguggcuagagUcgu.....................	1	1	seq
....uuucgagagugAcuagaggcgu.....................	2	1	seq
....uuucAagaguggcuagaggcgu.....................	1	1	seq
....Cuucgagaguggcuagaggcgu.....................	1	1	seq
....uCucgagaguggcuagaggcgu.....................	1	1	seq
....uuCcgagaguggcuagaggcgu.....................	3	1	seq

```
                                                  novel-nve-miR-34_guide
                 novel-nve-miR-34_star
uuagccugcaaaagcagaugccuuuagcuguuugcgaaacaacucguuauuucgagaguggcuagaggcgucugcuuucgcgggcuaaaacc

.................................................uuucgagaguggcuagaggUgu.....................    2    1    seq
.................................................uuucgagaguggcCagaggcgu.....................    1    1    seq
.................................................uuucgagaguggcuagaggcguA....................    2    1    seq
.................................................Cuucgagaguggcuagaggcguc....................    1    1    seq
.................................................uuAcgagaguggcuagaggcguc....................    1    1    seq
.................................................uuCcgagaguggcuagaggcguc....................    1    1    seq
.................................................uuucgagaguggcuagaggcguc....................    45   0    seq
.................................................uuucgagaguggcuagaggcguU....................    23   1    seq
.................................................uuucgagaguggUuagaggcguc....................    1    1    seq
.................................................uuucgagaguggcuagaggcgucA...................    61   1    seq
.................................................Cuucgagaguggcuagaggcgucu...................    2    1    seq
.................................................uuucgagaguggcuagaggcgucu...................    513  0    seq
.................................................uuucAagaguggcuagaggcgucu...................    1    1    seq
.................................................uCucgagaguggcuagaggcgucu...................    4    1    seq
.................................................uuucgagaguggcCagaggcgucu...................    2    1    seq
.................................................uuucgagaguggcuagaggcgucG...................    10   1    seq
.................................................uuucgUgaguggcuagaggcgucu...................    1    1    seq
.................................................uuucgagGguggcuagaggcgucu...................    5    1    seq
.................................................uuucgagaguAgcuagaggcgucu...................    1    1    seq
.................................................uuucgagaguggcuagGggcgucu...................    1    1    seq
.................................................Auucgagaguggcuagaggcgucu...................    7    1    seq
.................................................uuucgagaguggcuagaAgcgucu...................    3    1    seq
.................................................uuucgagaguggcuGgaggcgucu...................    1    1    seq
.................................................uuucgagaguggcGagaggcgucu...................    1    1    seq
.................................................uuucgagaguggcuCgaggcgucu...................    1    1    seq
.................................................uuucgagaguggcuagaggcgucC...................    84   1    seq
.................................................uAucgagaguggcuagaggcgucu...................    4    1    seq
.................................................uuucgagagAggcuagaggcgucu...................    1    1    seq
.................................................uuucgagaguggcuagCggcgucu...................    1    1    seq
.................................................uuCcgagaguggcuagaggcgucu...................    3    1    seq
.................................................uuucUagaguggcuagaggcgucu...................    1    1    seq
.................................................uuucgagaguggcuagaggcguUu...................    9    1    seq
.................................................uuucgagagCggcuagaggcgucu...................    5    1    seq
.................................................uuucgagaguggcuagaggcgCcu...................    3    1    seq
.................................................uuucgGgaguggcuagaggcgucu...................    8    1    seq
.................................................uuucgagaguggcuagaggcgucuU..................    4    1    seq
.................................................uuucgagaguggcuagaggcgucuUc.................    4    1    seq
..................................................uuUgagaguggcuagagg........................    1    1    seq
..................................................uucgagaguggcuagagA........................    3    1    seq
..................................................uucgagaguggcuGgagg........................    1    1    seq
..................................................uucgagaguggcuagagg........................    8    0    seq
..................................................uucgagaguggcuagaggU.......................    6    1    seq
..................................................uucgagaguggcuagaggc.......................    6    0    seq
..................................................uucgagaguggcuagaggG.......................    1    1    seq
..................................................Aucgagaguggcuagaggcg......................    1    1    seq
..................................................Cucgagaguggcuagaggcg......................    1    1    seq
..................................................uucgagaguggcuagaCgcg......................    1    1    seq
..................................................uucgagaguggcuagaggcU......................    2    1    seq
..................................................uucgagaguggUuagaggcg......................    1    1    seq
..................................................uucgagaguggcuagaggcA......................    6    1    seq
..................................................uucgagaguggcuagaggcg......................    36   0    seq
..................................................uucgaAaguggcuagaggcg......................    1    1    seq
..................................................uCcgagaguggcuagaggcgu.....................    1    1    seq
..................................................uucgagaguAgcuagaggcgu.....................    2    1    seq
..................................................uucgagaguggcuagaggAgu.....................    1    1    seq
..................................................uucgagaCuggcuagaggcgu.....................    1    1    seq
..................................................uucgagaguggUuagaggcgu.....................    3    1    seq
..................................................uAcgagaguggcuagaggcgu.....................    1    1    seq
..................................................uucgagaguggcuagaggcgu.....................    219  0    seq
..................................................uucgGgaguggcuagaggcgu.....................    1    1    seq
..................................................uucgagaguggcuGgaggcgu.....................    4    1    seq
..................................................Aucgagaguggcuagaggcgu.....................    3    1    seq
..................................................uucgagaAuggcuagaggcgu.....................    1    1    seq
..................................................uuAgagaguggcuagaggcgu.....................    1    1    seq
..................................................uucgagaguggcuagaggcgG.....................    3    1    seq
..................................................uucgagaguggAuagaggcgu.....................    1    1    seq
..................................................uucgagaguggcuagaggcgC.....................    42   1    seq
..................................................uucgagGguggcuagaggcgu.....................    1    1    seq
..................................................uucgagaguggcuagaggcCu.....................    1    1    seq
```

```
                                                 novel-nve-miR-34_guide
                 novel-nve-miR-34_star
uuagccugcaaaagcagaugccuuuagcuguuugcgaaacaacucguuauuucgagaguggcuagaggcgucugcuuucgcgggcuaaaacc

..................................................uucgagaguggcuagaggcgA.....................   5     1    seq
..................................................uucgagagugAcuagaggcguc....................   1     1    seq
..................................................uucgagaguggcuagaggcAuc....................   1     1    seq
..................................................uucgagaguggcuagaggcguA....................   3     1    seq
..................................................uucgagaguggcuagaggcguc....................   38    0    seq
..................................................uucgGgaguggcuagaggcguc....................   2     1    seq
..................................................uucgagaguggcuagaggcguU....................   13    1    seq
..................................................uuUgagaguggcuagaggcguc....................   1     1    seq
..................................................uucgagaguggcuagaggUguc....................   1     1    seq
..................................................uucgagaguggcuagaggcgucG...................   9     1    seq
..................................................uucgagaguggcuagaggcgCcu...................   1     1    seq
..................................................uucgagaguggcuagUggcgucu...................   1     1    seq
..................................................uucgagaguggcuagaggcgGcu...................   1     1    seq
..................................................uucgagaguggcuUgaggcgucu...................   1     1    seq
..................................................uucgagaguggcCagaggcgucu...................   2     1    seq
..................................................uucgagaguggcuagaggcgucC...................   50    1    seq
..................................................uucgagaguggcuagaggcgucA...................   28    1    seq
..................................................uucgagaguggcuagaggcguUu...................   6     1    seq
..................................................uucgagaguggcuagGggcgucu...................   2     1    seq
..................................................uCcgagaguggcuagaggcgucu...................   1     1    seq
..................................................uucgagagAggcuagaggcgucu...................   1     1    seq
..................................................Cucgagaguggcuagaggcgucu...................   1     1    seq
..................................................uucgGgaguggcuagaggcgucu...................   1     1    seq
..................................................uucgagaguggcuagaggcgucu...................   281   0    seq
..................................................uucgagaguggcuCgaggcgucu...................   1     1    seq
..................................................Gucgagaguggcuagaggcgucu...................   2     1    seq
..................................................uucgagaguggcuagaggcgucuA..................   3     1    seq
...................................................uAgagaguggcuagaggcgu.....................   1     1    seq
...................................................uUgagaguggcuagaggcgu.....................   1     1    seq
...................................................uUgagaguggcuagaggcguc....................   1     1    seq
...................................................ucgagaguggcuagaggcgucA...................   1     1    seq
...................................................uUgagaguggcuagaggcgucu...................   1     1    seq
...................................................ucgagaguggcuagaggcgucC...................   1     1    seq
...................................................ucgagaguggcuagaggcgucu...................   5     0    seq
```

Provisional ID		novel-nve-miR-35
Score total	:	6.1
Score for star read(s)	:	-1.3
Score for read counts	:	0
Score for mfe	:	2.8
Score for randfold	:	1.6
Score for cons. seed	:	3
Total read count	:	535
Mature read count	:	535
Loop read count	:	0
Star read count	:	0

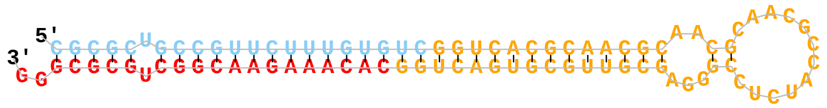

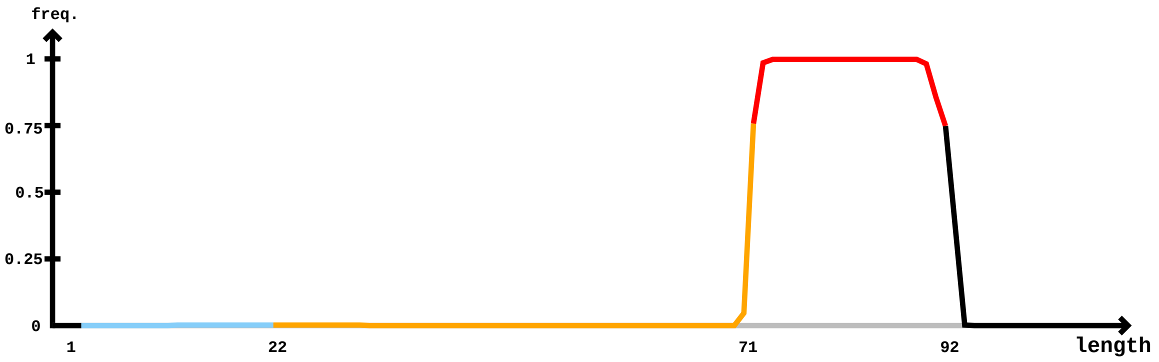

```
     Star                                                                 Mature
5'- ccgcgcugccguucuuugugucggucacgcaacgcaacgcaacgccaucucccgggagcguugcgugacuggcacaaagaacggcugcgcgggagacuacacaggggucgg  -3'   exp
    ((((((.((((((((((((((((((((((((((((..((............))...)))))))))))))))))))))))))))).))))))..((((.......))))..   reads   mm   sample
    ..........uucuuugugucggucacgca................................................................................   1       0    seq
    .....................................................................gcacaaagaacggcugcgcU.....................   1       1    seq
    .....................................................................gcacaaagaacggcugcgcg.....................   4       0    seq
    .....................................................................gcacaaagaacggcugcgcA.....................   1       1    seq
    .....................................................................Ccacaaagaacggcugcgcgg....................   1       1    seq
    .....................................................................Ucacaaagaacggcugcgcgg....................   1       1    seq
    .....................................................................gcacaaagaacggcugcgcgA....................   1       1    seq
    .....................................................................gcacaaagaacggcugcgcgg....................   1       0    seq
    .....................................................................Ccacaaagaacggcugcgcggg...................   1       1    seq
    .....................................................................gcacaaagaacggcugcgcggg...................   8       0    seq
    .....................................................................Ucacaaagaacggcugcgcggg...................   2       1    seq
    .....................................................................gcacaaagaacggcugcgcgCg...................   1       1    seq
    .....................................................................gcacaaagaacggcugcgcggA...................   1       1    seq
    .....................................................................gcaUaaagaacggcugcgcggg...................   1       1    seq
    .....................................................................Acacaaagaacggcugcgcgggag.................   1       1    seq
    ......................................................................cacaaagaacggcugcgU......................   1       1    seq
    ......................................................................cacaaagaacggcugcgc......................   8       0    seq
    ......................................................................cacaaagaacggcugUgcg.....................   2       1    seq
    ......................................................................cacaaagaacggcugcgcC.....................   1       1    seq
    ......................................................................cacaaGgaacggcugcgcg.....................   1       1    seq
    ......................................................................cacaaagaacggcugcgcA.....................   4       1    seq
    ......................................................................cacaaagaCcggcugcgcg.....................   1       1    seq
    ......................................................................cacaaagaacggcCgcgcg.....................   1       1    seq
    ......................................................................cacaaagaacggcugcgcg.....................   42      0    seq
    ......................................................................cacaaagCacggcugcgcgg....................   1       1    seq
    ......................................................................cacaaagaacggcugcgcgg....................   34      0    seq
    ......................................................................cacaaagaacggcugUgcgg....................   1       1    seq
    ......................................................................cacaaagaacggcugcgcgA....................   5       1    seq
    ......................................................................cacaaagaacggUugcgcggg...................   2       1    seq
    ......................................................................cacaaagaacggcugcgcggA...................   19      1    seq
    ......................................................................cacaaagaacggcugcgcgUg...................   1       1    seq
    ......................................................................cacaaagaacggcugUgcggg...................   1       1    seq
    ......................................................................cacaaagaUcggcugcgcggg...................   1       1    seq
    ......................................................................cacaaagaacggcugcgcggC...................   4       1    seq
```

Star Mature

```
ccgcgcugccguucuuugugucggucacgcaacgcaacgcaacgccaucucccgggagcguugcgugacuggcacaaagaacggcugcgcgggagacuacacaggggucgg
.......................................................................cUcaaagaacggcugcgcggg..................   2    1    seq
.......................................................................cacGaagaacggcugcgcggg..................   2    1    seq
.......................................................................cacaaagaCcggcugcgcggg..................   1    1    seq
.......................................................................caGaaagaacggcugcgcggg..................   1    1    seq
.......................................................................cGcaaagaacggcugcgcggg..................   1    1    seq
.......................................................................cacaaagaacggcugcgcggU..................   6    1    seq
.......................................................................cacaaagaacggcugcgcAgg..................   1    1    seq
.......................................................................cacaaagaacggcugcgcggg..................   205  0    seq
.......................................................................cacaaagaacgAcugcgcggg..................   1    1    seq
.......................................................................cacaaagaacggcugcCcggg..................   1    1    seq
.......................................................................cacaaagaacUgcugcgcggg..................   1    1    seq
.......................................................................cacaaagaacggcuCcgcggg..................   1    1    seq
.......................................................................Aacaaagaacggcugcgcggg..................   1    1    seq
.......................................................................cacaaagaacggcuAcgcggga.................   1    1    seq
.......................................................................cacaaagaacggcugcgcgggC.................   3    1    seq
.......................................................................cacaaagaacggcugcgcgggU.................   10   1    seq
.......................................................................cacaaagaacggcugcgcgggG.................   1    1    seq
.......................................................................cacaaagaacggcugcgcggga.................   11   0    seq
.......................................................................cacaaagaGcggcugcgcggga.................   1    1    seq
........................................................................acaaagaacggcugcgcg....................   6    0    seq
........................................................................acaaagaacggcugcgcC....................   1    1    seq
........................................................................acaaagaacggcugcgcA....................   2    1    seq
........................................................................acaaagaacggcugcgcgg...................   9    0    seq
........................................................................aUaaagaacggcugcgcgg...................   1    1    seq
........................................................................acaaagaacggcugcgcgA...................   2    1    seq
........................................................................acaaagaacUgcugcgcgg...................   1    1    seq
........................................................................acaCagaacggcugcgcggg..................   1    1    seq
........................................................................acaaagaacggcugcgcggA..................   1    1    seq
........................................................................acaaagaacggcugcgcggg..................   17   0    seq
........................................................................acGaagaacggcugcgcggg..................   1    1    seq
........................................................................acaaagaaAggcugcgcggga.................   1    1    seq
........................................................................acaaagaacggcugcgcggga.................   20   0    seq
........................................................................acaaagaacggcugcgcgggU.................   50   1    seq
........................................................................Ccaaagaacggcugcgcggga.................   1    1    seq
........................................................................acaaagaacggcugcgcgggC.................   8    1    seq
.........................................................................caaagaacggcugcgcggg..................   2    0    seq
.........................................................................caaagaacggcugcgcggU..................   1    1    seq
.........................................................................caaagaacggcugcgcgggG.................   1    1    seq
.........................................................................caaagaacggcugcgcgggU.................   3    1    seq
```

miRBase precursor : novel-nve-miR-36-1
Total read count : 2592
novel-nve-miR-36-1_guide read count : 2592
novel-nve-miR-36-1_star read count : 0
remaining reads : 0

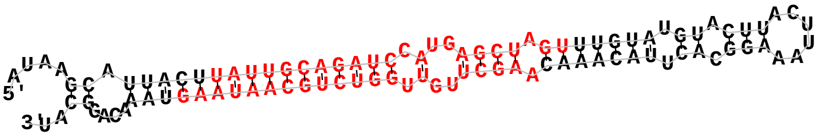

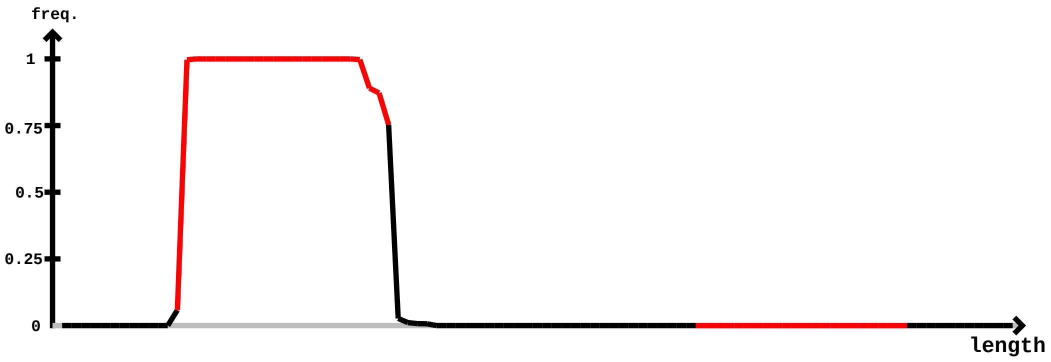

novel-nve-miR-36-1_guide

novel-nve-miR-36-1_star

```
5'- auaagcauuacuuauugcagauccaugagcuaguuuguauguacuuacuuaaaggcacuuacaaacaagcuuguuggucugcaauaaguaaacaggcau -3'    exp
    ....((.((((((((((((((((.(..((((.(((((((.((.(((......))).)).))))))).))))..).))))))))))))))))....))..    reads   mm   sample
    ...........uuauugcagUuccaugagcu....................................................................    1       1    seq
    ...........uuauugcagauccaugagcC....................................................................    4       1    seq
    ...........uuauugcagauccaugagcu....................................................................    6       0    seq
    ...........uuauugcagauccaugagcua...................................................................    5       0    seq
    ...........uuauugcagauccaugagcuaA..................................................................    3       1    seq
    ...........uuauugcagauccaugagcuag..................................................................    8       0    seq
    ...........uuauugcagauccaCgagcuag..................................................................    1       1    seq
    ...........uuauugcagauccaugagcuGg..................................................................    1       1    seq
    ...........uuauugcagauccCugagcuagu.................................................................    1       1    seq
    ...........uuauugcagauccaugaCcuagu.................................................................    1       1    seq
    ...........Cuauugcagauccaugagcuagu.................................................................    1       1    seq
    ...........Auauugcagauccaugagcuagu.................................................................    1       1    seq
    ...........uuauugcagauccaugagcuagu.................................................................    84      0    seq
    ...........uuauugcagauccaugagcuagC.................................................................    16      1    seq
    ...........uuauugcagauccaCgagcuagu.................................................................    1       1    seq
    ...........uuauuAcagauccaugagcuagu.................................................................    1       1    seq
    ...........uuauugcagauccaugagcuGgu.................................................................    2       1    seq
    ...........uuauugcagauccaugagcuagA.................................................................    1       1    seq
    ...........uuauGgcagauccaugagcuagu.................................................................    1       1    seq
    ...........uuauugcagauccaugaAcuagu.................................................................    1       1    seq
    ...........uuauugUagauccaugagcuagu.................................................................    1       1    seq
    ...........uuauugcagauccaugagcuaguu................................................................    4       0    seq
    ...........uuauugcagauccaugagcuaguuu...............................................................    2       0    seq
    ...........uuauugcagauccaugagcuaguuA...............................................................    2       1    seq
    ...........uuauugcagauccaugagcuaguuuAu.............................................................    1       1    seq
    ............uauugcagauccaugagc.....................................................................    5       0    seq
    ............uauugcagauccaugagU.....................................................................    2       1    seq
    ............uauugcagaucAaugagcu....................................................................    1       1    seq
    ............Aauugcagauccaugagcu....................................................................    3       1    seq
    ............uauugcagauccGugagcu....................................................................    1       1    seq
    ............uauugcagauccaCgagcu....................................................................    3       1    seq
    ............uaCugcagauccaugagcu....................................................................    2       1    seq
    ............uauugcagauccaugagcG....................................................................    4       1    seq
    ............uauCgcagauccaugagcu....................................................................    2       1    seq
```

```
           novel-nve-miR-36-1_guide
                                                       novel-nve-miR-36-1_star
auaagcauuacuuauugcagauccaugagcuaguuuguauguacuuacuuaaaggcacuuacaaacaagcuuguuggucugcaauaaguaaacaggcau
```

............uauugcGgauccaugagcu..	3	1	seq
............uauugcagauccaugagcA..	14	1	seq
............uauugcagauccaugagcu..	201	0	seq
............uauugcagauccaugagcC..	20	1	seq
............uauugcagauccaugGgcu..	1	1	seq
............uauugcaUauccaugagcu..	1	1	seq
............uaAugcagauccaugagcu..	1	1	seq
............uauugcagGuccaugagcu..	2	1	seq
............uauugUagauccaugagcu..	3	1	seq
............uauugcagaucUaugagcu..	4	1	seq
............uauugcagauccaugagcGa...	1	1	seq
............uauugcagGuccaugagcua...	1	1	seq
............uauugcagauccaugagcuG...	1	1	seq
............Aauugcagauccaugagcua...	1	1	seq
............uauugcagaAccaugagcua...	1	1	seq
............uauugcagauccaugagcua...	35	0	seq
............uauugcagauccaugagUua...	1	1	seq
............uauugcagauccauUagcua...	1	1	seq
............Aauugcagauccaugagcuag..	3	1	seq
............uauugcagauccaugagcuaC..	1	1	seq
............uauugcagauccaugagcuaU..	4	1	seq
............uauugcUgauccaugagcuag..	1	1	seq
............uauugcagauccaugGgcuag..	1	1	seq
............uauugcagauccauAagcuag..	2	1	seq
............uaAugcagauccaugagcuag..	1	1	seq
............uauugcagauAcaugagcuag..	1	1	seq
............uauugcagauccaugagcGag..	1	1	seq
............uauugcagauccaugagcuGg..	4	1	seq
............uauugcagauccaugagcuaA..	20	1	seq
............uauugcagauccauUagcuag..	1	1	seq
............uauAgcagauccaugagcuag..	2	1	seq
............uauugcagGuccaugagcuag..	2	1	seq
............uauugcGgauccaugagcuag..	1	1	seq
............Cauugcagauccaugagcuag..	3	1	seq
............uauugcagauccaugagcuag..	240	0	seq
............uauuAcagauccaugagcuag..	2	1	seq
............uauugcagaucAaugagcuag..	1	1	seq
............uauugAagauccaugagcuag..	1	1	seq
............uauugcagauccGugagcuag..	2	1	seq
............uauugcagaucAaugagcuagu...	4	1	seq
............uauugcagauccaugagcuagu...	1383	0	seq
............uauugcagauccaGgagcuagu...	1	1	seq
............uauugcagauccGugagcuagu...	4	1	seq
............uauugcagauccaugGgcuagu...	13	1	seq
............uauugcagauccaugagcuaCu...	1	1	seq
............uauugcGgauccaugagcuagu...	14	1	seq
............uauugcagauccaugagcuUgu...	2	1	seq
............uauugcagauccaugCgcuagu...	4	1	seq
............uauugcagauccUugagcuagu...	1	1	seq
............uaGugcagauccaugagcuagu...	1	1	seq
............uauugcagauccaugagcuCgu...	1	1	seq
............uauugcagGuccaugagcuagu...	8	1	seq
............uauugcagaCccaugagcuagu...	13	1	seq
............uauugcagauccaugagcuagG...	17	1	seq
............uauugAagauccaugagcuagu...	1	1	seq
............uauugcagauccaugagUuagu...	6	1	seq
............uaCugcagauccaugagcuagu...	4	1	seq
............uauugcagaucUaugagcuagu...	8	1	seq
............uauugcagauccaugagcuGgu...	13	1	seq
............uauGgcagauccaugagcuagu...	1	1	seq
............uauugcagauccauAagcuagu...	3	1	seq
............uauugcagauUcaugagcuagu...	3	1	seq
............uauugcagauccaugagAuagu...	1	1	seq
............uauugcagauccaugagcuagC...	163	1	seq
............Cauugcagauccaugagcuagu...	4	1	seq
............uauugcagauccaugUgcuagu...	1	1	seq
............uauuCcagauccaugagcuagu...	1	1	seq
............uauugcaAauccaugagcuagu...	1	1	seq
............uauuAcagauccaugagcuagu...	2	1	seq

```
            novel-nve-miR-36-1_guide
                                                                  novel-nve-miR-36-1_star
auaagcauuacuuauugcagauccaugagcuaguuuguauguacuuacuuaaaggcacuuacaaacaagcuuguuggucugcaauaaguaaacaggcau

............uauCgcagauccaugagcuagu.................................................................    5    1    seq
............uauugcagauccaAgagcuagu.................................................................    1    1    seq
............uauugcaUauccaugagcuagu.................................................................    1    1    seq
............uauugcagauccaugagcCagu.................................................................    3    1    seq
............uauugcagauccaugagcuagA.................................................................    28   1    seq
............Gauugcagauccaugagcuagu.................................................................    3    1    seq
............uauAgcagauccaugagcuagu.................................................................    2    1    seq
............uauugUagauccaugagcuagu.................................................................    12   1    seq
............uaAugcagauccaugagcuagu.................................................................    4    1    seq
............uauugcagauccaugagcGagu.................................................................    1    1    seq
............uauugcUgauccaugagcuagu.................................................................    2    1    seq
............uGuugcagauccaugagcuagu.................................................................    2    1    seq
............uauugcagauccaCgagcuagu.................................................................    6    1    seq
............uauugcagauAcaugagcuagu.................................................................    1    1    seq
............Aauugcagauccaugagcuagu.................................................................    17   1    seq
............uauugcagauccaugagcuaguu................................................................    19   0    seq
............uauugcagauccaugagcuaguG................................................................    1    1    seq
............uauugcagauccaugagcuaguA................................................................    7    1    seq
............uauugcagauccaugagcuaguC................................................................    3    1    seq
............uauugcagauccGugagcuaguu................................................................    1    1    seq
............uauugcagauAcaugagcuaguu................................................................    1    1    seq
............uauugUagauccaugagcuaguu................................................................    1    1    seq
............uauugcagauccaugagcuGguu................................................................    1    1    seq
............uauugcagaucUaugagcuaguuu...............................................................    4    1    seq
............uauugcagauccaugagcuaguuu...............................................................    2    0    seq
............uauugcagauccaugagcuaguuuU..............................................................    2    1    seq
............uauugcagauccaugagcuaguuuAu.............................................................    2    1    seq
............uauugcagauccaugagcuaguuuUu.............................................................    14   1    seq
.............auugcagauccaugagcuag..................................................................    1    0    seq
.............auugcagauccaugagcuagu.................................................................    5    0    seq
.............auugcagauccaugagcuaguA................................................................    2    1    seq
```

miRBase precursor : novel-nve-miR-38
Total read count : 720
novel-nve-miR-38_guide read count : 720
novel-nve-miR-38_star read count : 0
remaining reads : 0

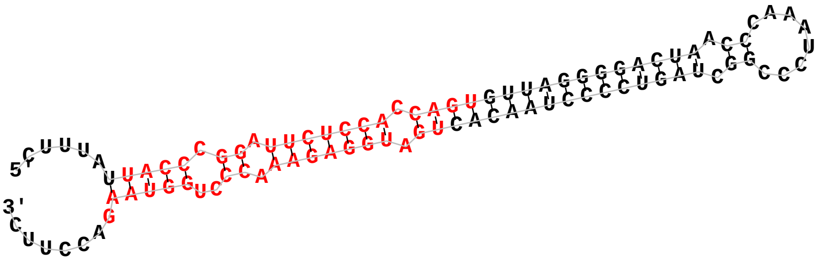

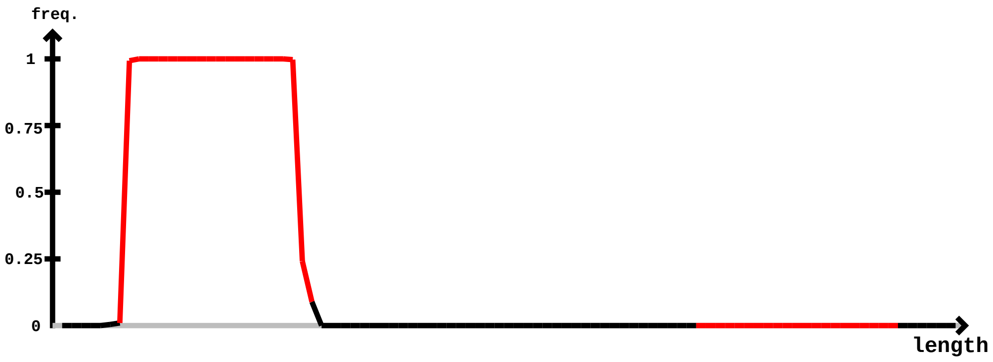


```
                                                                    novel-nve-miR-38_star
        novel-nve-miR-38_guide
5'- cuuuauuacccggauucuccaccaguguuaggggacuaacccaaaucccggcuaguccccuaacacugauggagaaacccugguaagaccuuc   -3'    exp
    .....(((((.((.(((((((.((((((((((((((((.((........)).)))))))))))))))).))))))).))..)))))......   reads     mm       sample
    ....Guuacccggauucuccacc......................................................................   1         1        seq
    ....Guuacccggauucuccacca.....................................................................   2         1        seq
    .....uuacccUgauucuccacc......................................................................   1         1        seq
    .....uuacccggauucuccacca.....................................................................   2         0        seq
    .....Guacccggauucuccacca.....................................................................   1         1        seq
    ......uacccggauucuccaUca.....................................................................   1         1        seq
    ......uacccggauucuccacca.....................................................................   163       0        seq
    ......uacccggauuUuccacca.....................................................................   2         1        seq
    ......Aacccggauucuccacca.....................................................................   1         1        seq
    ......uCcccggauucuccacca.....................................................................   7         1        seq
    ......uacccggauucuccaccU.....................................................................   26        1        seq
    ......uaUccggauucuccacca.....................................................................   4         1        seq
    ......uacccggauucuccacAa.....................................................................   1         1        seq
    ......uacAcggauucuccacca.....................................................................   1         1        seq
    ......uacccggauucuccGcca.....................................................................   1         1        seq
    ......uacccggauucuccaccC.....................................................................   7         1        seq
    ......Cacccggauucuccacca.....................................................................   2         1        seq
    ......uacccggaCucuccacca.....................................................................   2         1        seq
    ......uacccggauucuccaccG.....................................................................   245       1        seq
    ......uUcccggauucuccacca.....................................................................   55        1        seq
    ......uGcccggauucuccacca.....................................................................   1         1        seq
    ......uacccggGuucuccacca.....................................................................   16        1        seq
    ......uacccggauucCccacca.....................................................................   1         1        seq
    ......uacccggauCcuccacca.....................................................................   1         1        seq
    ......uacccggauucuAcacca.....................................................................   1         1        seq
    ......uacccggauucuccUcca.....................................................................   1         1        seq
    ......uaccUggauucuccaccag....................................................................   1         1        seq
    ......uacccggGuucuccaccag....................................................................   1         1        seq
    ......uCcccggauucuccaccag....................................................................   1         1        seq
    ......uacccggauucuccaccaC....................................................................   14        1        seq
    ......uacccggauucuccaccGg....................................................................   1         1        seq
    ......uacccggauuUuccaccag....................................................................   1         1        seq
    ......uacccggauucuccaccag....................................................................   13        0        seq
    ......uacccggauucuccacUag....................................................................   1         1        seq
```

```
                                                                  novel-nve-miR-38_star
      novel-nve-miR-38_guide
cuuuauuacccggauucuccaccaguguuaggggacuaacccaaaucccggcuaguccccuaacacugauggagaaacccugguaagaccuuc

......uacccggauucuccaccCg....................................................................    1    1    seq
......uacccggauucuccaccaA....................................................................    7    1    seq
......uUcccggauucuccaccag....................................................................    12   1    seq
......uacccggauucuccaccaU....................................................................    48   1    seq
......uacccggauucuccaccUg....................................................................    8    1    seq
......uacccggauucucUaccag....................................................................    1    1    seq
......uacccggauucuccaccaUu...................................................................    52   1    seq
......uacccggauucuccaccaCu...................................................................    7    1    seq
.......acccggauucuccaccaCu...................................................................    1    1    seq
.......acccggauucuccaccaUu...................................................................    4    1    seq
```

miRBase precursor : novel-nve-miR-39-1
Total read count : 1113
novel-nve-miR-39-1_guide read count : 1112
novel-nve-miR-39-1_star read count : 0
remaining reads : 1

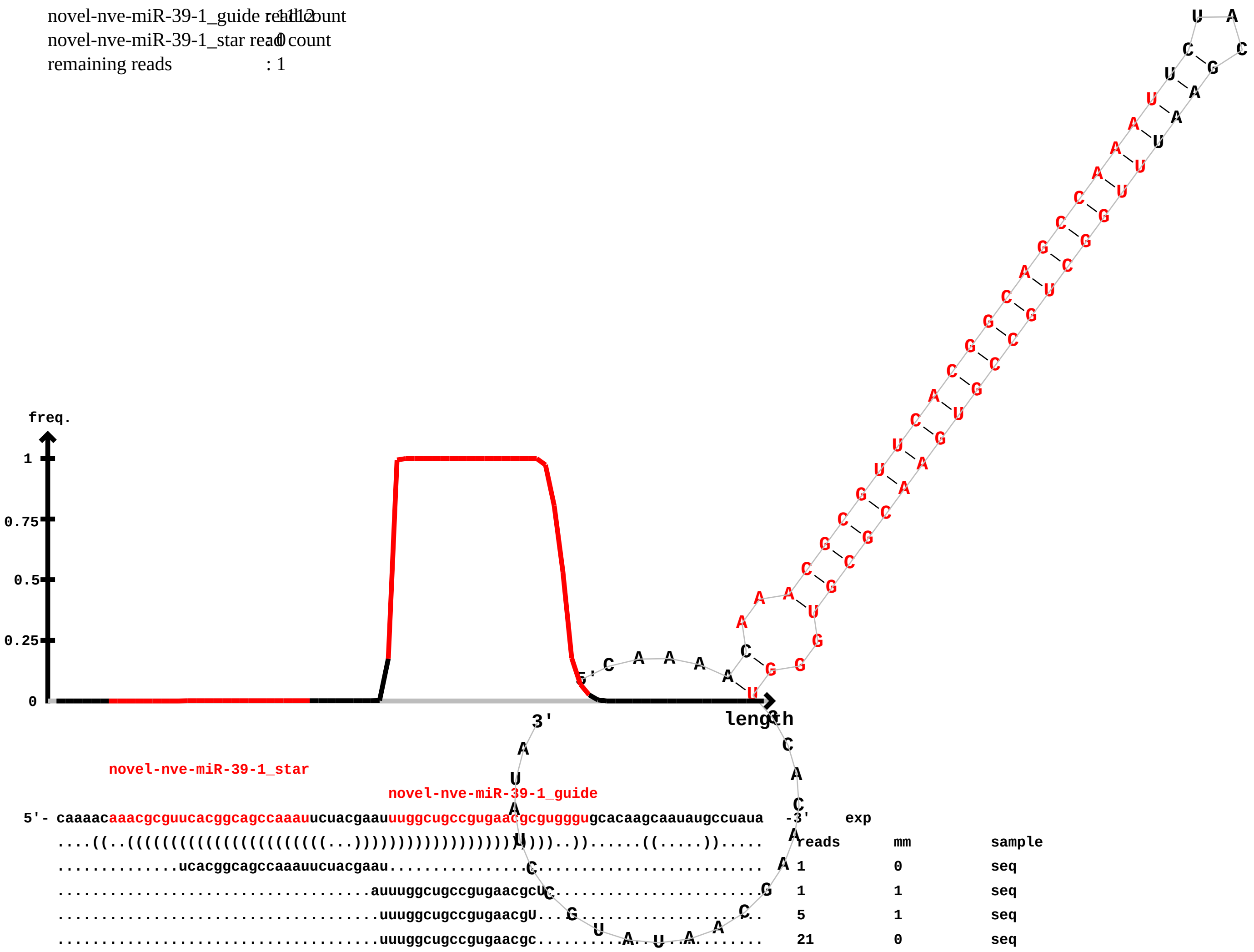

novel-nve-miR-39-1_star

novel-nve-miR-39-1_guide

5'- caaaacaaacgcguucacggcagccaaauucuacgaauuuggcugccgugaacgcgugggugcacaagcaauaugccuaua -3'	exp		
....((..(((((((((((((((((((((((...)))))))))))))))))))))))..))......((.....)).....	reads	mm	sample
..............ucacggcagccaaauucuacgaau...	1	0	seq
....................................auuuggcugccgugaacgcU.........................	1	1	seq
.....................................uuuggcugccgugaacgU..........................	5	1	seq
.....................................uuuggcugccgugaacgc..........................	21	0	seq
.....................................uuuggcugccgAgaacgc..........................	1	1	seq
.....................................uuuggcugccgugaacgA..........................	2	1	seq
.....................................uuugCcugccgugaacgcg.........................	1	1	seq
.....................................uuuggcugccgugaacgcA.........................	7	1	seq
.....................................uCuggcugccgugaacgcg.........................	2	1	seq
.....................................uuuggcugccgugaacgcg.........................	39	0	seq
.....................................uuuggcugcUgugaacgcg.........................	1	1	seq
.....................................uuuggcugccgugaacgcU.........................	1	1	seq
.....................................uuuggcugccgugaacgcgu........................	50	0	seq
.....................................uuuggcugccgugGacgcgu........................	1	1	seq
.....................................Auuggcugccgugaacgcgu........................	1	1	seq
.....................................uuuggcugccgugaacgcgA........................	1	1	seq
.....................................uuuggcugccgugaacgcgG........................	1	1	seq
.....................................uuuggcugccgugaacgcgC........................	9	1	seq
.....................................uuuggcugccgugaGcgcgu........................	1	1	seq
.....................................uuuggAugccgugaacgcgu........................	1	1	seq
.....................................uuuggcugccgugaacgcgug.......................	10	0	seq
.....................................uuuggcugccgugaacgcguC.......................	2	1	seq
.....................................uuuggcugccgugaacgcguU.......................	21	1	seq
.....................................uuuggcugccgugaacgcguA.......................	3	1	seq
.....................................uuuggcugccgugaacgcgCg.......................	1	1	seq
.....................................uuuggcugccgugaacgcgugU......................	4	1	seq
.....................................uuuggcugccgugaacgcgugg......................	3	0	seq
.....................................uuuggcugccgugaacgcgugA......................	1	1	seq
.....................................uuuggcugccgugaacgcgugggC....................	1	1	seq
.....................................uuuggcugccgugaacgcgugggu....................	1	0	seq
......................................Cuggcugccgugaacgcg.........................	1	1	seq
......................................uuggcugccgugaCcgcg.........................	1	1	seq
......................................uuggcugccgugaacgcU.........................	4	1	seq
......................................uuggcugccgugaacgcA.........................	15	1	seq

```
      novel-nve-miR-39-1_star
                                      novel-nve-miR-39-1_guide
caaaacaaacgcguucacggcagccaaauucuacgaauuuggcugccgugaacgcguggguGcacaagcaauaugccuaua

......................................uuggcugUcgugaacgcg.........................    1     1     seq
......................................uuggcugccgCgaacgcg.........................    1     1     seq
......................................uuggcugccgugaGcgcg.........................    2     1     seq
......................................uuggcugccgugaacgcg.........................    102   0     seq
......................................uuggcugccgugaUcgcg.........................    1     1     seq
......................................uuggcugccgugaacgcC.........................    3     1     seq
......................................uuggcCgccgugaacgcg.........................    1     1     seq
......................................Auggcugccgugaacgcg.........................    1     1     seq
......................................uuggcugcAgugaacgcg.........................    1     1     seq
......................................uuUgcugccgugaacgcg.........................    1     1     seq
......................................uuggcugcUgugaacgcgu........................    1     1     seq
......................................uuggcAgccgugaacgcgu........................    1     1     seq
......................................uuggAugccgugaacgcgu........................    1     1     seq
......................................uuggcugccgugaGcgcgu........................    2     1     seq
......................................uuAgcugccgugaacgcgu........................    1     1     seq
......................................uuggcugccgugaacgcgC........................    30    1     seq
......................................uuggcCgccgugaacgcgu........................    1     1     seq
......................................uuggcugccgugaacgGgu........................    2     1     seq
......................................uuggcugccgugaacgcgu........................    181   0     seq
......................................uCggcugccgugaacgcgu........................    1     1     seq
......................................Auggcugccgugaacgcgu........................    1     1     seq
......................................uuggcugccgugaacAcgu........................    1     1     seq
......................................uuggcugccgugaacgcgA........................    11    1     seq
......................................uuggcugccgugaacgcgG........................    5     1     seq
......................................uuggcugccgugaacgcgug.......................    262   0     seq
......................................uuggcAgccgugaacgcgug.......................    2     1     seq
......................................uuggcugccgugaacgcguA.......................    28    1     seq
......................................uuggUugccgugaacgcgug.......................    1     1     seq
......................................uuggAugccgugaacgcgug.......................    2     1     seq
......................................uuggcugccgugaacgcgCg.......................    3     1     seq
......................................uugUcugccgugaacgcgug.......................    1     1     seq
......................................uuggcCgccgugaacgcgug.......................    1     1     seq
......................................uAggcugccgugaacgcgug.......................    1     1     seq
......................................uuggcugccgugaaUgcgug.......................    1     1     seq
......................................uuggcugccgugaacgcguU.......................    36    1     seq
......................................uuggcugccguUaacgcgug.......................    2     1     seq
......................................uuggcugccgCgaacgcgug.......................    4     1     seq
......................................uuggcugccgGgaacgcgug.......................    1     1     seq
......................................uuggcugccgugaacgcgGg.......................    2     1     seq
......................................uuggcugccgugaacgcguC.......................    2     1     seq
......................................uuggcugccgugaGcgcgug.......................    1     1     seq
......................................Guggcugccgugaacgcgug.......................    2     1     seq
......................................Cuggcugccgugaacgcgug.......................    1     1     seq
......................................uuggcugccguCaacgcgug.......................    1     1     seq
......................................Auggcugccgugaacgcgug.......................    2     1     seq
......................................uuggcugccgugaaUgcgugg......................    1     1     seq
......................................uuggcugcAgugaacgcgugg......................    1     1     seq
......................................uuggcugccgugaacgcgugg......................    71    0     seq
......................................uuggcugccgugaacgcgugC......................    6     1     seq
......................................uuggcugcUgugaacgcgugg......................    1     1     seq
......................................uuggcugccgugaacgcgugA......................    13    1     seq
......................................uuggcCgccgugaacgcgugg......................    1     1     seq
......................................uuggcugccgugaacgcgugU......................    15    1     seq
......................................uuggcugccgugaacgUgugg......................    1     1     seq
......................................uuggcugccgugaacgcgugCg.....................    1     1     seq
......................................uuggcugccgugaacgcguggU.....................    15    1     seq
......................................uuggcugccgugaacgcguggg.....................    24    0     seq
......................................uuggcugcAgugaacgcguggg.....................    1     1     seq
......................................uuggcugccgugaacgcguggA.....................    4     1     seq
......................................uuggcugccgugaacgcguggC.....................    2     1     seq
......................................uuggcugccgugaacgcgugggC....................    6     1     seq
......................................uuggcugcUgugaacgcgugggu....................    1     1     seq
......................................uuggcugccgugaacgcgugggA....................    2     1     seq
......................................uuggcugccgugaacgcgugggu....................    12    0     seq
......................................uuggcugccgugaacgcgugggCg...................    1     1     seq
......................................uuggcugccgugaacgcguggguU...................    2     1     seq
......................................uuggcugccgugaacgcguggug...................    1     0     seq
......................................uuggcugccgugaacgcguggguA...................    1     1     seq
.......................................uggcugccgugaacgcgA........................    1     1     seq
```

```
     novel-nve-miR-39-1_star
                                      novel-nve-miR-39-1_guide
caaaacaaacgcguucacggcagccaaauucuacgaauuuggcugccgugaacgcgugggugcacaagcaauaugccuaua

.......................................uggcugccgugaacgcguU.......................    1          1          seq
.......................................uggcugccgugaacgcgugg......................    2          0          seq
.......................................uggcugccgugaacgcgugA......................    1          1          seq
.......................................uggcugccgugaacgcguggC.....................    1          1          seq
```

miRBase precursor : novel-nve-miR-44-1
Total read count : 2038
novel-nve-miR-44-1_guide read count : 2038
novel-nve-miR-44-1_star read count : 0
remaining reads : 0

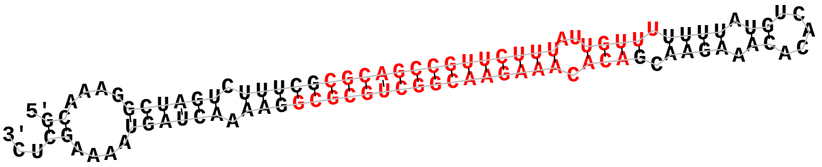

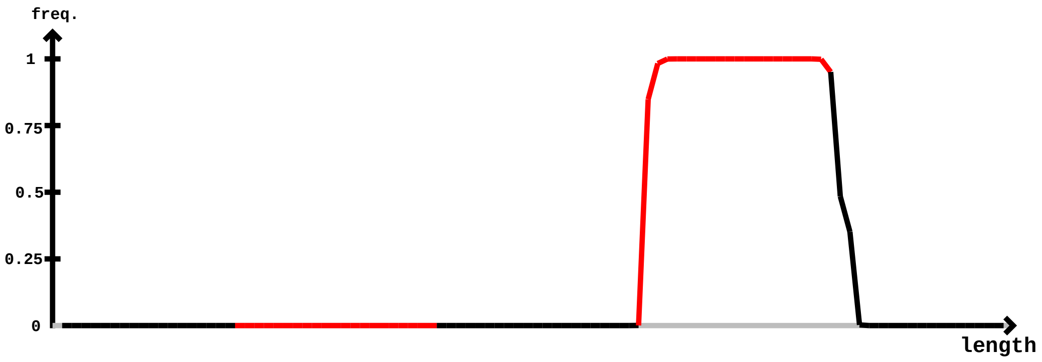


```
                                                                       novel-nve-miR-44-1_star
                                                                                                         novel-nve-miR-44-1_guide
5'- gcaaaggcuagucuuucgcgcagccguucuuuauuguuuuuuuaugucacacaaagaacgacacaaagaacggcugcgcggaaaacuaguaaaagcuc  -3'   exp
    ((....((((((.((((((((((((((((((..((((.((((.((.....)).)))).)))).)))))))))))))))))).))))))....))..   reads   mm   sample
    ...........................................................Cacacaaagaacggcugcgcg..................   1       1    seq
    ............................................................acacaaagaacggcugcU....................   1       1    seq
    ............................................................acacaaagaacggcugcg....................   2       0    seq
    ............................................................acacaaagaacggcugcUc...................   1       1    seq
    ............................................................acacaaagaacggcugcgU...................   22      1    seq
    ............................................................acacaaUgaacggcugcgc...................   1       1    seq
    ............................................................acacaaagaacggcugcgG...................   2       1    seq
    ............................................................acacaaagaacggcugcgc...................   55      0    seq
    ............................................................Ucacaaagaacggcugcgc...................   1       1    seq
    ............................................................acacGaagaacggcugcgc...................   1       1    seq
    ............................................................acacaaagaacggcugcgA...................   3       1    seq
    ............................................................acacaaagCacggcugcgcg..................   2       1    seq
    ............................................................acacaaagaGcggcugcgcg..................   1       1    seq
    ............................................................aUacaaagaacggcugcgcg..................   1       1    seq
    ............................................................acacaaagaacggcugcgcg..................   575     0    seq
    ............................................................acacaaagaacggcugUgcg..................   1       1    seq
    ............................................................acacaaaCaacggcugcgcg..................   1       1    seq
    ............................................................acacaaagaacggUugcgcg..................   1       1    seq
    ............................................................acacaaagaCcggcugcgcg..................   1       1    seq
    ............................................................acacaaagaaAggcugcgcg..................   1       1    seq
    ............................................................acGcaaagaacggcugcgcg..................   1       1    seq
    ............................................................acacaaagaacggcugcgcC..................   11      1    seq
    ............................................................acacaaagaacggAugcgcg..................   1       1    seq
    ............................................................acacaaagaaUggcugcgcg..................   2       1    seq
    ............................................................acUcaaagaacggcugcgcg..................   1       1    seq
    ............................................................acaAaaagaacggcugcgcg..................   1       1    seq
    ............................................................Ccacaaagaacggcugcgcg..................   3       1    seq
    ............................................................acacaaagaacggcuAcgcg..................   1       1    seq
    ............................................................acacaaagaacggcugAgcg..................   1       1    seq
    ............................................................acacaaagaacggcugcgcA..................   147     1    seq
    ............................................................acacaaagaacgCcugcgcg..................   1       1    seq
    ............................................................acacaaagaacggcAgcgcg..................   1       1    seq
    ............................................................acacaaagaacgUcugcgcg..................   1       1    seq
    ............................................................acacaaagaacggcugcgcU..................   34      1    seq
```

```
                     novel-nve-miR-44-1_star
                                                                  novel-nve-miR-44-1_guide
gcaaaggcuagucuuucgcgcagccguucuuuauuguuuuuuuaugucacacaaagaacgacacaaagaacggcugcgcggaaaacuaguaaaagcuc

............................................................aAacaaagaacggcugcgcg..................   1     1     seq
............................................................acacaaagaacggcugcgAg..................   2     1     seq
............................................................acacaaagaacggcugcCcg..................   1     1     seq
............................................................acacaaagaacggcugcUcg..................   1     1     seq
............................................................acacaaUgaacggcugcgcg..................   2     1     seq
............................................................acacaaagaacggcugcAcg..................   3     1     seq
............................................................acacaaagaacgAcugcgcg..................   3     1     seq
............................................................acacaaGgaacggcugcgcg..................   2     1     seq
............................................................acacaaagaacggcugcgUg..................   2     1     seq
............................................................acacaGagaacggcugcgcg..................   3     1     seq
............................................................acacaaagaacggcugcgGg..................   4     1     seq
............................................................acacaaagaaAggcugcgcgg.................   1     1     seq
............................................................acGcaaagaacggcugcgcgg.................   1     1     seq
............................................................acacaaagaacggcugcgcgU.................   12    1     seq
............................................................acacaaagaacggcugcgUgg.................   1     1     seq
............................................................acacaaagaacggcugcgcgg.................   140   0     seq
............................................................acacaaagaacggcugcgcgC.................   9     1     seq
............................................................acacGaagaacggcugcgcgg.................   1     1     seq
............................................................acacaaagCacggcugcgcgg.................   1     1     seq
............................................................acacaaGgaacggcugcgcgg.................   1     1     seq
............................................................acacaaagaacgCcugcgcgg.................   1     1     seq
............................................................acacaaagaacggcugcgGgg.................   2     1     seq
............................................................acacaaagaacggcugcgcgA.................   34    1     seq
............................................................acaAaaagaacggcugcgcgg.................   1     1     seq
............................................................acUcaaagaacggcugcgcgga................   1     1     seq
............................................................acacaCagaacggcugcgcgga................   1     1     seq
............................................................acacaaagaacggcugcgcggU................   129   1     seq
............................................................acacaaaAaacggcugcgcgga................   1     1     seq
............................................................acaAaaagaacggcugcgcgga................   1     1     seq
............................................................acacaaagaacggcugcgAgga................   2     1     seq
............................................................acacaaagaacggcugcgcggC................   64    1     seq
............................................................acacaGagaacggcugcgcgga................   6     1     seq
............................................................acacaaagaacggcugcgcgUa................   3     1     seq
............................................................acacaaaCaacggcugcgcgga................   1     1     seq
............................................................acacaaagaacggcugcCcgga................   1     1     seq
............................................................acacaaagaacAgcugcgcgga................   2     1     seq
............................................................acacaaagaacggAugcgcgga................   1     1     seq
............................................................acacaaagaacgCcugcgcgga................   2     1     seq
............................................................Gcacaaagaacggcugcgcgga................   1     1     seq
............................................................acacaaagaacggcugcgGgga................   2     1     seq
............................................................Ccacaaagaacggcugcgcgga................   1     1     seq
............................................................acGcaaagaacggcugcgcgga................   1     1     seq
............................................................aAacaaagaacggcugcgcgga................   1     1     seq
............................................................acacaaagaacggGugcgcgga................   1     1     seq
............................................................acacaaGgaacggcugcgcgga................   1     1     seq
............................................................acacaaagaacggcAgcgcgga................   1     1     seq
............................................................acacaaagaacggUugcgcgga................   1     1     seq
............................................................acacaaagaGcggcugcgcgga................   1     1     seq
............................................................acacaaagaacggcuAcgcgga................   1     1     seq
............................................................acacaaagaacggcCgcgcgga................   2     1     seq
............................................................acacaaagGacggcugcgcgga................   1     1     seq
............................................................acacaaagaacggcugcgUgga................   2     1     seq
............................................................acacaaagaacCgcugcgcgga................   1     1     seq
............................................................Ucacaaagaacggcugcgcgga................   1     1     seq
............................................................acacaaagaacggcugcgcgCa................   3     1     seq
............................................................acacaaagaaUggcugcgcgga................   2     1     seq
............................................................acacaaagaacUgcugcgcgga................   2     1     seq
............................................................acacaaagaacggcugcgcgga................   373   0     seq
............................................................acacaaagaacggcugAgcgga................   2     1     seq
............................................................acacaaagaacggcugUgcgga................   1     1     seq
............................................................acacaaagaacggcugcgcggaG...............   1     1     seq
............................................................acacaaagaacggcugcgcggCa...............   1     1     seq
............................................................acacaaagaacggcugcgcggUa...............   1     1     seq
.............................................................cacaaagaacggcugcgc...................   7     0     seq
.............................................................cacaaagaacggcugcgU...................   3     1     seq
.............................................................cacaaagaacggcugcgA...................   1     1     seq
.............................................................cacaaagaacggcugcgcg..................   90    0     seq
.............................................................cacaaagaacggcugcgGg..................   2     1     seq
.............................................................cacaaagaacggcugcgUg..................   1     1     seq
```

```
                  novel-nve-miR-44-1_star
                                                            novel-nve-miR-44-1_guide
gcaaaggcuagucuuucgcgcagccguucuuuauuguuuuuuuaugucacacaaagaacgacacaaagaacggcugcgcggaaaacuaguaaaagcuc

.............................................................cacaaagaacggcugcgcU..................    3     1     seq
.............................................................cacaaagaCcggcugcgcg..................    1     1     seq
.............................................................cacaaagGacggcugcgcg..................    1     1     seq
.............................................................cacUaagaacggcugcgcg..................    1     1     seq
.............................................................Uacaaagaacggcugcgcg..................    1     1     seq
.............................................................cacaaagaacggcugcgcA..................    18    1     seq
.............................................................cacaaagaacggcugcgcgC.................    3     1     seq
.............................................................cacaaagaacggcugcgcgA.................    12    1     seq
.............................................................cacaaagaacggcugcgcgg.................    36    0     seq
.............................................................cacaaagaacggcugcgcgU.................    2     1     seq
.............................................................cacaGagaacggcugcgcgg.................    1     1     seq
.............................................................cacUaagaacggcugcgcgga................    1     1     seq
.............................................................cacaaagaacggcugcgcgga................    46    0     seq
.............................................................cacaaagaacggcugcgUgga................    2     1     seq
.............................................................cacaaGgaacggcugcgcgga................    2     1     seq
.............................................................cacaaagaacggcugcgcgUa................    1     1     seq
.............................................................cacaaagaacggcugcgcggC................    7     1     seq
.............................................................cGcaaagaacggcugcgcgga................    1     1     seq
.............................................................cacaaagaacggcugcAcgga................    1     1     seq
.............................................................cacaaagaacggcugcgcggU................    26    1     seq
.............................................................cacaaagaacggcugcgcUga................    1     1     seq
.............................................................cacaaagaaAggcugcgcgga................    1     1     seq
.............................................................caUaaagaacggcugcgcgga................    1     1     seq
.............................................................cacaaagaacggcugcgcggCa...............    1     1     seq
..............................................................Ccaaagaacggcugcgcg..................    1     1     seq
..............................................................acaaaCaacggcugcgcg..................    1     1     seq
..............................................................acaaagaacggcugcgcA..................    5     1     seq
..............................................................acaaagaacggcugcgcg..................    11    0     seq
..............................................................acaaagaacggcugcgcgA.................    4     1     seq
..............................................................acaaagaacggcugcgcgU.................    3     1     seq
..............................................................acaaagaacggcugcgcgg.................    4     0     seq
..............................................................acaaagaacggcugcgcggC................    1     1     seq
..............................................................acaaagaacggcugcgcggU................    1     1     seq
..............................................................acaaagaacggcugcgcgga................    2     0     seq
..............................................................acaaagaacggcugcgcggaU...............    1     1     seq
...............................................................caaagaacggcugcgcggU................    1     1     seq
```

miRBase precursor : novel-nve-miR-44-2
Total read count : 13570
novel-nve-miR-44-2_guide read count : 13556
novel-nve-miR-44-2_star read count : 0
remaining reads : 14

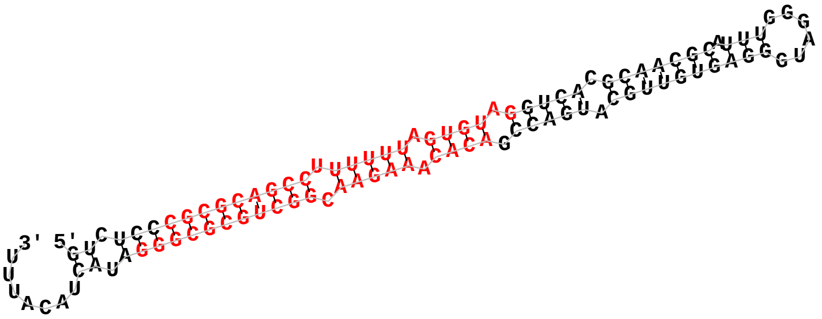

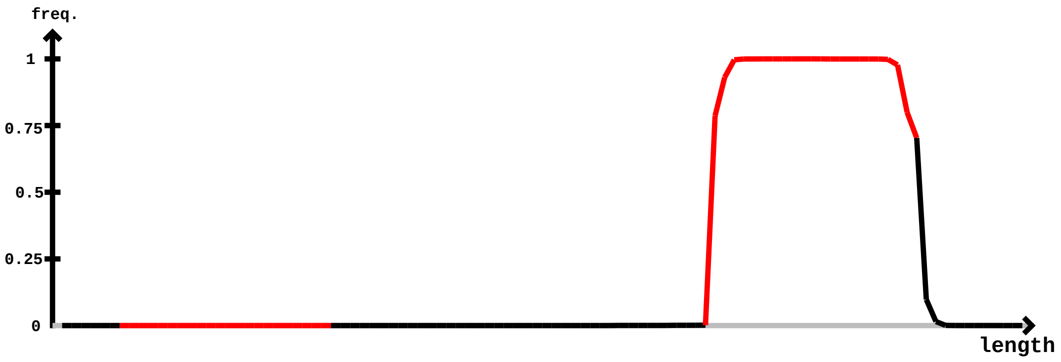


```
                                                                  novel-nve-miR-44-2_guide
        novel-nve-miR-44-2_star
5'- gucucccgcgcagccuuuuuuaguguaggucacgcaacgcauuugggaugggaguguugcaugaccgacacaaagaacggcugcgcgggauacuacauuu -3'   exp
    ((.((((((((((((.(((((.((((.(((((.(((((((.(((......)))))))))).))))).)))).))))).))))))))))).)).......  reads  mm  sample
    .........................................................ugcGugaccgacacaaagaac......................  1      1   seq
    .........................................................ugcGugaccgacacaaagaacg.....................  4      1   seq
    ..............................................................gaccgacacaaagaacggcugcgcgg............  1      0   seq
    ..............................................................gaccgacacaaagaacggcugcgcUgg...........  1      1   seq
    ...............................................................acUgacacaaagaacggcug.................  1      1   seq
    ...............................................................accgacacaaagaacggcugcg...............  1      0   seq
    ...............................................................accgacacaaagaacggcugcgU..............  4      1   seq
    ................................................................cgacacaaagaacggcugcg................  1      0   seq
    ................................................................cgacacaaagaacggcugcgG...............  1      1   seq
    .................................................................gacacaaagaacggcugc.................  1      0   seq
    .................................................................gacacaaagGacggcugcgc...............  2      1   seq
    ..................................................................Cacacaaagaacggcugcgcg.............  1      1   seq
    ..................................................................Cacacaaagaacggcugcgcggg...........  3      1   seq
    ...................................................................acacaaagaacggcugcA...............  2      1   seq
    ...................................................................acacaaagaacggcugcU...............  1      1   seq
    ...................................................................acacaaagaacggcugcg...............  14     0   seq
    ...................................................................acacaaagaacggcAgcgc..............  1      1   seq
    ...................................................................acacGaagaacggcugcgc..............  1      1   seq
    ...................................................................acacaaagGacggcugcgc..............  2      1   seq
    ...................................................................acacaaagaacggUugcgc..............  1      1   seq
    ...................................................................acacaaagaacggcuAcgc..............  1      1   seq
    ...................................................................acacaaagaaUggcugcgc..............  1      1   seq
    ...................................................................acacaaagaacggcugcgU..............  52     1   seq
    ...................................................................Ucacaaagaacggcugcgc..............  1      1   seq
    ...................................................................acacaaagaacggcugcgc..............  142    0   seq
    ...................................................................acacaaagaacggcugcUc..............  1      1   seq
    ...................................................................acacaaagaacggcugcgA..............  11     1   seq
    ...................................................................acacaaagaacggcugcgG..............  5      1   seq
    ...................................................................acacaaUgaacggcugcgc..............  1      1   seq
    ...................................................................acacaaagaacggcugcCcg.............  1      1   seq
    ...................................................................acacaaagaacggcugcgcA.............  336    1   seq
    ...................................................................acacaaagaacggcugcgcC.............  21     1   seq
    ...................................................................acacaaagaacggcCgcgcg.............  1      1   seq
    ...................................................................acacaaagaacggcugcgUg.............  5      1   seq
```

novel-nve-miR-44-2_guide

novel-nve-miR-44-2_star

gucucccgcgcagccuuuuuuaguguaggucacgcaacgcauuugggaugggaguguugcaugaccgacacaaagaacggcugcgcgggauacuacauuu

....acacaaagaacggcugGgcg............. 1 1 seq
....acacaGagaacggcugcgcg............. 6 1 seq
....acacaaagaacggcugcAcg............. 4 1 seq
....acacaaagCacggcugcgcg............. 3 1 seq
....acacaaagaacggcugcUcg............. 1 1 seq
....acacGaagaacggcugcgcg............. 3 1 seq
....acacaaagaacggcugcgcg............. 1461 0 seq
....Ccacaaagaacggcugcgcg............. 4 1 seq
....acacaaagaacggcAgcgcg............. 1 1 seq
....acacaaagaacggcugcgGg............. 4 1 seq
....acacaaagaaAggcugcgcg............. 1 1 seq
....acacaaagaacggcugAgcg............. 2 1 seq
....acacaaagaacAgcugcgcg............. 1 1 seq
....acacaaagaacggcuAcgcg............. 2 1 seq
....acacaaagGacggcugcgcg............. 4 1 seq
....acUcaaagaacggcugcgcg............. 2 1 seq
....acacaaagaGcggcugcgcg............. 5 1 seq
....acacaaagaacggcugcgcU............. 70 1 seq
....acacaaagaCcggcugcgcg............. 1 1 seq
....acacaaagaacggcugUgcg............. 5 1 seq
....acacaaUgaacggcugcgcg............. 2 1 seq
....aAacaaagaacggcugcgcg............. 4 1 seq
....aUacaaagaacggcugcgcg............. 4 1 seq
....acacaaagaacggAugcgcg............. 2 1 seq
....acacaaagaacgCcugcgcg............. 1 1 seq
....acaUaaagaacggcugcgcg............. 4 1 seq
....acacaaagaacgAcugcgcg............. 4 1 seq
....acacaaagaacggUugcgcg............. 3 1 seq
....acacaaagaacggcugcgAg............. 6 1 seq
....acaAaaagaacggcugcgcg............. 4 1 seq
....acacaaagaacCgcugcgcg............. 3 1 seq
....acacaaaCaacggcugcgcg............. 1 1 seq
....acCcaaagaacggcugcgcg............. 1 1 seq
....acacaaCgaacggcugcgcg............. 1 1 seq
....acacaaagaaUggcugcgcg............. 4 1 seq
....acGcaaagaacggcugcgcg............. 6 1 seq
....acacaaagaacgUcugcgcg............. 1 1 seq
....acacaaGgaacggcugcgcg............. 3 1 seq
....acacaaagaacggcugcgGgg............ 4 1 seq
....Ccacaaagaacggcugcgcgg............ 1 1 seq
....acacaaagaacggcugcgcgA............ 128 1 seq
....acacaaagaacggcugAgcgg............ 1 1 seq
....acacaaagaaAggcugcgcgg............ 2 1 seq
....acacaaagaacggcugcgcgU............ 60 1 seq
....acacaaGgaacggcugcgcgg............ 3 1 seq
....acacaaagaacgCcugcgcgg............ 1 1 seq
....acacaaagaacggcugcgcUg............ 2 1 seq
....aAacaaagaacggcugcgcgg............ 1 1 seq
....acacaaagaacggcugcgUgg............ 4 1 seq
....acacaaagaacggAugcgcgg............ 2 1 seq
....acacaGagaacggcugcgcgg............ 2 1 seq
....acGcaaagaacggcugcgcgg............ 5 1 seq
....acacaaagaacggcugcgcCg............ 2 1 seq
....acacaaagaacggcugUgcgg............ 1 1 seq
....acacaaagaGcggcugcgcgg............ 1 1 seq
....acacaaagaacggcCgcgcgg............ 2 1 seq
....acacaaagaacggcugcgcAg............ 2 1 seq
....acacaaagaacggcugcgcgg............ 672 0 seq
....acacaaagCacggcugcgcgg............ 2 1 seq
....acaAaaagaacggcugcgcgg............ 1 1 seq
....acacGaagaacggcugcgcgg............ 1 1 seq
....acacaaagaacggcugcAcgg............ 1 1 seq
....acacaaaUaacggcugcgcgg............ 1 1 seq
....Ucacaaagaacggcugcgcgg............ 1 1 seq
....acacaaagaacggcugcgcgC............ 22 1 seq
....acacaaagaaUggcugcgcgg............ 1 1 seq
....acacaaagaacggUugcgcgg............ 3 1 seq
....acacaaagaCcggcugcgcgg............ 1 1 seq
....acacaaagaacggcugcgcgAg........... 15 1 seq

novel-nve-miR-44-2_guide

novel-nve-miR-44-2_star

gucucccgcgcagccuuuuuuaguguaggucacgcaacgcauuugggaugggaguguugcaugaccgacacaaagaacggcugcgcgggauacuacauuu

....acacaaagaaUggcugcgcggg...........	12	1	seq
....acacaaagaacggcugcgAggg...........	13	1	seq
....acacaaaUaacggcugcgcggg...........	1	1	seq
....acacaaagaacggcugcCcggg...........	4	1	seq
....acacaaagGacggcugcgcggg...........	44	1	seq
....acacaaagaacggcugcgcAgg...........	20	1	seq
....acacaGagaacggcugcgcggg...........	20	1	seq
....acacaaagaacggcugcgUggg...........	6	1	seq
....acacaaagaGcggcugcgcggg...........	18	1	seq
....acacaaagaacgAcugcgcggg...........	2	1	seq
....acacaaagaacggcugGgcggg...........	2	1	seq
....acacaaagaaGggcugcgcggg...........	1	1	seq
....acacaaagaacggcCgcgcggg...........	13	1	seq
....acacaaagaacggcugcgcggg...........	5999	0	seq
....acacaaagaacggAugcgcggg...........	5	1	seq
....acacaaagaacggcugcUcggg...........	4	1	seq
....acacaaagaacggcugcgcggU...........	307	1	seq
....acacaaagaacgUcugcgcggg...........	8	1	seq
....aAacaaagaacggcugcgcggg...........	7	1	seq
....aGacaaagaacggcugcgcggg...........	2	1	seq
....acacUaagaacggcugcgcggg...........	5	1	seq
....Ucacaaagaacggcugcgcggg...........	10	1	seq
....aUacaaagaacggcugcgcggg...........	17	1	seq
....acacaaaCaacggcugcgcggg...........	4	1	seq
....acacaaCgaacggcugcgcggg...........	2	1	seq
....acacaaaAaacggcugcgcggg...........	3	1	seq
....acacaaagaacggcugcgcCgg...........	10	1	seq
....acacaUagaacggcugcgcggg...........	7	1	seq
....acacaaagaacggcAgcgcggg...........	7	1	seq
....acacaaagaacggcugUgcggg...........	27	1	seq
....acacaaagaacgCcugcgcggg...........	8	1	seq
....acacaaagCacggcugcgcggg...........	10	1	seq
....acacaaagaacggcuCcgcggg...........	2	1	seq
....acacaaagaacggcuUcgcggg...........	9	1	seq
....acacaaagUacggcugcgcggg...........	3	1	seq
....acacaaagaacggcugcgGggg...........	4	1	seq
....acacaaagaacggcugcgcUgg...........	2	1	seq
....acacGaagaacggcugcgcggg...........	31	1	seq
....acCcaaagaacggcugcgcggg...........	1	1	seq
....acacaaagaCcggcugcgcggg...........	4	1	seq
....acacaaagaacggcugcgcgUg...........	26	1	seq
....acUcaaagaacggcugcgcggg...........	6	1	seq
....acacaaagaacCgcugcgcggg...........	7	1	seq
....acacaaagaacggcGgcgcggg...........	1	1	seq
....acGcaaagaacggcugcgcggg...........	26	1	seq
....acacaaagaacAgcugcgcggg...........	15	1	seq
....acacaaagaacggcuAcgcggg...........	7	1	seq
....acacaaagaUcggcugcgcggg...........	4	1	seq
....acacaaUgaacggcugcgcggg...........	3	1	seq
....acacaaagaacggUugcgcggg...........	8	1	seq
....Ccacaaagaacggcugcgcggg...........	22	1	seq
....acacaaGgaacggcugcgcggg...........	23	1	seq
....acacaaagaacggcugAgcggg...........	10	1	seq
....acaUaaagaacggcugcgcggg...........	13	1	seq
....acaAaaagaacggcugcgcggg...........	12	1	seq
....acacaaagaacggcugcAcggg...........	9	1	seq
....acacaaagaacggcugcgcggC...........	125	1	seq
....acacaaagaacUgcugcgcggg...........	6	1	seq
....acacaaagaacggcugcgcgCg...........	39	1	seq
....acacaaagaaAggcugcgcggg...........	12	1	seq
....acaUaaagaacggcugcgcggga..........	2	1	seq
....acacaaagaacggcugcgcgUga..........	1	1	seq
....acacaaagaGcggcugcgcggga..........	1	1	seq
....acacaaagaacggcugcgUggga..........	1	1	seq
....acacaaagaacggcugcgcgggU..........	162	1	seq
....acacaGagaacggcugcgcggga..........	1	1	seq
....acacaaagaacggcugcgcgggG..........	14	1	seq
....acacaaagaacggcugcgcggUa..........	4	1	seq
....acacaaagaacAgcugcgcggga..........	2	1	seq

```
                                                                   novel-nve-miR-44-2_guide
      novel-nve-miR-44-2_star
gucucccgcgcagccuuuuuuaguguaggucacgcaacgcauuugggaugggaguguugcaugaccgacacaaagaacggcugcgcgggauacuacauuu

...................................................................acacaaagCacggcugcgcggga..........    1      1      seq
...................................................................acacaaagaacggcugcgcggCa..........    1      1      seq
...................................................................acacaaagaacggcugcgcgggC..........    52     1      seq
...................................................................acacaaagaacggcugcgcggga..........    151    0      seq
...................................................................acGcaaagaacggcugcgcggga..........    2      1      seq
...................................................................acacaaagaacggcuAcgcggga..........    1      1      seq
...................................................................acacaaGgaacggcugcgcggga..........    1      1      seq
...................................................................acacaaagaacggcugcgcgggaA.........    3      1      seq
...................................................................acacaaagaacggcugcgcgggaG.........    1      1      seq
...................................................................acacaaagaacggcugcgcgggUu.........    28     1      seq
...................................................................acacaaagaacggcugcgcgggau.........    2      0      seq
...................................................................acacaaagaacggcugcgcgggauU........    1      1      seq
...................................................................acacaaagaacggcugcgcgggauC........    1      1      seq
...................................................................acacaaagaacggcugcgcgggaGa........    1      1      seq
...................................................................acacaaagaacggcugcgcgggauUc.......    1      1      seq
...................................................................acacaaagaacggcugcgcgggauaU.......    1      1      seq
....................................................................cacaaagaacggAugcgc..............    1      1      seq
....................................................................cacaaagaacggcugcgA..............    3      1      seq
....................................................................cacaaagaacggcugcgc..............    42     0      seq
....................................................................cacaaGgaacggcugcgc..............    1      1      seq
....................................................................cacaaagaacggcugcgU..............    7      1      seq
....................................................................cacaaagaacggcugcgGg.............    2      1      seq
....................................................................cacaaagaacggcugcgcU.............    11     1      seq
....................................................................cacaaagaacggcCgcgcg.............    1      1      seq
....................................................................Uacaaagaacggcugcgcg.............    2      1      seq
....................................................................cacaGagaacggcugcgcg.............    1      1      seq
....................................................................cacaaagaacggcugcgcC.............    6      1      seq
....................................................................cacGaagaacggcugcgcg.............    1      1      seq
....................................................................cGcaaagaacggcugcgcg.............    1      1      seq
....................................................................cacaaagGacggcugcgcg.............    1      1      seq
....................................................................cacaaGgaacggcugcgcg.............    1      1      seq
....................................................................cacaaagaacggcugcCcg.............    1      1      seq
....................................................................cacaaagaacggcugcgcg.............    271    0      seq
....................................................................cacaaagaacggcugcgUg.............    1      1      seq
....................................................................cacaaagaGcggcugcgcg.............    1      1      seq
....................................................................caAaaagaacggcugcgcg.............    1      1      seq
....................................................................cacaaagaacggcugUgcg.............    2      1      seq
....................................................................cacUaagaacggcugcgcg.............    1      1      seq
....................................................................cacaaagaacggcugcgcA.............    60     1      seq
....................................................................cacaaagaCcggcugcgcg.............    3      1      seq
....................................................................cacaaagaacggcugcgcgA............    43     1      seq
....................................................................cacaaagaacCgcugcgcgg............    1      1      seq
....................................................................cacaaagCacggcugcgcgg............    1      1      seq
....................................................................cacaaagaaAggcugcgcgg............    1      1      seq
....................................................................cacaaagaacggcugcgcgg............    240    0      seq
....................................................................cacaaagaUcggcugcgcgg............    1      1      seq
....................................................................cUcaaagaacggcugcgcgg............    1      1      seq
....................................................................cacaaagaacggcugUgcgg............    2      1      seq
....................................................................cGcaaagaacggcugcgcgg............    1      1      seq
....................................................................cacaaagGacggcugcgcgg............    1      1      seq
....................................................................cacaaagaacggcugcgcgU............    11     1      seq
....................................................................cacaaagaacggcugcgGgg............    2      1      seq
....................................................................cacaaagaacggcugcgcAg............    1      1      seq
....................................................................cacaaagaacggcuAcgcgg............    1      1      seq
....................................................................cacaaagaacggcugcgcUg............    1      1      seq
....................................................................cacaGagaacggcugcgcgg............    1      1      seq
....................................................................cacaaagaacggcugcgcgC............    6      1      seq
....................................................................Uacaaagaacggcugcgcgg............    1      1      seq
....................................................................Uacaaagaacggcugcgcggg...........    2      1      seq
....................................................................cacaaagaacggcugUgcggg...........    4      1      seq
....................................................................caGaaagaacggcugcgcggg...........    1      1      seq
....................................................................cacaaagaacggcAgcgcggg...........    1      1      seq
....................................................................cacaaagaacggcugcgcggC...........    20     1      seq
....................................................................cacaaGgaacggcugcgcggg...........    2      1      seq
....................................................................cacaaagaGcggcugcgcggg...........    4      1      seq
....................................................................cacaaagGacggcugcgcggg...........    3      1      seq
....................................................................cacaaagaacggcuCcgcggg...........    1      1      seq
....................................................................cacaaagaacggAugcgcggg...........    1      1      seq
....................................................................cUcaaagaacggcugcgcggg...........    4      1      seq
```

novel-nve-miR-44-2_guide

novel-nve-miR-44-2_star

gucucccgcgcagccuuuuuuaguguaggucacgcaacgcauuugggaugggaguguugcaugaccgacacaaagaacggcugcgcgggauacuacauuu

....Aacaaagaacggcugcgcggg...........	2	1	seq
....cacUaagaacggcugcgcggg...........	1	1	seq
....cacGaagaacggcugcgcggg...........	3	1	seq
....cacaaagaUcggcugcgcggg...........	1	1	seq
....cacaaagaacggcugcCcggg...........	2	1	seq
....cacaaagaacggcugcgAggg...........	3	1	seq
....caUaaagaacggcugcgcggg...........	1	1	seq
....cacaaagaacggcugcgcAgg...........	4	1	seq
....cacaaagaacggcugcgcgCg...........	6	1	seq
....cacaaagaacggcugcgcggU...........	62	1	seq
....cacaaagaacggcugcgcCgg...........	1	1	seq
....cacaaagaacggcugcgcggg...........	922	0	seq
....cacaaagaacgAcugcgcggg...........	2	1	seq
....cacaaagaacggcugAgcggg...........	1	1	seq
....cacaaagaacggcugcAcggg...........	1	1	seq
....cacaaagaacggUugcgcggg...........	3	1	seq
....cacaaagaCcggcugcgcggg...........	2	1	seq
....cacaaagaacUgcugcgcggg...........	1	1	seq
....cCcaaagaacggcugcgcggg...........	1	1	seq
....cacaGagaacggcugcgcggg...........	2	1	seq
....cacaaagaacggcugcgcgUg...........	4	1	seq
....cacaaagaacggcugcgcgAg...........	2	1	seq
....cacaaAaacggcugcgcggg...........	1	1	seq
....cGcaaagaacggcugcgcggg...........	4	1	seq
....cacaaagaGcggcugcgcggga..........	1	1	seq
....cacaaagaacggcugcgcggga..........	40	0	seq
....cacaaagaacggcugcgcgggG..........	3	1	seq
....cacaaagaacggcugcgcgggU..........	62	1	seq
....cacaaagaacggcuAcgcggga..........	1	1	seq
....cacaaagaacggcugcgcgggC..........	19	1	seq
....caUaaagaacggcugcgcggga..........	1	1	seq
....cacaaagaacggcugcgcggUa..........	1	1	seq
....cacaaagaacggcugcgcggCa..........	1	1	seq
....cacaGagaacggcugcgcggga..........	1	1	seq
....Gacaaagaacggcugcgcgggau.........	1	1	seq
....cacaaagaacggcugcgcgggau.........	1	0	seq
....cacaaagaacggcugcgcgggUu.........	17	1	seq
....acaaagaacAgcugcgcg.............	1	1	seq
....acaaagaacggcugcgcA.............	8	1	seq
....acaaagaacggcugcgcg.............	30	0	seq
....acaaaCaacggcugcgcg.............	1	1	seq
....acaaagaacggcugcgcC.............	2	1	seq
....Ccaaagaacggcugcgcg.............	1	1	seq
....acaaagaacggcugcgcU.............	1	1	seq
....acaaagaacggcugcgcgC............	1	1	seq
....acaaagaacggcugcgcgA............	12	1	seq
....acaaagaacggcugcgcgU............	7	1	seq
....Gcaaagaacggcugcgcgg............	1	1	seq
....acaaagaacUgcugcgcgg............	1	1	seq
....acaaagaacggcugcCcgg............	1	1	seq
....aUaaagaacggcugcgcgg............	1	1	seq
....acaaagaacggcugcgcgg............	30	0	seq
....acaaagaacggcugcgGggg...........	1	1	seq
....acaaagaacggcugcAcggg...........	1	1	seq
....acaaagaacggcugcgcggC...........	3	1	seq
....acaaagaacAgcugcgcggg...........	1	1	seq
....acaaagaacggcugcgcggU...........	4	1	seq
....acGaagaacggcugcgcggg...........	2	1	seq
....acaaagaacggcugUgcggg...........	1	1	seq
....acaaagaacggcugcgcggg...........	75	0	seq
....acaCagaacggcugcgcggg...........	1	1	seq
....Gcaaagaacggcugcgcggg...........	1	1	seq
....acaaagaacggAugcgcggg...........	1	1	seq
....acaaagaacgAcugcgcggg...........	1	1	seq
....acaaagaacAgcugcgcggga..........	1	1	seq
....acaaagaacggcugcgcgggG..........	11	1	seq
....Ccaaagaacggcugcgcggga..........	4	1	seq
....acaaagaacggcugcgcgggU..........	385	1	seq
....acaaagaacggcugcgcggga..........	72	0	seq

```
                                                                    novel-nve-miR-44-2_guide
      novel-nve-miR-44-2_star
gucucccgcgcagccuuuuuuaguguaggucacgcaacgcauuugggaugggaguguugcaugaccgacacaaagaacggcugcgcgggauacuacauuu

.....................................................................acaaagaacggcCgcgcgga..........    1     1     seq
.....................................................................acaaagaacggcugcgcgggC..........    89    1     seq
.....................................................................acaaagaaAggcugcgcggga..........    1     1     seq
.....................................................................acaaagaaUggcugcgcggga..........    1     1     seq
.....................................................................Gcaaagaacggcugcgcggga..........    1     1     seq
.....................................................................acaaagaacggcugcgcgggCu.........    2     1     seq
.....................................................................acaaagaacggcugcgcgggUu.........    123   1     seq
.....................................................................acaaagaacggcugcgcgggau.........    9     0     seq
.....................................................................acaaagaacggcugcgcgggaA.........    1     1     seq
.....................................................................acaaagaacggcugcgcgggUua........    3     1     seq
.....................................................................acaaagaacggcugcgcgggauC........    1     1     seq
......................................................................caaagaUcggcugcgcggg...........    1     1     seq
......................................................................caaagaacggcugcgcggU...........    3     1     seq
......................................................................caaagaacggcugcgcggg...........    4     0     seq
......................................................................caaagaacggcugcgcggC...........    1     1     seq
......................................................................caaagaacggUugcgcggga..........    1     1     seq
......................................................................caaagaacAgcugcgcggga..........    1     1     seq
......................................................................caaagaacggcugcgcgggC..........    4     1     seq
......................................................................caaagaacggcugcgcggga..........    7     0     seq
......................................................................caaagaacggcugcgcgggG..........    1     1     seq
......................................................................caaagaacggcugcgcgggU..........    11    1     seq
......................................................................caaagaacggcugcgcgggUu.........    3     1     seq
......................................................................caaagaacggcugcgcgggaC.........    1     1     seq
......................................................................caaagaacggcugcgcgggau.........    2     0     seq
.......................................................................aaagaacggcugcgcggg...........    1     0     seq
.......................................................................aaagaacggcugcgcgggUu.........    1     1     seq
........................................................................aagaacggcuUcgcggga..........    3     1     seq
............................................................................acggcugcgcgggaGacuaca...    1     1     seq
```

miRBase precursor : novel-nve-miR-50
Total read count : 998
novel-nve-miR-50_guide read count : 998
novel-nve-miR-50_star read count : 0
remaining reads : 0

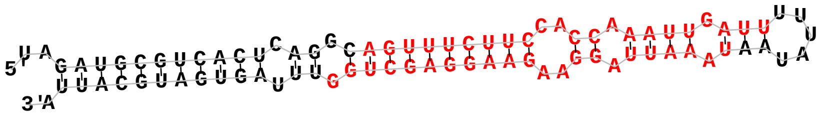

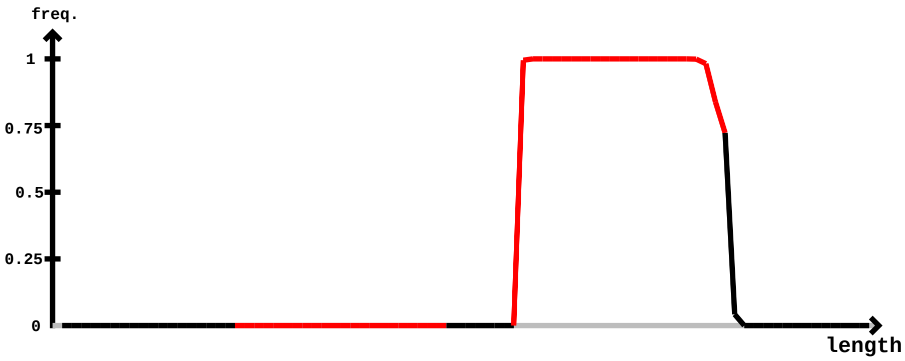


```
                                      novel-nve-miR-50_star
                                                                  novel-nve-miR-50_guide
5'- uagaugcgucacucaggcaguuucuuccaccaaauugauuuuuauaauaaauuaggaagaaggagcugguuuagugaugcauua   -3'    exp
    ..(((((((((((.((.((((((((((..((.((((.(((.....))).)))).))..)))))))))).)).))))))))))).    reads     mm        sample
    ...............................................uaaauuaggaagaaggag...................    1         0         seq
    ...............................................uaaauuaggaagaaggagU..................    4         1         seq
    ...............................................uaaauuaggaagaaggagc..................    12        0         seq
    ...............................................uaaauuaggaagaaggagcA.................    13        1         seq
    ...............................................uGaauuaggaagaaggagcu.................    1         1         seq
    ...............................................uaaauuaggaagaaggUgcu.................    1         1         seq
    ...............................................uaGauuaggaagaaggagcu.................    1         1         seq
    ...............................................uaaauuaggaagaaggagcC.................    13        1         seq
    ...............................................uaaauuaggaagaaggGgcu.................    1         1         seq
    ...............................................Aaaauuaggaagaaggagcu.................    2         1         seq
    ...............................................uaaauuaggaGgaaggagcu.................    4         1         seq
    ...............................................uaaGuuaggaagaaggagcu.................    1         1         seq
    ...............................................uaaauuaggaagaaggagcu.................    100       0         seq
    ...............................................uUaauuaggaagaaggagcu.................    1         1         seq
    ...............................................uaaauuaggaagaaggagcG.................    2         1         seq
    ...............................................uaaaCuaggaagaaggagcu.................    1         1         seq
    ...............................................uaaauAaggaagaaggagcu.................    1         1         seq
    ...............................................uaaauuaggaagaaggagcCg................    3         1         seq
    ...............................................uaaauuaggaagaaggagcuU................    3         1         seq
    ...............................................uaaauuaggaagaGggagcug................    1         1         seq
    ...............................................uaaauuaggaagaaggagcuC................    2         1         seq
    ...............................................uaaauuaggaagaaggGgcug................    3         1         seq
    ...............................................Aaaauuaggaagaaggagcug................    2         1         seq
    ...............................................Caaauuaggaagaaggagcug................    1         1         seq
    ...............................................uaaauuaggaagaaggagcuA................    14        1         seq
    ...............................................uaaauuaggaagGaggagcug................    1         1         seq
    ...............................................uaaaAuaggaagaaggagcug................    1         1         seq
    ...............................................uaaauuaggaagaaggaUcug................    1         1         seq
    ...............................................uaaauuaggaagaaggagcug................    81        0         seq
    ...............................................uaaauuaggaagaaggagcGg................    2         1         seq
    ...............................................uaaGuuaggaagaaggagcugg...............    1         1         seq
    ...............................................Gaaauuaggaagaaggagcugg...............    2         1         seq
    ...............................................uaaauuaggaagaaggGgcugg...............    2         1         seq
    ...............................................uaaauuGggaagaaggagcugg...............    2         1         seq
```

```
                  novel-nve-miR-50_star
                                                 novel-nve-miR-50_guide
uagaugcgucacucaggcaguuucuuccaccaaauugauuuuuauaauaaauuaggaagaaggagcugguuuagugaugcauua

...............................................uaaauuaggaagGaggagcugg...............    3      1      seq
...............................................uGaauuaggaagaaggagcugg...............    1      1      seq
...............................................Caaauuaggaagaaggagcugg...............    1      1      seq
...............................................Aaaauuaggaagaaggagcugg...............    9      1      seq
...............................................uaaaAuaggaagaaggagcugg...............    1      1      seq
...............................................uaaauuaggaagaGggagcugg...............    1      1      seq
...............................................uaaauuaggaagaaggagcCgg...............    2      1      seq
...............................................uaaauuaggaagaaggagcugU...............    35     1      seq
...............................................uaaCuuaggaagaaggagcugg...............    1      1      seq
...............................................uaaauuaggaagaaggagcugC...............    121    1      seq
...............................................uaaauuaggaagaaggagcugA...............    43     1      seq
...............................................uaaauuaggaagaaggagUugg...............    1      1      seq
...............................................uaaauuaggGagaaggagcugg...............    2      1      seq
...............................................uaaauuaAgaagaaggagcugg...............    2      1      seq
...............................................uaaauuaggaGgaaggagcugg...............    17     1      seq
...............................................uaGauuaggaagaaggagcugg...............    2      1      seq
...............................................uaaauAaggaagaaggagcugg...............    1      1      seq
...............................................uaaauuagCaagaaggagcugg...............    3      1      seq
...............................................uaaauuaggaagaaggagcugg...............    421    0      seq
...............................................uaaauuagAaagaaggagcugg...............    1      1      seq
...............................................uaaaGuaggaagaaggagcugg...............    1      1      seq
...............................................uaaauuaggaagaaggagcAgg...............    1      1      seq
...............................................uaaauuaggaagaaggagcugCu..............    28     1      seq
...............................................uaaauuaggaagaagUagcuggu..............    1      1      seq
...............................................uaaauuaggaagaaggagcuggu..............    11     0      seq
...............................................uaaauuaggaagaaggagcuggA..............    1      1      seq
...............................................uaaauuaggaagaaggagcuggC..............    1      1      seq
................................................aaauuaggaagaaggagU..................    1      1      seq
................................................Uaauuaggaagaaggagcu.................    1      1      seq
................................................aaauuaggaagaaggagcug................    1      0      seq
................................................aaauuaggaagaaggagcugg...............    2      0      seq
```

miRBase precursor : novel-nve-miR-67
Total read count : 353
novel-nve-miR-67_guide read count : 353
novel-nve-miR-67_star read count : 0
remaining reads : 0

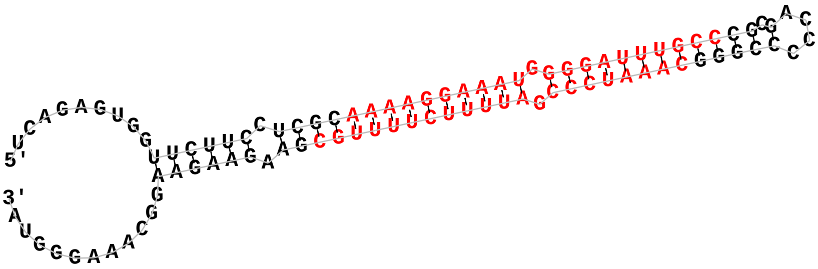

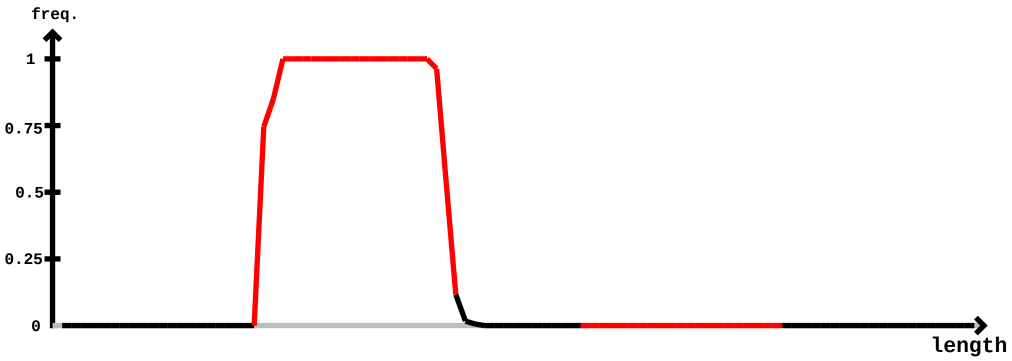


```
                                                              novel-nve-miR-67_star
                         novel-nve-miR-67_guide
5'- ucagagugguucuuccucgcaaaaggaaauggggauuugcccgcgacccccgggcaaaucccgauuuucuuuugcgaagaagaaggcaaagggua  -3'   exp
    .....((..((((((.((((((((((((((.((((((((((((.(....))))))))))))).)))))))))))))).)))))).))........    reads   mm   sample
    ....................aaaaggaaauggggauuG.........................................................    1       1    seq
    ....................aaaaggaaauCgggauuu.........................................................    12      1    seq
    ....................aaaaggaaauggggauuug........................................................    2       0    seq
    ....................aaaaggaaauCgggauuug........................................................    123     1    seq
    ....................aaaaggaaauggggauuuA........................................................    1       1    seq
    ....................aaaaggaaauUgggauuugc.......................................................    1       1    seq
    ....................aaaaggaaauggggauuugc.......................................................    2       0    seq
    ....................aaaaggaaauCgggauuugc.......................................................    99      1    seq
    ....................aaaaggaaauggggauuugU.......................................................    1       1    seq
    ....................aaaaggaaauggggauuugcc......................................................    2       0    seq
    ....................aaaaggaaauCgggauuugcc......................................................    16      1    seq
    ....................aaaaggaaauggggauuugcU......................................................    1       1    seq
    ....................aaaaggaaauCgggauuugccc.....................................................    2       1    seq
    .....................aaaggaaauggggauuCg........................................................    2       1    seq
    .....................aaaggaaauCgggauuug........................................................    16      1    seq
    .....................aaaggaaauggggauuug........................................................    7       0    seq
    .....................aaaggaaauCgggauuugc.......................................................    3       1    seq
    .....................aaaggaaauggggauuugc.......................................................    4       0    seq
    .....................aaaggaaauCgggauuugcc......................................................    2       1    seq
    .....................aaaggaaauggggauuugcG......................................................    1       1    seq
    .....................aaaggaaauggggauuugccU.....................................................    1       1    seq
    .....................aaaggaaauggggauuugccG.....................................................    1       1    seq
    ......................aaggaaauCgggauuugc.......................................................    37      1    seq
    ......................aaggaaauggggauuugG.......................................................    1       1    seq
    ......................aaggaaauCgggauuugcc......................................................    13      1    seq
    ......................aaggaaauCgggauuugcccg....................................................    1       1    seq
    ......................aaggaaauggggauuugcccg....................................................    1       0    seq
```

miRBase precursor : novel-nve-miR-70
Total read count : 122
novel-nve-miR-70_guide read count : 122
novel-nve-miR-70_star read count : 0
remaining reads : 0

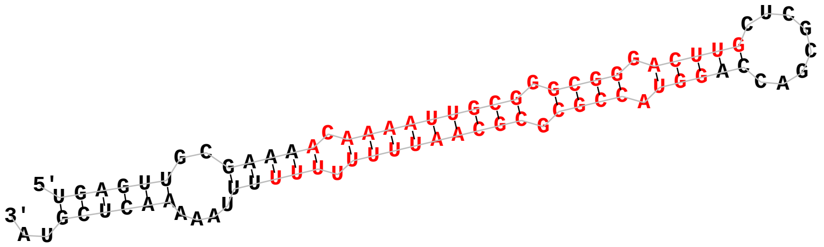

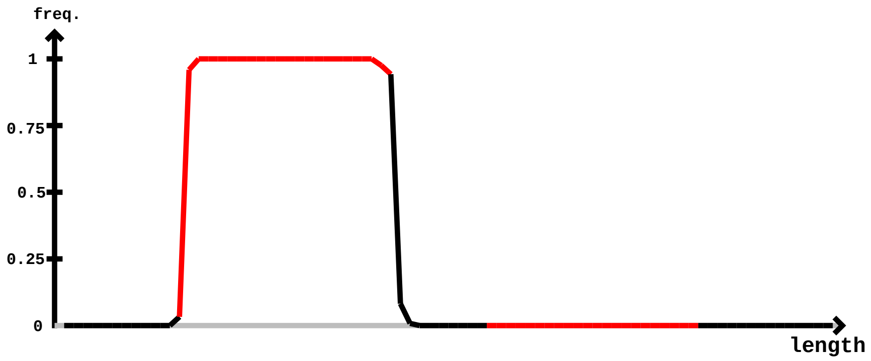


```
                                                     novel-nve-miR-70_star
               novel-nve-miR-70_guide
5'- ugaguugcgaaaacaaaauugcgggcgggacuugcucgcgaccagguaccgcgcgcaauuuuuuuuuuaaaaacucgua   -3'    exp
    .(((((..(((((.(((((((((.((((.(((((........))))).)))).))))))))).)))))....)))))...    reads      mm         sample
    ...........aacaaaauugcgggAgggacuug..............................................    1          1          seq
    ...........Gacaaaauugcgggcgggacuug..............................................    1          1          seq
    ...........aacaaaauugcgggcgggacuug..............................................    2          0          seq
    ............acaaaauugcgggcgggacu................................................    2          0          seq
    ............acaaaauugcgggcgggacA................................................    1          1          seq
    ............acaaaauugcgggcgggacuu...............................................    4          0          seq
    ............acaaaauugcgggcgggacuuU..............................................    1          1          seq
    ............acGaaauugcgggcgggacuug..............................................    1          1          seq
    ............acaaaauugcgggcgggacuuA..............................................    11         1          seq
    ............acaaaauugcgggcgggacuug..............................................    81         0          seq
    ............Gcaaaauugcgggcgggacuug..............................................    2          1          seq
    ............acaGaauugcgggcgggacuug..............................................    3          1          seq
    ............acaaaGuugcgggcgggacuug..............................................    1          1          seq
    ............Ucaaaauugcgggcgggacuug..............................................    1          1          seq
    ............acaaaauugcgggcgggacuugA.............................................    1          1          seq
    ............acaaaauugcgggcgggacuugU.............................................    3          1          seq
    ............acaaaauugcgggcgggacuugcu............................................    1          0          seq
    .............caaaauugcgggcgggacuugU.............................................    2          1          seq
    .............caaaauugcgggcgggacuugc.............................................    3          0          seq
```

miRBase precursor : novel-nve-miR-74
Total read count : 199
novel-nve-miR-74_guide read count : 199
novel-nve-miR-74_star read count : 0
remaining reads : 0

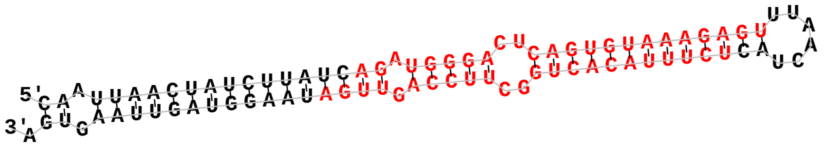

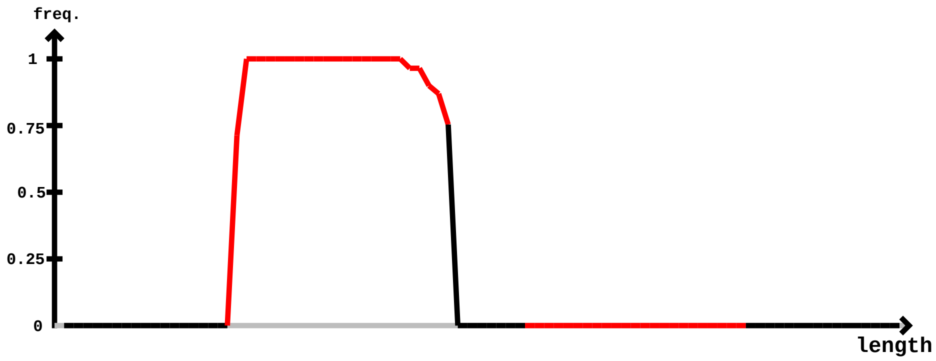


```
                                  novel-nve-miR-74_guide
                                                              novel-nve-miR-74_star
5'- caauuaacuaucuuaucagaugggacucaguguaaagaguuuaacuacucuuuacacuggcuuccaguugauaagguaguuaaguga   -3'    exp
    ((.((((((((((((((((.(((((..(((((((((((((......)))))))))))))..))))).)))))))))))))))).)).    reads     mm        sample
    .................agaugggacucaguguaa....................................................    7         0         seq
    .................agaugggacucaguguaaag..................................................    4         0         seq
    .................agaugggacucaguguaGaga.................................................    1         1         seq
    .................agaugggacucaguguaaaga.................................................    5         0         seq
    .................agaugggacucaguguaaagag................................................    14        0         seq
    .................agaugggacuGaguguaaagag................................................    1         1         seq
    .................agGugggacucaguguaaagag................................................    1         1         seq
    .................agaugggacCcaguguaaagag................................................    1         1         seq
    .................agaugggacucaguguaaagagC...............................................    14        1         seq
    .................Ggaugggacucaguguaaagagu...............................................    1         1         seq
    .................agaugggacGcaguguaaagagu...............................................    1         1         seq
    .................agauAggacucaguguaaagagu...............................................    1         1         seq
    .................agaugggacucaguguaaagagu...............................................    82        0         seq
    .................agaugggacucaguguaaagagA...............................................    4         1         seq
    .................agaAgggacucaguguaaagagu...............................................    2         1         seq
    .................agaugggacucaguguaaGgagu...............................................    1         1         seq
    .................agGugggacucaguguaaagagu...............................................    1         1         seq
    .................agaugggacucagugCaaagagu...............................................    1         1         seq
    ..................gaugggacucaguguaaag..................................................    9         0         seq
    ..................gaugggacucaguguaaagag................................................    5         0         seq
    ..................gauCggacucaguguaaagag................................................    1         1         seq
    ..................gaugggacucaguguaaagagC...............................................    4         1         seq
    ..................gaugggacucaguguaaagagu...............................................    32        0         seq
    ..................gaugggacucagAguaaagagu...............................................    1         1         seq
    ..................gaugggacuUaguguaaagagu...............................................    3         1         seq
    ..................gaugggacucaguguGaagagu...............................................    1         1         seq
    ..................Aaugggacucaguguaaagagu...............................................    1         1         seq
```

miRBase precursor : novel-nve-miR-78
Total read count : 87
novel-nve-miR-78_guide read count : 87
novel-nve-miR-78_star read count : 0
remaining reads : 0

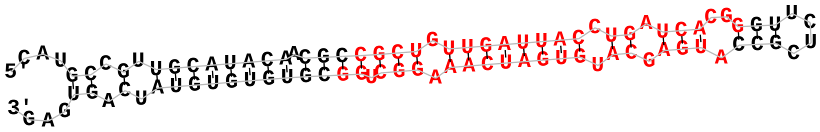

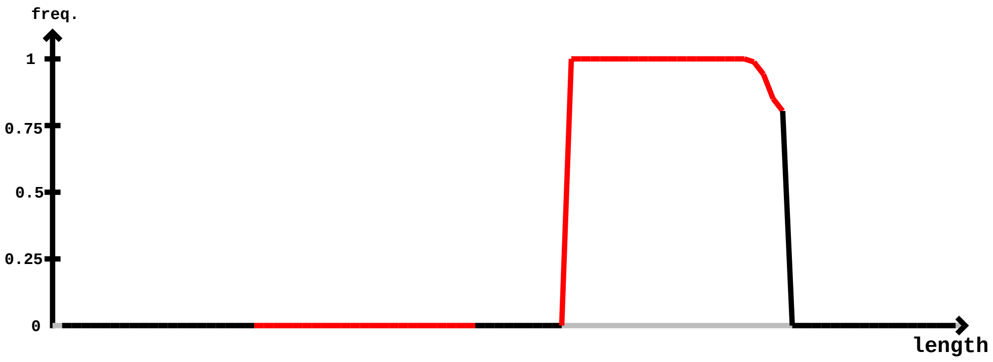

novel-nve-miR-78_star
novel-nve-miR-78_guide

```
5'- caugccguugcauacaacgccgcuguugauuaccugaucacggguucucgccaugagcaugugaucaaaggcuggcgugugugua ucagugag -3'   exp
    (((.....((((((((.(((((((.((((((((.((.(((..(((....))).))).)).)))))))).))).)))))))))))...)))..    reads   mm   sample
    ....................................................augagcaugugaucaaagg......................    1       0    seq
    ....................................................augagcaugugaucaaaggc.....................    3       0    seq
    ....................................................augGgcaugugaucaaaggc.....................    1       1    seq
    ....................................................augagcaugugaucaaaggcu....................    6       0    seq
    ....................................................augagcaugugaucaaaggcC....................    1       1    seq
    ....................................................Gugagcaugugaucaaaggcu....................    1       1    seq
    ....................................................augagcaugugaucaaaggcuU...................    1       1    seq
    ....................................................augagcaugugaucaaaggcug...................    3       0    seq
    ....................................................augUgcaugugaucaaaggcugg..................    1       1    seq
    ....................................................augagcGugugaucaaaggcugg..................    1       1    seq
    ....................................................augagcaugugaucaaaggcugC..................    1       1    seq
    ....................................................augagcaugugaucaaagAcugg..................    1       1    seq
    ....................................................augagcaugugaucaaaggcugU..................    3       1    seq
    ....................................................augagcaugugGucaaaggcugg..................    1       1    seq
    ....................................................augagcaugugaucaaaggcugA..................    8       1    seq
    ....................................................augagcaugugaucaaaggcugg..................    54      0    seq
```

miRBase precursor : novel-nve-miR-80
Total read count : 121
novel-nve-miR-80_guide read count : 121
novel-nve-miR-80_star read count : 0
remaining reads : 0

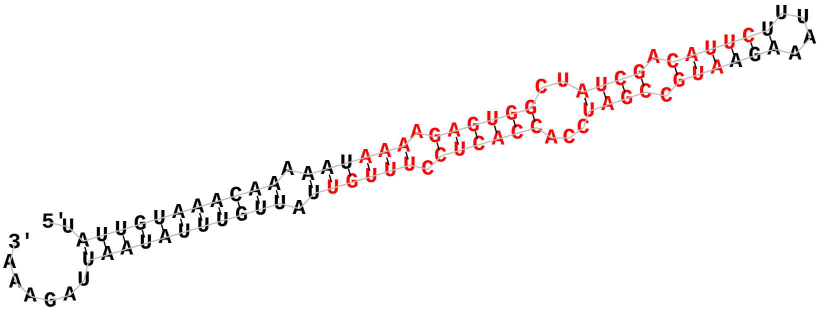

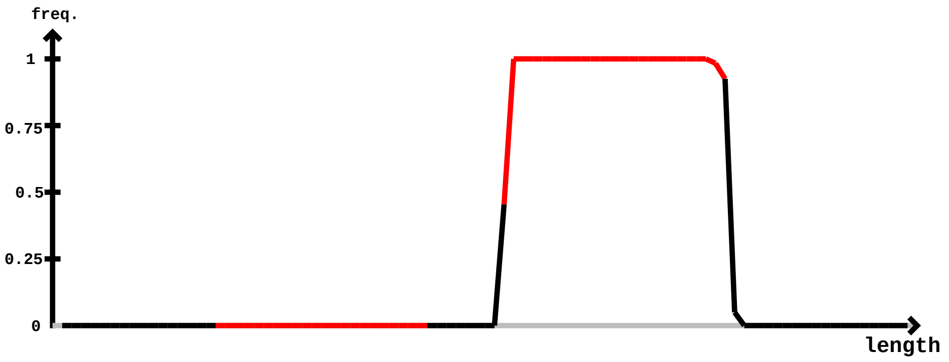

novel-nve-miR-80_star

novel-nve-miR-80_guide

```
5'- uauuguaaacaaaaauaaaagaguggcuaucgacauucuuuaaagaaugccgauccaccacuccuuuguuauuguuuauaauuagaaa   -3'    exp
    .((((((((((.((((((.((((((..((((.((((((....)))))).))))...)))))).)))))).))))))))))......    reads      mm         sample
    .............................................aaugccgauccaccacuccuuu.....................    2          0          seq
    .............................................aaugccgauccaccacuccuuug....................    3          0          seq
    .............................................aaugccgauccaccacuccuuuA....................    1          1          seq
    .............................................aaugccgauccaccacuccuuCgu...................    1          1          seq
    .............................................aaugccgauccaccacuccuuugG...................    1          1          seq
    .............................................aaugccgauccaccacuccuuugC...................    10         1          seq
    .............................................aaugccgauccaccacuccuuugA...................    1          1          seq
    .............................................aaugccgauccaccacuUcuuugu...................    1          1          seq
    .............................................aaugccgauccaccacuccuuugu...................    35         0          seq
    ..............................................augccgauccaccacuccuuGg....................    1          1          seq
    ..............................................augccgauccaccacuccuuug....................    2          0          seq
    ..............................................augccgauccaccacuccuuugC...................    8          1          seq
    ..............................................augccgauccaccacuccuuugG...................    2          1          seq
    ..............................................augccgaucGaccacuccuuugu...................    1          1          seq
    ..............................................augccgauccaccacuccuuugA...................    6          1          seq
    ..............................................augccgauccaccacuccuuugu...................    36         0          seq
    ..............................................aCgccgauccaccacuccuuugu...................    1          1          seq
    ..............................................augccgauccaccacCccuuugu...................    1          1          seq
    ..............................................Gugccgauccaccacuccuuugu...................    2          1          seq
    ..............................................augccgauccaccacuccuuuguu..................    5          0          seq
    ..............................................augccgauccaAcacuccuuuguu..................    1          1          seq
```

miRBase precursor : novel-nve-miR-81
Total read count : 90
novel-nve-miR-81_guide read count : 90
novel-nve-miR-81_star read count : 0
remaining reads : 0

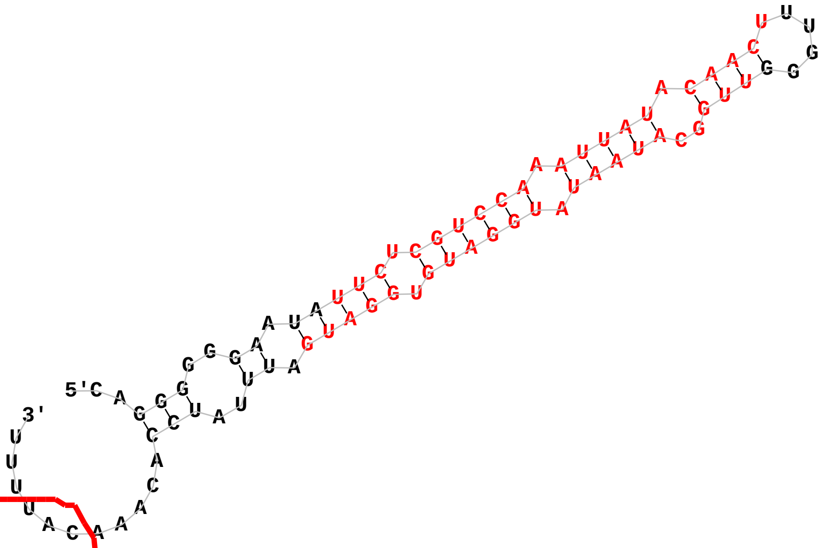
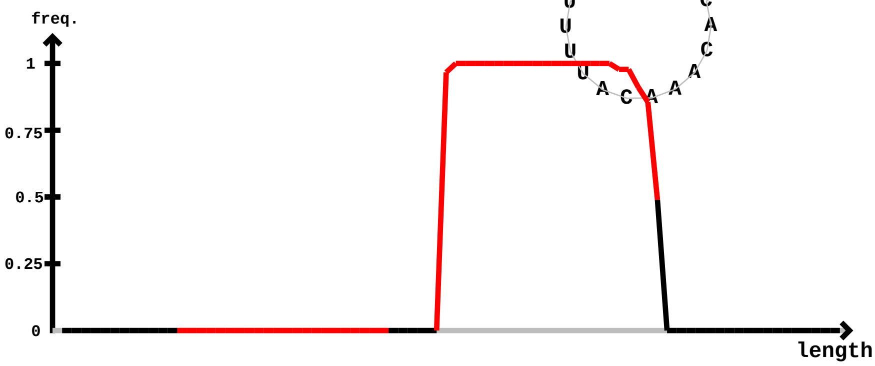

novel-nve-miR-81_star

novel-nve-miR-81_guide

```
5'- caggggggaauauucucguccaaauuauacaacuuuggguuggcauaauauggauguggaugauuuauccacaaacauuuu   -3'    exp
    ..(((..((.(((((.((((((.(((((.((((.....))))..))))).)))))).))))).))..)))...........    reads      mm         sample
    .......................................uuggUauaauauggaugu........................    1          1          seq
    .......................................uuggcauaauauggaugu........................    1          0          seq
    .......................................uuggcauaauauggaugugg......................    5          0          seq
    .......................................uuggcauaauauggaugugA......................    1          1          seq
    .......................................uuggcauUauauggaugugga.....................    3          1          seq
    .......................................uuggcauaauauggaugugga.....................    2          0          seq
    .......................................uuCgcauaauauggauguggau....................    1          1          seq
    .......................................uuAgcauaauauggauguggau....................    1          1          seq
    .......................................uuggcauaauauggauguggau....................    23         0          seq
    .......................................uuggcauaGuauggauguggau....................    1          1          seq
    .......................................uuggcauaauauggauguggaC....................    4          1          seq
    .......................................uuggcauaauauggauguggauC...................    10         1          seq
    .......................................uuggcauaauauggauguggauU...................    34         1          seq
    ........................................uggcauaauauggauAuggau....................    1          1          seq
    ........................................uggcauaauauggauguggau....................    2          0          seq
```

miRBase precursor : novel-nve-miR-82
Total read count : 226
novel-nve-miR-82_guide read count : 226
novel-nve-miR-82_star read count : 0
remaining reads : 0

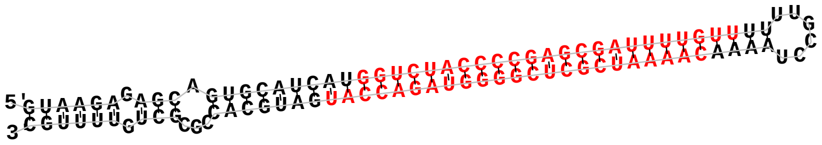

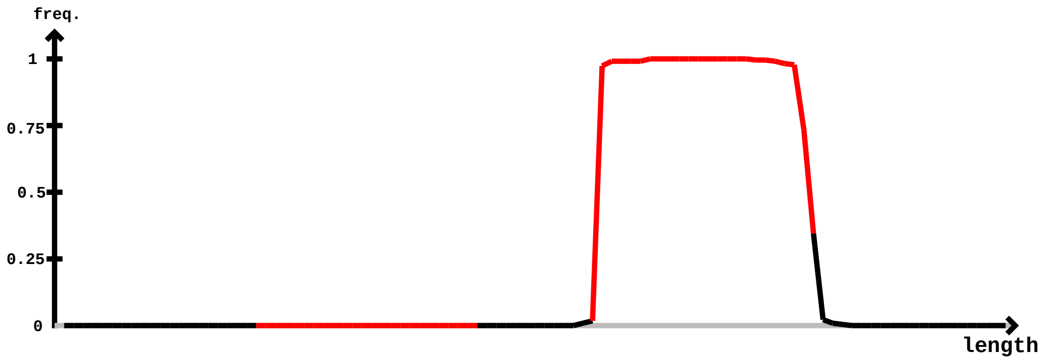


```
                                                       novel-nve-miR-82_guide
                    novel-nve-miR-82_star
5'- guaagagagcagugcaucauggucuaccccgagcgauuuuguuuuuugccuaaaacaaaaucgcucgggguagaccaugaugcaccgcgcuguuuugc  -3'    exp
    (((((.(((.(((((((((((((((((((((((((((((((((((......)))))))))))))))))))))))))))))))))))...))).)))))   reads   mm   sample
    .....................................................aacaaaaucgcucggggu...........................   1       0    seq
    .....................................................aacaaaaucgcucgggguag.........................   1       0    seq
    ......................................................acaaaaucgcucgggguagacU......................   1       1    seq
    ......................................................acaaaaucgcucgggguagacA......................   1       1    seq
    .......................................................caaaaucgcucgggguaga........................   2       0    seq
    .......................................................caaaaucgcucgggguagac.......................   1       0    seq
    .......................................................caaaaucgcuUgggguagacc......................   2       1    seq
    .......................................................caaaaucgcucgggguagacA......................   5       1    seq
    .......................................................caaaaucgcucgggguagacG......................   2       1    seq
    .......................................................caaaaucgcucgggguGgacc......................   1       1    seq
    .......................................................caaaaucgcucgggguagacc......................   17      0    seq
    .......................................................caaaaucgcucgggguagacU......................   25      1    seq
    .......................................................caaaaucgcucgggguagacca.....................   69      0    seq
    .......................................................caaaaucgcucgggguagaccC.....................   2       1    seq
    .......................................................caaaaucgcucgggguagCcca.....................   1       1    seq
    .......................................................caaaaucgcucgggguagaccU.....................   4       1    seq
    .......................................................caaaaucUcucgggguagacca.....................   1       1    seq
    .......................................................caaaaucgcucgggguGgacca.....................   4       1    seq
    .......................................................Uaaaaucgcucgggguagacca.....................   1       1    seq
    .......................................................caaaaucgcucCggguagacca.....................   1       1    seq
    .......................................................caaaaucgcucgggguagaccG.....................   3       1    seq
    .......................................................caaaaucgcAcgggguagaccau....................   1       1    seq
    .......................................................caaaaucgcucgggguagaccaC....................   7       1    seq
    .......................................................caaaaucgcGcgggguagaccau....................   1       1    seq
    .......................................................caaaaucgcCcgggguagaccau....................   1       1    seq
    .......................................................caaaaucgcucgggguagGccau....................   1       1    seq
    .......................................................caaaaucgcucgggguagaccau....................   56      0    seq
    .......................................................caaaGucgcucgggguagaccau....................   2       1    seq
    .......................................................caaaaucgcucgggguGgaccau....................   3       1    seq
    .......................................................caaaaucgcucgggguagaccauU...................   2       1    seq
    .......................................................caaaaucgcucgggguagaccauC...................   1       1    seq
    ........................................................Caaaucgcucgggguagacc......................   1       1    seq
    ........................................................aaaaucgcucgggguagacca.....................   1       0    seq
    ........................................................Caaaucgcucgggguagacca.....................   1       1    seq
```

```
                                                       novel-nve-miR-82_guide
                    novel-nve-miR-82_star
guaagagagcagugcaucauggucuaccccgagcgauuuuguuuuuugccuaaaacaaaaucgcucgggguagaccaugaugcaccgcgcuguuuugc

........................................................aaaaucgcucgggguagaccau....................    1    0    seq
............................................................ucgcucgggguagaccauga..................    1    0    seq
............................................................ucgcucgggguagaccaugau.................    1    0    seq
```

miRBase precursor : novel-nve-miR-93
Total read count : 242
novel-nve-miR-93_guide read count : 242
novel-nve-miR-93_star read count : 0
remaining reads : 0

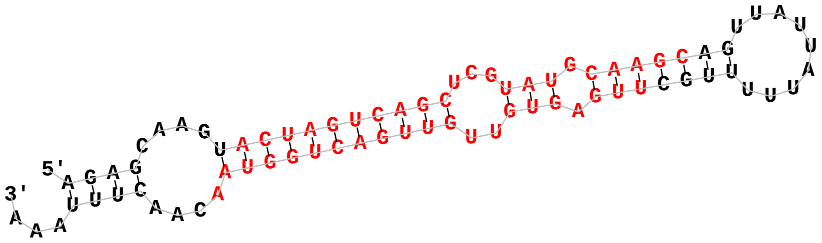

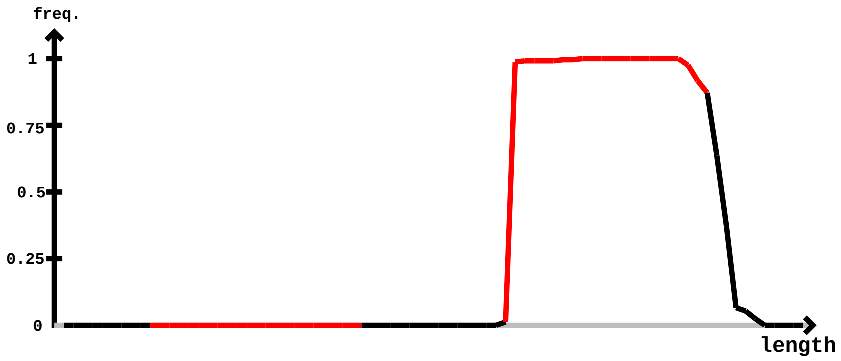


```
                 novel-nve-miR-93_star
                                                     novel-nve-miR-93_guide
5'- agagcaaguacuagucagcucguaugcaagcaguuauuauuuuugcuugaguguuguugacugguaacaacuuuaaa  -3'   exp
    ((((....(((((((((((...(((.(((((((.........))))))).)))..)))))))))))....))))...   reads    mm    sample
    .............................................cuugaguguuguugacugU.............   1        1     seq
    .............................................cuugaguguuguugacugguaa..........   1        0     seq
    .............................................cuugaguguuguugacugguaU..........   1        1     seq
    ..............................................uugaguguuguugacugg.............   2        0     seq
    ..............................................uugaguguuguugacugU.............   1        1     seq
    ..............................................uugaguguuguugacugA.............   2        1     seq
    ..............................................uugaguguuguugacuggC............   4        1     seq
    ..............................................uugaguguuguugacuggA............   1        1     seq
    ..............................................uugaguguuguugacuggu............   9        0     seq
    ..............................................uugaguguuguugacuggua...........   10       0     seq
    ..............................................uugaguguuguugacugUua...........   1        1     seq
    ..............................................uugaguguuguugacugguaU..........   5        1     seq
    ..............................................uugaguguuguuUacugguaa..........   1        1     seq
    ..............................................Gugaguguuguugacugguaa..........   1        1     seq
    ..............................................uugaguguuguugacugguaC..........   1        1     seq
    ..............................................uugGguguuguugacugguaa..........   1        1     seq
    ..............................................uugagugCuguugacugguaa..........   1        1     seq
    ..............................................uugaguguuguugacugguaa..........   40       0     seq
    ..............................................uugaguguuguugacugguaG..........   3        1     seq
    ..............................................uugaguguuguugacugCuaa..........   1        1     seq
    ..............................................uCgaguguuguugacugguaa..........   1        1     seq
    ..............................................uugaguguuAuugacugguaac.........   1        1     seq
    ..............................................uugaguguuguugacugguaaU.........   13       1     seq
    ..............................................uCgaguguuguugacugguaac.........   1        1     seq
    ..............................................uugaguguuguugacugguGac.........   1        1     seq
    ..............................................uugaguguuguugacugguaac.........   46       0     seq
    ..............................................uugaguguuguugacugguaUc.........   1        1     seq
    ..............................................uAgaguguuguugacugguaac.........   1        1     seq
    ..............................................uugaguguuguugacugguaacC........   9        1     seq
    ..............................................uugaguguuguugacugguaacG........   4        1     seq
    ..............................................uugaguguuguugacugguaaca........   27       0     seq
    ..............................................uugaguguuguugacugguaacU........   33       1     seq
    ..............................................uugaguguuguugacugguaacaG.......   1        1     seq
    ..............................................uugaguguuguugacugguaacaa.......   1        0     seq
```

```
         novel-nve-miR-93_star
                                              novel-nve-miR-93_guide
agagcaaguacuagucagcucguaugcaagcaguuauuauuuuugcuugaguguuguugacugguaacaacuuuaaa

..............................................uugaAuguuguugacugguaacaa.......    1          1          seq
..............................................uugaguguuguugacugguaacaaU......    5          1          seq
..............................................uugaguguuguugacugguaacaac......    2          0          seq
..............................................uugaguguuguugacugguaacaacA.....    1          1          seq
..............................................uugaguguuguugacugguaacaacu.....    3          0          seq
...............................................ugaguguuguugacugguaacaacu.....    1          0          seq
...................................................uguuguugacugguaaca........    1          0          seq
.....................................................uuguugacugguaacaacu.....    1          0          seq
```

miRBase precursor : novel-nve-miR-95
Total read count : 85
novel-nve-miR-95_guide read count : 85
novel-nve-miR-95_star read count : 0
remaining reads : 0

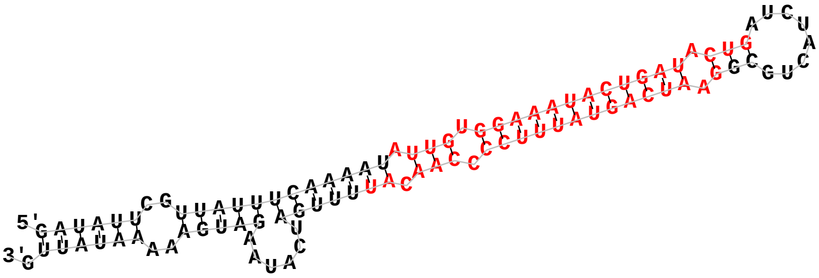

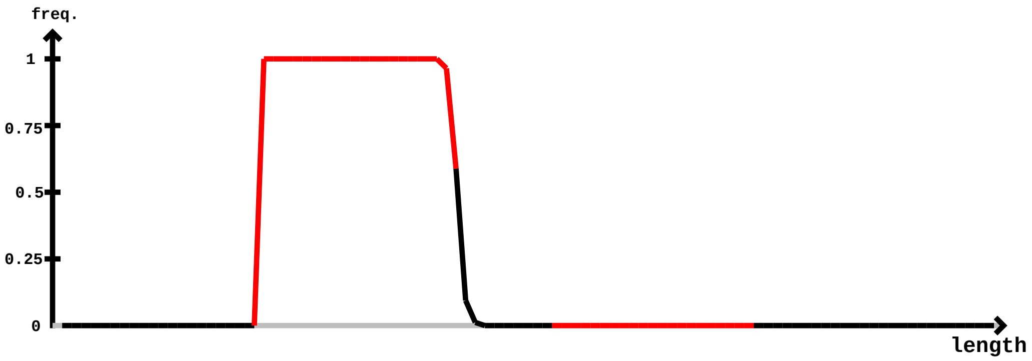


```
                                                         novel-nve-miR-95_star
                     novel-nve-miR-95_guide
5'- gauauucguuauuucaaaauauuguggaaauacugauacugaucuacugcggaaucaguauuuccccaacauuuugaucauaagaugaaaaauauug  -3'  exp
    ((((((..((((((((((((.(((.((((((((((((.(((........))).))))))))))))).))).)))))))......)))))..)))))).    reads  mm  sample
    ....................auuguggaaauacugauaU..........................................................    1      1   seq
    ....................auuguggaaauacugauac..........................................................    2      0   seq
    ....................auuguggaaauacugauacu.........................................................    23     0   seq
    ....................auuguggaaauacugauacA.........................................................    3      1   seq
    ....................auuguggaaauacugauacC.........................................................    5      1   seq
    ....................Guuguggaaauacugauacu.........................................................    1      1   seq
    ....................auuguggaaauacugauacug........................................................    33     0   seq
    ....................auuguggGaauacugauacug........................................................    1      1   seq
    ....................auuguggaaauacugauacCg........................................................    1      1   seq
    ....................auuguggaaauacugauacuA........................................................    7      1   seq
    ....................auuguggaaauacugauacugC.......................................................    1      1   seq
    ....................auuguggaaauacugauacugU.......................................................    3      1   seq
    ....................auuguggaaauacugauacuga.......................................................    3      0   seq
    ....................auuguggaaauacugauacugau......................................................    1      0   seq
```

miRBase precursor : novel-nve-miR-97
Total read count : 42
novel-nve-miR-97_guide read count : 42
novel-nve-miR-97_star read count : 0
remaining reads : 0

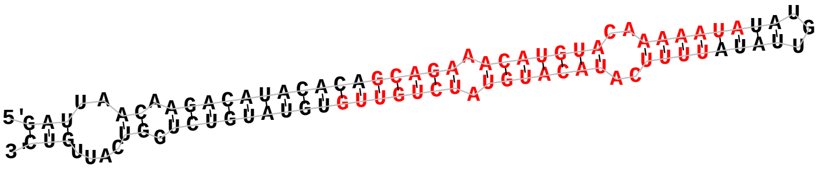

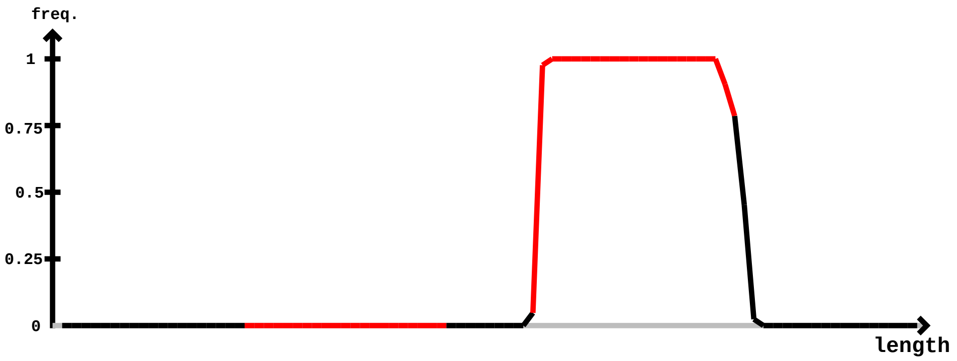


```
                                   novel-nve-miR-97_star
                                                        novel-nve-miR-97_guide
5'- gauuaacaagacauacacagcagaaacauguacaaaaauauauguuauauuuucauacauguaucuguuguguaugucuggucauuguc   -3'    exp
    ((((....((((((((((((((((.(((((((..((((((((...))))))))..))))))).))))))))))))))))))))......    reads      mm         sample
    ................................................auuuucauacauguaucugC.....................    1          1          seq
    ................................................auuuucauacauguaucuguu....................    1          0          seq
    .................................................uuuucauacauguaucugA.....................    1          1          seq
    .................................................uuuucauacauguaucugu.....................    1          0          seq
    .................................................uuuucauacauguaucugC.....................    1          1          seq
    .................................................uuuuUauacauguaucuguu....................    1          1          seq
    .................................................uuuucauacauguaucuguu....................    3          0          seq
    .................................................uuuuUauacauguaucuguug...................    1          1          seq
    .................................................uuuucauacauguaucuguug...................    11         0          seq
    .................................................uuuucauacauguaucuguuU...................    2          1          seq
    .................................................uuuucauacauguaucuguugu..................    10         0          seq
    .................................................uuuucauacauguaucuguugA..................    2          1          seq
    .................................................uuuucauacauguaucuguuUu..................    1          1          seq
    .................................................uuuucauacauguaucuguugC..................    4          1          seq
    .................................................uuuucauacauguaucuguuguU.................    1          1          seq
    ..................................................uuucauacauguaucuguugu..................    1          0          seq
```

Provisional ID		novel-nve-miR-98
Score total	:	1.6
Score for star read(s)	:	-1.3
Score for read counts	:	0
Score for mfe	:	1.9
Score for randfold	:	1.6
Score for cons. seed	:	-0.6
Total read count	:	3226
Mature read count	:	3226
Loop read count	:	0
Star read count	:	0

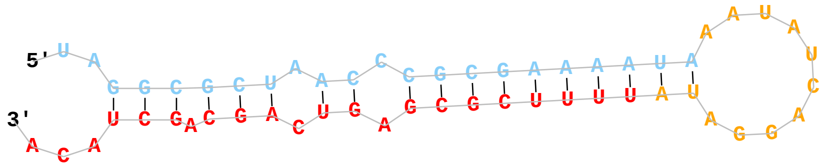

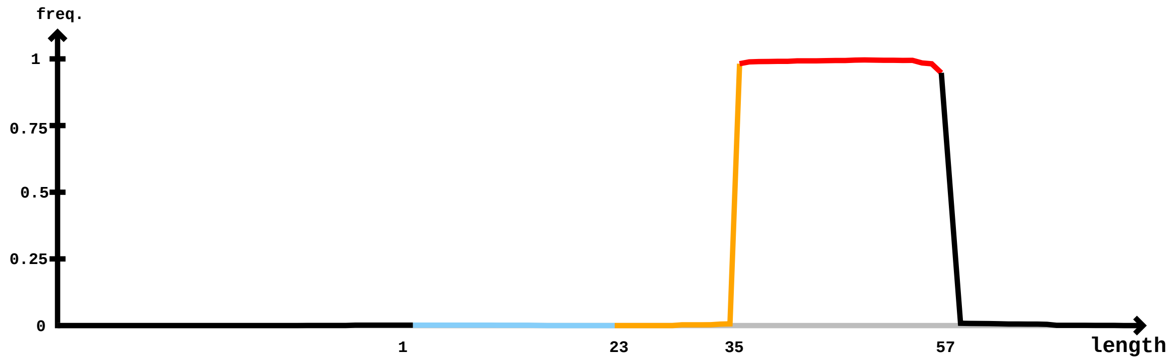


```
                                   Star                              Mature
5'- gaagcacagauuugauuaccaacauagccuacguguaggcgcuaacccgcgaaaauaaauaucaggauauuuucgcgagucagcagcuacacguaggcuauuaccaacaca  -3'   exp
    .......................((((((((((((((((..(((.((.(((((((((..........))))))))).)).)))..)))))))))))))))).........   reads   mm   sample
    ........................uagccuacguguagCcgcua...................................................................   1   1   seq
    .............................uacguguaggcgcuaaccG...............................................................   2   1   seq
    .............................uacguguaggcgcuaacccA..............................................................   2   1   seq
    ...............................................................aggauauuuucgcgaguc..............................   4   0   seq
    ...............................................................aggauauuuucgcgagucag............................   1   0   seq
    ...............................................................aggauauuuucgcgagucaA............................   1   1   seq
    ...............................................................aggauauuuucgcgagucaC............................   1   1   seq
    ...............................................................aggauauuuucgcgagucagc...........................   1   0   seq
    ...............................................................aggauauuuucgcgagucagU...........................   1   1   seq
    ..................................................................auauuuucgcgagucagcagcu.......................   1   0   seq
    ...................................................................uauuuucgcgagucagcGg.........................   2   1   seq
    ...................................................................uauuuucgcgagucagcGgc........................   1   1   seq
    ...................................................................uauuuucgcgagucagcGgcu.......................   5   1   seq
    ....................................................................auuuucgcgagucagcagcuac.....................   1   0   seq
    ....................................................................auuuucgcgagucagcagcuacU....................   2   1   seq
    .....................................................................uuuucgcgagucagcagU........................   1   1   seq
    .....................................................................uuuucgcgagucagcCgcu.......................   1   1   seq
    .....................................................................uuuucgcgagucagcagcu.......................   15   0   seq
    .....................................................................uAuucgcgagucagcagcu.......................   1   1   seq
    .....................................................................uuuCcgcgagucagcagcu.......................   1   1   seq
    .....................................................................uuuucgcgagucagcagcA.......................   3   1   seq
    .....................................................................uuuucgcgagucGgcagcu.......................   1   1   seq
    .....................................................................uuuucgcgagucagcagcG.......................   4   1   seq
    .....................................................................uuuucgcgagucagcagcuU......................   1   1   seq
    .....................................................................uuuucgcgagucagcagcuC......................   1   1   seq
    .....................................................................uuuucgcgagucagcagcua......................   7   0   seq
    .....................................................................uuuucgcgagucagcagcGa......................   1   1   seq
    .....................................................................uuuucgcgagucagcGgcuac.....................   1   1   seq
    .....................................................................uuuucgcgagucagcagcuaA.....................   5   1   seq
    .....................................................................uuuucgcAagucagcagcuac.....................   1   1   seq
    .....................................................................uuuucgcgagCcagcagcuac.....................   1   1   seq
    .....................................................................uuuucgcgagucagcagcuaU.....................   44   1   seq
    .....................................................................uuuucgcgagucagcagcuac.....................   56   0   seq
    .....................................................................uuuucgcgagucagcagcuaG.....................   1   1   seq
```

Star Mature

```
gaagcacagauuugauuaccaacauagccuacguguaggcgcuaacccgcgaaaauaaauaucaggauauuuucgcgagucagcagcuacacguaggcuauuaccaacaca
.....................................................................uuuucgcgagucagcagcuacG....................    257    1    seq
.....................................................................uuuucgcgagucagcagcuaca....................    200    0    seq
.....................................................................uuuucgcgagucagcGgcuaca....................    1      1    seq
.....................................................................uuuuGgcgagucagcagcuaca....................    1      1    seq
.....................................................................uuuucgcgaUucagcagcuaca....................    1      1    seq
.....................................................................Cuuucgcgagucagcagcuaca....................    1      1    seq
.....................................................................uuuucgcgagucagcaAcuaca....................    1      1    seq
.....................................................................uAuucgcgagucagcagcuaca....................    1      1    seq
.....................................................................uuuucgcgagucagcagcuGca....................    1      1    seq
.....................................................................uuuucgcgGgucagcagcuaca....................    4      1    seq
.....................................................................Guuucgcgagucagcagcuaca....................    1      1    seq
.....................................................................uuuucgcgagucagcagcuacU....................    2080   1    seq
.....................................................................uuuucgcgagucUgcagcuaca....................    1      1    seq
.....................................................................uuuucgcgagucagcagcuacC....................    478    1    seq
.....................................................................uuuuAgcgagucagcagcuaca....................    2      1    seq
.....................................................................uuuucgcgagucagcagcuacaU...................    1      1    seq
.....................................................................uuuucgcgagucagcagcuacUc...................    6      1    seq
......................................................................uuucgcgagucagcagcuac.....................    1      0    seq
......................................................................uuucgcgagucagcagcuacG....................    6      1    seq
......................................................................uuucgcgagucagcagcuacU....................    9      1    seq
......................................................................uuucgcgagucagcagcuacC....................    6      1    seq
.......................................................................uucgcgagucagcagcuacU....................    4      1    seq
........................................................................ucgcgagucagcaAcuacacguaggcu............    1      1    seq
.........................................................................cgcgagucagcagcuacacU..................    2      1    seq
...........................................................................cgagucagcaAcuacacgu.................    1      1    seq
...........................................................................cgagucagcagcuaUacgua................    1      1    seq
...........................................................................cgagucagcaAcuacacguag...............    2      1    seq
...........................................................................cgagucagcaAcuacacguagg..............    2      1    seq
..............................................................................gucagcaAcuacacguagg..............    1      1    seq
..............................................................................gucagcGgcuacacguaggcuauu.........    1      1    seq
...............................................................................ucagcGgcuacacguaggcuauu.........    2      1    seq
.................................................................................agcagcuacacguaggcuaC..........    2      1    seq
.................................................................................agcGgcuacacguaggcuauu.........    7      1    seq
..................................................................................gcGgcuacacguaggcuauu.........    2      1    seq
...................................................................................cUgcuacacguaggcuau..........    1      1    seq
.......................................................................................uacacguaggcuauuaccU.....    1      1    seq
.......................................................................................uacacguaggcuauuaccaaca..    1      0    seq
.......................................................................................uacacguaggcuauuaccaacU..    1      1    seq
```

miRBase precursor : novel-nve-miR-99-1
Total read count : 75
novel-nve-miR-99-1_guide read count : 75
novel-nve-miR-99-1_star read count : 0
remaining reads : 0

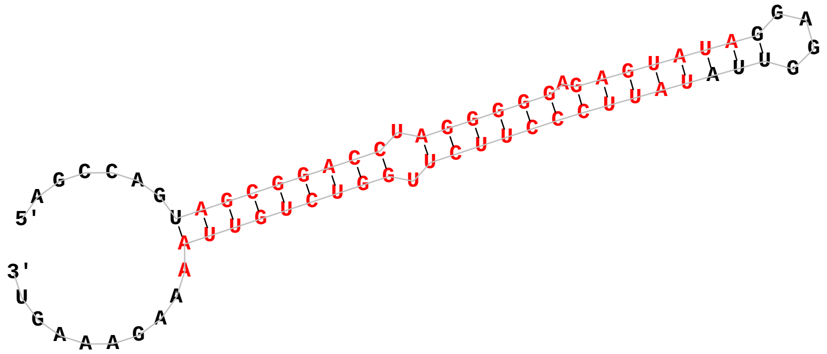

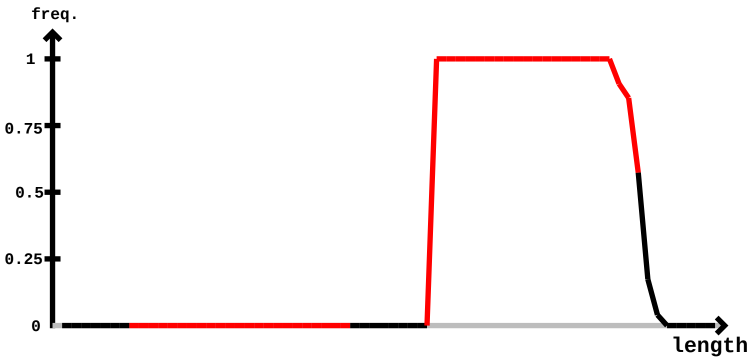


```
                                      novel-nve-miR-99-1_guide
       novel-nve-miR-99-1_star
5'- agccaguagcggaccuagggggagaguauaggagguuauauucccuucuuggucuguuaaaagaaagu  -3'    exp
    ......((((((((.((((((.((((((((....)))))))))))))).))))))))...........    reads      mm         sample
    ......................................uauucccuucuuggucugC...........    2          1          seq
    ......................................uauuUccuucuuggucugu...........    1          1          seq
    ......................................uauucccuucuuggucugu...........    4          0          seq
    ......................................uauucccuucuuggucuguu..........    4          0          seq
    ......................................uauucccuucCuggucuguua.........    1          1          seq
    ......................................uauucccuucuuggucuguuC.........    3          1          seq
    ......................................uauucccuucuuggucuguua.........    7          0          seq
    ......................................uauucccuucuuggucuguuU.........    9          1          seq
    ......................................uCuucccuucuuggucuguua.........    1          1          seq
    ......................................uauucccuucuuggucuguuaU........    6          1          seq
    ......................................uauucccuucuuggCcuguuaa........    1          1          seq
    ......................................uauucccuucuuggucuguuUa........    1          1          seq
    ......................................uauucccuucuuggucuguuaa........    22         0          seq
    ......................................uauucccuucuuggucuguuaaU.......    4          1          seq
    ......................................uauucccuucuuggucuguuaaa.......    3          0          seq
    ......................................uauucccuucuuggucuguuaaC.......    1          1          seq
    ......................................uauucccuucuuggucuguuaUa.......    1          1          seq
    ......................................uaGucccuucuuggucuguuaaa.......    1          1          seq
    ......................................uauucccuucuuggucuguuaaaU......    3          1          seq
```

miRBase precursor : novel-nve-miR-100-1
Total read count : 141
novel-nve-miR-100-1_guide read count : 141
novel-nve-miR-100-1_star read count : 0
remaining reads : 0

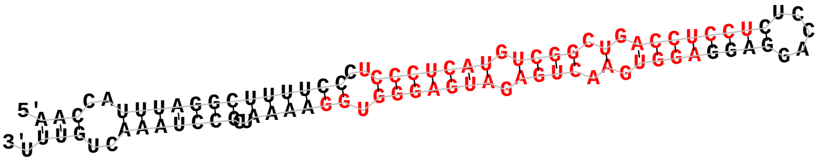

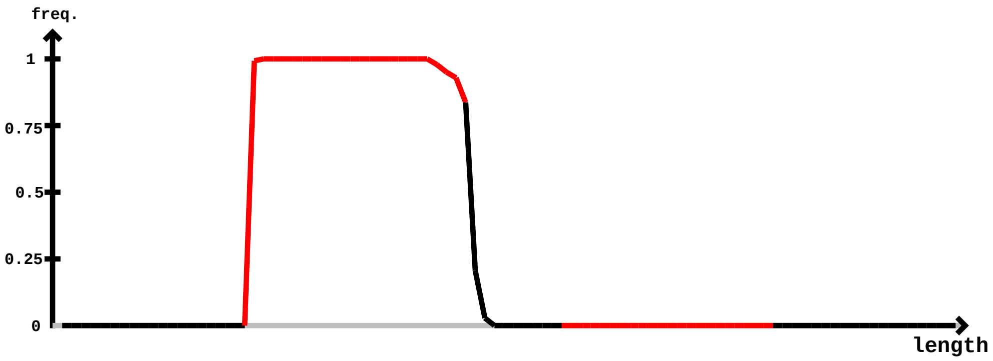


```
                   novel-nve-miR-100-1_guide
                                                                 novel-nve-miR-100-1_star
5'- aaccauuuaggcuuuucccucccucaugucggcugaccuccucuccaggaggaggugaacugagaugagggugg aaaaucgccuaaacuguuu   -3'    exp
    (((..(((((((((((((..(((((((.((((.(.((((((((.....)))))))).).)))).))))))).))))))..)))))))..))).   reads   mm   sample
    ...................ucccucaugucggcugacA.......................................................   1       1    seq
    ...................ucccucaugucggcugacU.......................................................   1       1    seq
    ...................ucccucaugucggcugacc.......................................................   1       0    seq
    ...................ucccucaugucggcugaccu......................................................   3       0    seq
    ...................ucccucaugucggcugaAcu......................................................   1       1    seq
    ...................ucccucaugucggcugaccuc.....................................................   2       0    seq
    ...................ucccucaugucggcugaccuU.....................................................   1       1    seq
    ...................ucccucaugucggcugaccucc....................................................   7       0    seq
    ...................ucccucaugucggcugaccucA....................................................   2       1    seq
    ...................ucccucaugucggcugCccucc....................................................   1       1    seq
    ...................ucccucaugucggcugaccucG....................................................   1       1    seq
    ...................ucccucaugucggcugaccucU....................................................   2       1    seq
    ...................ucccucaugucAgcugaccuccu...................................................   2       1    seq
    ...................ucccucaugucggcugaccuccA...................................................   6       1    seq
    ...................ucccucaugucggcugaccuccG...................................................   5       1    seq
    ...................ucccucaugucggcugaccuccu...................................................   59      0    seq
    ...................ucccucaugucggcugaccuGcu...................................................   1       1    seq
    ...................ucccucaugucggcugaccuccC...................................................   15      1    seq
    ...................ucccucaugucggcugCccuccuc..................................................   1       1    seq
    ...................ucccucaugucAgcugaccuccuc..................................................   1       1    seq
    ...................ucccucaugucggcugaccuccuc..................................................   8       0    seq
    ...................ucccucaugucggcugaccuccuU..................................................   15      1    seq
    ...................ucccucaugucggcugaccuccuUu.................................................   4       1    seq
    ....................Uccucaugucggcugaccuccu...................................................   1       1    seq
```

miRBase precursor : novel-nve-miR-101-1
Total read count : 86
novel-nve-miR-101-1_guide read count : 86
novel-nve-miR-101-1_star read count : 0
remaining reads : 0

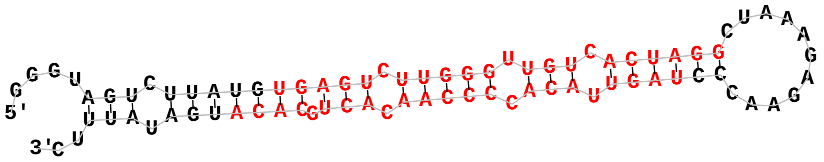

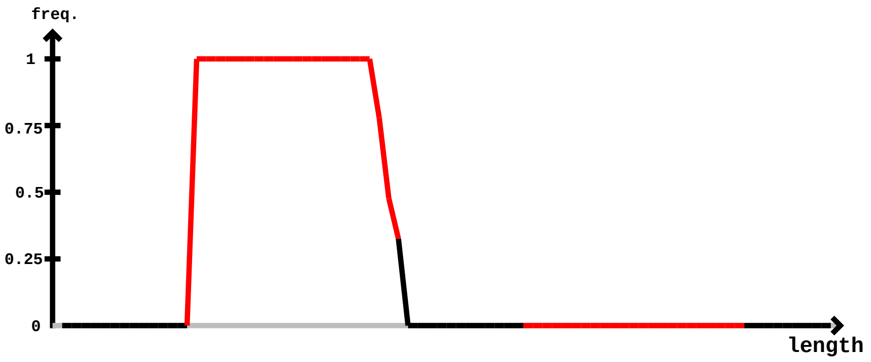


```
                                                  novel-nve-miR-101-1_star
                novel-nve-miR-101-1_guide
5'- ggguagucuuaugugagucuuggguugucacuaggcuaaagagaacccuaguuacaccccaacacugcacaugauauuuc   -3'    exp
    ........((((((((((.(((((.(((.((((((...........)))))).))).))))).))).)))))))......    reads      mm         sample
    .............ugagucuuggguugucacC................................................    1          1          seq
    .............ugagucuuggguugucacA................................................    2          1          seq
    .............ugagucuAggguugucacu................................................    1          1          seq
    .............ugagucuuggguugucacG................................................    1          1          seq
    .............ugagucuuggguugucacu................................................    14         0          seq
    .............Cgagucuuggguugucacua...............................................    1          1          seq
    .............ugagucuuggguuAucacua...............................................    1          1          seq
    .............ugagucuuggguugucacuC...............................................    1          1          seq
    .............ugagucuuggguugucacua...............................................    20         0          seq
    .............ugagucuuggguugucacuU...............................................    1          1          seq
    .............ugagucuuggguCgucacua...............................................    1          1          seq
    .............ugagucuuggguAgucacua...............................................    1          1          seq
    .............ugagucuuggguugucacuaU..............................................    2          1          seq
    .............ugagucuuggguugucacuag..............................................    11         0          seq
    .............ugagucuuggguugucacuagA.............................................    2          1          seq
    .............ugagucuCggguugucacuagg.............................................    1          1          seq
    .............ugagucuuggguugucacuGgg.............................................    1          1          seq
    .............ugagucuuggguugucacuagg.............................................    23         0          seq
    .............ugagucuuggguugucacuagU.............................................    1          1          seq
```
